# Comparing decentralized machine learning and AI clinical models to local and centralized alternatives: a systematic review

**DOI:** 10.1038/s41746-025-02329-z

**Published:** 2026-02-14

**Authors:** José Miguel Diniz, Henrique Vasconcelos, Rita Rb-Silva, Carolina Ameijeiras-Rodriguez, Daniel Rodrigues, Pedro Ramos, António Tomás, Yu Gao, Júlio Souza, Alberto Freitas

**Affiliations:** 1 RISE-Health, MEDCIDS, Faculty of Medicine, University of Porto, Porto, Portugal; 2 ULS São José, Lisbon, Portugal; 3 NOVA National School of Public Health (NOVA NSPH), Lisbon, Portugal; 4 Research Centre of the Portuguese Institute of Oncology of Porto (CI-IPOP), Porto, Portugal; 5 Institute of Engineering – Polytechnic of Porto, Porto, Portugal

**Keywords:** Machine learning, Predictive medicine, Computational models, Prognosis, Experimental models of disease

## Abstract

This systematic review evaluates how decentralized learning (DL) approaches—e.g., federated learning, swarm learning, ensemble–compare with traditional models in healthcare applications. We searched eight databases (01/2012 to 03/2024), screening 165,010 studies with two independent reviewers. Analysis included 160 articles comprising 710 DL models and 8149 performance comparisons across clinical domains, predominantly in oncology, COVID-19, and neurological diagnostics. In paired comparisons, centralized learning (CL) demonstrated advantages in threshold-dependent metrics (78% favourability for accuracy and Dice score with large effect sizes), while DL achieved comparable performance in ranking metrics (51% centralized favourability for AUROC with small effect size). DL consistently outperformed local learning (LL) across all metrics, particularly precision (86% favourability) and accuracy (83% favourability). Clinical threshold analysis (≥0.80 performance) revealed that CL rescued DL viability in up to 18% of comparisons, though when both achieved clinical viability, improvements typically represented “excellent versus acceptable” performance (median difference of 0.7–1.5pp) rather than “acceptable versus inadequate.” DL rescued LL viability with substantial improvements (median difference of 7.6–27pp). These findings demonstrate DL offers clinically acceptable alternatives for privacy-constrained contexts, with implementation decisions balancing marginal performance trade-offs against regulation (e.g., GDPR, AI Act) and application. Future research requires standardized privacy-performance reporting.

## Introduction

In recent decades, health systems worldwide have been facing unprecedented profound challenges. The epidemiological transition has intensified care needs and costs^1–4^, while technological advancements and pharmaceutical innovations have driven increasing expenditures^4,5^. Simultaneously, these systems still struggle to achieve and ensure universal coverage^6,7^, while facing increasing critical workforce shortages and limited investments and action across levels of prevention and health determinants^8^. These problems demand innovative approaches to improve the quality of care, while optimizing resources, particularly in clinical decision-making tasks (e.g., establishing diagnoses, prescribing therapies, offering prognoses).

Against this backdrop, machine learning systems and artificial intelligence (AI) technologies have been proposed as methods to address these demands. The convergence of ubiquitous digital systems, large-scale data, and advanced computational capabilities creates a unique opportunity to address the health domain’s most pressing demands.

However, despite these technological advances, healthcare systems have not yet been successful in developing and implementing effective solutions. As computational capability increases, the primary bottleneck to data science may lie in accessing and utilizing high-quality health data^9,10^, something particularly challenging in health-related domains. Public and anonymized databases are valuable resources, but they often lack external validity for developing robust healthcare applications. In contrast, real-world data (RWD), the clinical evidence derived from data collected during routine healthcare delivery, may be a more adequate and representative data source^11^.

While RWD offers potential for developing representative and generalizable models, the implementation of machine learning systems using these data faces multiple barriers. Legal and regulatory frameworks mandate increasingly stringent technical safeguards for data collection, maintenance, analysis, and disposal. Operational challenges include interoperability issues and integration with existing or new clinical workflows, as well as with legacy information systems. In particular, a fundamental tension exists between model performance and privacy protection: more granular data improves performance but increases privacy risks.

In response to these challenges, decentralized learning approaches have emerged as possible solutions. These approaches aim to enable machine learning on distributed healthcare data while maintaining privacy and regulatory compliance. In federated learning (FL), models are trained locally, and only tuned parameters (e.g., weights and biases in a neural network) of participating parties are shared with a central server for aggregation^12^. In swarm learning (SL), model parameters aggregation occurs peer-to-peer, in a fully decentralized way^13^. This obviates the need to use a central and authoritative controller. Applications cover a variety of data formats and conditions, including some particularly privacy-sensitive, using FL^14–20^ and SL^21,22^. Complementarily, ensemble methods, such as bagging techniques^23^, offer simpler aggregation schemes, based on plurality voting to produce the global result. By design, they are more flexible and integrate different learning methods more easily.

However, a fine balance lies between the ambition to maximize model performance and the need to minimize privacy risks and compromises. Still, comparing decentralized and traditional methods requires a nuanced view. Pivotal problems include regulatory compliance, technical feasibility, and privacy guarantees. Depending on the use case, issues such as number of controllers and points of failures can be seen as either positives or negatives. Considering the General Data Protection Regulation (GDPR)^24^ and the AI Act^24^, attention has been directed towards the goal of decentralized learning models achieving performance comparable to their traditional counterparts^25,26^.

Despite the growing interest in decentralized learning for healthcare applications, there is a lack of robust synthesis comparing their performance with traditional, non-decentralized approaches. Such a systematic comparison would provide crucial insights into their relative effectiveness, practical advantages, and limitations across different clinical contexts and tasks. This knowledge gap hampers informed decision-making about implementing these technologies in healthcare settings and highlights the need for a comprehensive literature assessment. Existing systematic reviews have important limitations regarding their size and scope^27–29^, the specificity of health care applications^30^, and the adequacy and comprehensiveness of query prompts and search strategies^31^. This review builds on a registered and published protocol^32^ to provide a comprehensive and replicable analysis.

This systematic literature review seeks to compare the performance of health data models developed using decentralized learning approaches (e.g., federated learning, swarm learning, ensemble) with those developed using traditional centralized or local methods, as the primary objective. The performance comparison is grouped using the metrics reported in the original articles (e.g., accuracy, precision, AUROC), covering a wide range of medical conditions (e.g., COVID-19, breast cancer, type 2 Diabetes), through different clinical tasks (e.g., diagnosis, segmentation, prognosis). Secondary objectives include describing the types of data and datasets used, the nature of the decentralized model architectures, and the reporting of resource demands or privacy impacts.

We expect to help inform policy-making and operational decisions on the applicability of these methods, integrating their reported strengths and shortcomings with the intended use cases. Moreover, this work suggests further research questions and study designs to better understand the privacy protection benefits, as well as challenges and opportunities concerning their validity and implementation.

## Results

### Study selection

**Fig. 1:**
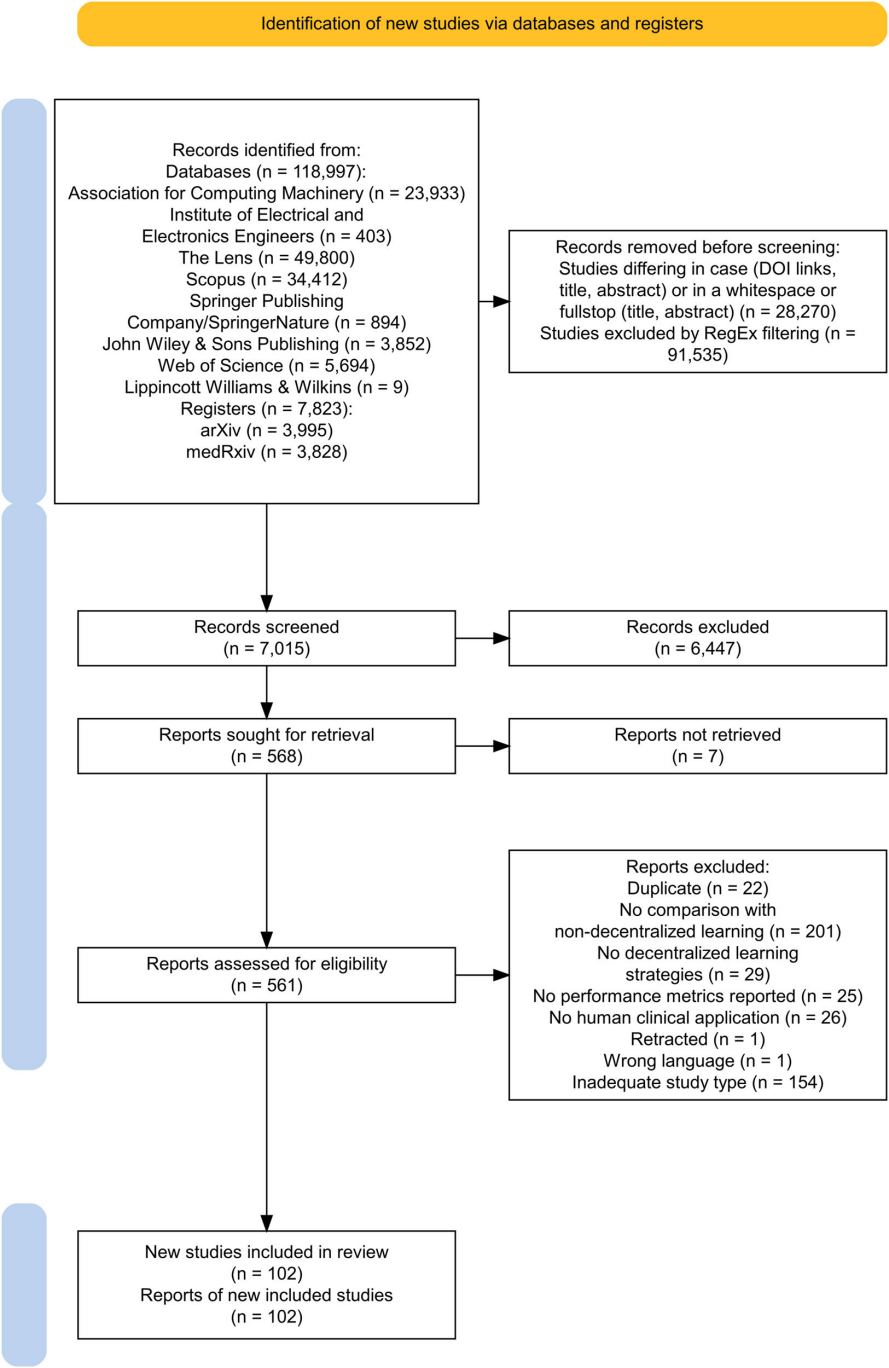
Flow diagram of the study identification and selection process, following Preferred Reporting Items for Systematic Reviews and Meta-Analyses (PRISMA) guidelines – Phase 1. The diagram illustrates the study identification and selection process. Boxes detail the number of records at each stage, with arrows indicating the flow between identification, screening, eligibility assessment, and inclusion stages. Side boxes detail reasons for exclusion at each step.

**Fig. 2:**
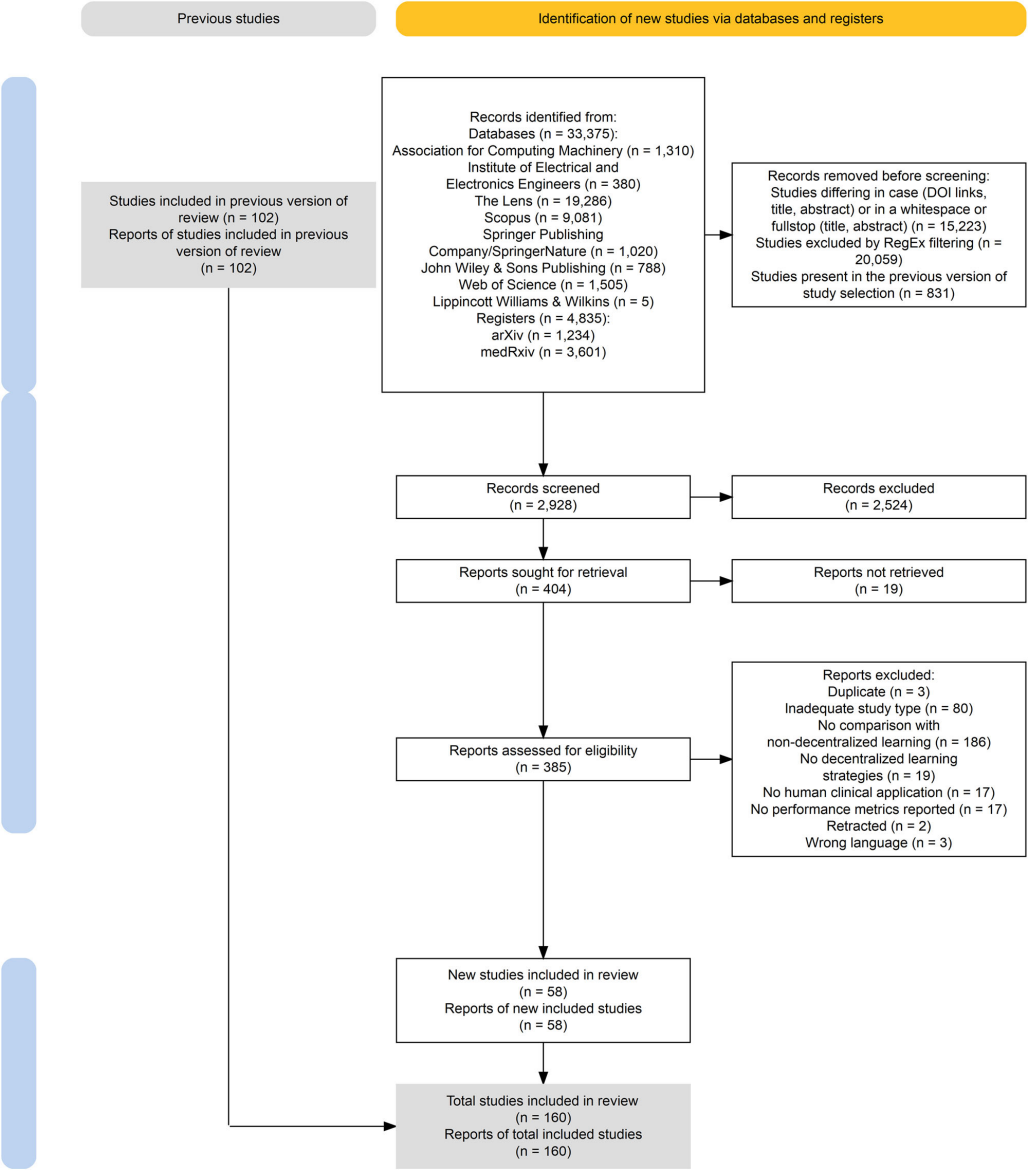
Flow diagram of the study identification and selection process, following Preferred Reporting Items for Systematic Reviews and Meta-Analyses (PRISMA) guidelines – Phase 2. The diagram illustrates the study identification and selection process. Boxes detail the number of records at each stage, with arrows indicating the flow between identification, screening, eligibility assessment, and inclusion stages. Side boxes detail reasons for exclusion at each step.

Our systematic review identified a total of 165,010 studies. Figures 1 and 2 describe the phases 1 and 2 of the identification, screening, and selection processes. Before screening, three processes were undertaken to exclude irrelevant or redundant entries. First, exact and apparent duplicates (i.e., only differing in case for the DOI link, title or abstract, or only differing in a whitespace or a full-stop) were removed (43,493 articles). Subsequently, applying the regular expressions filter described in “Search Strategy”, another 111,594 studies were removed. For Phase 2, duplicates of studies already assessed in Phase 1 were excluded from repeated analysis. In the end, 9943 articles were screened, resulting in the exclusion of 8971 articles. The remaining studies were sought for retrieval, with 26 not being able to be retrieved. During the inclusion stage, applying the eligibility criteria to the full text version of the papers, 946 studies were assessed. In the end, 160 primary articles were analysed, comprising 710 decentralized learning models and 8149 comparisons.

### Study characteristics

The included studies and their main characteristics are presented in Table 1. The most popular broad clinical domains covered were oncological diseases, COVID-19 and neurological conditions, as presented in Table 2. “Diagnosis” was the most common clinical application, as seen in Table 3. A clear trend arises in an increasing number of yearly publications, with 2024 data only including publication until March 28th, as seen in Table 4. The most popular article sources are presented in Table 5.

**Table 1:** Characteristics of included studies

Authorship	Year	Title	Scientific Journal	Link	Number of Models	Number of Comparisons	Clinical Application	Clinical Domain	Data Type
Matteo Pennisi, Federica Proietto Salanitri, Giovanni Bellitto et al.	2024	FedER: Federated Learning through Experience Replay and privacy-preserving data synthesis	Computer Vision and Image Understanding	10.1016/j.cviu.2023.103882	2	5	Diagnosis	Oncology, Other Respiratory	Dermoscopic images, X-Ray
João Coutinho-Almeida, Ricardo João Cruz-Correia, Pedro Pereira Rodrigues	2024	Evaluating distributed-learning on real-world obstetrics data: comparing distributed, centralized and local models	Scientific Reports	10.1038/s41598-024-61371-1	1	4	Prediction	Others	Electronic Health Records / Text
Daniel Truhn, Soroosh Tayebi Arasteh, Oliver Lester Saldanha et al.	2024	Encrypted federated learning for secure decentralized collaboration in cancer image analysis	Medical Image Analysis	10.1016/j.media.2023.103059	2	5	Segmentation	Oncology, Various	MRI + Pathology / Whole slide images
Erfan Darzi, Nanna M. Sijtsema, P.M.A van Ooijen	2024	A Comparative Study of Federated Learning Methods for COVID-19 Detection	Scientific Reports	https://www.nature.com/articles/s41598-024-54323-2	5	40	Diagnosis	COVID-19	CT Scan
Lei Bai, Dongang Wang, Hengrui Wang et al.	2024	Improving multiple sclerosis lesion segmentation across clinical sites: A federated learning approach with noise-resilient training	Artificial Intelligence in Medicine	https://pubmed.ncbi.nlm.nih.gov/38701636/	7	96	Segmentation + Classification	Neurology	MRI
Angela Mitrovska, Pooyan Safari, Kerstin Ritter et al.	2024	Secure federated learning for Alzheimer’s disease detection	Frontiers in Aging Neuroscience	10.3389/fnagi.2024.1324032	2	16	Diagnosis	Neurology	MRI
Junmo Kim, Min Hyuk Lim, Kwangsoo Kim et al.	2024	Continual learning framework for a multicenter study with an application to electrocardiogram	BMC Medical Informatics and Decision Making	10.1186/s12911-024-02464-9	16	36	Diagnosis	Cardiology	ECG / EKG
Jiacheng Wang, Yueming Jin, Danail Stoyanov et al.	2024	FedDP: Dual Personalization in Federated Medical Image Segmentation	IEEE Transactions on Medical Imaging	10.1109/TMI.2023.3299206	18	180	Segmentation	Gastric & Digestive, Neurology	Endoscopic image, Retina fundus image
Yuning Yang, Xiaohong Liu, Tianrun Gao et al.	2024	Dense Contrastive-based Federated Learning for Dense Prediction Tasks on Medical Images	IEEE Journal of Biomedical and Health Informatics	10.1109/jbhi.2024.3357947	5	30	Diagnosis	Oncology	CT Scan
Hussain Alsalman, Mabrook S. Al-Rakhami, Taha Alfakih et al.	2024	Federated Learning Approach for Breast Cancer Detection Based on DCNN	IEEE Access	10.1109/ACCESS.2024.3374650	1	6	Diagnosis	Oncology	Mammography
Dipanjali Kundu, Mahbubur Rahman, Anichur Rahman et al.	2024	Federated Deep Learning for Monkeypox Disease Detection on GAN-Augmented Dataset	IEEE Access	10.1109/ACCESS.2024.3370838	4	8	Diagnosis	Others	Dermoscopic images
Thi Phuoc Van Nguyen, Wencheng Yang, Zhaohui Tang et al.	2024	Lightweight federated learning for STIs/HIV prediction	Scientific Reports	10.1038/s41598-024-56115-0	2	28	Prediction	Others	Electronic Health Records / Text
Jiaqi Ge, Gaochao Xu, Jianchao Lu et al.	2024	FedAGA: A federated learning framework for enhanced inter-client relationship learning	Knowledge-Based Systems	10.1016/j.knosys.2024.111399	9	18	Diagnosis	Oncology	Pathology / Whole slide images
Haroon Wahab, Irfan Mehmood, Hassan Ugail et al.	2024	Federated Deep Learning for Wireless Capsule Endoscopy Analysis: Enabling Collaboration Across Multiple Data Centers for Robust Learning of Diverse Pathologies	Future Generation Computer Systems	10.1016/j.future.2023.10.007	4	16	Diagnosis	Gastric & Digestive	Endoscopic video (Wireless capsule endoscopy)
Isaac Adjei-Mensah, Xiaoling Zhang, Isaac Osei Agyemang et al.	2024	Cov-Fed: Federated learning-based framework for COVID-19 diagnosis using chest X-ray scans	Engineering Applications of Artificial Intelligence	10.1016/j.engappai.2023.107448	1	60	Diagnosis	COVID-19	X-Ray
Gaeun Sung, Eunjeong Park	2024	Aggregate and transfer knowledge of functional connectivity of brain for detecting autism spectrum disorder for multi-site research	Biomedical Signal Processing and Control	10.1016/j.bspc.2024.106068	2	200	Diagnosis	Psychology & Psychiatry	MRI
I. De Falco, A. Della Cioppa, T. Koutny et al.	2024	Model-Free-Communication Federated Learning: Framework and application to Precision Medicine	Biomedical Signal Processing and Control	10.1016/j.bspc.2023.105416	1	42	Prediction	Diabetes	Other
Jong Chan Yeom, Jae Hoon Kim, Young Jae Kim et al.	2024	A Comparative Study of Performance Between Federated Learning and Centralized Learning Using Pathological Image of Endometrial Cancer.	Journal of Imaging Informatics in Medicine	10.1007/s10278-024-01020-1	4	14	Segmentation + Classification	Oncology	Pathology / Whole slide images
Isaac Shiri, Yazdan Salimi, Nasim Sirjani et al.	2024	Differential privacy preserved federated learning for prognostic modeling in COVID-19 patients using large multi-institutional chest CT dataset	Medical Physics	10.1002/mp.16964	1	10	Prognosis (including Mortality)	COVID-19	CT Scan
Vi Thi-Tuong Vo, Tae-ho Shin, Hyung-Jeong Yang et al.	2024	A comparison between centralized and asynchronous federated learning approaches for survival outcome prediction using clinical and PET data from non-small cell lung cancer patients	Computer Methods and Programs in Biomedicine	10.1016/j.cmpb.2024.108104	4	20	Prognosis	Oncology	Electronic Health Records / Text, Other
Soroosh Tayebi Arasteh, Peter Isfort, Marwin Saehn et al.	2023	Collaborative training of medical artificial intelligence models with non-uniform labels	Scientific Reports	10.1038/s41598-023-33303-y	5	5	Diagnosis	Others	X-Ray
Quan Nguyen, Hieu H. Pham, Kok-Seng Wong et al.	2023	FedDCT: Federated Learning of Large Convolutional Neural Networks on Resource-Constrained Devices Using Divide and Collaborative Training	IEEE Transactions on Network and Service Management	10.1109/TNSM.2023.3314066	1	1	Diagnosis	Oncology	Dermoscopic images
Xuanang Xu, Hannah H. Deng, Jaime Gateno et al.	2023	Federated Multi-Organ Segmentation With Inconsistent Labels	IEEE Transactions on Medical Imaging	10.1109/TMI.2023.3270140	2	120	Segmentation	Various	CT Scan
Hasan Kassem, Deepak Alapatt, Pietro Mascagni et al.	2023	Federated Cycling (FedCy): Semi-Supervised Federated Learning of Surgical Phases	IEEE Transactions on Medical Imaging	10.1109/TMI.2022.3222126	16	88	Other	Gastric & Digestive	Laparoscopic cholecystectomy videos
Shivam Kalra, Junfeng Wen, Jesse C. Cresswell et al.	2023	Decentralized federated learning through proxy model sharing	Nature Communications	10.1038/s41467-023-38569-4	14	14	Diagnosis	Gastric & Digestive, Oncology	Endoscopic image, Pathology / Whole slide images
Amelia Jiménez-Sánchez, Mickael Tardy, Miguel A. González Ballester et al.	2023	Memory-aware curriculum federated learning for breast cancer classification	Computer Methods and Programs in Biomedicine	10.1016/j.cmpb.2022.107318	1	2	Diagnosis	Oncology	Mammography
Nawrin Tabassum, Mustofa Ahmed, Nushrat Jahan Shorna et al.	2023	Depression Detection Through Smartphone Sensing: A Federated Learning Approach	International Journal of Interactive Mobile Technologies	10.3991/ijim.v17i01.35131	1	4	Diagnosis	Psychology & Psychiatry	Other
Xikang Jiang, Jinhui Zhang, Lin Zhang	2023	FedRadar: Federated Multi-Task Transfer Learning for Radar-Based Internet of Medical Things	IEEE Transactions on Network and Service Management	10.1109/TNSM.2023.3281133	2	128	Diagnosis	Cardiology	ECG / EKG
Ivanoe De Falco, Antonio Della Cioppa, Tomas Koutny et al.	2023	A Federated Learning-Inspired Evolutionary Algorithm: Application to Glucose Prediction	Sensors (Basel)	10.3390/s23062957	1	15	Prediction	Diabetes	Other
Hassaan Malik, Tayyaba Anees, Ahmad Naeem et al.	2023	Blockchain-Federated and Deep-Learning-Based Ensembling of Capsule Network with Incremental Extreme Learning Machines for Classification of COVID-19 Using CT Scans	Bioengineering	10.3390/bioengineering10020203	2	42	Diagnosis, Segmentation	COVID-19	CT Scan
Laëtitia Launet, Yuandou Wang, Adrián Colomer et al.	2023	Federating Medical Deep Learning Models from Private Jupyter Notebooks to Distributed Institutions	Applied Sciences	10.3390/app13020919	1	18	Diagnosis	Oncology	Pathology / Whole slide images
Miloš Savić, Vladimir Kurbalija, Mihailo Ilić et al.	2023	The application of machine learning techniques in prediction of quality of life features for cancer patients	Computer Science and Information Systems	10.2298/CSIS220227061S	10	132	Prediction	Oncology	Electronic Health Records / Text
Dong Yun Lee, Byungjin Choi, Chungsoo Kim et al.	2023	Privacy-Preserving Federated Model Predicting Bipolar Transition in Patients With Depression: Prediction Model Development Study	Journal of Medical Internet Research	https://www.jmir.org/2023//e46165/	1	4	Prediction	Psychology & Psychiatry	Electronic Health Records / Text
Jun Jie Sim, Weizhuang Zhou, Fook Mun Chan et al.	2023	CoVnita, an end-to-end privacy-preserving framework for SARS-CoV-2 classification	Scientific Reports	10.1038/s41598-023-34535-8	1	60	Diagnosis	COVID-19	Genome
Martin Baumgartner, Sai Pavan Kumar Veeranki, Dieter Hayn et al.	2023	Introduction and Comparison of Novel Decentral Learning Schemes with Multiple Data Pools for Privacy-Preserving ECG Classification	Journal of Healthcare Informatics Research	10.1007/s41666-023-00142-5	2	48	Diagnosis	Cardiology	ECG / EKG
Suraj Rajendran, Zhenxing Xu, Weishen Pan et al.	2023	Data heterogeneity in federated learning with Electronic Health Records: Case studies of risk prediction for acute kidney injury and sepsis diseases in critical care	PLoS One Digital Health	10.1371/journal.pdig.0000117	6		Prediction	Nephrology, Systemic	Electronic Health Records / Text & X-Ray
Xing Wu, Jie Pei, Cheng Chen et al.	2023	Federated Active Learning for Multicenter Collaborative Disease Diagnosis	IEEE Transactions on Medical Imaging	10.1109/tmi.2022.3227563	2	40	Diagnosis	COVID-19, Gastric & Digestive	CT Scan, Endoscopic image
Weiping Ding, Mohamed Abdel-Basset, Hossam Hawash et al.	2023	Generalizable Segmentation of COVID-19 Infection From Multi-Site Tomography Scans: A Federated Learning Framework	IEEE Transactions on Emerging Topics in Computational Intelligence	10.1109/tetci.2023.3245103	1	10	Segmentation	COVID-19	CT Scan
Sarah Haggenmüller, Max Schmitt, Eva Krieghoff-Henning et al.	2023	Federated Learning for Decentralized Artificial Intelligence in Melanoma Diagnostics	JAMA Dermatology	10.1001/jamadermatol.2023.5550	4	80	Diagnosis	Oncology	Pathology / Whole slide images
Weiping Ding, Mohamed Abdel-Basset, Hossam Hawash et al.	2023	MIC-Net: A deep network for cross-site segmentation of COVID-19 infection in the fog-assisted IoMT	Information Sciences	10.1016/j.ins.2022.12.017	1	18	Segmentation	COVID-19	CT Scan
Abhejit Rajagopal, Ekaterina Redekop, Anil Kemisetti et al.	2023	Federated Learning with Research Prototypes: Application to Multi-Center MRI-based Detection of Prostate Cancer with Diverse Histopathology	Academic Radiology	10.1016/j.acra.2023.02.012	4	16	Segmentation, Diagnosis	Oncology	MRI + Pathology / Whole slide images
Mehmet Nergiz	2023	Federated learning-based colorectal cancer classification by convolutional neural networks and general visual representation learning	International Journal of Imaging Systems and Technology (IMA)	10.1002/ima.22875	14	84	Diagnosis	Oncology	Pathology / Whole slide images
Dongnan Liu, Mariano Cabezas, Donggang Wang et al.	2023	Multiple sclerosis lesion segmentation: revisiting weighting mechanisms for federated learning	Frontiers in Neuroscience	10.3389/fnins.2023.1167612	5	80	Segmentation	Neurology	MRI
Soroosh Tayebi Arasteh, Christiane Kuhl, Marwin-Jonathan Saehn et al.	2023	Enhancing domain generalization in the AI-based analysis of chest radiographs with federated learning	Scientific Reports	https://www.nature.com/articles/s41598-023-49956-8	2	120	Diagnosis	Other Respiratory	X-Ray
Matthis Manthe, Stefan Duffner, Carole Lartizien	2023	Federated brain tumor segmentation: An extensive benchmark	Medical Image Analysis	https://www.sciencedirect.com/science/article/pii/S1361841524001956	17	105	Segmentation	Oncology	MRI
Zahra Tabatabaei, Yuandou Wang, Adrián Colomer et al.	2023	WWFedCBMIR: World-Wide Federated Content-Based Medical Image Retrieval	Bioengineering	https://www.mdpi.com/2306-5354/10/10/1144	2	24	Diagnosis	Oncology	Pathology / Whole slide images
Geun Hyeong Lee, Jonggul Park, Jihyeong Kim et al.	2023	Feasibility Study of Federated Learning on the Distributed Research Network of OMOP Common Data Model	Healthcare Informatics Research	10.4258/hir.2023.29.2.168	1	6	Prediction	Others	Electronic Health Records / Text
Raghdah Saemaldahr, Mohammad Ilyas	2023	Patient-Specific Preictal Pattern-Aware Epileptic Seizure Prediction with Federated Learning	Sensors	10.3390/s23146578	4	24	Prediction	Neurology	EEG, Other
Faizan Ullah, Muhammad Nadeem, Mohammad Abrar et al.	2023	Enhancing Brain Tumor Segmentation Accuracy through Scalable Federated Learning with Advanced Data Privacy and Security Measures	Mathematics	10.3390/math11194189	1	15	Segmentation + Classification	Neurology	MRI
Tapotosh Ghosh, Md Istakiak Adnan Palash, Mohammad Abu Yousuf et al.	2023	A Robust Distributed Deep Learning Approach to Detect Alzheimer’s Disease from MRI Images	Mathematics	10.3390/math11122633	1	48	Diagnosis	Neurology	MRI
Wu-Chun Chung, Yan-Hui Lin, Sih-Han Fang	2023	FedISM: Enhancing Data Imbalance via Shared Model in Federated Learning	Mathematics	10.3390/math11102385	4	44	Diagnosis	COVID-19	X-Ray
Giovanni Paragliola, Patrizia Ribino, Zaib Ullah	2023	A Federated Learning Approach to Support the Decision-Making Process for ICU Patients in a European Telemedicine Network	Journal of Sensor and Actuator Networks	10.3390/jsan12060078	1	26	Prediction	COVID-19	EEG
Chengxiao Yan, Xiaoyang Zeng, Rui Xi et al.	2023	PLA - A Privacy-Embedded Lightweight and Efficient Automated Breast Cancer Accurate Diagnosis Framework for the Internet of Medical Things	Electronics	10.3390/electronics12244923	1	16	Diagnosis	Oncology	Pathology / Whole slide images
Maryum Butt, Noshina Tariq, Muhammad Ashraf et al.	2023	A Fog-Based Privacy-Preserving Federated Learning System for Smart Healthcare Applications	Electronics	10.3390/electronics12194074	1	12	Diagnosis	Other Respiratory	X-Ray
Liuyan Yang, Juanjuan He, Yue Fu et al.	2023	Federated Learning for Medical Imaging Segmentation via Dynamic Aggregation on Non-IID Data Silos	Electronics	10.3390/electronics12071687	11	572	Segmentation + Classification	COVID-19	CT Scan
Chetna Gupta, Vikas Khullar, Nitin Goyal et al.	2023	Cross-Silo, Privacy-Preserving, and Lightweight Federated Multimodal System for the Identification of Major Depressive Disorder Using Audio and Electroencephalogram.	Diagnostics	10.3390/diagnostics14010043	1	8	Diagnosis	Psychology & Psychiatry	Other
Ivar Walskaar, Minh Christian Tran, Ferhat Ozgur Catak	2023	A Practical Implementation of Medical Privacy-Preserving Federated Learning Using Multi-Key Homomorphic Encryption and Flower Framework	Cryptography	https://www.mdpi.com/2410-387X/7/4/48	2	64	Diagnosis	COVID-19	X-Ray
Mohamed Chetoui, Moulay A. Akhloufi	2023	Federated Learning for Diabetic Retinopathy Detection Using Vision Transformers	BioMedInformatics	10.3390/biomedinformatics3040058	2	32	Diagnosis	Neurology	Retina fundus image
Telmo Baptista, Carlos Soares, Tiago Oliveira et al.	2023	Federated Learning for Computer-Aided Diagnosis of Glaucoma Using Retinal Fundus Images	Applied Sciences	10.3390/app132111620	5	150	Diagnosis	Neurology	Retina fundus image
Ali Akbar Siddique, S. M. Umar Talha, M. Aamir et al.	2023	COVID-19 Classification from X-Ray Images: An Approach to Implement Federated Learning on Decentralized Dataset	Computers, Materials & Continua	10.32604/cmc.2023.037413	6	32	Diagnosis	COVID-19	X-Ray
Kavitha Srinivasasn, Sainath Prasanna, Rohit Midha et al.	2023	Federated Learning Framework for IID and Non-IID datasets of Medical Images	EMITTER International Journal of Engineering Technology	10.24003/emitter.v11i1.742	3	4	Diagnosis, Segmentation	Neurology, Other Respiratory	CT Scan, X-Ray
Dhanunjay Potti, Mandavalli N V Saisandeep, V Madhu Viswanatham et al.	2023	Heart Stroke Prediction Using Federated Learning	International Journal of Membrane Science and Technology	10.15379/ijmst.v10i3.1800	2	8	Prediction	Cardiology	Electronic Health Records / Text
Nanqing Dong, Michael Kampffmeyer, Irina Voiculescu et al.	2023	Federated Partially Supervised Learning With Limited Decentralized Medical Images	IEEE Transactions on Medical Imaging	10.1109/tmi.2022.3231017	8	277	Diagnosis	Other Respiratory	X-Ray
Qingguo Zhou, Rui Zhao, Yilin Hu et al.	2023	Hierarchical Hybrid Networks for Automatic Pulmonary Blood Vessel Segmentation in Computed Tomography Images	IEEE/ACM Transactions on Computational Biology and Bioinformatics	10.1109/TCBB.2023.3281828	1	5	Segmentation	Other Respiratory	CT Scan
Ziyuan Yang, Yingyu Chen, Huijie Huangfu et al.	2023	Dynamic Corrected Split Federated Learning With Homomorphic Encryption for U-Shaped Medical Image Networks	IEEE Journal of Biomedical and Health Informatics	10.1109/jbhi.2023.3317632	9	36	Segmentation	Cardiology	MRI
Adriana Anido-Alonso, Diego Alvarez-Estevez	2023	Decentralized Data-Privacy Preserving Deep-Learning Approaches for Enhancing Inter-Database Generalization in Automatic Sleep Staging	IEEE Journal of Biomedical and Health Informatics	10.1109/jbhi.2023.3310869	2	72	Diagnosis	Others	Other
Bless Lord Y Agbley, Jian Ping Li, Amin Ul Haq et al.	2023	Federated Fusion of Magnified Histopathological Images for Breast Tumor Classification in the Internet of Medical Things	IEEE Journal of Biomedical and Health Informatics	10.1109/jbhi.2023.3256974	2	24	Diagnosis	Oncology	Pathology / Whole slide images
Andrew A S Soltan, Anshul Thakur, Jenny Yang et al.	2023	A scalable federated learning solution for secondary care using low-cost microcomputing: privacy-preserving development and evaluation of a COVID-19 screening test in UK hospitals	The Lancet Digit Health	10.1016/S2589-7500(23)00226-1	2	56	Diagnosis	COVID-19	Electronic Health Records / Text
Bastian Pfeifer, Hryhorii Chereda, Roman Martin et al.	2023	Ensemble-GNN: federated ensemble learning with graph neural networks for disease module discovery and classification	Bioinformatics	10.1093/2Fbioinformatics/2Fbtad703	2	4	Prediction	Oncology	Genome
Soroosh Tayebi Arasteh, Christiane Kuhl, Marwin-Jonathan Saehn et al.	2023	Enhancing domain generalization in the AI-based analysis of chest radiographs with federated learning	Scientific Reports	10.1038/s41598-023-49956-8	10	110	Diagnosis	Various	X-Ray
Daniele Raimondi, Haleh Chizari, Nora Verplaetse et al.	2023	Genome interpretation in a federated learning context allows the multi-center exome-based risk prediction of Crohn’s disease patients	Scientific Reports	10.1038/s41598-023-46887-2	5	180	Diagnosis	Gastric & Digestive	Genome
Ruijie Tang, Hengrui Liang, Yuchen Guo et al.	2023	Pan-mediastinal neoplasm diagnosis via nationwide federated learning: a multicentre cohort study	The Lancet Digit Health	10.1016/S2589-7500(23)00106-1	1	30	Segmentation + Classification	Oncology	CT Scan
Weishen Pan, Zhenxing Xu, Suraj Rajendran et al.	2023	An adaptive federated learning framework for clinical risk prediction with electronic health records from multiple hospitals	Patterns	10.1016/j.patter.2023.100898	4	72	Prediction	Systemic, Nephrology	Electronic Health Records / Text
Bruno Casella, Walter Riviera, Marco Aldinucci et al.	2023	MERGE: A model for multi-input biomedical federated learning	Patterns	10.1016/j.patter.2023.100856	2	54	Diagnosis	COVID-19, Neurology	X-Ray, MRI
Benteng Ma, Yu Feng, Geng Chen et al.	2023	Federated adaptive reweighting for medical image classification	Pattern Recognition	10.1016/j.patcog.2023.109880	6	72	Diagnosis	Others	Dermoscopic images, X-Ray
Judith Sáinz-Pardo Díaz, Álvaro López García	2023	Study of the performance and scalability of federated learning for medical imaging with intermittent clients	Neurocomputing	10.1016/j.neucom.2022.11.011	1	3	Diagnosis	Other Respiratory	X-Ray
Amer Kareem, Haiming Liu, Vladan Velisavljevic	2023	A federated learning framework for pneumonia image detection using distributed data	Healthcare Analytics	10.1016/j.health.2023.100204	5	15	Diagnosis	Other Respiratory	X-Ray
Alberto Archetti, Francesca Ieva, Matteo Matteucci	2023	Scaling survival analysis in healthcare with federated survival forests: A comparative study on heart failure and breast cancer genomics	Future Generation Computer Systems	10.1016/j.future.2023.07.036	22	22	Prognosis (including Mortality)	Cardiology, Oncology	Genome
William Hoyos, Jose Aguilar, Mauricio Toro	2023	Federated learning approaches for fuzzy cognitive maps to support clinical decision-making in dengue	Engineering Applications of Artificial Intelligence	10.1016/j.engappai.2023.106371	6	30	Prediction, Therapy	Others	Electronic Health Records / Text
Isaac Shiri, Behrooz Razeghi, Alireza Vafaei Sadr et al.	2023	Multi-institutional PET/CT image segmentation using federated deep transformer learning	Computer Methods and Programs in Biomedicine	10.1016/j.cmpb.2023.107706	7	91	Segmentation	Oncology	Other
Pascal Riedel, Reinhold von Schwerin, Daniel Schaudt et al.	2023	ResNetFed: Federated Deep Learning Architecture for Privacy-Preserving Pneumonia Detection from COVID-19 Chest Radiographs	Journal of Healthcare Informatics Research	10.1007/s41666-023-00132-7	1	3	Diagnosis	COVID-19	X-Ray
Wei-Kai Lee, Jia-Sheng Hong, Yi-Hui Lin et al.	2023	Federated Learning: A Cross-Institutional Feasibility Study of Deep Learning Based Intracranial Tumor Delineation Framework for Stereotactic Radiosurgery	Journal of Magnetic Resonance Imaging	10.1002/jmri.28950	1	30	Segmentation	Oncology	MRI
Yuan Yang, Lin Zhang, Lei Ren et al.	2023	Distributed autoencoder classifier network for small-scale and scattered COVID-19 dataset classification	International Journal of Imaging Systems and Technology	10.1002/ima.22972	2	30	Diagnosis	COVID-19	CT Scan
Bo Guan, Lei Yu, Yang Li et al.	2023	Assessment of patients with Parkinson’s disease based on federated learning	International Journal of Machine Learning and Cybernetics	10.1007/s13042-023-01986-4	1	1	Other	Neurology	Other
Wided Moulahi, Imen Jdey, Tarek Moulahi et al.	2023	A blockchain-based federated learning mechanism for privacy preservation of healthcare IoT data	Computers in Biology and Medicine	10.1016/j.compbiomed.2023.107630	1	3	Prediction	Diabetes	Electronic Health Records / Text
Miao Zhang, Liangqiong Qu, Praveer Singh et al.	2022	SplitAVG: A Heterogeneity-Aware Federated Deep Learning Method for Medical Imaging	IEEE Journal of Biomedical and Health Informatics	10.1109/2FJBHI.2022.3185956	38	78	Segmentation + Classification, Prediction, Diagnosis, Segmentation	Diabetes, Others, Oncology, Neurology	Retina fundus image, X-Ray, MRI
Tien-Dung Cao, Tram Truong-Huu, Hien Tran et al.	2022	A federated deep learning framework for privacy preservation and communication efficiency	Journal of Systems Architecture	10.1016/j.sysarc.2022.102413	1	8	Diagnosis	Neurology	MRI
Ahmed Sleem, Ibrahim Elhenawy	2022	Collaborative Segmentation of COVID-19 From non-IID Topographies in the Internet of Medical Things (IoMT)	Journal of Intelligent Systems & Internet of Things	10.54216/jisiot.070201	3	24	Segmentation	COVID-19	CT Scan
Yawen Wu, Dewen Zeng, Zhepeng Wang et al.	2022	Distributed contrastive learning for medical image segmentation	Medical Image Analysis	10.1016/j.media.2022.102564	4	4	Segmentation	Cardiology	MRI
Praveen Joshi, Chandra Thapa, Seyit Camtepe et al.	2022	Performance and Information Leakage in Splitfed Learning and Multi-Head Split Learning in Healthcare Data and Beyond	Methods Protoc	10.3390/mps5040060	4	4	Diagnosis	Various	ECG / EKG, Dermoscopic images
Mohamed Abdel-Basset, Hossam Hawash, Mohamed Abouhawwash	2022	Collaborative Screening of COVID-19-like Disease from Multi-Institutional Radiographs: A Federated Learning Approach	Mathematics	10.3390/math10244766	2	36	Segmentation	COVID-19	CT Scan
Lucian Mihai Florescu, Costin Teodor Streba, Mircea-Sebastian Şerbănescu et al.	2022	Federated Learning Approach with Pre-Trained Deep Learning Models for COVID-19 Detection from Unsegmented CT images	Life (Basel)	10.3390/life12070958	1	5	Diagnosis	COVID-19	CT Scan
Lingxiao Li, Niantao Xie, Sha Yuan	2022	A Federated Learning Framework for Breast Cancer Histopathological Image Classification	Electronics	10.3390/electronics11223767	4	20	Diagnosis	Oncology	Pathology / Whole slide images
Tingyang Yang, Jingshuang Xu, Mengxiao Zhu et al.	2022	FedZaCt: Federated Learning with Z Average and Cross-Teaching on Image Segmentation	Electronics	10.3390/electronics11203262	23	76	Segmentation	Others, Various	Unstated, Dermoscopic images
Bless Lord Y. Agbley, Jianping Li, Md Altab Hossin et al.	2022	Federated Learning-Based Detection of Invasive Carcinoma of No Special Type with Histopathological Images	Diagnostics	10.3390/diagnostics12071669	1	48	Diagnosis	Oncology	Pathology / Whole slide images
Songshang Liu, Howard H. Yang, Yiqi Tao et al.	2022	Privacy-Preserved Federated Learning for 3D Tooth Segmentation in Intra-Oral Mesh Scans	Frontiers in Communications and Networks	10.3389/frcmn.2022.907388	1	36	Segmentation	Others	Intra-Oral Mesh Scans
Barkha Kakkar, Prashant Johri, Yogesh Kumar et al.	2022	An IoMT-Based Federated and Deep Transfer Learning Approach to the Detection of Diverse Chest Diseases Using Chest X-Rays	Human-centric Computing and Information Sciences	10.22967/HCIS.2022.12.024	5	15	Diagnosis	Other Respiratory	Electronic Health Records / Text & X-Ray
Geun Hyeong Lee, Soo-Yong Shin	2022	Federated Learning on Clinical Benchmark Data: Performance Assessment	JMIR Medical Informatics	10.2196/20891	2	34	Mortality, Diagnosis	Others, Cardiology	Electronic Health Records / Text, ECG / EKG
T. V. Nguyen, M. A. Dakka, S. M. Diakiw et al.	2022	A novel decentralized federated learning approach to train on globally distributed, poor quality, and protected private medical data	Scientific Reports	10.21203/rs.3.rs-1371143/v1	2	2	Diagnosis	Others	Microscopy
Mohammed Adnan, Shivam Kalra, Jesse C. Cresswell et al.	2022	Federated learning and differential privacy for medical image analysis	Scientific Reports	10.21203/rs.3.rs-1005694/v1	2	24	Diagnosis	Oncology	Pathology / Whole slide images
Omneya Atef, Mustafa Abdul Salam, Hisham Abdelsalam	2022	Federated Learning Approach for Measuring the Response of Brain Tumors to Chemotherapy	International Journal of Advanced Computer Science and Applications	10.14569/IJACSA.2022.0131060	1	2	Prognosis	Oncology	MRI
Dhurgham Hassan Mahlool, Mohamed Hamzah Abed	2022	Distributed brain tumor diagnosis using a federated learning environment	Bulletin of Electrical Engineering and Informatics	10.11591/eei.v11i6.4131	1	8	Diagnosis	Oncology	MRI
Ziyu Wang, Lei Cai, Xuewu Zhang et al.	2022	A COVID-19 Auxiliary Diagnosis Based on Federated Learning and Blockchain	Computational and Mathematical Methods in Medicine	10.1155/2022/7078764	2	20	Diagnosis	COVID-19	CT Scan
Xiaolong Xu, Hao Tian, Xuyun Zhang et al.	2022	DisCOV: Distributed COVID-19 Detection on X-Ray Images With Edge-Cloud Collaboration	IEEE Transactions on Services Computing	10.1109/tsc.2022.3142265	1	3	Diagnosis	COVID-19	X-Ray
Thang Ngo, Dinh C. Nguyen, Pubudu N. Pathirana et al.	2022	Federated Deep Learning for the Diagnosis of Cerebellar Ataxia: Privacy Preservation and Auto-Crafted Feature Extractor	IEEE Transactions on Neural Systems and Rehabilitation Engineering	10.1109/tnsre.2022.3161272	1	1	Diagnosis	Neurology	Electronic Health Records / Text
Ling-Li Zeng, Zhipeng Fan, Jianpo Su et al.	2022	Gradient Matching Federated Domain Adaptation for Brain Image Classification	IEEE Transactions on Neural Networks and Learning Systems	10.1109/tnnls.2022.3223144	6	90	Diagnosis	Psychology & Psychiatry	MRI
Zheyao Gao, Fuping Wu, Weiguo Gao et al.	2022	A New Framework of Swarm Learning Consolidating Knowledge From Multi-Center Non-IID Data for Medical Image Segmentation	IEEE Transactions on Medical Imaging	10.1109/tmi.2022.3220750	17	85	Segmentation	Cardiology, Others, Oncology	MRI, CT Scan
Liang Zou, Zexin Huang, Xinhui Yu et al.	2022	Automatic Detection of Congestive Heart Failure Based on Multiscale Residual UNet + +: From Centralized Learning to Federated Learning	IEEE Transactions on Instrumentation and Measurement	10.1109/TIM.2022.3227955	1	27	Diagnosis	Cardiology	ECG / EKG
Jeffry Wicaksana, Zengqiang Yan, Xin Yang et al.	2022	Customized Federated Learning for Multi-Source Decentralized Medical Image Classification	IEEE Journal of Biomedical and Health Informatics	10.1109/jbhi.2022.3198440	4	16	Diagnosis	Oncology	MRI, Dermoscopic images
Amin Aminifar, Matin Shokri, Fazle Rabbi et al.	2022	Extremely Randomized Trees With Privacy Preservation for Distributed Structured Health Data	IEEE Access	10.1109/access.2022.3141709	8	128	Diagnosis	Cardiology, Oncology, Psychology & Psychiatry	Other, Pathology / Whole slide images
Oliver Lester Saldanha, Philip Quirke, Nicholas P. West et al.	2022	Swarm learning for decentralized artificial intelligence in cancer histopathology	Nature Medicine	10.1038/s41591-022-01768-5	3	27	Prediction	Oncology	Pathology / Whole slide images
Brendon Lutnick, David Manthey, Jan U. Becker et al.	2022	A tool for federated training of segmentation models on whole slide images	Journal of Pathology Informatics	10.1016/j.jpi.2022.100101	1	6	Segmentation	Nephrology	Pathology / Whole slide images
Isaac Shiri, Alireza Vafaei Sadr, Mehdi Amini et al.	2022	Decentralized Distributed Multi-institutional PET Image Segmentation Using a Federated Deep Learning Framework	Clinical Nuclear Medicine	10.1097/RLU.0000000000004194	1	54	Segmentation	Oncology	PET image
Le Peng, Gaoxiang Luo, Andrew Walker et al.	2022	Evaluation of federated learning variations for COVID-19 diagnosis using chest radiographs from 42 US and European hospitals	Journal of the American Medical Informatics Association	10.1093/jamia/ocac188	4	41	Diagnosis	COVID-19	X-Ray
Akis Linardos, Kaisar Kushibar, Sean Walsh et al.	2022	Federated learning for multi-center imaging diagnostics: a simulation study in cardiovascular disease	Scientific Reports	10.1038/s41598-022-07186-4	11	83	Diagnosis	Cardiology	MRI
Mahbubur Rahman, Dipanjali Kundu, Sayma Alam Suha et al.	2022	Hospital patients’ length of stay prediction: A federated learning approach	Journal of King Saud University - Computer and Information Sciences	10.1016/j.jksuci.2022.07.006	3	90	Prognosis	Various	Electronic Health Records / Text
Dianbo Liu, Kathe Fox, Griffin Weber et al.	2022	Confederated learning in healthcare: Training machine learning models using disconnected data separated by individual, data type and identity for Large-Scale health system Intelligence	Journal of Biomedical Informatics	10.1016/j.jbi.2022.104151	6	24	Prediction	Diabetes, Psychology & Psychiatry, Cardiology	Electronic Health Records / Text
Giovanni Paragliola, Antonio Coronato	2022	Definition of a novel federated learning approach to reduce communication costs	Expert Systems with Applications	10.1016/j.eswa.2021.116109	3	25	Prediction	Systemic	ECG / EKG
Rajesh Kumar, Jay Kumar, Abdullah Aman Khan et al.	2022	Blockchain and homomorphic encryption based privacy-preserving model aggregation for medical images	Computerized Medical Imaging and Graphics	10.1016/j.compmedimag.2022.102139	6	45	Diagnosis, Segmentation	COVID-19	CT Scan
Suresh Dara, Ambedkar Kanapala, A. Ramesh Babu et al.	2022	Scalable Federated-Learning and Internet-of-Things enabled architecture for Chest Computer Tomography image classification	Computers and Electrical Engineering	10.1016/j.compeleceng.2022.108266	1	6	Diagnosis	COVID-19	CT Scan
Jinli Li, Ming Jiang, Yunbai Qin et al.	2022	Intelligent depression detection with asynchronous federated optimization	Complex & Intelligent Systems	10.1007/s40747-022-00729-2	2	24	Diagnosis	Psychology & Psychiatry	Other
Oliver Lester Saldanha, Hannah Sophie Muti, Heike I Grabsch et al.	2022	Direct prediction of genetic aberrations from pathology images in gastric cancer with swarm learning	Gastric Cancer	10.1007/s10120-022-01347-0	1	8	Diagnosis	Oncology	Pathology / Whole slide images
Ittai Dayan, Holger R. Roth, Aoxiao Zhong et al.	2021	Federated learning for predicting clinical outcomes in patients with COVID-19	Nature Medicine	https://www.nature.com/articles/s41591-021-01506-3	1	2	Prognosis (including Mortality)	COVID-19	Electronic Health Records / Text & X-Ray
Xiang Bai, Hanchen Wang, Liya Ma et al.	2021	Advancing COVID-19 diagnosis with privacy-preserving collaboration in artificial intelligence	Nature Machine Intelligence	https://www.nature.com/articles/s42256-021-00421-z	1	24	Diagnosis	COVID-19	CT Scan
Xiaohang Xu, Hao Peng, Lichao Sun et al.	2021	Privacy-Preserving Federated Depression Detection From Multisource Mobile Health Data	IEEE Transactions on Industrial Informatics	10.1109/TII.2021.3113708	9	36	Prediction	Psychology & Psychiatry	Other
Dong Yang, Ziyue Xu, Wenqi Li et al.	2021	Federated semi-supervised learning for COVID region segmentation in chest CT using multi-national data from China, Italy, Japan	Medical Image Analysis	10.1016/j.media.2021.101992	1	186	Segmentation	COVID-19	CT Scan
Saleh Baghersalimi, Tomas Teijeiro, David Atienza et al.	2021	Personalized Real-Time Federated Learning for Epileptic Seizure Detection	IEEE Journal of Biomedical and Health Informatics	https://ieeexplore.ieee.org/document/9479691	3	15	Diagnosis	Neurology	EEG
Akhil Vaid, Suraj K Jaladanki, Jie Xu et al.	2021	Federated Learning of Electronic Health Records to Improve Mortality Prediction in Hospitalized Patients With COVID-19: Machine Learning Approach	JMIR Medical Informatics	10.2196/24207	2	2	Prognosis (including Mortality)	COVID-19	Electronic Health Records / Text
Jianfei Cui, He Zhu, Hao Deng et al.	2021	FeARH: Federated machine learning with anonymous random hybridization on electronic medical records	Journal of Biomedical Informatics	10.1016/j.jbi.2021.103735	4	8	Mortality	Various	Electronic Health Records / Text
Yoo Jeong Ha, Minjae Yoo, Gusang Lee et al.	2021	Spatio-Temporal Split Learning for Privacy-Preserving Medical Platforms: Case Studies With COVID-19 CT, X-Ray, and Cholesterol Data	IEEE Access	10.1109/ACCESS.2021.3108455	1	10	Diagnosis	Other Respiratory	X-Ray
Mohd Adli MD Ali, Edre Mohammad Aidid, Hafidzul Abdullah	2021	Respecting Patient Privacy with Federated Artificial Intelligence	Journal of Information Systems and Digital Technologies	https://journals.iium.edu.my/kict/index.php/jisdt/article/view/220	2	21	Diagnosis	Other Respiratory	X-Ray
Ricardo R. Lopes, Marco Mamprin, Jo M. Zelis et al.	2021	Local and Distributed Machine Learning for Inter-hospital Data Utilization: An Application for TAVI Outcome Prediction	Frontiers in Cardiovascular Medicine	10.3389/fcvm.2021.787246	20	40	Mortality	Cardiology	Electronic Health Records / Text, Unstated
Haeyun Lee, Young Jun Chai, Hyunjin Joo et al.	2021	Federated Learning for Thyroid Ultrasound Image Analysis to Protect Personal Information: Validation Study in a Real Health Care Environment	JMIR Medical Informatics	10.2196/25869	5	70	Diagnosis	Oncology	Ultrasound
Jessica Chia Liu, Jack Goetz, Srijan Sen et al.	2021	Learning From Others Without Sacrificing Privacy: Simulation Comparing Centralized and Federated Machine Learning on Mobile Health Data	JMIR mHealth and uHealth	10.2196/23728	2	4	Diagnosis	Others	Other
Ji Ae Park, Min Dong Sung, Ho Heon Kim et al.	2021	Weight-Based Framework for Predictive Modeling of Multiple Databases With Noniterative Communication Without Data Sharing: Privacy-Protecting Analytic Method for Multi-Institutional Studies	JMIR Medical Informatics	10.2196/21043	4	125	Mortality	Systemic	Electronic Health Records / Text
Mustafa Abdul Salam, Sanaa Taha, Mohamed Ramadan	2021	COVID-19 detection using federated machine learning	PLoS One	10.1371/journal.pone.0252573	5	12	Prognosis, Diagnosis	COVID-19	Electronic Health Records / Text, X-Ray
Xiaodong Wang, Zhen’an He, Ying Wang et al.	2021	An Intestinal Centerline Extraction Algorithm Based on Federated Framework	Wireless Communications and Mobile Computing	10.1155/2021/2979214	2	6	Segmentation	Gastric & Digestive	CT Scan
Dinh C. Nguyen, Ming Ding, Pubudu N. Pathirana et al.	2021	Federated Learning for COVID-19 Detection With Generative Adversarial Networks in Edge Cloud Computing	IEEE Internet of Things Journal	10.1109/jiot.2021.3120998	4	30	Diagnosis	COVID-19	X-Ray
Georgios Kaissis, Alexander Ziller, Jonathan Passerat-Palmbach et al.	2021	End-to-end privacy preserving deep learning on multi-institutional medical imaging	Nature Machine Intelligence	10.1038/s42256-021-00337-8	3	54	Diagnosis	COVID-19	X-Ray
Qi Dou, Tiffany Y. So, Meirui Jiang et al.	2021	Federated deep learning for detecting COVID-19 lung abnormalities in CT: a privacy-preserving multinational validation study	npj Digital Medicine	10.1038/s41746-021-00431-6	1	48	Diagnosis	COVID-19	CT Scan
Stefanie Warnat-Herresthal, Hartmut Schultze, Krishnaprasad Lingadahalli Shastry et al.	2021	Swarm Learning for decentralized and confidential clinical machine learning	Nature	10.1038/s41586-021-03583-3	4	1,309	Diagnosis	Oncology, Other Respiratory, COVID-19	Genome, Other
Julian Lo, Timothy T. Yu, Da Ma et al.	2021	Federated Learning for Microvasculature Segmentation and Diabetic Retinopathy Classification of OCT Data	Ophthalmology Science	10.1016/j.xops.2021.100069	1	80	Segmentation + Classification	Diabetes	OCTA + OCT
Fadila Zerka, Visara Urovi, Fabio Bottari et al.	2021	Privacy preserving distributed learning classifiers – Sequential learning with small sets of data	Computers in Biology and Medicine	10.1016/j.compbiomed.2021.104716	36	36	Diagnosis, Prognosis	Oncology, Gastroenterology	Pathology / Whole slide images, Electronic Health Records / Text
Ines Feki, Sourour Ammar, Yousri Kessentini et al.	2021	Federated learning for COVID-19 screening from Chest X-ray images	Applied Soft Computing	10.1016/j.asoc.2021.107330	4	24	Diagnosis	COVID-19	X-Ray
Xiaoxiao Li, Yufeng Gu, Nicha Dvornek et al.	2020	Multi-site fMRI analysis using privacy-preserving federated learning and domain adaptation: ABIDE results	Medical Image Analysis	10.1016/j.media.2020.101765	4	44	Diagnosis	Psychology & Psychiatry	MRI
M. Lincy, Dr. A. Meena Kowshalya	2020	Early Detection of Type-2 Diabetes Using Federated Learning	International Journal of Scientific Research in Science Engineering and Technology	10.32628/IJSRSET207644	3	6	Diagnosis	Diabetes	Unstated
Mohammed Alawad, Hong-Jun Yoon, Shang Gao et al.	2020	Privacy-Preserving Deep Learning NLP Models for Cancer Registries	IEEE Transactions on Emerging Topics in Computational Intelligence	10.1109/tetc.2020.2983404	3	32	Segmentation, Segmentation + Classification	COVID-19, Oncology	CT Scan, Electronic Health Records / Text
Xiaoye Qian, Huan Chen, Haotian Jiang et al.	2020	Wearable Computing With Distributed Deep Learning Hierarchy: A Study of Fall Detection	IEEE Sensors Journal	10.1109/jsen.2020.2988667	1	4	Diagnosis	Others	Unstated
Zengqiang Yan, Jeffry Wicaksana, Zhiwei Wang et al.	2020	Variation-Aware Federated Learning With Multi-Source Decentralized Medical Image Data	IEEE Journal of Biomedical and Health Informatics	10.1109/jbhi.2020.3040015	2	80	Diagnosis	Oncology	MRI
Fadila Zerka, Visara Urovi, Akshayaa Vaidyanathan et al.	2020	Blockchain for Privacy Preserving and Trustworthy Distributed Machine Learning in Multicentric Medical Imaging (C-DistriM)	IEEE Access	https://ieeexplore.ieee.org/document/9216036	1	24	Prognosis	Oncology	CT Scan
Niranjan Balachandar, Ken Chang, Jayashree Kalpathy-Cramer et al.	2020	Accounting for data variability in multi-institutional distributed deep learning for medical imaging	Journal of the American Medical Informatics Association	10.1093/jamia/ocaa017	10	60	Diagnosis	Diabetes, Various	Retina fundus image, X-Ray
Marta Bogowicz, Arthur Jochems, Timo M. Deist et al.	2020	Privacy-preserving distributed learning of radiomics to predict overall survival and HPV status in head and neck cancer	Scientific Reports	10.1038/s41598-020-61297-4	10	10	Prediction, Mortality	Others	Electronic Health Records / Text
Noah Lewis, Harshvardhan Gazula, Sergey M. Plis et al.	2020	Decentralized distribution-sampled classification models with application to brain imaging	Journal of Neuroscience Methods	10.1016/j.jneumeth.2019.108418	3	5	Diagnosis	Psychology & Psychiatry	MRI
Elena Czeizler, Wolfgang Wiessler, Thorben Koester et al.	2020	Using federated data sources and Varian Learning Portal framework to train a neural network model for automatic organ segmentation	Physica Medica	10.1016/j.ejmp.2020.03.011	3	6	Segmentation	Nephrology, Gastric & Digestive, Others	CT Scan
Samuel W. Remedios, Snehashis Roy, Camilo Bermudez et al.	2020	Distributed deep learning across multisite datasets for generalized CT hemorrhage segmentation	Medical Physics	10.1002/mp.13880	1	8	Segmentation	Neurology	CT Scan
Li Huang, Andrew L. Shea, Huining Qian et al.	2019	Patient clustering improves efficiency of federated machine learning to predict mortality and hospital stay time using distributed electronic medical records	Journal of Biomedical Informatics	10.1016/j.jbi.2019.103291	10	20	Prognosis (including Mortality)	Systemic	Electronic Health Records / Text
Theodora S Brisimi, Ruidi Chen, Theofanie Mela et al.	2018	Federated learning of predictive models from federated Electronic Health Records	International Journal of Medical Informatics	10.1016/j.ijmedinf.2018.01.007	2	6	Prediction	Cardiology	Electronic Health Records / Text
Petr Dluhoš, Daniel Schwarz, Wiepke Cahn et al.	2017	Multi-center machine learning in imaging psychiatry: A meta-model approach	NeuroImage	10.1016/j.neuroimage.2017.03.027	1	96	Diagnosis	Psychology & Psychiatry	MRI
Arthur Jochems, Timo M. Deist, Issam El Naqa et al.	2017	Developing and Validating a Survival Prediction Model for NSCLC Patients Through Distributed Learning Across 3 Countries	International Journal of Radiation Oncology*Biology*Physics	10.1016/j.ijrobp.2017.04.021	1	1	Mortality	Oncology	Electronic Health Records / Text
Timo M. Deist, A. Jochems, Johan van Soest et al.	2017	Infrastructure and distributed learning methodology for privacy-preserving multi-centric rapid learning health care: euroCAT	Clinical and Translational Radiation Oncology	10.1016/j.ctro.2016.12.004	5	5	Prognosis	Oncology	Electronic Health Records / Text

**Table 2:** Summary of broader clinical domains coverage, per number of articles, number of models and number of comparisons

Broader Clinical Domain	Number of Articles	Number of Models	Number of Comparisons
Oncology	44	184	1780
COVID-19	35	84	2171
Others	18	78	422
Neurology	17	53	629
Cardiology	16	85	515
Other Respiratory	12	29	763
Psychology & Psychiatry	12	36	639
Diabetes	8	21	191
Gastric & Digestive	8	45	409
Various	7	36	378
Systemic	4	19	206
Nephrology	3	4	44
Gastroenterology	1	9	9

**Table 3:** Summary of clinical application coverage, per number of articles, number of models and number of comparisons

Clinical Application	Number of Articles	Number of Models	Number of Comparisons
Diagnosis	93	335	5032
Segmentation	28	132	1245
Prediction	22	70	522
Prognosis (including Mortality)	18	94	408
Segmentation + Classification	8	32	845
Other	2	17	89
Therapy	1	3	15

**Table 4:** Number of Articles Published, per Year

Publication Year	Number of Articles
2024	20
2023	65
2022	37
2021	22
2020	11
2019	1
2018	1
2017	3

**Table 5:** Number of Published Articles by Publication Sources with more than one Article Published

Publication Source	Number of Articles
Scientific Reports	12
IEEE Journal of Biomedical and Health Informatics	8
IEEE Transactions on Medical Imaging	6
Electronics	5
IEEE Access	5
Medical Image Analysis	5
JMIR Medical Informatics	4
Mathematics	4
Computer Methods and Programs in Biomedicine	3
Journal of Biomedical Informatics	3
Applied Sciences	2
Bioengineering	2
Biomedical Signal Processing and Control	2
Computers in Biology and Medicine	2
Diagnostics	2
Engineering Applications of Artificial Intelligence	2
Future Generation Computer Systems	2
IEEE Transactions on Emerging Topics in Computational Intelligence	2
IEEE Transactions on Network and Service Management	2
Journal of Healthcare Informatics Research	2
Journal of the American Medical Informatics Association	2
Medical Physics	2
Nature Machine Intelligence	2
Nature Medicine	2
Patterns	2
The Lancet Digit Health	2

Regarding the decentralized models, the most common TRIPOD type was 2a (74 articles), followed by types 1b, 2b and 3 (26, 23, and 23 articles respectively), with 11 being unclear and only 3 classified as type 1a. Concerning code access, 106 articles did not report code availability, 43 provided all code, 6 provided some code, 4 explicitly stated no code would be available, and 1 had pending code access requests. For data access, 64 articles provided all data, 36 did not report data access, 32 had pending data access requests, 20 provided some data, and 8 provided no data access.

Regarding the model architectures used, Federated Learning was the most common (557 models), followed by Fully Decentralized approaches, including Swarm Learning (111 models), Ensemble methods (21), Split or Transfer Learning (14), and Secure Multi-Party Computation (4). Most models (687) use real data, while only 16 use synthetic data and 5 use both types.

In terms of the data used, Electronic Health Records/Text is the most commonly used data type (121 models). Image-based data was used very frequently, namely MRI (101 models), X-Ray (96 models), Pathology/Whole slide images (67 models), CT Scans (65 models), retina fundus images (37 models), ECG/EKG (27 models), dermoscopic images (21 models), EEG (5 models) and ultrasound (5 models). Genome data was used in 33 models. Some models use combinations of data types, such as Electronic Health Records/Text + X-Ray (12 models) and MRI + Pathology/Whole slide images (6 models). Less frequent data types were endoscopic videos (17 models), laparoscopic cholecystectomy videos (16 models), and wireless capsule endoscopy videos (4 models). The least common data types, used by only 1 or 2 models, include Mammography, Microscopy, Intra-Oral Mesh Scans, OCTA + OCT and PET images.

**Fig. 3:**
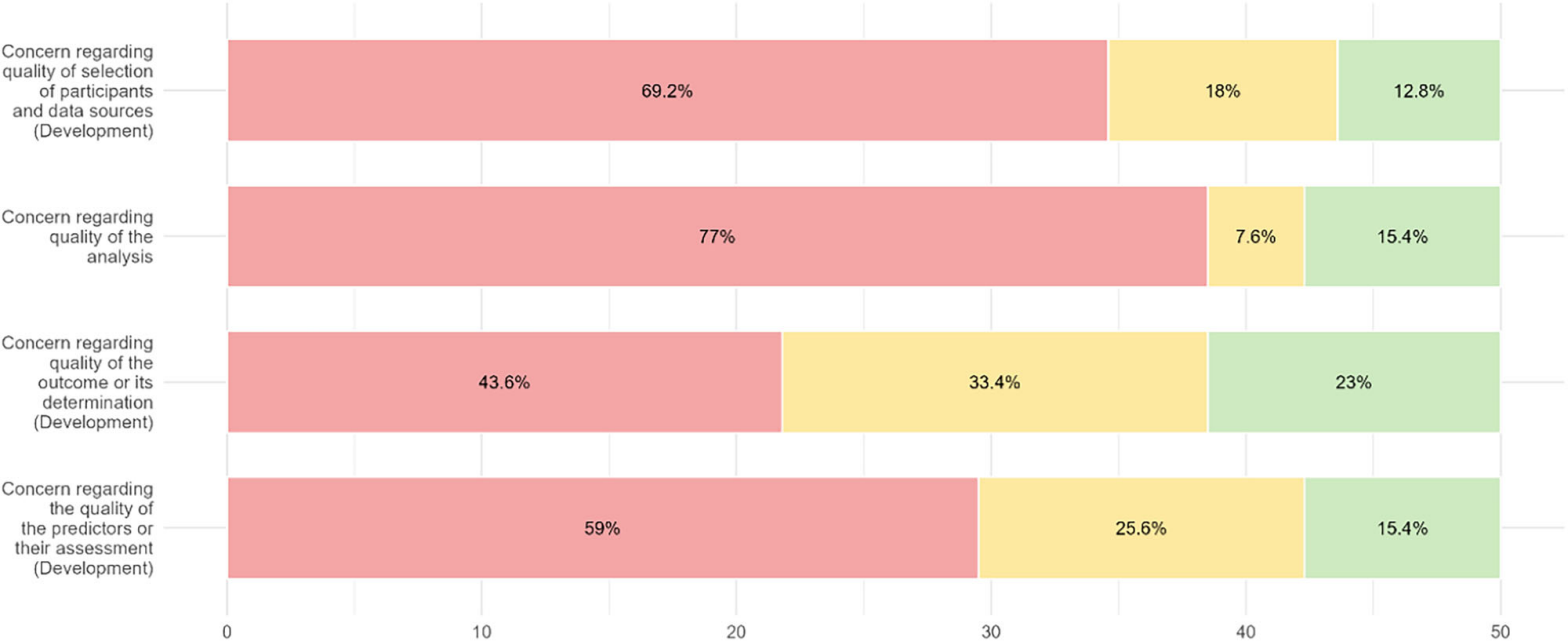
Results of PROBAST + AI Application - Quality Concern. Red segments, yellow segments and green segments represent the proportion of classifications of high concern level, unclear concern level and low concern level.

**Fig. 4:**
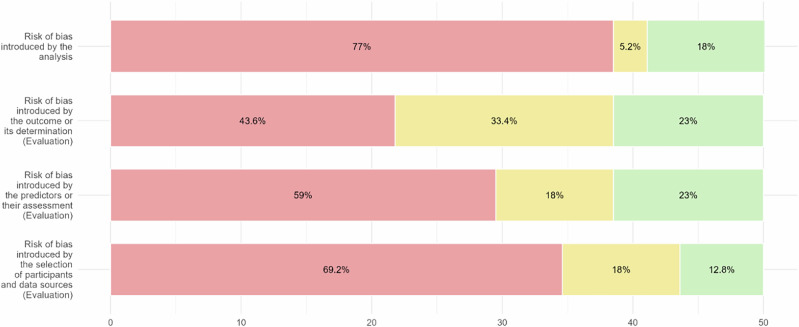
Results of PROBAST + AI Application - Risk of Bias Assessment. Red segments, yellow segments and green segments represent the proportion of classifications of high risk of bias, unclear risk of bias and low risk of bias.

**Fig. 5:**
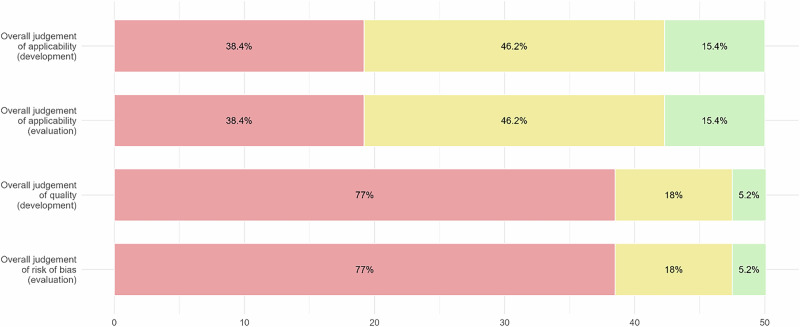
Results of PROBAST + AI Application - Overall Judgement. Red segments, yellow segments and green segments represent the proportion of classifications of high concern level, unclear concern level and low concern level.

**Fig. 6:**
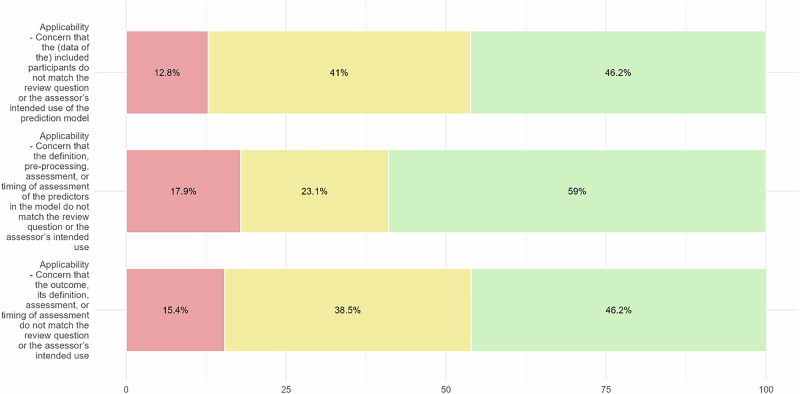
Results of PROBAST + AI Application – Applicability. Red segments, yellow segments and green segments represent the proportion of classifications of high concern level, unclear concern level and low concern level.

Using the PROBAST + AI tool, we appraised the 25 most cited articles of the TRIPOD Types 2a, 2b and 3, considering up to two models for each article (Figs. 3–6).

### Performance comparison

We grouped our findings by the main types of model evaluations conducted (i.e., combinations of broader clinical domain, clinical application and performance metric). Regarding centralized learning models, as presented in Table 6, the most common evaluation was for diagnostic accuracy in Oncology (53 models and 189 comparisons). Other frequent clinical domains include COVID-19 and Cardiology. Regarding local learning models, as presented in Table 7, the most common evaluation was for diagnostic accuracy in COVID-19 (31 models and 195 comparisons). Similarly, Oncology and Cardiology were also frequently explored.

**Table 6:** Number of Models and Comparisons per Group of Broader Clinical Domain, Performance Metric and Clinical Application, with Median, 25th Percentile and 75th Percentile of Centralized Models Performances (10 models or more)

Broader Clinical Domain	Performance Metric	Clinical Application	Number of Models	Number of Comparisons	Median	25th Percentile	75th Percentile
Oncology	Accuracy	Diagnosis	53	189	0.853	0.768	0.972
Oncology	AUROC	Diagnosis	39	140	0.910	0.891	0.996
Oncology	AUROC	Prognosis (including Mortality)	36	59	0.727	0.700	0.970
Oncology	Dice score	Segmentation	33	138	0.873	0.800	0.907
COVID-19	Accuracy	Diagnosis	32	183	0.913	0.910	0.970
Cardiology	AUROC	Diagnosis	30	129	0.856	0.797	0.903
Cardiology	Dice score	Segmentation	22	58	0.886	0.883	0.914
Neurology	Accuracy	Diagnosis	22	162	0.803	0.779	0.842
Others	Dice score	Segmentation	21	57	0.882	0.870	0.895
Oncology	Sensitivity/Recall	Diagnosis	19	82	0.863	0.832	0.937
COVID-19	Sensitivity/Recall	Diagnosis	17	78	0.930	0.896	0.940
Oncology	95th percentile of the Hausdorff Distance	Segmentation	17	21	4.565	4.565	4.565
Oncology	F1 score	Diagnosis	17	51	0.888	0.829	0.983
Psychology & Psychiatry	Accuracy	Diagnosis	17	138	0.710	0.680	0.818
Gastric & Digestive	F1 score	Other	16	88	0.633	0.465	0.725
Oncology	Precision/Positive Predictive Value	Diagnosis	16	59	0.811	0.743	0.984
Others	Mean segmentation intersection-over-union	Segmentation	16	27	0.803	0.785	0.826
Others	Precision/Positive Predictive Value	Segmentation	15	15	0.891	0.887	0.928
Others	Sensitivity/Recall	Segmentation	15	15	0.863	0.808	0.872
Other Respiratory	AUROC	Diagnosis	14	293	0.721	0.628	0.833
Systemic	AUROC	Prognosis (including Mortality)	14	135	0.854	0.800	0.859
COVID-19	AUROC	Diagnosis	12	89	0.950	0.906	0.992
COVID-19	F1 score	Diagnosis	12	63	0.872	0.562	0.942
Oncology	Specificity	Diagnosis	12	61	0.865	0.700	0.986
COVID-19	Accuracy	Segmentation + Classification	11	176	0.992	0.988	0.994
COVID-19	Dice score	Segmentation + Classification	11	220	0.732	0.722	0.804
COVID-19	Sensitivity/Recall	Segmentation + Classification	11	176	0.694	0.591	0.800
Cardiology	AUROC	Prognosis (including Mortality)	11	11	0.734	0.732	0.735
COVID-19	Dice score	Segmentation	10	231	0.590	0.575	0.615
COVID-19	Precision/Positive Predictive Value	Diagnosis	10	43	0.946	0.879	0.963
Systemic	PR-AUC	Prognosis (including Mortality)	10	10	0.130	0.114	0.145
Various	Dice score	Segmentation	10	68	0.922	0.845	0.953

**Table 7:** Number of Models and Comparisons per Group of Broader Clinical Domain, Performance Metric and Clinical Application, with Median, 25th Percentile and 75th Percentile of Local Models Performances (10 models or more)

Broader Clinical Domain	Performance Metric	Clinical Application	Number of Models	Number of Comparisons	Median	25th Percentile	75th Percentile
COVID-19	Accuracy	Diagnosis	31	195	0.923	0.824	0.979
COVID-19	Sensitivity/Recall	Diagnosis	26	183	0.915	0.855	0.961
Oncology	Dice score	Segmentation	26	110	0.859	0.790	0.895
Oncology	Accuracy	Diagnosis	25	187	0.953	0.823	0.983
COVID-19	F1 score	Diagnosis	21	151	0.930	0.771	0.967
COVID-19	Precision/Positive Predictive Value	Diagnosis	21	156	0.956	0.879	0.987
Cardiology	AUROC	Prognosis (including Mortality)	21	51	0.630	0.610	0.670
Cardiology	AUROC	Diagnosis	18	40	0.901	0.823	0.910
Oncology	95th percentile of the Hausdorff Distance	Segmentation	17	17	5.163	5.054	6.120
Gastric & Digestive	F1 score	Other	16	88	0.470	0.386	0.585
Oncology	Sensitivity/Recall	Diagnosis	14	132	0.959	0.817	0.984
Various	Accuracy	Diagnosis	14	14	0.745	0.734	0.812
COVID-19	AUROC	Diagnosis	13	120	0.984	0.927	0.998
Oncology	AUROC	Diagnosis	12	128	0.983	0.913	0.998
COVID-19	Accuracy	Segmentation + Classification	11	176	0.991	0.983	0.993
COVID-19	Dice score	Segmentation + Classification	11	220	0.702	0.605	0.753
COVID-19	Sensitivity/Recall	Segmentation + Classification	11	176	0.582	0.524	0.742
Oncology	AUROC	Prognosis (including Mortality)	11	11	0.738	0.730	0.761
Oncology	Precision/Positive Predictive Value	Diagnosis	11	99	0.969	0.871	0.985
Neurology	Dice score	Segmentation	10	53	0.917	0.899	0.922
Oncology	F1 score	Diagnosis	10	99	0.977	0.945	0.981
Various	AUROC	Diagnosis	10	80	0.865	0.805	0.912
Various	Sensitivity/Recall	Diagnosis	10	10	0.780	0.755	0.845
Various	Specificity	Diagnosis	10	10	0.738	0.730	0.808

In terms of performance comparisons, the paired differences between decentralized and non-decentralized learning approaches are summarized in Fig. 7. Supplementary Figs. 2–15 present the distribution these differences for each performance metric and type of non-decentralized model, alongside additional data (e.g., 25th and 75th percentiles of differences, 95% confidence intervals). To describe the magnitude of these differences, the effect sizes for these comparisons are provided in Fig. 8.

**Fig. 7:**
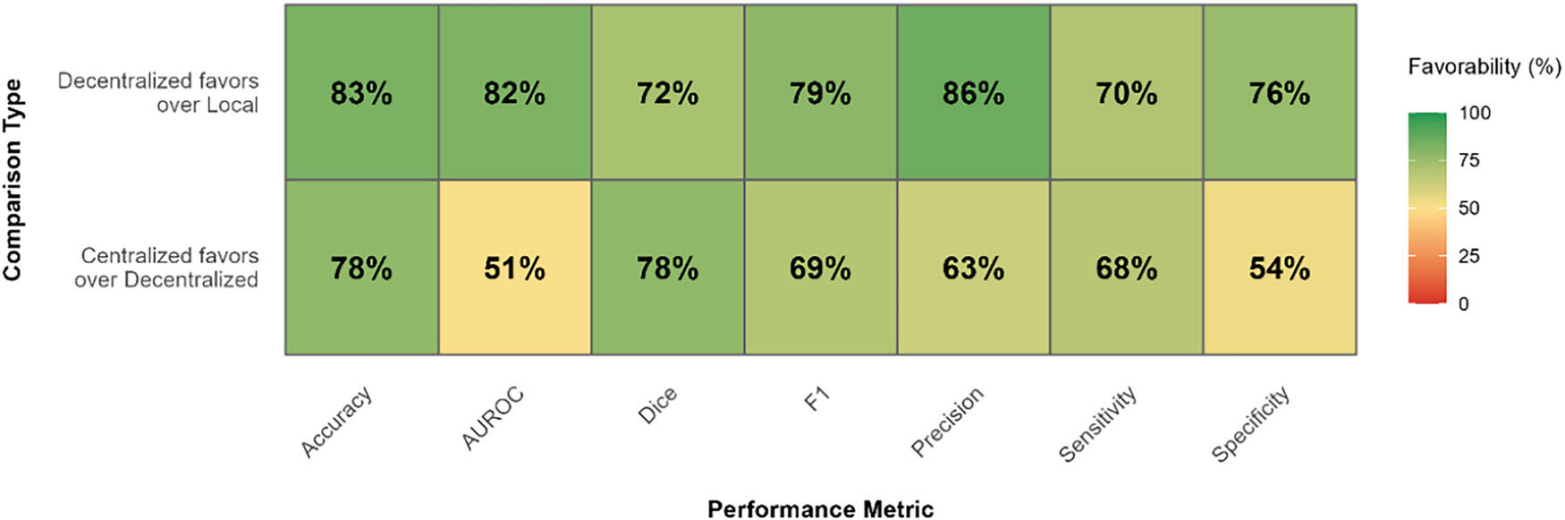
Performance comparison summary of favourability across metrics. Higher percentage corresponds to increase favourability. Row 1 represents the proportion of paired comparisons in which Decentralized Learning overperforms Local Learning. Row 2 represents the proportion of paired comparisons in which Centralized Learning overperforms Decentralized Learning.

**Fig. 8:**
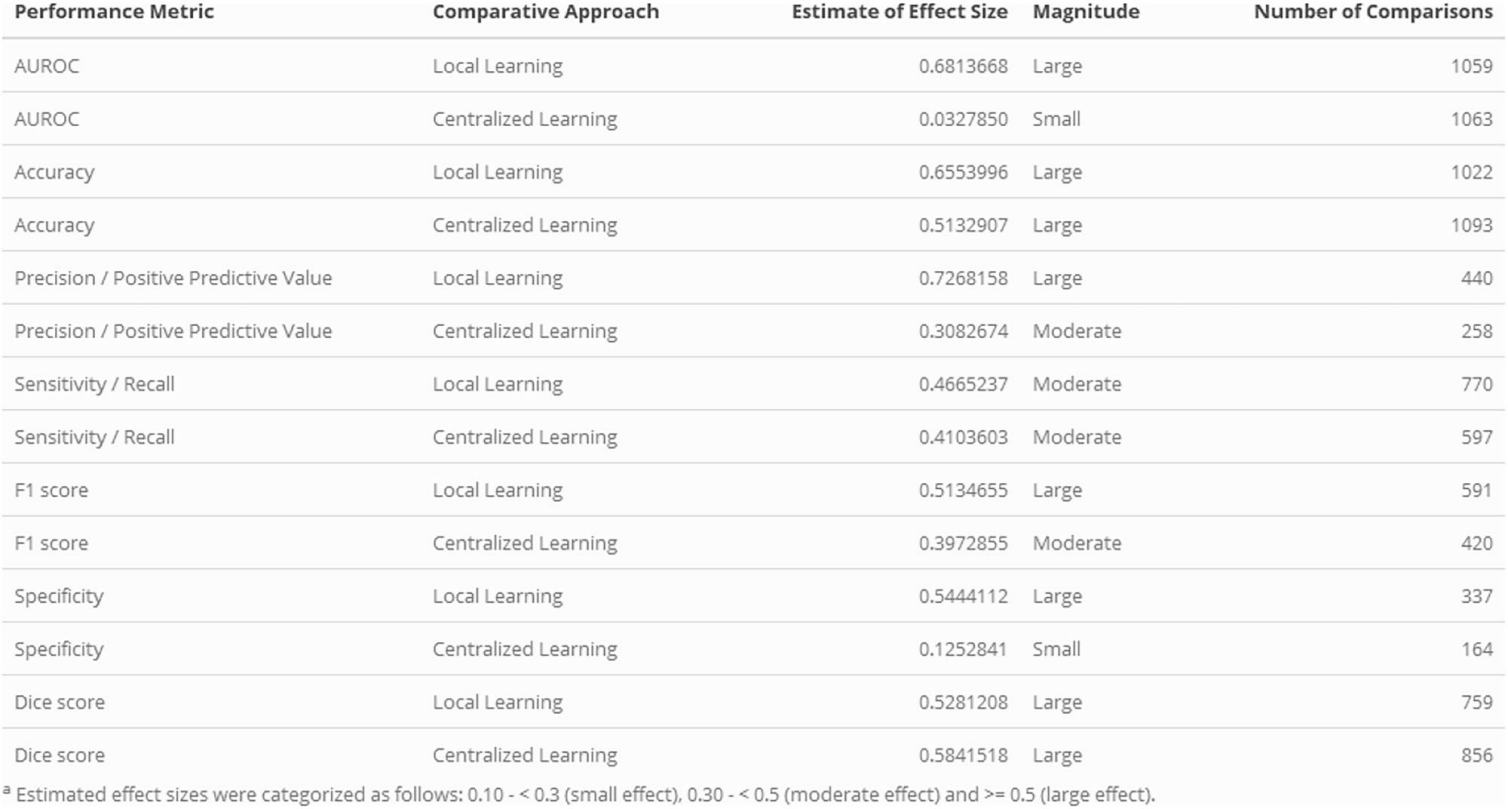
Estimation of Effect Sizes using Wilcoxon two-sample paired signed-rank test. The table presents effect sizes calculated using the Wilcoxon two-sample paired signed-rank test across seven performance metrics (AUROC, Accuracy, Precision/Positive Predictive Value, Sensitivity/Recall, F1 score, Specificity, and Dice score). Each metric is compared against both Local Learning and Centralized Learning approaches using the corresponding Decentrralized Learning values. Columns display the estimate of effect size, magnitude classification, and number of comparisons.

### Centralized learning

Overall, centralized learning presents a clear performance advantage across different metrics. This approach is particularly superior to decentralized learning in accuracy and Dice score, with a higher performance in 78% of 1089 comparisons and 78% of 856 comparisons respectively, with narrow interquartile ranges and large effect sizes (≥0.5).

In contrast, AUROC and specificity comparisons present a more balanced distribution, with centralized learning performing better in 51% of 1063 comparisons and 54% of 160 comparisons, respectively. For both metrics, the effect sizes estimated are small (<0.3). The remaining metrics also favor centralized learning, with less pronounced favourability (from 63% to 69%), wider interquartile ranges and moderate effect size.

### Local learning

In contrast, decentralized learning models consistently outperform their local counterparts. In particular, accuracy and precision/positive predictive value metrics, performing better in 83% of 1023 comparisons and 86% of 440 comparisons respectively, featuring large effect sizes. Similarly, strong preference is observed in AUROC and F1 score comparisons, with decentralized learning models being superior in 82% and 79% of cases, respectively, in association with large effect sizes.

The decentralized learning advantage is smaller for Dice score and specificity metrics (72% and 76% of comparisons, respectively), while still featuring large effect sizes. Sensitivity/recall shows decentralized learning performing better in 71% of cases, with a wider interquartile range and a moderate effect size.

### Performance difference significance

Considering the direction, magnitude and statistical relevance of the performance differences, we subsequently explored the clinical relevance of these findings using a clinical viability threshold (≥0.80).

**Fig. 9:**
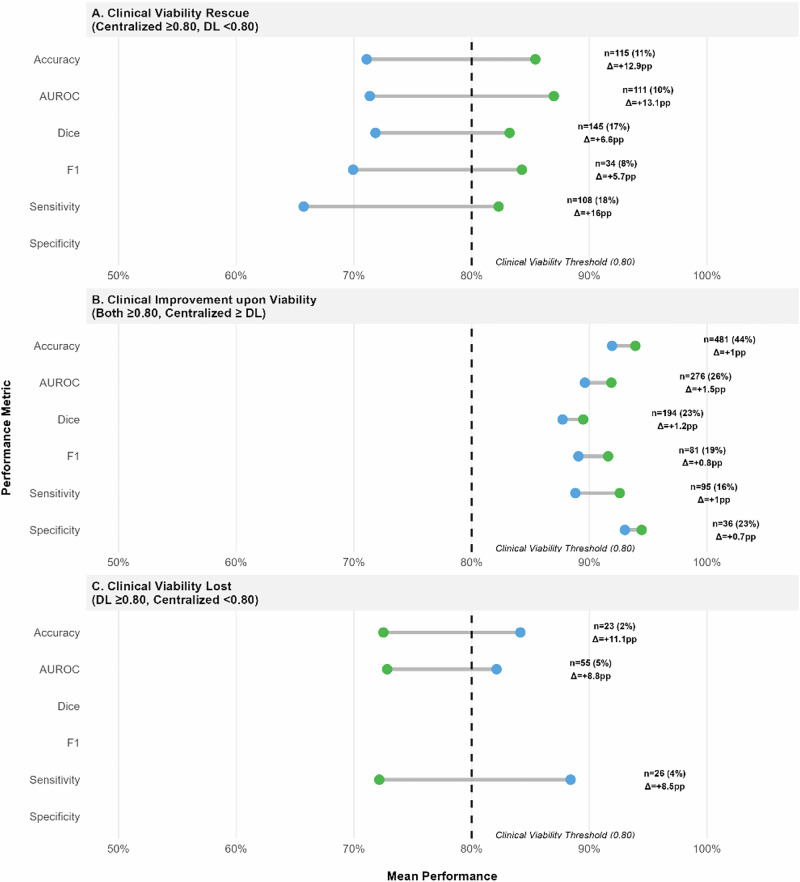
Clinical significance focused comparison between Decentralized Learning and Centralized Learning. Blue = Decentralized Learning; Green = Centralized Learning; Gray line = Difference. Clinical viability threshold set at 80%. Lines connect DL to centralized model performance. Δ indicates median performance difference (only comparisons with *n* ≥ 20 shown). **A** Centralized model rescues clinical viability. **B** Both models are viable but centralized performs better. **C** Centralized model loses viability compared to viable DL model.

When comparing centralized and decentralized models, centralized approaches frequently rescued clinical viability from underperforming DL models (Fig. 9, Panel A). Specifically, centralized models provided clinically valid alternatives to DL in up to 18% of comparisons, particularly for sensitivity (median difference of 16pp) and accuracy metrics. Conversely, even when both models achieved clinical viability (≥0.80), centralized approaches typically demonstrated only marginal advantages (Panel B), with median performance differences ranging from 0.7 to 1.5 percentage points across metrics. DL models rarely offered superior alternatives to viable centralized counterparts (Panel C).

**Fig. 10:**
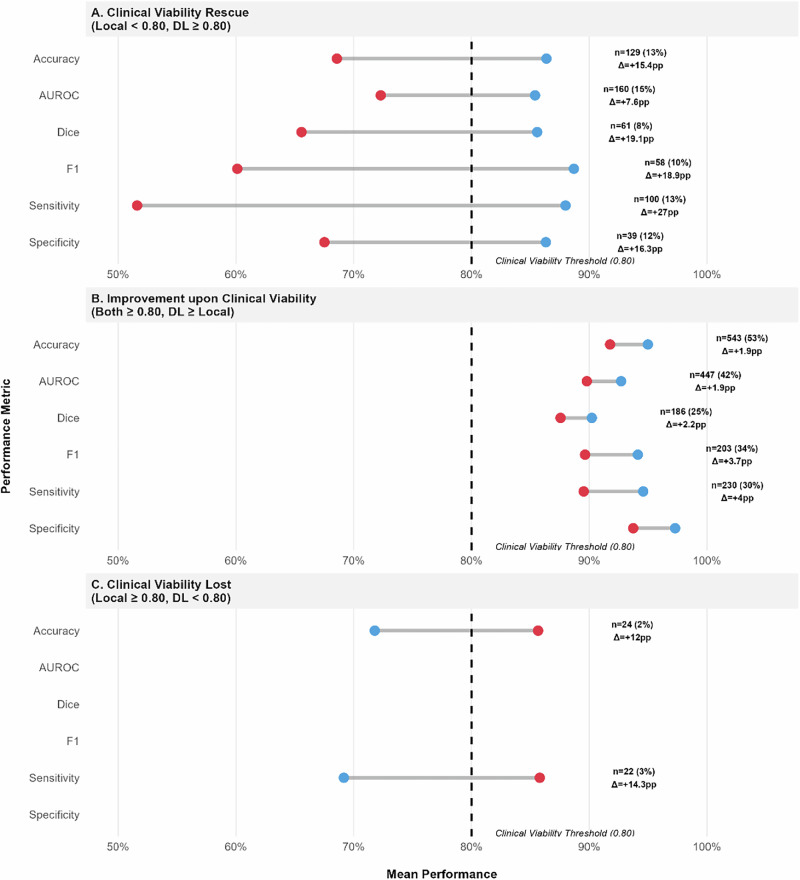
Clinical significance focused comparison between Decentralized Learning and Local Learning. Blue = Decentralized Learning; Red = Local Learning; Gray line = Difference. Clinical viability threshold set at 80%. Lines connect DL to local model performance. Δ indicates median performance difference (only comparisons with *n* ≥ 20 shown). **A** Decentralized model rescues clinical viability. **B** Both models are viable but DL performs better. **C** Centralized model loses viability compared to viable DL model.

In comparisons with local models, DL consistently rescued clinical viability across substantial proportions of cases (Fig. 10, Panel A). The rescue effect was most pronounced for threshold-dependent metrics, particularly sensitivity (median difference of 27pp for 100 comparisons) compared to ranking metrics like AUROC (median difference of 7.6pp). DL provided clinically acceptable alternatives across metrics with median improvements ranging from 7.6 to 27 percentage points. When both approaches met the viability threshold (Panel B), DL maintained performance advantages, with accuracy superior in 53% of comparisons (median difference of 1.9pp). DL underperformance against viable local models was rare (Panel C) and associated with significant losses.

Detailed metric-specific analyses are provided as Supplementary Material.

### Additional privacy-preserving techniques and secondary aims

On the technical side, we report details of the methods used (e.g., encryption, GAN, homomorphic encryption) strictly based on the original manuscripts. On the resources demand side, we report details on various metrics–ranging from energy consumption, server and client memory requirements to time needed for model training and model inference. This information is presented as Supplementary Material.

#### Supplementary analyses

For further granularity, we developed an online dynamic dashboard to produce different graphical representations of data collected. Available filters are performance metrics, non-decentralized learning approach, clinical applications, larger clinical domains and data type. Additionally, it is possible to restrict decentralized model comparisons to only federated learning models.

To complement the analyses of the performance distributions, we provide histogram representations by nature of data collection used by the models compared (“primary”, “secondary” and “both”) in Supplementary Figs. 23–36.

Additionally, we produced sub-analyses on the distribution of the absolute differences (i.e., the difference between non-decentralized approach and decentralized approach), as well as on distribution of the relative differences (i.e., the quotient of the absolute differences with the difference between 1 and the non-decentralized approach). These representations correspond to Supplementary Figs. 37–64.

## Discussion

This systematic review provides the most comprehensive analysis to date comparing decentralized learning approaches with traditional methods in healthcare, examining 160 studies comprising 710 decentralized models and 8149 performance comparisons.

The rapid growth in research output, particularly since 2020 and multidisciplinary scope reflects increasing recognition of decentralized learning’s potential in healthcare applications.

Considering the paired comparisons between decentralized and centralized methods, performance differences present low magnitude median values and reduced interquartile ranges. This demonstrates that decentralized approaches can broadly achieve comparable performance, although moderately inferior. In particular, strong relative performance in AUROC (51% centralized favourability, small effect size) suggests that the observation ranking ability is preserved through decentralized learning processes. In turn, threshold-dependent metrics—such as accuracy and Dice score– show increased centralized relative advantages, with mostly moderate or large effect sizes. These findings reveal calibration challenges and spatial feature averaging difficulties, respectively. However, DL seems to overperform in terms of specificity (54% centralized favourability, small effect size), suggesting that aggregation processes differentially affect error types. In particular, multi-site learning may filter site-specific false positive patterns while simultaneously diluting rare positive case signals, given case presentation variation and uneven distribution of rare cases across sites.

Focusing on the application viability of these models, centralized models can offer clinically useful alternatives to underperforming decentralized counterparts, in up to 18% of the cases. Sensitivity and accuracy are particularly benefited by the centralized approach, aligning with DL limitations to identify true positive cases.

Regarding the differences with local approaches across all metrics, decentralized performance is dominant, despite some heterogeneity. While, decentralized models benefit from more and often more diverse data, they are less tuned to the specific distribution of a local dataset. In particular, DL demonstrates the strongest advantage in precision (86% favourability), substantially exceeding gains in other metrics.

This likely reflects multi-site models’ ability to filter out site-specific artifacts (e.g., differences in imaging protocols, scanner calibration). Local models overfit to these artifacts, leading to overconfident predictions that inflate false positive rates when encountering variation. In turn, sensitivity shows the smallest DL advantage (70%), with only a moderate effect size, likely reflecting challenges in aggregating rare or subtle pathological patterns across heterogeneous sites. Specificity shows greater improvement (76% favourability), as normal imaging features are more consistent across sites than disease presentations, and DL models learn to avoid falsely flagging benign site-specific variations. This asymmetry reflects a fundamental trade-off: local models can optimize to site-specific patterns—potentially overfitting—at the expense of external validity, whereas DL prioritizes features robust across heterogeneous sites. A similar pattern emerges when comparing DL to centralized models, where challenges in aggregating rare signals similarly constrain sensitivity improvements.

With our sensitivity analysis, excluding observations from articles with the most comparisons, variations in favourability ratios were generally within single-digit percentage points. This strengthens the validity of the data presented and our conclusions.

Focusing on clinical applicability, the threshold-stratified analysis (≥0.80) reveals important patterns for implementation decisions. Centralized models can rescue clinical viability from underperforming DL in up to 18% of cases, primarily for sensitivity and accuracy. This aligns with DL’s documented limitations in identifying true positive cases, particularly rare or subtle pathological patterns across heterogeneous sites.

Importantly, the clinical threshold analysis demonstrates that when both centralized and DL approaches achieve clinical viability, centralized superiority typically represents “excellent versus acceptable” performance rather than “acceptable versus inadequate.” While centralized improvements occur frequently, their magnitude is limited (median difference ranging from 0.7pp to 1.5pp). This suggests that when DL models achieve clinically acceptable performance, centralized alternatives provide only modest incremental gains. This positions DL as a viable alternative for contexts where centralized approaches are prohibited by privacy regulations or data sharing constraints.

Regarding differences with local approaches, DL demonstrates dominant performance across all metrics. The clinical rescue effect is substantial, with median improvements of 7.6–27pp depending on metric. The disproportionate improvement in threshold-dependent metrics (27pp for sensitivity) compared to ranking metrics (7.6pp for AUROC) reveals that local models suffer from overfitting to site-specific patterns and class distributions. Decentralized learning mitigates this by learning features robust across heterogeneous clinical settings, resulting in more generalizable decision boundaries. Notably, even when local models achieve clinical viability, DL frequently offers performance increases that should be considered alongside potentially superior external validity.

Regarding additional privacy-preserving techniques and the secondary aims of this study, these data points are reported infrequently and not in a standardized fashion. Even when reported, key variables (e.g., noise levels) are often fixed, making it impossible to assess their impact in each study. Due to differences in datasets, clinical domains, clinical applications or different computational set-ups, cross-study comparisons would not provide reliable insights. Overall, decentralized models are more resource demanding than their counterparts, especially when privacy-preserving methodologies are added.

A qualitative synthesis of the evidence presents some notable patterns. Noise levels of 0.001 provides a superficial level of protection with negligible impacts on performance. Memory and data transmission requirements, outside of resource scarce environments, should not cause significant hardship for model development. While some techniques can increase development time, these rarely duplicate the duration for their standard counterparts. In real-world settings inference time may be a more relevant constraint. Depending on the techniques used, this can lead to compounded increases and may function as an effective bottleneck to the deployment of larger and more complex models.

The findings from this systematic review enable evidence-based decision-making for healthcare AI implementations balancing privacy preservation with clinical performance requirements. To allow actionable application of these insights we propose a simple decision framework.

We start by highlighting when decentralized learning can be recommended. DL represents the optimal approach in three primary scenarios. First, when data sharing is legally prohibited or institutionally restricted (e.g., under GDPR constraints, cross-border regulations or institutional data governance policies), DL enables model development that would otherwise be impossible. Our analysis demonstrates DL achieves clinically acceptable performance (≥0.80) in the majority of applications, with 83% favourability over local approaches for accuracy and 82% for AUROC. Second, when local data alone yields insufficient performance, DL rescues clinical viability in 12% to 15% of cases with substantial improvements (median difference of 7.6–27pp depending on metric). Third, when external validity is prioritized over maximal performance, DL’s multi-site learning reduces site-specific overfitting, particularly valuable for precision metrics where DL shows 86% favourability over local models.

In turn, centralized approaches should be selected when privacy constraints are manageable and maximal performance is required. Centralized models demonstrate advantages in threshold-dependent metrics, particularly accuracy (78% favourability) and Dice score (78% favourability), with large effect sizes. Clinical threshold analysis reveals these advantages typically represent mostly “excellent versus acceptable” rather than “acceptable versus inadequate” performance. When both approaches achieve clinical viability, centralized improvements average only 0.7–1.5pp, in 16% to 44% of comparisons. However, centralized approaches still provide clinically meaningful rescue in 6% to 17% of comparisons. Therefore, centralized learning is justified primarily when: (1) marginal performance improvements are clinically critical, (2) working with rare pathological patterns requiring maximum sensitivity or (3) privacy-preserving infrastructure is unavailable.

Alternatively, local-only approaches should be avoided for deployment across multiple sites or generalizable applications. Local models systematically underperform DL across all metrics, with particularly poor precision (14% favourability) due to overfitting to site-specific artifacts. The 27pp sensitivity improvement from DL versus local models in rescue scenarios indicates local approaches risk missing true positive cases when applied beyond their training environment. Local models may only be appropriate for strictly site-specific applications where external validity is not required and privacy or technical constraints prevent any data collaboration.

Decision-makers should consider that DL’s primary trade-off is not clinical inadequacy but rather marginal performance concessions (typically 1–2pp) for privacy preservation. The resource overhead—while measurable—rarely doubles development time, though inference latency may constrain deployment of complex models. Organizations should prioritize DL when regulatory compliance, institutional policies, or ethical considerations prohibit centralized data aggregation, accepting that performance will be clinically acceptable rather than optimal in most scenarios.

Despite the robustness of this work, some limitations may have affected these results. Publication bias, reporting bias, and selection bias could influence which results are available for inclusion, potentially skewing the aggregated findings. No specific efforts were made to assess or address these. In addition, gray literature or publications outside primary scientific articles were not examined. Our focus on peer-reviewed publications prioritized methodological rigor and clinical applicability, although this approach may have introduced a temporal lag in capturing the most recent developments and reduced the breadth of included results. We mitigated this by searching for published versions of identified preprints and conducting updated searches through March 2024, to balance evidence quality with timeliness. However, a single moment for evidence retrieval and classification would have been preferable. While we aimed for a clear selection and definition of decentralized learning approaches considered, we recognize other interpretations may be valid. However, the majority of data concerns well established methods (e.g., Federated Learning, Swarm Learning). In addition, we recognize some mistakes (i.e., random errors) may have occurred during our extensive process. During our peer-review process, a small number of otherwise eligible papers^33,34^ were by mistake not considered.

Regarding data quality of the included studies, many included articles relied on secondary data or inadequately detailed primary data collection. Both private and public datasets featured instances of insufficient number of participants, observations or predictors, as well as the poor quality of reporting of eligibility criteria, outcome definitions and methods used. In practice, these challenges, alongside inconsistent reporting formats, made identifying different health data models, their characteristics, and performance comparisons more difficult.

Therefore, our evidence appraisal document issues related to the primary studies used. Due to the broad scope of our research question and the comparability of the decentralized and non-decentralized model development and evaluation processes, we believe evidence used to be of low concern for this purpose. Additionally, while clinical applicability performance thresholds vary by application and context, 0.80 provides a standardized benchmark across heterogeneous domains.

Considering the main implications of the study, this systematic review makes three novel contributions to the field: (1) quantification of favourability ratios between traditional and decentralized learning approaches across performance metrics, (2) identification of performance ranges where variations are most pronounced, and (3) clinical significance assessment through threshold-stratified analysis.

This is the first study that presents a quantitative evaluation of the difference between decentralized and non-decentralized approaches at a paired comparison level and grouped by clinical application characteristics. This work demonstrates the ability for DL to present robust ranking assessments, while still struggling to retain positive and rare signals, especially when compared to their centralized counterparts. When considering clinically relevant performance ranges, centralized learning superiority is deepened. Compared to local learning, DL advantages are significant, especially in AUROC, accuracy and precision, and present sizable performance increases, when considering clinical applicability. Therefore, decentralized learning represents a clear superior alternative to local-only approaches, centralized learning continues to be the gold standard. However, DL offers a viable alternative for contexts in which centralized learning is not possible.

As the AI Act advocates for performance parity between traditional and privacy-preserving techniques, the quantitative synthesis of the evidence provides an objective insight for monitoring the state of art and evolution of these approaches. In parallel, our limited findings on privacy-performance trade-off support the need for increase adoption of standardized privacy evaluation metrics. In particular, we recommend more rigorous comparative studies, better documentation of implementation details and focus on practical deployment in healthcare settings. Heterogeneous and infrequent reporting does not allow for an adequate study of dynamics between privacy-preserving guarantees and performance cost.

Considering the issues raised during the evidence appraisal of the most cited, and the variety of specific clinical use cases, these results cannot validate particular implementations for widespread deployment. Problems related to reporting of sampling processes, target population definition and data collection methods compromise external validity of the studies considered. In addition, small variations in performance metrics even for a specific disease can have different clinical and operational impacts (e.g., screening versus diagnosis application). Nonetheless, we encourage the exploration of different sub-analyses in our online dashboard to identify promising research fields.

Comparing this study with similar recent reviews, this work provides a detailed and quantitative assessment of the results from the primary articles. Contemporary research mostly focuses on reporting the article and model characteristics, commonly using narrative syntheses of the primary articles^35–38^. In addition, these works do not provide actionable information on the added benefit of using decentralized approaches in contrast with traditional methods already being used, nor valuable syntheses of the evidence. Moreover, to the best of our knowledge, no published review on the topic was preceded by the respective protocol publication or registry.

In this domain, future research should focus on the impact of local adaptation processes on decentralized learning performance. A two-step paradigm including local calibration learning followed by local calibration may balance privacy preservation, feature generalization and clinically relevant performance. New studies on the topic should present higher methodological quality, with clearer reporting of eligibility criteria, data collection strategies, outcome definitions and model performance comparisons. For privacy-preserving reporting, guiding references—including quantitative and qualitative dimensions – are needed for comparability. While GDPR and AI Act intentionally do not offer specific metrics, there are alternatives^39,40^ from experts on the field.

Other topics regarding the adoption of decentralized learning methods require further discussion. From data distribution challenges to considerable technical overheads and machine “unlearning”^41^ requirements, data collaboration still faces foundational constraints that may limit its widespread adoption. Meanwhile, novel methods such as local fine-tuning pre-trained models, the advent of AI-capable personal devices and normative AI approaches^42^ can help leverage the development of decentralized learning models.

## Methods

### Eligibility criteria

The inclusion criteria were: (1) original and published primary research scientific journal articles; (2) studies addressing clinical decisions regarding specific human medical conditions (e.g., diagnosis, segmentation, prognosis); (3) application of decentralized learning methods for model development; (4) comparison against centralized or local methods; (5) numeric reporting of model performance using at least one relevant metric (e.g., accuracy, precision). While unpublished studies (e.g., pre-prints) and other presentations (e.g., conference proceedings) were retrieved, they were only considered insofar as to search for their corresponding version matching this criterion. Performance metrics were not considered during the screening phase but were used in the appraisal phase. Articles excluded were marked with the first unmet eligibility criterion. Regarding the exclusion criteria, papers published before 2012 were not considered for this analysis.

### Information sources

Eleven databases were queried—covering biomedical scientific research (namely, SpringerLink, Lippincott Williams & Wilkins), computer science and informatics engineering (namely, the Association for Computing and Machinery Digital Library or Guide to Computing Literature and IEEE Xplore), and more general sources (namely, Wiley Online Library, Scopus, Web of Science, and Lens [including the PubMed database]). Additional databases were consulted, including those containing not peer-reviewed papers or unpublished research papers (such as medRxiv and arXiv). The Cochrane Database of Systematic Reviews and PROSPERO registers were consulted on the same dates to identify other ongoing or finished systematic reviews on the topic.

For every listed source, searches were conducted in two moments, retrieving article meta-data from both databases and registries. The first moment concerned articles from January 1st, 2012 until the query dates: April 6th (for all sources, except ACM DL) and April 7th (for ACM DL) 2023. The second moment targeted articles from April 6th, 2023 to those available on March 28th, 2024.

In this stage, articles of different natures (i.e., unpublished, conference proceedings, pre-prints) were considered for retrieval, but only peer-reviewed articles were included when available.

### Search strategy

For searching evidence in this recent and multidisciplinary domain, it was necessary to devise a broad search strategy, considering a large pool of databases and advanced query techniques. A representation of the intended query is seen in Table 8. Due to the popularity of some terms and heterogeneous search engine features, a filtration process was applied, using regular expressions (RegEx) code. Detailed information is available in Supplementary Material.

**Table 8:** General query terms by group

Group	Terms
A – Model Architecture	decentrali*, distributed, federated, central*, multi-party computation, blockchain
B – Model Synonym	learn*, model*, network*, AI, artificial intelligence, ML, machine learning, train*, tensor*, perceptron, algorithm*
C – Health-related	health*, medic*, clinic*, patient*, physician*, doctor*
D – Performance Metrics	AUROC, ROC, receiver operating characteristic curve, F1, Jensen-Shannon, sensitivity, recall, specificity, accuracy, precision, predictive value, Dice, conversion

*AI* artificial intelligence, *ML* machine learning, *AUROC* area under the receiver operating characteristic, *ROC* receiver operating characteristic.

The intended search query results were to include terms from the first and second group separated by no more than two other terms. The order in which they appeared in the title or abstract was not considered relevant.

### Selection process

During the screening phase, titles and abstracts were reviewed. During the appraisal phase, the papers were evaluated using their full-text versions. For each exclusion, the unmet eligibility criterion was registered. For these tasks, the Rayyan^43^ platform was used.

To assess eligibility: (1) reviewers verified publication type using the DOI link or, if unavailable, the title and abstract information and the source to evaluate its nature; (2) clinical applications on specific human medical conditions were verified by identifying specific health targets; (3) application of decentralized learning methods to develop health data models was confirmed when models were trained on data that remained local to each party; (4) comparisons between the decentralized learning models performance and their non-decentralized counterparts supported by the presence of local (i.e., data from a single silo) and centralized learning strategies (i.e., combined data from multiple parties); (5) model performance comparisons were gathered based on written numeric data in the manuscript text or within tables, graphs, and figures, if the numeric information and the model they represent were clear. Efforts were made to also include data from the Supplementary Material.

To classify different model development approaches, we adopted an operational framework we used, based on two core dimensions. Approaches were classified based on data movement (i.e., whether raw, primary data leaves its original source) and participation of parties (i.e., whether one or multiple parties contribute to model development). Using these criteria, we define the categories as follows:

- **Local Learning**: Model development is carried out by a single entity using only its own data. No data sharing or coordination with external parties occurs.
- **Centralized Learning**: Multiple parties contribute to model development by sharing raw data with a central aggregator, where training is conducted.
- **Decentralized Learning**: Multiple parties participate in model development ***without exchanging raw data***. Instead, models, parameters, or privacy-preserving computations are shared to enable collaborative learning.

Within **decentralized learning**, we distinguish the following approaches:

- **Federated Learning**: A central server coordinates the training of local models on distributed data. Only model updates (e.g., weights or gradients) are shared; raw data remains local.
- **Swarm Learning**: A fully decentralized version of federated learning with **no central server**; model updates are aggregated peer-to-peer.
- **Ensemble Methods**: Independent models are trained locally by each party and later combined (e.g., via voting or stacking) without creating a unified global model or sharing data. These are considered decentralized as model combination occurs without raw data exchange.
- **Split or Transfer Learning**: Due to their similarity and reduced number of observations, we group split learning and transfer learning approaches, using the following definitions. In **split learning**, the model is partitioned into segments, with early layers trained locally and intermediate outputs (e.g., activations) passed to another party for further training. In **transfer learning**, a model trained by one party is fine-tuned or extended by another using local data. As long as only model components, intermediate representations, or parameters are exchanged—and no primary data is shared—these methods are considered decentralized under our operational framework.
- **Secure Multi-Party Computation (SMPC)**: Parties collaboratively compute a shared model using cryptographic protocols that ensure privacy of inputs. While SMPC is a privacy-enhancing technology rather than a learning paradigm per se, under our framework it qualifies as a decentralized learning approach when used to support joint model training without data exposure.

To resolve classification ambiguity—especially for hybrid or multi-stage training setups—we applied the following rule: If no raw (primary) data is shared between parties throughout the model development process, the approach is classified as decentralized, regardless of whether models, parameters, or representations are exchanged.

For the selection process, papers retrieved through the search strategy were evaluated by researchers acting independently and blinded for each other’s decisions. Each paper was classified by two researchers, with a total of seven reviewers. Whenever there was not a complete agreement on the decision, the researchers reviewed their decisions and discussed them to achieve a consensus. A third senior researcher was identified to resolve potential remaining conflicts. No automation tools were used during the selection process.

Due to a longer than expected initial article selection and the fast-moving research field, it was decided to update and apply the search strategy a second time, using the same methodology. The complete selection process is summarized using the PRISMA (Preferred Reporting Items for Systematic Reviews and Meta-Analyses) 2020 flow diagram in accordance with the corresponding guidelines^44^.

### Data collection process

Data were collected from the full-text version of the selected articles by two researchers, using a prepared online document piloted before its implementation. Researchers worked on different articles and discussed any doubts regarding the process to produce a harmonized data collection. The first author conducted a subsequent data collection verification looking for wrong, unclear, or missing records. Both researchers agreed by consensus on the version of the database reported. Data collection was organized in three ordered steps: general article information, models information and performance comparison information.

Regarding the model demands, we extracted reported data on multiple dimensions. First, for time-based metrics, we identified the following variables: training time, computation time, communication time, execution/inference/prediction time, encryption/decryption time, searching time and latency measurements. As far as resources consumption goes, the following metrics were covered: memory consumption (server and client memory), energy consumption and battery capacity requirements, power consumption and bandwidth consumption. Communication and data transfer variables considered were data upload volumes and total moved/transferred data. Specific privacy budget (ε-differential privacy parameters) and ξ, ζ-differential privacy impacts on performance metrics were collected.

### Effect measures

The primary effect measures were the performance metrics values of the decentralized learning models and their non-decentralized counterparts. These values were extracted directly from the included studies. To explore non-parametric effect sizes the Wilcoxon two-sample paired signed-rank test were used, comparing the distributions of the individual performance comparisons. Estimates of effect sizes and their respective magnitude are presented.

### Synthesis methods

Data collected were grouped by each performance metric and divided in the classes of the following variables: decentralized learning architecture, larger clinical domain and clinical application. Individual performance metrics with at least 30 comparisons collected were explored. An online dashboard was produced to allow for a customized search of relevant performance comparisons, using the Shiny R package – https://jmdiniz.shinyapps.io/phdiniz_systematic_review_analysis/.

The distribution of individual model performance differences between decentralized and non-decentralized alternatives across the difference performance metrics is presented using histograms and calculating their median difference, the 25th percentile and the 75th percentile, as well as the bootstrapped 95% Confidence Intervals, based on 10.000 simulations.

For sensitivity analysis, variations of these histograms are produced without the contributions of the study with the most observations – available in the Supplementary Material in Supplementary Figs. 43–56.

Specific detailed syntheses were produced for performance metrics-larger clinical domain-clinical application combinations, for instances with at least 10 comparisons and featuring at least 5 different studies.

Given the heterogeneity of clinical domains and applications, to assess clinically acceptable performance we set a threshold value of 0.80 (80%). Using this standard, we examined scenarios where local models failed to achieve clinical viability (<0.80) but DL achieved acceptable performance (≥0.80). Moreover, we identified the cases in which both local and decentralized models are clinically viable, but DL is superior, as well as instances in which local performance is clinically viable, but DL are not. The symmetric analysis was conducted considering centralized and decentralized models.

The only data processing concerned the conversion of values presented in percentages in some instances. Due to the heterogeneity in the data collected, no meta-analysis was conducted.

For each comparison and metric pairing, data were segmented into 10 equal-width intervals based on the range of the decentralized model performance. Within each segment, decentralized models were compared to their counterparts, based on the paired performance comparisons. In each facet, both the decile and the corresponding decentralized model performance range are showcased.

### Evidence appraisal

We applied the PROBAST + AI tool and the TRIPOD checklist for model type^45,46^ to the 25 most cited included research papers. For each paper, up to two models were considered, in order of presentation. Due to their inherent limitations, we opted to exclude TRIPOD Type 1a (i.e., all data used for model development without validation) and Type 1b articles (i.e., all data used for model development, evaluation using resampling). Using an approximation of the relative prevalence of the remaining TRIPOD types, 15 Type 2a articles, 5 Type 2b articles and 5 Type articles were included. Each article and its corresponding appraisals were conducted by a single reviewer.

### Registration and protocol

The research protocol for this study was published^32^, on June 6th, 2023. It was previously registered with PROSPERO, under the number 393126, on February 3^rd^, 2023, and accessible through https://www.crd.york.ac.uk/prospero/display_record.php?ID=CRD42023393126.

Details about the changes made to the protocol, and the rationale used, are presented in the Supplementary Material.

## Supplementary information

Supplementary file

Supplementary information

Supplementary information

Supplementary information

## Data Availability

A dashboard for select metrics is made available. Detailed data extracted from the included studies, including data used for analyses, the data processing and analytic code, is made available upon request. Moreover, documentation is provided regarding the specific queries used, tailored to each source, including their adapted formulation and filters, to ease reproducibility. Whenever possible, a direct URL link to the query is included.
