# Supplementary material for: Comparing decentralized machine learning and AI clinical models to local and centralized alternatives: a systematic review
Source: NPJ Digit Med. 2026 Feb 14;9:174. doi: 10.1038/s41746-025-02329-z (PMC12916833; doi:10.1038/s41746-025-02329-z)
Supplement: Supplementary file 1 — Supplementary file [file 41746_2025_2329_MOESM1_ESM.docx]

## Supplementary File

### Database Query

First, a simpler version of the query, suitable for all search engines, was used to retrieve a less specific group of abstracts.

For groups A, B, and C, the fields Title, Abstract, Keyword, and Field of Study, when available, will be searched. Terms from group A and B must be near each other, with a maximum of 2 words in between them. For terms in group D, the full-text document will be searched. The query will not be case-sensitive. The * symbol represents the wild card.

Thus, the search query will look for papers with at least 1 term, within the considered fields, from every group.

As per eligibility criteria, only primary papers from 2012 and beyond will be relevant. The queries used different time filters, whenever possible, according to the moment in which they were conducted.

#### Phase 1

##### DL ACM

**Query**: Validated

**Query Link:** Validated

Revised on 2023.04.04

**Extraction date:** 2023.04.06

**Branch #1: Abstract**

distributed OR federated OR decentrali* OR centrali* OR multi-party computation OR blockchain

**Branch #2: Abstract**

learn* OR model* OR train* OR tensor* OR perceptron OR algorithm* OR network* OR AI OR “artificial intelligence” OR ML OR “machine learning”

**Branch #3: Abstract**

health* OR medic* OR patient* OR clinic* OR physician* OR doctor*

**Branch #4: Full Text**

AUROC OR ROC OR “receiver operating characteristic curve” OR F1 OR “Jensen Shannon” OR Jensen-Shannon OR sensitivity OR recall OR specificity OR accuracy OR precision OR “predictive value” OR Dice OR conversion OR “performance”

**Date-limited:** 2021-2023

**Direct Link (Starting Point):**

Part 1 - Abstract-focused

<https://dl.acm.org/action/doSearch?fillQuickSearch=false&target=advanced&expand=dl&field1=Abstract&text1=distributed+OR+federated+OR+decentrali*+OR+centrali*+OR+%22multi-party+computation%22+OR+blockchain&field2=Abstract&text2=learn*+OR+model*+OR+train*+OR+tensor*+OR+perceptron+OR+algorithm*+OR+network*+OR+AI+OR+%22artificial+intelligence%22+OR+ML+OR+%22machine+learning%22&field3=Fulltext&text3=health*+OR+medic*+OR+patient*+OR+clinic*+OR+physician*+OR+doctor*&field4=Fulltext&text4=AUROC+OR+ROC+OR+%22receiver+operating+characteristic+curve%22+OR+F1+OR+%22Jensen+Shannon%22+OR+Jensen-Shannon+OR+sensitivity+OR+recall+OR+specificity+OR+accuracy+OR+precision+OR+%22predictive+value%22+OR+Dice+OR+conversion+OR+performance>

For Python:

[https://dl.acm.org/action/doSearch?fillQuickSearch=false&target=advanced&expand=dl&field1=Abstract&text1=distributed+OR+federated+OR+decentrali*+OR+centrali*+OR+\"multi-party+computation\"+OR+blockchain&field2=Abstract&text2=learn*+OR+model*+OR+train*+OR+tensor*+OR+perceptron+OR+algorithm*+OR+network*+OR+AI+OR+\"artificial+intelligence\"+OR+ML+OR+\"machine+learning\"&field3=Fulltext&text3=health*+OR+medic*+OR+patient*+OR+clinic*+OR+physician*+OR+doctor*&field4=Fulltext&text4=AUROC+OR+ROC+OR+\"receiver+operating+characteristic+curve\"+OR+F1+OR+\"Jensen+Shannon\"+OR+Jensen-Shannon+OR+sensitivity+OR+recall+OR+specificity+OR+accuracy+OR+precision+OR+\"predictive+value\"+OR+Dice+OR+conversion+OR+performance](https://dl.acm.org/action/doSearch?fillQuickSearch=false&target=advanced&expand=dl&field1=Abstract&text1=distributed+OR+federated+OR+decentrali*+OR+centrali*+OR+\%22multi-party+computation\%22+OR+blockchain&field2=Abstract&text2=learn*+OR+model*+OR+train*+OR+tensor*+OR+perceptron+OR+algorithm*+OR+network*+OR+AI+OR+\%22artificial+intelligence\%22+OR+ML+OR+\%22machine+learning\%22&field3=Fulltext&text3=health*+OR+medic*+OR+patient*+OR+clinic*+OR+physician*+OR+doctor*&field4=Fulltext&text4=AUROC+OR+ROC+OR+\%22receiver+operating+characteristic+curve\%22+OR+F1+OR+\%22Jensen+Shannon\%22+OR+Jensen-Shannon+OR+sensitivity+OR+recall+OR+specificity+OR+accuracy+OR+precision+OR+\%22predictive+value\%22+OR+Dice+OR+conversion+OR+performance)

Part 2 - Title-focused

[https://dl.acm.org/action/doSearch?fillQuickSearch=false&target=advanced&expand=dl&field1=Title&text1=distributed+OR+federated+OR+decentrali*+OR+centrali*+OR+"multi-party+computation"+OR+blockchain&field2=Title&text2=learn*+OR+model*+OR+train*+OR+tensor*+OR+perceptron+OR+algorithm*+OR+network*+OR+AI+OR+"artificial+intelligence"+OR+ML+OR+"machine+learning"&field3=Fulltext&text3=health*+OR+medic*+OR+patient*+OR+clinic*+OR+physician*+OR+doctor*&field4=Fulltext&text4=AUROC+OR+ROC+OR+"receiver+operating+characteristic+curve"+OR+F1+OR+"Jensen+Shannon"+OR+Jensen-Shannon+OR+sensitivity+OR+recall+OR+specificity+OR+accuracy+OR+precision+OR+"predictive+value"+OR+Dice+OR+conversion+OR+performance](https://dl.acm.org/action/doSearch?fillQuickSearch=false&target=advanced&expand=dl&field1=Title&text1=distributed+OR+federated+OR+decentrali*+OR+centrali*+OR+%22multi-party+computation%22+OR+blockchain&field2=Title&text2=learn*+OR+model*+OR+train*+OR+tensor*+OR+perceptron+OR+algorithm*+OR+network*+OR+AI+OR+%22artificial+intelligence%22+OR+ML+OR+%22machine+learning%22&field3=Fulltext&text3=health*+OR+medic*+OR+patient*+OR+clinic*+OR+physician*+OR+doctor*&field4=Fulltext&text4=AUROC+OR+ROC+OR+%22receiver+operating+characteristic+curve%22+OR+F1+OR+%22Jensen+Shannon%22+OR+Jensen-Shannon+OR+sensitivity+OR+recall+OR+specificity+OR+accuracy+OR+precision+OR+%22predictive+value%22+OR+Dice+OR+conversion+OR+performance)

For Python:

[https://dl.acm.org/action/doSearch?fillQuickSearch=false&target=advanced&expand=dl&field1=Title&text1=distributed+OR+federated+OR+decentrali*+OR+centrali*+OR+\"multi-party+computation\"+OR+blockchain&field2=Title&text2=learn*+OR+model*+OR+train*+OR+tensor*+OR+perceptron+OR+algorithm*+OR+network*+OR+AI+OR+\"artificial+intelligence\"+OR+ML+OR+\"machine+learning\"&field3=Fulltext&text3=health*+OR+medic*+OR+patient*+OR+clinic*+OR+physician*+OR+doctor*&field4=Fulltext&text4=AUROC+OR+ROC+OR+\"receiver+operating+characteristic+curve\"+OR+F1+OR+\"Jensen+Shannon\"+OR+Jensen-Shannon+OR+sensitivity+OR+recall+OR+specificity+OR+accuracy+OR+precision+OR+\"predictive+value\"+OR+Dice+OR+conversion+OR+performance](https://dl.acm.org/action/doSearch?fillQuickSearch=false&target=advanced&expand=dl&field1=Title&text1=distributed+OR+federated+OR+decentrali*+OR+centrali*+OR+\%22multi-party+computation\%22+OR+blockchain&field2=Title&text2=learn*+OR+model*+OR+train*+OR+tensor*+OR+perceptron+OR+algorithm*+OR+network*+OR+AI+OR+\%22artificial+intelligence\%22+OR+ML+OR+\%22machine+learning\%22&field3=Fulltext&text3=health*+OR+medic*+OR+patient*+OR+clinic*+OR+physician*+OR+doctor*&field4=Fulltext&text4=AUROC+OR+ROC+OR+\%22receiver+operating+characteristic+curve\%22+OR+F1+OR+\%22Jensen+Shannon\%22+OR+Jensen-Shannon+OR+sensitivity+OR+recall+OR+specificity+OR+accuracy+OR+precision+OR+\%22predictive+value\%22+OR+Dice+OR+conversion+OR+performance)

**Procedure:**

- For each results page, select all results, hitting the checkbox on the upper left corner of the list of results
- Select the option “Export Citations”
- Verify message: “Loading 250 citations…”
- Verify the option “BibTeX” is selected on the menu
- Click the blue arrow to download
- Change file name to “acm_export_01”
- Move to the next page and repeat the process, updating the file name according to the results page number

##### arXiv

**Query**: Validated

**Query Link:** Validated

Revised on 2023.04.04

**Extraction date:** 2023.04.06

Batches of 200 exportable articles (out of 2,687 results)

API: <https://info.arxiv.org/help/api/basics.html#using>

Query: order: submitted_date; size: 200; date_range: from 2012-01-01 to 2023-12-31; classification: Computer Science (cs), Electrical Engineering and Systems Science (eess), Quantitative Biology (q-bio); include_cross_list: True; terms: AND abstract=distributed OR federated OR decentral* OR central* OR “multi-party computation” OR blockchain; AND abstract=learn* OR model* OR train* OR tensor* OR perceptron OR algorithm* OR network* OR AI OR “artificial intelligence” OR ML OR “machine learning”; AND abstract=health* OR medic* OR patient* OR clinic* OR physician* OR doctor*

[https://arxiv.org/search/advanced?advanced=&terms-0-operator=AND&terms-0-term=distributed+OR+federated+OR+decentrali*+OR+centrali*+OR+"multi-party+computation"+OR+blockchain&terms-0-field=abstract&terms-1-operator=AND&terms-1-term=learn*+OR+model*+OR+train*+OR+tensor*+OR+perceptron+OR+algorithm*+OR+network*+OR+AI+OR+"artificial+intelligence"+OR+ML+OR+"machine+learning"&terms-1-field=abstract&terms-2-operator=AND&terms-2-term=health*+OR+medic*+OR+patient*+OR+clinic*+OR+physician*+OR+doctor*&terms-2-field=abstract&classification-computer_science=y&classification-eess=y&classification-physics_archives=all&classification-q_biology=y&classification-include_cross_list=include&date-year=&date-filter_by=date_range&date-from_date=2012&date-to_date=2023&date-date_type=submitted_date&abstracts=show&size=200&order=submitted_date](https://arxiv.org/search/advanced?advanced=&terms-0-operator=AND&terms-0-term=distributed+OR+federated+OR+decentrali*+OR+centrali*+OR+%22multi-party+computation%22+OR+blockchain&terms-0-field=abstract&terms-1-operator=AND&terms-1-term=learn*+OR+model*+OR+train*+OR+tensor*+OR+perceptron+OR+algorithm*+OR+network*+OR+AI+OR+%22artificial+intelligence%22+OR+ML+OR+%22machine+learning%22&terms-1-field=abstract&terms-2-operator=AND&terms-2-term=health*+OR+medic*+OR+patient*+OR+clinic*+OR+physician*+OR+doctor*&terms-2-field=abstract&classification-computer_science=y&classification-eess=y&classification-physics_archives=all&classification-q_biology=y&classification-include_cross_list=include&date-year=&date-filter_by=date_range&date-from_date=2012&date-to_date=2023&date-date_type=submitted_date&abstracts=show&size=200&order=submitted_date)

##### IEEE

**Query**: Validated

**Query Link:** Validated

Revised on 2023.04.04

**Extraction date:** 2023.04.06

**Notes:** There is a wildcard limited. Subscribed content allows for full-text access.

Hit the “Export” button, without additional selections. Exports up to 2.000 results

First, conduct a general export and then a filtered export (by “Subscribed Content”)

In R, separate the “Subscribed Content” and produce two dataframe

**Branch #1: Title or Abstract**

((“Document Title”: distributed OR federated OR decentralised OR decentralized OR centralised OR centralized OR multi-party computation OR blockchain) OR (“Abstract”: distributed OR federated OR decentralised OR decentralized OR centralised OR centralized OR multi-party computation OR blockchain))

- NEAR/2

**Branch #2: Title or Abstract**

((“Document Title”: learn OR learns OR learning OR learned OR learnt OR model OR models OR modeling OR modeled OR network OR networks OR networking OR perceptron OR perceptrons OR AI OR artificial intelligence OR ML OR machine learning OR train OR trains OR training OR trained OR algorithm OR algorithms OR tensor OR tensors) OR (“Abstract”: learn OR learns OR learning OR learned OR learnt OR model OR models OR modeling OR modeled OR network OR networks OR networking OR perceptron OR perceptrons OR AI OR artificial intelligence OR ML OR machine learning OR train OR trains OR training OR trained OR algorithm OR algorithms OR tensor OR tensors))

**Branch #3: Title or Abstract**

((“Document Title”: health OR healthy OR medical OR medicine OR medic OR clinical OR clinic OR clinician OR patient OR patients OR doctor OR doctors OR physician OR physicians) OR (“Abstract”: health OR healthy OR medical OR medicine OR medic OR clinical OR clinic OR clinician OR patient OR patients OR doctor OR doctors OR physician OR physicians))

- Select only “Conferences”, “Journals”, “Early Access Articles”, “Magazines”, “Standards”
- Filter 2012 onwards

<https://ieeexplore.ieee.org/search/searchresult.jsp?action=search&matchBoolean=true&queryText=>(((“Document Title”: distributed OR federated OR decentralised OR decentralized OR centralised OR centralized OR multi-party computation OR blockchain) OR (“Abstract”: distributed OR federated OR decentralised OR decentralized OR centralised OR centralized OR multi-party computation OR blockchain)) NEAR%2F2 ((“Document Title”: learn OR learns OR learning OR learned OR learnt OR model OR models OR modeling OR modeled OR network OR networks OR networking OR perceptron OR perceptrons OR AI OR artificial intelligence OR ML OR machine learning OR train OR trains OR training OR trained OR algorithm OR algorithms OR tensor OR tensors) OR (“Abstract”: learn OR learns OR learning OR learned OR learnt OR model OR models OR modeling OR modeled OR network OR networks OR networking OR perceptron OR perceptrons OR AI OR artificial intelligence OR ML OR machine learning OR train OR trains OR training OR trained OR algorithm OR algorithms OR tensor OR tensors)) AND ((“Document Title”: health OR healthy OR medical OR medicine OR medic OR clinical OR clinic OR clinician OR patient OR patients OR doctor OR doctors OR physician OR physicians) OR (“Abstract”: health OR healthy OR medical OR medicine OR medic OR clinical OR clinic OR clinician OR patient OR patients OR doctor OR doctors OR physician OR physicians))%0A)&highlight=true&returnType=SEARCH&matchPubs=true&returnFacets=ALL&ranges=2012_2023_Year&refinements=ContentType:Conferences&refinements=ContentType:Journals&refinements=ContentType:Early Access Articles&refinements=ContentType:Magazines&refinements=ContentType:Standards&rowsPerPage=100&pageNumber=1

Non-Subscribed Content = 30 – To be removed

- Publisher:
  - 13 = IEEE (only some)
  - **2 = KICKS (only some)**
    - Decentralized gene regulatory networks: An approach to energy efficient node coordination with delay constraints for wireless sensor systems
    - Special issue on big data networking-challenges and applications
  - **7 = MIT Press**
  - **1 = PTP**
  - **2 = OUP**
  - **5 = IET**

Subscribed Content = 1,787

**Branch #4: Full Text**

(“Full Text Only”: AUROC OR ROC OR receiver operating characteristic curve OR F1 OR Jensen Shannon OR Jensen-Shannon OR sensitivity OR recall OR specificity OR accuracy OR precision OR predictive value OR Dice OR conversion OR performance)

<https://ieeexplore.ieee.org/search/searchresult.jsp?action=search&matchBoolean=true&queryText=>(((%20((%22Document%20Title%22:%20distributed%20OR%20federated%20OR%20decentralised%20OR%20decentralized%20OR%20centralised%20OR%20centralized%20OR%20multi-party%20computation%20OR%20blockchain)%20OR%20(%22Abstract%22:%20distributed%20OR%20federated%20OR%20decentralised%20OR%20decentralized%20OR%20centralised%20OR%20centralized%20OR%20multi-party%20computation%20OR%20blockchain))%20NEAR%2F2%20((%22Document%20Title%22:%20learn%20OR%20learns%20OR%20learning%20OR%20learned%20OR%20learnt%20OR%20model%20OR%20models%20OR%20modeling%20OR%20modeled%20OR%20network%20OR%20networks%20OR%20networking%20OR%20perceptron%20OR%20perceptrons%20OR%20AI%20OR%20artificial%20intelligence%20OR%20ML%20OR%20machine%20learning%20OR%20train%20OR%20trains%20OR%20training%20OR%20trained)%20OR%20(%22Abstract%22:%20learn%20OR%20learns%20OR%20learning%20OR%20learned%20OR%20learnt%20OR%20model%20OR%20models%20OR%20modeling%20OR%20modeled%20OR%20network%20OR%20networks%20OR%20networking%20OR%20perceptron%20OR%20perceptrons%20OR%20AI%20OR%20artificial%20intelligence%20OR%20ML%20OR%20machine%20learning%20OR%20train%20OR%20trains%20OR%20training%20OR%20trained))%20)%20AND%20((%22Document%20Title%22:%20health%20OR%20healthy%20OR%20medical%20OR%20medicine%20OR%20medic%20OR%20clinical%20OR%20clinic%20OR%20clinician%20OR%20patient%20OR%20patients)%20OR%20(%22Abstract%22:%20health%20OR%20healthy%20OR%20medical%20OR%20medicine%20OR%20medic%20OR%20clinical%20OR%20clinic%20OR%20clinician%20OR%20patient%20OR%20patients))%20)%20AND%20(%22Full%20Text%20Only%22:%20AUROC%20OR%20ROC%20OR%20receiver%20operating%20characteristic%20curve%20OR%20F1%20OR%20Jensen%20Shannon%20OR%20Jensen-Shannon%20OR%20sensibility%20OR%20recall%20OR%20specificity%20OR%20accuracy%20OR%20precision%20OR%20predictive%20value%20OR%20Dice%20OR%20conversion%20OR%20perfomarnce))&highlight=true&returnType=SEARCH&matchPubs=true&returnFacets=ALL&ranges=2012_2023_Year&refinements=ContentType:Conferences&refinements=ContentType:Journals&refinements=ContentType:Early%20Access%20Articles&refinements=ContentType:Magazines&subscribed=true

##### Lens.org

**Query**: Validated

**Query Link:** Validated

Revised on 2023.04.04

**Extraction date:** 2023.04.06

**Branch #1: Title, Abstract, Keywords or Field of Study**

(title:(“distributed”) OR abstract:(“distributed”) OR keyword:(“distributed”) OR field_of_study:(“distributed”) OR title:(“federated”) OR abstract:(“federated”) OR keyword:(“federated”) OR field_of_study:(“federated”) OR title:(“decentrali*”) OR abstract:(”decentrali*”) OR keyword:(“decentrali*”) OR field_of_study:(”decentrali*”) OR title:(“centrali*”) OR abstract:(”centrali*”) OR keyword:(“centrali*”) OR field_of_study:(”centrali*”) OR title:(“blockchain”) OR abstract:(“blockchain”) OR keyword:(“blockchain”) OR field_of_study:(“blockchain”) OR title:(“multi-party computation”) OR abstract:(“multi-party computation”) OR keyword:(“multi-party computation”) OR field_of_study:(“multi-party computation”))

**Branch #2: Title, Abstract, Keywords or Field of Study**

(title:(learn*) OR abstract:(learn*) OR keyword:(learn*) OR field_of_study:(learn*) OR title:(model*) OR abstract:(model*) OR keyword:(model*) OR field_of_study:(model*) OR title:(network*) OR abstract:(network*) OR keyword:(network*) OR field_of_study:(network*) OR title:(perceptron*) OR abstract:(perceptron*) OR keyword:(perceptron*) OR field_of_study:(perceptron*) OR title:(algorithm*) OR abstract:(algorithm*) OR keyword:(algorithm*) OR field_of_study:(algorithm*) OR title:(AI) OR abstract:(AI) OR keyword:(AI) OR field_of_study:(AI) OR title:(artificial intelligence) OR abstract:(artificial intelligence) OR keyword:(artificial intelligence) OR field_of_study:(artificial intelligence) OR title:(ML) OR abstract:(ML) OR keyword:(ML) OR field_of_study:(ML) OR title:(machine learning) OR abstract:(machine learning) OR keyword:(machine learning) OR field_of_study:(machine learning) OR title:(train*) OR abstract:(train*) OR keyword:(train*) OR field_of_study:(train*))

**Branch #3: Title, Abstract, Keywords or Field of Study**

(title:(health*) OR abstract:(health*) OR keyword:(health*) OR field_of_study:(health*) OR title:(medic*) OR abstract:(medic*) OR keyword:(medic*) OR field_of_study:(medic*) OR title:(clinic*) OR abstract:(clinic*) OR keyword:(clinic*) OR field_of_study:(clinic*) OR title:(patient*) OR abstract:(patient*) OR keyword:(patient*) OR field_of_study:(patient*) OR title:(physician*) OR abstract:(physician*) OR keyword:(physician*) OR field_of_study:(physician*) OR title:(doctor*) OR abstract:(doctor*) OR keyword:(doctor*) OR field_of_study:(doctor*))

**Date-limited:** 2021-2023

Exclusion of “Book”, “Book chapter”, “Letter”, “Editorial”, “News”, “Review”, “Dataset”, “Component”

**No Full Text** (Segmentation without further searches) Excluding “Has Full Text”

<https://www.lens.org/lens/search/scholar/list?q=(title>:(%22distributed%22)%20OR%20abstract:(%22distributed%22)%20OR%20keyword:(%22distributed%22)%20OR%20field_of_study:(%22distributed%22)%20OR%20title:(%22federated%22)%20OR%20abstract:(%22federated%22)%20OR%20keyword:(%22federated%22)%20OR%20field_of_study:(%22federated%22)%20OR%20title:(%22decentrali*%22)%20OR%20abstract:(%22decentrali*%22)%20OR%20keyword:(%22decentrali*%22)%20OR%20field_of_study:(%22decentrali*%22)%20OR%20title:(%22centrali*%22)%20OR%20abstract:(%22centrali*%22)%20OR%20keyword:(%22centrali*%22)%20OR%20field_of_study:(%22centrali*%22)%20OR%20title:(%22blockchain%22)%20OR%20abstract:(%22blockchain%22)%20OR%20keyword:(%22blockchain%22)%20OR%20field_of_study:(%22blockchain%22)%20OR%20title:(%22multi-party%20computation%22)%20OR%20abstract:(%22multi-party%20computation%22)%20OR%20keyword:(%22multi-party%20computation%22)%20OR%20field_of_study:(%22multi-party%20computation%22))%20AND%20(title:(learn*)%20OR%20abstract:(learn*)%20OR%20keyword:(learn*)%20OR%20field_of_study:(learn*)%20OR%20title:(model*)%20OR%20abstract:(model*)%20OR%20keyword:(model*)%20OR%20field_of_study:(model*)%20OR%20title:(network*)%20OR%20abstract:(network*)%20OR%20keyword:(network*)%20OR%20field_of_study:(network*)%20OR%20title:(perceptron*)%20OR%20abstract:(perceptron*)%20OR%20keyword:(perceptron*)%20OR%20field_of_study:(perceptron*)%20OR%20title:(algorithm*)%20OR%20abstract:(algorithm*)%20OR%20keyword:(algorithm*)%20OR%20field_of_study:(algorithm*)%20OR%20title:(AI)%20OR%20abstract:(AI)%20OR%20keyword:(AI)%20OR%20field_of_study:(AI)%20OR%20title:(artificial%20intelligence)%20OR%20abstract:(artificial%20intelligence)%20OR%20keyword:(artificial%20intelligence)%20OR%20field_of_study:(artificial%20intelligence)%20OR%20title:(ML)%20OR%20abstract:(ML)%20OR%20keyword:(ML)%20OR%20field_of_study:(ML)%20OR%20title:(machine%20learning)%20OR%20abstract:(machine%20learning)%20OR%20keyword:(machine%20learning)%20OR%20field_of_study:(machine%20learning)%20OR%20title:(train*)%20OR%20abstract:(train*)%20OR%20keyword:(train*)%20OR%20field_of_study:(train*))%20AND%20(title:(health*)%20OR%20abstract:(health*)%20OR%20keyword:(health*)%20OR%20field_of_study:(health*)%20OR%20title:(medic*)%20OR%20abstract:(medic*)%20OR%20keyword:(medic*)%20OR%20field_of_study:(medic*)%20OR%20title:(clinic*)%20OR%20abstract:(clinic*)%20OR%20keyword:(clinic*)%20OR%20field_of_study:(clinic*)%20OR%20title:(patient*)%20OR%20abstract:(patient*)%20OR%20keyword:(patient*)%20OR%20field_of_study:(patient*)%20OR%20title:(physician*)%20OR%20abstract:(physician*)%20OR%20keyword:(physician*)%20OR%20field_of_study:(physician*)%20OR%20title:(doctor*)%20OR%20abstract:(doctor*)%20OR%20keyword:(doctor*)%20OR%20field_of_study:(doctor*))&p=0&n=10&s=_score&d=%2B&f=false&e=false&l=en&authorField=author&dateFilterField=publishedYear&orderBy=%2B_score&presentation=false&preview=true&stemmed=true&useAuthorId=false&publicationType.must=journal%20article&publicationType.must=unknown&publicationType.must=conference%20proceedings%20article&publicationType.must=dissertation&publicationType.must=preprint&publicationType.must=conference%20proceedings&publicationType.must=other&publicationType.must=report&publicationType.must=clinical%20trial&publicationType.must=reference%20entry&publicationType.must=journal%20issue&publicationType.must=journal&publicationType.must=journal%20volume&publicationType.must=libguide&publicationType.must=clinical%20study&publicationType.must=standard&publicationType.mustNot=component&publicationType.mustNot=dataset&publicationType.mustNot=book%20chapter&publicationType.mustNot=book&publicationType.mustNot=editorial&publicationType.mustNot=letter&publicationType.mustNot=review&publicationType.mustNot=news&publishedYear.from=2012&publishedYear.to=2024&hasFullText=false

**Yield: 47,983**

**Branch #4: Full Text**

(fulltext:AUROC OR ROC OR “receiver operating characteristic curve” OR F1 OR “Jensen Shannon” OR Jensen-Shannon OR sensitivity OR recall OR specificity OR accuracy OR precision OR “predictive value” OR Dice OR conversion OR performance)

<https://www.lens.org/lens/search/scholar/list?q=(title>:(%22distributed%22)%20OR%20abstract:(%22distributed%22)%20OR%20keyword:(%22distributed%22)%20OR%20field_of_study:(%22distributed%22)%20OR%20title:(%22federated%22)%20OR%20abstract:(%22federated%22)%20OR%20keyword:(%22federated%22)%20OR%20field_of_study:(%22federated%22)%20OR%20title:(%22decentrali*%22)%20OR%20abstract:(%22decentrali*%22)%20OR%20keyword:(%22decentrali*%22)%20OR%20field_of_study:(%22decentrali*%22)%20OR%20title:(%22centrali*%22)%20OR%20abstract:(%22centrali*%22)%20OR%20keyword:(%22centrali*%22)%20OR%20field_of_study:(%22centrali*%22)%20OR%20title:(%22blockchain%22)%20OR%20abstract:(%22blockchain%22)%20OR%20keyword:(%22blockchain%22)%20OR%20field_of_study:(%22blockchain%22)%20OR%20title:(%22multi-party%20computation%22)%20OR%20abstract:(%22multi-party%20computation%22)%20OR%20keyword:(%22multi-party%20computation%22)%20OR%20field_of_study:(%22multi-party%20computation%22))%20AND%20(title:(learn*)%20OR%20abstract:(learn*)%20OR%20keyword:(learn*)%20OR%20field_of_study:(learn*)%20OR%20title:(model*)%20OR%20abstract:(model*)%20OR%20keyword:(model*)%20OR%20field_of_study:(model*)%20OR%20title:(network*)%20OR%20abstract:(network*)%20OR%20keyword:(network*)%20OR%20field_of_study:(network*)%20OR%20title:(perceptron*)%20OR%20abstract:(perceptron*)%20OR%20keyword:(perceptron*)%20OR%20field_of_study:(perceptron*)%20OR%20title:(algorithm*)%20OR%20abstract:(algorithm*)%20OR%20keyword:(algorithm*)%20OR%20field_of_study:(algorithm*)%20OR%20title:(AI)%20OR%20abstract:(AI)%20OR%20keyword:(AI)%20OR%20field_of_study:(AI)%20OR%20title:(artificial%20intelligence)%20OR%20abstract:(artificial%20intelligence)%20OR%20keyword:(artificial%20intelligence)%20OR%20field_of_study:(artificial%20intelligence)%20OR%20title:(ML)%20OR%20abstract:(ML)%20OR%20keyword:(ML)%20OR%20field_of_study:(ML)%20OR%20title:(machine%20learning)%20OR%20abstract:(machine%20learning)%20OR%20keyword:(machine%20learning)%20OR%20field_of_study:(machine%20learning)%20OR%20title:(train*)%20OR%20abstract:(train*)%20OR%20keyword:(train*)%20OR%20field_of_study:(train*))%20AND%20(title:(health*)%20OR%20abstract:(health*)%20OR%20keyword:(health*)%20OR%20field_of_study:(health*)%20OR%20title:(medic*)%20OR%20abstract:(medic*)%20OR%20keyword:(medic*)%20OR%20field_of_study:(medic*)%20OR%20title:(clinic*)%20OR%20abstract:(clinic*)%20OR%20keyword:(clinic*)%20OR%20field_of_study:(clinic*)%20OR%20title:(patient*)%20OR%20abstract:(patient*)%20OR%20keyword:(patient*)%20OR%20field_of_study:(patient*)%20OR%20title:(physician*)%20OR%20abstract:(physician*)%20OR%20keyword:(physician*)%20OR%20field_of_study:(physician*)%20OR%20title:(doctor*)%20OR%20abstract:(doctor*)%20OR%20keyword:(doctor*)%20OR%20field_of_study:(doctor*))%20AND%20(fulltext:AUROC%20OR%20ROC%20OR%20%22receiver%20operating%20characteristic%20curve%22%20OR%20F1%20OR%20%22Jensen%20Shannon%22%20OR%20Jensen-Shannon%20OR%20sensitivity%20OR%20recall%20OR%20specificity%20OR%20accuracy%20OR%20precision%20OR%20%22predictive%20value%22%20OR%20Dice%20OR%20conversion%20OR%20performance)&p=0&n=10&s=_score&d=%2B&f=false&e=false&l=en&authorField=author&dateFilterField=publishedYear&orderBy=%2B_score&presentation=false&preview=true&stemmed=true&useAuthorId=false&publicationType.must=journal%20article&publicationType.must=unknown&publicationType.must=conference%20proceedings%20article&publicationType.must=preprint&publicationType.must=dissertation&publicationType.must=conference%20proceedings&publicationType.must=other&publicationType.must=report&publicationType.must=clinical%20trial&publicationType.must=journal%20issue&publicationType.must=reference%20entry&publicationType.must=journal%20volume&publicationType.must=journal&publicationType.must=standard&publicationType.mustNot=book%20chapter&publicationType.mustNot=book&publicationType.mustNot=component&publicationType.mustNot=dataset&publicationType.mustNot=letter&publicationType.mustNot=editorial&publicationType.mustNot=news&publicationType.mustNot=review&publishedYear.from=2012&publishedYear.to=2024&hasFullText=true

Yield: 1,783

##### LWW

**Query**: Validated

**Query Link:** Validated

Revised on 2023.04.04

**Extraction date:** 2023.04.06

Exportable to EndNote -> May need conversion: <https://www.bibtex.com/c/endnote-to-bibtex-converter/>

<https://lww.com/pages/results.aspx?txtKeywords=(health+OR+medical+OR+patient+OR+clinical+OR+clinic+OR+physician+OR+doctor)+AND+(distributed+OR+federated+OR+decentralized+OR+decentralised+OR+centralized+OR+centralised+OR+multi-party+computation+OR+blockchain)+AND+(learning+OR+model+OR+training+OR+tensor+OR+perceptron+OR+algorithm+OR+network+OR+AI+OR+artificial+intelligence+OR+ML+OR+machine+learning)>

##### medRxiv

**Query**: Validated

**Query Link:** Validated

Revised on 2023.04.04

**Extraction date:** 2023.04.06

**Direct Link:**

<https://www.medrxiv.org/search/%20abstract_title%3Afederated%252C%2Bdistributed%252C%2Bdecentrali%252A%252C%2Bmulti-party%2Bcomputation%252C%2Bmulti%2Bparty%2Bcomputation%252C%2Bcentrali%252A%20abstract_title_flags%3Amatch-any%20jcode%3Amedrxiv%20numresults%3A450%20sort%3Arelevance-rank%20format_result%3Astandard>

**Procedure:**

- Select “Add All Citations”
- Repeat for all other pages
- Go to “View Selected Citations”
- Export all results in a EndNote 8 format (xml)

##### Scopus

**Query**: Validated

**Query Link:** Validated

Revised on 2023.04.04

**Note:** Some extra exclusion keywords were added

**Extraction date:** 2023.04.06

**Branch #1: Title or Abstract**

(TITLE-ABS(distributed OR federated OR decentrali* OR (multi-party computation) OR (multi party computation) OR centrali* OR blockchain )) AND (TITLE-ABS(learn* OR model* OR train* OR tensor* OR perceptron OR algorithm* OR network* OR AI OR (artificial intelligence) OR ML OR (machine learning))) AND (TITLE-ABS(health* OR medic* OR patient* OR clinic* OR physician* OR doctor*))

**Date Limit:** 2012-2024

Limit to: Article, Conference paper, Undefined

Exclude Keywords: Nonhuman, Animal, Animal Experiment, Animal Model, Mouse, Animal Tissue, Mice, Systematic Review, Rat, Animal Cell, “Disease Models, Animal”, Questionnaires, Rats, Animals, “Surveys and Questionnaires”,

Select “Try new version”

( TITLE-ABS ( distributed OR federated OR decentrali* OR ( multi-party AND computation ) OR ( multi AND party AND computation ) OR centrali* OR blockchain ) ) AND ( TITLE-ABS ( learn* OR model* OR train* OR tensor* OR perceptron OR algorithm* OR network* OR ai OR ( artificial AND intelligence ) OR ml OR ( machine AND learning ) ) ) AND ( TITLE-ABS ( health* OR medic* OR patient* OR clinic* OR physician* OR doctor* ) ) AND PUBYEAR > 2011 AND PUBYEAR < 2025 AND ( LIMIT-TO ( DOCTYPE , “ar” ) OR LIMIT-TO ( DOCTYPE , “cp” ) OR LIMIT-TO ( DOCTYPE , “Undefined” ) ) AND ( EXCLUDE ( EXACTKEYWORD , “Nonhuman” ) OR EXCLUDE ( EXACTKEYWORD , “Animals” ) OR EXCLUDE ( EXACTKEYWORD , “Animal” ) OR EXCLUDE ( EXACTKEYWORD , “Animal Experiment” ) OR EXCLUDE ( EXACTKEYWORD , “Animal Model” ) OR EXCLUDE ( EXACTKEYWORD , “Mouse” ) OR EXCLUDE ( EXACTKEYWORD , “Animal Tissue” ) OR EXCLUDE ( EXACTKEYWORD , “Mice” ) OR EXCLUDE ( EXACTKEYWORD , “Surveys And Questionnaires” ) OR EXCLUDE ( EXACTKEYWORD , “Systematic Review” ) OR EXCLUDE ( EXACTKEYWORD , “Rat” ) OR EXCLUDE ( EXACTKEYWORD , “Animal Cell” ) OR EXCLUDE ( EXACTKEYWORD , “Disease Models, Animal” ) OR EXCLUDE ( EXACTKEYWORD , “Questionnaires” ) OR EXCLUDE ( EXACTKEYWORD , “Rats” ) )

**Direct Link:**

<https://www.scopus.com/results/results.uri?sort=plf-f&src=s&sid=a4791c5e81f52e27d54c4db01796a7b4&sot=a&sdt=cl&cluster=scosubtype%2C%22ar%22%2Ct%2C%22cp%22%2Ct%2C%22Undefined%22%2Ct%2Bscoexactkeywords%2C%22Nonhuman%22%2Cf%2C%22Animals%22%2Cf%2C%22Animal%22%2Cf%2C%22Animal+Experiment%22%2Cf%2C%22Animal+Model%22%2Cf%2C%22Mouse%22%2Cf%2C%22Animal+Tissue%22%2Cf%2C%22Mice%22%2Cf%2C%22Surveys+And+Questionnaires%22%2Cf%2C%22Systematic+Review%22%2Cf%2C%22Rat%22%2Cf%2C%22Animal+Cell%22%2Cf%2C%22Disease+Models%2C+Animal%22%2Cf%2C%22Questionnaires%22%2Cf%2C%22Rats%22%2Cf&sessionSearchId=a4791c5e81f52e27d54c4db01796a7b4&origin=resultslist&editSaveSearch=&txGid=104d59dfe9b448b30fbe2d5767c369b3&featureToggles=FEATURE_DOCUMENT_RESULT_MICRO_UI%3A1&limit=10&s=%28+TITLE-ABS+%28+distributed+OR+federated+OR+decentrali*+OR+%28+multi-party+AND+computation+%29+OR+%28+multi+AND+party+AND+computation+%29+OR+centrali*+OR+blockchain+%29+%29+AND+%28+TITLE-ABS+%28+learn*+OR+model*+OR+train*+OR+tensor*+OR+perceptron+OR+algorithm*+OR+network*+OR+ai+OR+%28+artificial+AND+intelligence+%29+OR+ml+OR+%28+machine+AND+learning+%29+%29+%29+AND+%28+TITLE-ABS+%28+health*+OR+medic*+OR+patient*+OR+clinic*+OR+physician*+OR+doctor*+%29+%29+AND+PUBYEAR+%3E+2011+AND+PUBYEAR+%3C+2025&yearFrom=2012&yearTo=2023>

##### Springer Nature

**Query**: Validated

**Query Link:** Validated

Revised on 2023.03.24

**Extraction date:** 2023.04.06

<https://forums.zotero.org/discussion/28442/mass-import-of-references-from-springer-link>

- Individual extraction of all search results
- Loop using SpringerLink API to extract meta-data
- Create .csv file to import to Rayyan

Example: <http://api.springernature.com/meta/v2/json?q=doi:10.1007/s10462-023-10417-3&api_key=59d958331507afaa2644390c0ea486a6>

(distributed OR federated OR decentrali* OR centrali* OR “multi-party computation” OR blockchain) AND (learn* OR model* OR train* OR tensor* OR perceptron OR algorithm* OR network* OR AI OR “artificial intelligence” OR ML OR “machine learning”) AND (health* OR medic* OR patient* OR clinc* OR physician* OR doctor*)

Add time limit (2012-2024) and other filters (Article, Conference Paper, Reference Work Entry, Conference Proceedings, Reference Work)

###### Distributed

<https://rd.springer.com/search?query=%28distributed+NEAR%2F2+%28learn*+model*+OR+train*+OR+tensor*+OR+perceptron+OR+algorithm*+OR+network*+OR+AI+OR+%22artificial+intelligence%22+OR+ML+OR+%22machine+learning%22%29%29+AND+%28health*+OR+medic*+OR+patient*+OR+clinc*+OR+physician*+OR+doctor*%29&facet-end-year=2024&showAll=true&date-facet-mode=between&facet-start-year=2012>

###### Federated

<https://rd.springer.com/search?query=%28federated+NEAR%2F2+%28learn*+model*+OR+train*+OR+tensor*+OR+perceptron+OR+algorithm*+OR+network*+OR+AI+OR+%22artificial+intelligence%22+OR+ML+OR+%22machine+learning%22%29%29+AND+%28health*+OR+medic*+OR+patient*+OR+clinc*+OR+physician*+OR+doctor*%29>

###### Blockchain

<https://rd.springer.com/search?query=%28blockchain+NEAR%2F2+%28learn*+model*+OR+train*+OR+tensor*+OR+perceptron+OR+algorithm*+OR+network*+OR+AI+OR+%22artificial+intelligence%22+OR+ML+OR+%22machine+learning%22%29%29+AND+%28health*+OR+medic*+OR+patient*+OR+clinc*+OR+physician*+OR+doctor*%29>

###### Decentrali*

<https://rd.springer.com/search?query=%28decentrali*+NEAR%2F2+%28learn*+model*+OR+train*+OR+tensor*+OR+perceptron+OR+algorithm*+OR+network*+OR+AI+OR+%22artificial+intelligence%22+OR+ML+OR+%22machine+learning%22%29%29+AND+%28health*+OR+medic*+OR+patient*+OR+clinc*+OR+physician*+OR+doctor*%29>

###### Centrali*

<https://rd.springer.com/search?query=%28centrali*+NEAR%2F2+%28learn*+model*+OR+train*+OR+tensor*+OR+perceptron+OR+algorithm*+OR+network*+OR+AI+OR+%22artificial+intelligence%22+OR+ML+OR+%22machine+learning%22%29%29+AND+%28health*+OR+medic*+OR+patient*+OR+clinc*+OR+physician*+OR+doctor*%29>

###### MPC

<https://rd.springer.com/search?query=%28%22multi-party+computation%22+NEAR%2F2+%28learn*+model*+OR+train*+OR+tensor*+OR+perceptron+OR+algorithm*+OR+network*+OR+AI+OR+%22artificial+intelligence%22+OR+ML+OR+%22machine+learning%22%29%29+AND+%28health*+OR+medic*+OR+patient*+OR+clinc*+OR+physician*+OR+doctor*%29>

##### Web of Science

**Query**: Validated

**Query Link:** Validated

Revised on 2023.04.04

**Extraction date:** 2023.04.06

Batch export of 1.000 results

**Branch #1 & #2 (NEAR/2): Title or Abstract**

(TI=((distributed NEAR/2 learn*) OR (distributed NEAR/2 model*) OR (distributed NEAR/2 train*) OR (distributed NEAR/2 tensor*) OR (distributed NEAR/2 perceptron) OR (distributed NEAR/2 algorithm*) OR (distributed NEAR/2 network*) OR (distributed NEAR/2 AI) OR (distributed NEAR/2 “artificial intelligence”) OR (distributed NEAR/2 ML) OR (distributed NEAR/2 “machine learning”) OR (federated NEAR/2 learn*) OR (federated NEAR/2 model*) OR (federated NEAR/2 train*) OR (federated NEAR/2 tensor*) OR (federated NEAR/2 perceptron) OR (federated NEAR/2 algorithm*) OR (federated NEAR/2 network*) OR (federated NEAR/2 AI) OR (federated NEAR/2 “artificial intelligence”) OR (federated NEAR/2 ML) OR (federated NEAR/2 “machine learning”) OR (decentrali* NEAR/2 learn*) OR (decentrali* NEAR/2 model*) OR (decentrali* NEAR/2 train*) OR (decentrali* NEAR/2 tensor*) OR (decentrali* NEAR/2 perceptron) OR (decentrali* NEAR/2 algorithm*) OR (decentrali* NEAR/2 network*) OR (decentrali* NEAR/2 AI) OR (decentrali* NEAR/2 “artificial intelligence”) OR (decentrali* NEAR/2 ML) OR (decentrali* NEAR/2 “machine learning”) OR (centrali* NEAR/2 learn*) OR (centrali* NEAR/2 model*) OR (centrali* NEAR/2 train*) OR (centrali* NEAR/2 tensor*) OR (centrali* NEAR/2 perceptron) OR (centrali* NEAR/2 algorithm*) OR (centrali* NEAR/2 network*) OR (centrali* NEAR/2 AI) OR (centrali* NEAR/2 “artificial intelligence”) OR (centrali* NEAR/2 ML) OR (centrali* NEAR/2 “machine learning”) OR (“multi-party computation” NEAR/2 learn*) OR (“multi-party computation” NEAR/2 model*) OR (“multi-party computation” NEAR/2 train*) OR (“multi-party computation” NEAR/2 tensor*) OR (“multi-party computation” NEAR/2 perceptron) OR (“multi-party computation” NEAR/2 algorithm*) OR (“multi-party computation” NEAR/2 network*) OR (“multi-party computation” NEAR/2 AI) OR (“multi-party computation” NEAR/2 “artificial intelligence”) OR (“multi-party computation” NEAR/2 ML) OR (“multi-party computation” NEAR/2 “machine learning”) OR (blockchain NEAR/2 learn*) OR (blockchain NEAR/2 model*) OR (blockchain NEAR/2 train*) OR (blockchain NEAR/2 tensor*) OR (blockchain NEAR/2 perceptron) OR (blockchain NEAR/2 algorithm*) OR (blockchain NEAR/2 network*) OR (blockchain NEAR/2 AI) OR (blockchain NEAR/2 “artificial intelligence”) OR (blockchain NEAR/2 ML) OR (blockchain NEAR/2 “machine learning”))) OR (AB=((distributed NEAR/2 learn*) OR (distributed NEAR/2 model*) OR (distributed NEAR/2 train*) OR (distributed NEAR/2 tensor*) OR (distributed NEAR/2 perceptron) OR (distributed NEAR/2 algorithm*) OR (distributed NEAR/2 network*) OR (distributed NEAR/2 AI) OR (distributed NEAR/2 “artificial intelligence”) OR (distributed NEAR/2 ML) OR (distributed NEAR/2 “machine learning”) OR (federated NEAR/2 learn*) OR (federated NEAR/2 model*) OR (federated NEAR/2 train*) OR (federated NEAR/2 tensor*) OR (federated NEAR/2 perceptron) OR (federated NEAR/2 algorithm*) OR (federated NEAR/2 network*) OR (federated NEAR/2 AI) OR (federated NEAR/2 “artificial intelligence”) OR (federated NEAR/2 ML) OR (federated NEAR/2 “machine learning”) OR (decentrali* NEAR/2 learn*) OR (decentrali* NEAR/2 model*) OR (decentrali* NEAR/2 train*) OR (decentrali* NEAR/2 tensor*) OR (decentrali* NEAR/2 perceptron) OR (decentrali* NEAR/2 algorithm*) OR (decentrali* NEAR/2 network*) OR (decentrali* NEAR/2 AI) OR (decentrali* NEAR/2 “artificial intelligence”) OR (decentrali* NEAR/2 ML) OR (decentrali* NEAR/2 “machine learning”) OR (centrali* NEAR/2 learn*) OR (centrali* NEAR/2 model*) OR (centrali* NEAR/2 train*) OR (centrali* NEAR/2 tensor*) OR (centrali* NEAR/2 perceptron) OR (centrali* NEAR/2 algorithm*) OR (centrali* NEAR/2 network*) OR (centrali* NEAR/2 AI) OR (centrali* NEAR/2 “artificial intelligence”) OR (centrali* NEAR/2 ML) OR (centrali* NEAR/2 “machine learning”) OR (“multi-party computation” NEAR/2 learn*) OR (“multi-party computation” NEAR/2 model*) OR (“multi-party computation” NEAR/2 train*) OR (“multi-party computation” NEAR/2 tensor*) OR (“multi-party computation” NEAR/2 perceptron) OR (“multi-party computation” NEAR/2 algorithm*) OR (“multi-party computation” NEAR/2 network*) OR (“multi-party computation” NEAR/2 AI) OR (“multi-party computation” NEAR/2 “artificial intelligence”) OR (“multi-party computation” NEAR/2 ML) OR (“multi-party computation” NEAR/2 “machine learning”) OR (blockchain NEAR/2 learn*) OR (blockchain NEAR/2 model*) OR (blockchain NEAR/2 train*) OR (blockchain NEAR/2 tensor*) OR (blockchain NEAR/2 perceptron) OR (blockchain NEAR/2 algorithm*) OR (blockchain NEAR/2 network*) OR (blockchain NEAR/2 AI) OR (blockchain NEAR/2 “artificial intelligence”) OR (blockchain NEAR/2 ML) OR (blockchain NEAR/2 “machine learning”)))

**Branch #3: Title or Abstract**

((TI=(health* OR medic* OR patient* OR clinic* OR physician* OR doctor*)) OR (AB=(health* OR medic* OR patient* OR clinic* OR physician* OR doctor*)))

**Date-limited:** 2012-beyond

**Document Types:** “Article”, “Proceeding Paper”, “Early Access”, “Meeting Abstract”, “Correction”

Yield: 5,692

**Query:**

<https://www.webofscience.com/wos/woscc/summary/bb0cacc8-00c4-4db2-bc1d-f5a7cd487fce-7f9e6f19/date-ascending/1>

((TI=((distributed NEAR/2 learn*) OR (distributed NEAR/2 model*) OR (distributed NEAR/2 train*) OR (distributed NEAR/2 tensor*) OR (distributed NEAR/2 perceptron) OR (distributed NEAR/2 algorithm*) OR (distributed NEAR/2 network*) OR (distributed NEAR/2 AI) OR (distributed NEAR/2 “artificial intelligence”) OR (distributed NEAR/2 ML) OR (distributed NEAR/2 “machine learning”) OR (federated NEAR/2 learn*) OR (federated NEAR/2 model*) OR (federated NEAR/2 train*) OR (federated NEAR/2 tensor*) OR (federated NEAR/2 perceptron) OR (federated NEAR/2 algorithm*) OR (federated NEAR/2 network*) OR (federated NEAR/2 AI) OR (federated NEAR/2 “artificial intelligence”) OR (federated NEAR/2 ML) OR (federated NEAR/2 “machine learning”) OR (decentrali* NEAR/2 learn*) OR (decentrali* NEAR/2 model*) OR (decentrali* NEAR/2 train*) OR (decentrali* NEAR/2 tensor*) OR (decentrali* NEAR/2 perceptron) OR (decentrali* NEAR/2 algorithm*) OR (decentrali* NEAR/2 network*) OR (decentrali* NEAR/2 AI) OR (decentrali* NEAR/2 “artificial intelligence”) OR (decentrali* NEAR/2 ML) OR (decentrali* NEAR/2 “machine learning”) OR (centrali* NEAR/2 learn*) OR (centrali* NEAR/2 model*) OR (centrali* NEAR/2 train*) OR (centrali* NEAR/2 tensor*) OR (centrali* NEAR/2 perceptron) OR (centrali* NEAR/2 algorithm*) OR (centrali* NEAR/2 network*) OR (centrali* NEAR/2 AI) OR (centrali* NEAR/2 “artificial intelligence”) OR (centrali* NEAR/2 ML) OR (centrali* NEAR/2 “machine learning”) OR (“multi-party computation” NEAR/2 learn*) OR (“multi-party computation” NEAR/2 model*) OR (“multi-party computation” NEAR/2 train*) OR (“multi-party computation” NEAR/2 tensor*) OR (“multi-party computation” NEAR/2 perceptron) OR (“multi-party computation” NEAR/2 algorithm*) OR (“multi-party computation” NEAR/2 network*) OR (“multi-party computation” NEAR/2 AI) OR (“multi-party computation” NEAR/2 “artificial intelligence”) OR (“multi-party computation” NEAR/2 ML) OR (“multi-party computation” NEAR/2 “machine learning”) OR (blockchain NEAR/2 learn*) OR (blockchain NEAR/2 model*) OR (blockchain NEAR/2 train*) OR (blockchain NEAR/2 tensor*) OR (blockchain NEAR/2 perceptron) OR (blockchain NEAR/2 algorithm*) OR (blockchain NEAR/2 network*) OR (blockchain NEAR/2 AI) OR (blockchain NEAR/2 “artificial intelligence”) OR (blockchain NEAR/2 ML) OR (blockchain NEAR/2 “machine learning”))) OR (AB=((distributed NEAR/2 learn*) OR (distributed NEAR/2 model*) OR (distributed NEAR/2 train*) OR (distributed NEAR/2 tensor*) OR (distributed NEAR/2 perceptron) OR (distributed NEAR/2 algorithm*) OR (distributed NEAR/2 network*) OR (distributed NEAR/2 AI) OR (distributed NEAR/2 “artificial intelligence”) OR (distributed NEAR/2 ML) OR (distributed NEAR/2 “machine learning”) OR (federated NEAR/2 learn*) OR (federated NEAR/2 model*) OR (federated NEAR/2 train*) OR (federated NEAR/2 tensor*) OR (federated NEAR/2 perceptron) OR (federated NEAR/2 algorithm*) OR (federated NEAR/2 network*) OR (federated NEAR/2 AI) OR (federated NEAR/2 “artificial intelligence”) OR (federated NEAR/2 ML) OR (federated NEAR/2 “machine learning”) OR (decentrali* NEAR/2 learn*) OR (decentrali* NEAR/2 model*) OR (decentrali* NEAR/2 train*) OR (decentrali* NEAR/2 tensor*) OR (decentrali* NEAR/2 perceptron) OR (decentrali* NEAR/2 algorithm*) OR (decentrali* NEAR/2 network*) OR (decentrali* NEAR/2 AI) OR (decentrali* NEAR/2 “artificial intelligence”) OR (decentrali* NEAR/2 ML) OR (decentrali* NEAR/2 “machine learning”) OR (centrali* NEAR/2 learn*) OR (centrali* NEAR/2 model*) OR (centrali* NEAR/2 train*) OR (centrali* NEAR/2 tensor*) OR (centrali* NEAR/2 perceptron) OR (centrali* NEAR/2 algorithm*) OR (centrali* NEAR/2 network*) OR (centrali* NEAR/2 AI) OR (centrali* NEAR/2 “artificial intelligence”) OR (centrali* NEAR/2 ML) OR (centrali* NEAR/2 “machine learning”) OR (“multi-party computation” NEAR/2 learn*) OR (“multi-party computation” NEAR/2 model*) OR (“multi-party computation” NEAR/2 train*) OR (“multi-party computation” NEAR/2 tensor*) OR (“multi-party computation” NEAR/2 perceptron) OR (“multi-party computation” NEAR/2 algorithm*) OR (“multi-party computation” NEAR/2 network*) OR (“multi-party computation” NEAR/2 AI) OR (“multi-party computation” NEAR/2 “artificial intelligence”) OR (“multi-party computation” NEAR/2 ML) OR (“multi-party computation” NEAR/2 “machine learning”) OR (blockchain NEAR/2 learn*) OR (blockchain NEAR/2 model*) OR (blockchain NEAR/2 train*) OR (blockchain NEAR/2 tensor*) OR (blockchain NEAR/2 perceptron) OR (blockchain NEAR/2 algorithm*) OR (blockchain NEAR/2 network*) OR (blockchain NEAR/2 AI) OR (blockchain NEAR/2 “artificial intelligence”) OR (blockchain NEAR/2 ML) OR (blockchain NEAR/2 “machine learning”))) ) AND (((TI=(health* OR medic* OR patient* OR clinic* OR physician* OR doctor*)) OR (AB=(health* OR medic* OR patient* OR clinic* OR physician* OR doctor*))))

##### Wiley

**Query**: Validated

**Query Link:** Validated

Revised on 2023.04.04

**Extraction date:** 2023.04.06

Query 1

**Branch #1: Title**

distributed OR federated OR decentrali* OR centrali* OR multi-party computation OR blockchain

**Branch #2: Title**

learn* OR model* OR train* OR tensor* OR perceptron OR algorithm* OR network* OR AI OR “artificial intelligence” OR ML OR “machine learning”

**Branch #3: Anywhere** health* OR medic* OR patient* OR clinic* OR physician* OR doctor*

Limited to “Journals” Limited to Date

Query 2

**Branch #1: Abstract

distributed OR federated OR decentrali* OR centrali* OR multi-party computation OR blockchain

**Branch #2: Abstract**

learn* OR model* OR train* OR tensor* OR perceptron OR algorithm* OR network* OR AI OR “artificial intelligence” OR ML OR “machine learning”

**Branch #3: Anywhere** health* OR medic* OR patient* OR clinic* OR physician* OR doctor*

Limited to “Journals” Limited to Date

Direct Link (Two Queries):

<https://onlinelibrary.wiley.com/action/doSearch?Ppub=&field1=Title&field2=Title&field3=AllField&text1=distributed+OR+federated+OR+decentrali*+OR+centrali*+OR+multi-party+computation+OR+blockchain&text2=learn*+OR+model*+OR+train*+OR+tensor*+OR+perceptron+OR+algorithm*+OR+network*+OR+AI+OR+%22artificial+intelligence%22+OR+ML+OR+%22machine+learning%22&text3=health*+OR+medic*+OR+patient*+OR+clinic*+OR+physician*+OR+doctor*&startPage=&PubType=journal>

Yield = 128

<https://onlinelibrary.wiley.com/action/doSearch?Ppub=&field1=Abstract&field2=Abstract&field3=AllField&text1=distributed+OR+federated+OR+decentrali*+OR+centrali*+OR+multi-party+computation+OR+blockchain&text2=learn*+OR+model*+OR+train*+OR+tensor*+OR+perceptron+OR+algorithm*+OR+network*+OR+AI+OR+%22artificial+intelligence%22+OR+ML+OR+%22machine+learning%22&text3=health*+OR+medic*+OR+patient*+OR+clinic*+OR+physician*+OR+doctor*&startPage=&PubType=journal>

Yield = 3,702

**Procedure:**

- Choose filters (“Journals”, anos), in the sidebar
- Add “&pageSize=500” to the URL and reload page
- Hit the export citations button
- Open javascript console (F12), paste “$(":checkbox").prop("checked", true)” and run
- Unselect and select again any title
- Continue and export as BibTex. Place BibTex files in the Wiley folder
- Repeat for remaining pages (~ # of results/500 pages)
- Run wiley.py

#### Phase 2

##### DL ACM

**Query**: Validated

**Query Link:** Validated

Revised on 2024.03.28

**Extraction date:** 2024.03.28

**Branch #1: Abstract**

distributed OR federated OR decentrali* OR centrali* OR multi-party computation OR blockchain

**Branch #2: Abstract**

learn* OR model* OR train* OR tensor* OR perceptron OR algorithm* OR network* OR AI OR “artificial intelligence” OR ML OR “machine learning”

**Branch #3: Abstract**

health* OR medic* OR patient* OR clinic* OR physician* OR doctor*

**Branch #4: Full Text**

AUROC OR ROC OR “receiver operating characteristic curve” OR F1 OR “Jensen Shannon” OR Jensen-Shannon OR sensitivity OR recall OR specificity OR accuracy OR precision OR “predictive value” OR Dice OR conversion OR “performance”

**Date-limited:** 2023-2024

Filter by “Publication Date: Past year”, “Journals”

**Direct Link (Starting Point):**

Part 1 - Abstract-focused

[https://dl.acm.org/action/doSearch?fillQuickSearch=false&target=advanced&expand=dl&field1=Abstract&text1=distributed+OR+federated+OR+decentrali*+OR+centrali*+OR+"multi-party+computation"+OR+blockchain&field2=Abstract&text2=learn*+OR+model*+OR+train*+OR+tensor*+OR+perceptron+OR+algorithm*+OR+network*+OR+AI+OR+"artificial+intelligence"+OR+ML+OR+"machine+learning"&field3=Fulltext&text3=health*+OR+medic*+OR+patient*+OR+clinic*+OR+physician*+OR+doctor*&field4=Fulltext&text4=AUROC+OR+ROC+OR+"receiver+operating+characteristic+curve"+OR+F1+OR+"Jensen+Shannon"+OR+Jensen-Shannon+OR+sensitivity+OR+recall+OR+specificity+OR+accuracy+OR+precision+OR+"predictive+value"+OR+Dice+OR+conversion+OR+performance&startPage=&EpubDate=[20230406](https://dl.acm.org/action/doSearch?fillQuickSearch=false&target=advanced&expand=dl&field1=Abstract&text1=distributed+OR+federated+OR+decentrali*+OR+centrali*+OR+%22multi-party+computation%22+OR+blockchain&field2=Abstract&text2=learn*+OR+model*+OR+train*+OR+tensor*+OR+perceptron+OR+algorithm*+OR+network*+OR+AI+OR+%22artificial+intelligence%22+OR+ML+OR+%22machine+learning%22&field3=Fulltext&text3=health*+OR+medic*+OR+patient*+OR+clinic*+OR+physician*+OR+doctor*&field4=Fulltext&text4=AUROC+OR+ROC+OR+%22receiver+operating+characteristic+curve%22+OR+F1+OR+%22Jensen+Shannon%22+OR+Jensen-Shannon+OR+sensitivity+OR+recall+OR+specificity+OR+accuracy+OR+precision+OR+%22predictive+value%22+OR+Dice+OR+conversion+OR+performance&startPage=&EpubDate=%5B20230406) TO 202403282359]&queryID=51/6674775442

For Python:

[https://dl.acm.org/action/doSearch?fillQuickSearch=false&target=advanced&expand=dl&field1=Abstract&text1=distributed+OR+federated+OR+decentrali*+OR+centrali*+OR+\"multi-party+computation\"+OR+blockchain&field2=Abstract&text2=learn*+OR+model*+OR+train*+OR+tensor*+OR+perceptron+OR+algorithm*+OR+network*+OR+AI+OR+\"artificial+intelligence\"+OR+ML+OR+\"machine+learning\"&field3=Fulltext&text3=health*+OR+medic*+OR+patient*+OR+clinic*+OR+physician*+OR+doctor*&field4=Fulltext&text4=AUROC+OR+ROC+OR+\"receiver+operating+characteristic+curve\"+OR+F1+OR+\"Jensen+Shannon\"+OR+Jensen-Shannon+OR+sensitivity+OR+recall+OR+specificity+OR+accuracy+OR+precision+OR+\"predictive+value\"+OR+Dice+OR+conversion+OR+performance&startPage=&EpubDate=[20230406](https://dl.acm.org/action/doSearch?fillQuickSearch=false&target=advanced&expand=dl&field1=Abstract&text1=distributed+OR+federated+OR+decentrali*+OR+centrali*+OR+\%22multi-party+computation\%22+OR+blockchain&field2=Abstract&text2=learn*+OR+model*+OR+train*+OR+tensor*+OR+perceptron+OR+algorithm*+OR+network*+OR+AI+OR+\%22artificial+intelligence\%22+OR+ML+OR+\%22machine+learning\%22&field3=Fulltext&text3=health*+OR+medic*+OR+patient*+OR+clinic*+OR+physician*+OR+doctor*&field4=Fulltext&text4=AUROC+OR+ROC+OR+\%22receiver+operating+characteristic+curve\%22+OR+F1+OR+\%22Jensen+Shannon\%22+OR+Jensen-Shannon+OR+sensitivity+OR+recall+OR+specificity+OR+accuracy+OR+precision+OR+\%22predictive+value\%22+OR+Dice+OR+conversion+OR+performance&startPage=&EpubDate=%5B20230406) TO 202403282359]&queryID=51/6674775442

Part 2 - Title-focused

[https://dl.acm.org/action/doSearch?fillQuickSearch=false&target=advanced&expand=dl&field1=Title&text1=distributed+OR+federated+OR+decentrali*+OR+centrali*+OR+"multi-party+computation"+OR+blockchain&field2=Title&text2=learn*+OR+model*+OR+train*+OR+tensor*+OR+perceptron+OR+algorithm*+OR+network*+OR+AI+OR+"artificial+intelligence"+OR+ML+OR+"machine+learning"&field3=Fulltext&text3=health*+OR+medic*+OR+patient*+OR+clinic*+OR+physician*+OR+doctor*&field4=Fulltext&text4=AUROC+OR+ROC+OR+"receiver+operating+characteristic+curve"+OR+F1+OR+"Jensen+Shannon"+OR+Jensen-Shannon+OR+sensitivity+OR+recall+OR+specificity+OR+accuracy+OR+precision+OR+"predictive+value"+OR+Dice+OR+conversion+OR+performance&startPage=&EpubDate=[20230406](https://dl.acm.org/action/doSearch?fillQuickSearch=false&target=advanced&expand=dl&field1=Title&text1=distributed+OR+federated+OR+decentrali*+OR+centrali*+OR+%22multi-party+computation%22+OR+blockchain&field2=Title&text2=learn*+OR+model*+OR+train*+OR+tensor*+OR+perceptron+OR+algorithm*+OR+network*+OR+AI+OR+%22artificial+intelligence%22+OR+ML+OR+%22machine+learning%22&field3=Fulltext&text3=health*+OR+medic*+OR+patient*+OR+clinic*+OR+physician*+OR+doctor*&field4=Fulltext&text4=AUROC+OR+ROC+OR+%22receiver+operating+characteristic+curve%22+OR+F1+OR+%22Jensen+Shannon%22+OR+Jensen-Shannon+OR+sensitivity+OR+recall+OR+specificity+OR+accuracy+OR+precision+OR+%22predictive+value%22+OR+Dice+OR+conversion+OR+performance&startPage=&EpubDate=%5B20230406) TO 202403282359]&queryID=51/6674775442

For Python:

[https://dl.acm.org/action/doSearch?fillQuickSearch=false&target=advanced&expand=dl&field1=Title&text1=distributed+OR+federated+OR+decentrali*+OR+centrali*+OR+\"multi-party+computation\"+OR+blockchain&field2=Title&text2=learn*+OR+model*+OR+train*+OR+tensor*+OR+perceptron+OR+algorithm*+OR+network*+OR+AI+OR+\"artificial+intelligence\"+OR+ML+OR+\"machine+learning\"&field3=Fulltext&text3=health*+OR+medic*+OR+patient*+OR+clinic*+OR+physician*+OR+doctor*&field4=Fulltext&text4=AUROC+OR+ROC+OR+\"receiver+operating+characteristic+curve\"+OR+F1+OR+\"Jensen+Shannon\"+OR+Jensen-Shannon+OR+sensitivity+OR+recall+OR+specificity+OR+accuracy+OR+precision+OR+\"predictive+value\"+OR+Dice+OR+conversion+OR+performance&startPage=&EpubDate=[20230406](https://dl.acm.org/action/doSearch?fillQuickSearch=false&target=advanced&expand=dl&field1=Title&text1=distributed+OR+federated+OR+decentrali*+OR+centrali*+OR+\%22multi-party+computation\%22+OR+blockchain&field2=Title&text2=learn*+OR+model*+OR+train*+OR+tensor*+OR+perceptron+OR+algorithm*+OR+network*+OR+AI+OR+\%22artificial+intelligence\%22+OR+ML+OR+\%22machine+learning\%22&field3=Fulltext&text3=health*+OR+medic*+OR+patient*+OR+clinic*+OR+physician*+OR+doctor*&field4=Fulltext&text4=AUROC+OR+ROC+OR+\%22receiver+operating+characteristic+curve\%22+OR+F1+OR+\%22Jensen+Shannon\%22+OR+Jensen-Shannon+OR+sensitivity+OR+recall+OR+specificity+OR+accuracy+OR+precision+OR+\%22predictive+value\%22+OR+Dice+OR+conversion+OR+performance&startPage=&EpubDate=%5B20230406) TO 202403282359]&queryID=51/6674775442

**Procedure:**

- For each results page, select all results, hitting the checkbox on the upper left corner of the list of results
- Select the option “Export Citations”
- Verify message: “Loading 250 citations…”
- Verify the option “BibTeX” is selected on the menu
- Click the blue arrow to download
- Change file name to “acm_export_01”
- Move to the next page and repeat the process, updating the file name according to the results page number

##### arXiv

**Query**: Validated

**Query Link:** Validated

Revised on 2024.03.20

**Extraction date:** 2024.03.22

Bacthes of 200 exportable articles (out of 1,066 results)

API: <https://info.arxiv.org/help/api/basics.html#using>

Query: order: submitted_date; size: 200; date_range: from 2023-04-06 to 2024-03-20; classification: Computer Science (cs), Electrical Engineering and Systems Science (eess), Quantitative Biology (q-bio); include_cross_list: True; terms: AND abstract=distributed OR federated OR decentrali* OR centrali* OR “multi-party computation” OR blockchain; AND abstract=learn* OR model* OR train* OR tensor* OR perceptron OR algorithm* OR network* OR AI OR “artificial intelligence” OR ML OR “machine learning”; AND abstract=health* OR medic* OR patient* OR clinic* OR physician* OR doctor*

<https://arxiv.org/search/advanced?advanced=&terms-0-operator=AND&terms-0-term=distributed+OR+federated+OR+decentrali*+OR+centrali*+OR+%22multi-party+computation%22+OR+blockchain&terms-0-field=abstract&terms-1-operator=AND&terms-1-term=learn*+OR+model*+OR+train*+OR+tensor*+OR+perceptron+OR+algorithm*+OR+network*+OR+AI+OR+%22artificial+intelligence%22+OR+ML+OR+%22machine+learning%22&terms-1-field=abstract&terms-2-operator=AND&terms-2-term=health*+OR+medic*+OR+patient*+OR+clinic*+OR+physician*+OR+doctor*&terms-2-field=abstract&classification-computer_science=y&classification-eess=y&classification-physics_archives=all&classification-q_biology=y&classification-include_cross_list=include&date-year=&date-filter_by=date_range&date-from_date=2023-04-06&date-to_date=2024-03-20&date-date_type=submitted_date&abstracts=show&size=200&order=submitted_date>

##### IEEE

**Query**: Validated

**Query Link:** Validated

Revised on 2024.03.28

**Extraction date:** 2024.03.28

Filter by “Journals”

**Notes:** There is a wildcard limited. Subscribed content allows for full-text access.

Hit the “Export” button, without additional selections. Exports up to 2.000 results

First, conduct a general export and then a filtered export (by “Subscribed Content”)

In R, separate the “Subscribed Content” and produce two dataframe

**Branch #1: Title or Abstract**

((“Document Title”: distributed OR federated OR decentralised OR decentralized OR centralised OR centralized OR multi-party computation OR blockchain) OR (“Abstract”: distributed OR federated OR decentralised OR decentralized OR centralised OR centralized OR multi-party computation OR blockchain))

- NEAR/2

**Branch #2: Title or Abstract**

((“Document Title”: learn OR learns OR learning OR learned OR learnt OR model OR models OR modeling OR modeled OR network OR networks OR networking OR perceptron OR perceptrons OR AI OR artificial intelligence OR ML OR machine learning OR train OR trains OR training OR trained OR algorithm OR algorithms OR tensor OR tensors) OR (“Abstract”: learn OR learns OR learning OR learned OR learnt OR model OR models OR modeling OR modeled OR network OR networks OR networking OR perceptron OR perceptrons OR AI OR artificial intelligence OR ML OR machine learning OR train OR trains OR training OR trained OR algorithm OR algorithms OR tensor OR tensors))

**Branch #3: Title or Abstract**

((“Document Title”: health OR healthy OR medical OR medicine OR medic OR clinical OR clinic OR clinician OR patient OR patients OR doctor OR doctors OR physician OR physicians) OR (“Abstract”: health OR healthy OR medical OR medicine OR medic OR clinical OR clinic OR clinician OR patient OR patients OR doctor OR doctors OR physician OR physicians))

- Selecionar apenas “Conferences”, “Journals”, “Early Access Articles”, “Magazines”, “Standards”
- Filtrar de 2023 em diante

<https://ieeexplore.ieee.org/search/searchresult.jsp?action=search&matchBoolean=true&queryText=>(((“Document Title”: distributed OR federated OR decentralised OR decentralized OR centralised OR centralized OR multi-party computation OR blockchain) OR (“Abstract”: distributed OR federated OR decentralised OR decentralized OR centralised OR centralized OR multi-party computation OR blockchain)) NEAR%2F2 ((“Document Title”: learn OR learns OR learning OR learned OR learnt OR model OR models OR modeling OR modeled OR network OR networks OR networking OR perceptron OR perceptrons OR AI OR artificial intelligence OR ML OR machine learning OR train OR trains OR training OR trained OR algorithm OR algorithms OR tensor OR tensors) OR (“Abstract”: learn OR learns OR learning OR learned OR learnt OR model OR models OR modeling OR modeled OR network OR networks OR networking OR perceptron OR perceptrons OR AI OR artificial intelligence OR ML OR machine learning OR train OR trains OR training OR trained OR algorithm OR algorithms OR tensor OR tensors)) AND ((“Document Title”: health OR healthy OR medical OR medicine OR medic OR clinical OR clinic OR clinician OR patient OR patients OR doctor OR doctors OR physician OR physicians) OR (“Abstract”: health OR healthy OR medical OR medicine OR medic OR clinical OR clinic OR clinician OR patient OR patients OR doctor OR doctors OR physician OR physicians))%0A)&highlight=true&returnType=SEARCH&matchPubs=true&returnFacets=ALL&ranges=2023_2024_Year&refinements=ContentType:Conferences&refinements=ContentType:Journals&refinements=ContentType:Early Access Articles&refinements=ContentType:Magazines&refinements=ContentType:Standards&rowsPerPage=100&pageNumber=1

All Results: 1,237

Non-Subscribed Content = 30 - To be removed

- Publisher:
  - 13 = IEEE (only some)
  - **2 = KICKS (only some)**
    - Decentralized gene regulatory networks: An approach to energy efficient node coordination with delay constraints for wireless sensor systems
    - Special issue on big data networking-challenges and applications
  - **7 = MIT Press**
  - **1 = PTP**
  - **2 = OUP**
  - **5 = IET**

Subscribed Content = 436

**Branch #4: Full Text**

(“Full Text Only”: AUROC OR ROC OR receiver operating characteristic curve OR F1 OR Jensen Shannon OR Jensen-Shannon OR sensitivity OR recall OR specificity OR accuracy OR precision OR predictive value OR Dice OR conversion OR performance)

<https://ieeexplore.ieee.org/search/searchresult.jsp?action=search&matchBoolean=true&queryText=>(((%20((%22Document%20Title%22:%20distributed%20OR%20federated%20OR%20decentralised%20OR%20decentralized%20OR%20centralised%20OR%20centralized%20OR%20multi-party%20computation%20OR%20blockchain)%20OR%20(%22Abstract%22:%20distributed%20OR%20federated%20OR%20decentralised%20OR%20decentralized%20OR%20centralised%20OR%20centralized%20OR%20multi-party%20computation%20OR%20blockchain))%20NEAR%2F2%20((%22Document%20Title%22:%20learn%20OR%20learns%20OR%20learning%20OR%20learned%20OR%20learnt%20OR%20model%20OR%20models%20OR%20modeling%20OR%20modeled%20OR%20network%20OR%20networks%20OR%20networking%20OR%20perceptron%20OR%20perceptrons%20OR%20AI%20OR%20artificial%20intelligence%20OR%20ML%20OR%20machine%20learning%20OR%20train%20OR%20trains%20OR%20training%20OR%20trained)%20OR%20(%22Abstract%22:%20learn%20OR%20learns%20OR%20learning%20OR%20learned%20OR%20learnt%20OR%20model%20OR%20models%20OR%20modeling%20OR%20modeled%20OR%20network%20OR%20networks%20OR%20networking%20OR%20perceptron%20OR%20perceptrons%20OR%20AI%20OR%20artificial%20intelligence%20OR%20ML%20OR%20machine%20learning%20OR%20train%20OR%20trains%20OR%20training%20OR%20trained))%20)%20AND%20((%22Document%20Title%22:%20health%20OR%20healthy%20OR%20medical%20OR%20medicine%20OR%20medic%20OR%20clinical%20OR%20clinic%20OR%20clinician%20OR%20patient%20OR%20patients)%20OR%20(%22Abstract%22:%20health%20OR%20healthy%20OR%20medical%20OR%20medicine%20OR%20medic%20OR%20clinical%20OR%20clinic%20OR%20clinician%20OR%20patient%20OR%20patients))%20)%20AND%20(%22Full%20Text%20Only%22:%20AUROC%20OR%20ROC%20OR%20receiver%20operating%20characteristic%20curve%20OR%20F1%20OR%20Jensen%20Shannon%20OR%20Jensen-Shannon%20OR%20sensibility%20OR%20recall%20OR%20specificity%20OR%20accuracy%20OR%20precision%20OR%20predictive%20value%20OR%20Dice%20OR%20conversion%20OR%20perfomarnce))&highlight=true&returnType=SEARCH&matchPubs=true&returnFacets=ALL&ranges=2023_2024_Year&subscribed=true&refinements=ContentType:Conferences&refinements=ContentType:Journals&refinements=ContentType:Early%20Access%20Articles&refinements=ContentType:Magazines

##### Lens.org

**Query**: Validated

**Query Link:** Validated

Revised on 2024.03.28

**Extraction date:** 2024.03.28

**Branch #1: Title, Abstract, Keywords or Field of Study**

(title:(“distributed”) OR abstract:(“distributed”) OR keyword:(“distributed”) OR field_of_study:(“distributed”) OR title:(“federated”) OR abstract:(“federated”) OR keyword:(“federated”) OR field_of_study:(“federated”) OR title:(“decentrali*”) OR abstract:(”decentrali*”) OR keyword:(“decentrali*”) OR field_of_study:(”decentrali*”) OR title:(“centrali*”) OR abstract:(”centrali*”) OR keyword:(“centrali*”) OR field_of_study:(”centrali*”) OR title:(“blockchain”) OR abstract:(“blockchain”) OR keyword:(“blockchain”) OR field_of_study:(“blockchain”) OR title:(“multi-party computation”) OR abstract:(“multi-party computation”) OR keyword:(“multi-party computation”) OR field_of_study:(“multi-party computation”))

**Branch #2: Title, Abstract, Keywords or Field of Study**

(title:(learn*) OR abstract:(learn*) OR keyword:(learn*) OR field_of_study:(learn*) OR title:(model*) OR abstract:(model*) OR keyword:(model*) OR field_of_study:(model*) OR title:(network*) OR abstract:(network*) OR keyword:(network*) OR field_of_study:(network*) OR title:(perceptron*) OR abstract:(perceptron*) OR keyword:(perceptron*) OR field_of_study:(perceptron*) OR title:(algorithm*) OR abstract:(algorithm*) OR keyword:(algorithm*) OR field_of_study:(algorithm*) OR title:(AI) OR abstract:(AI) OR keyword:(AI) OR field_of_study:(AI) OR title:(artificial intelligence) OR abstract:(artificial intelligence) OR keyword:(artificial intelligence) OR field_of_study:(artificial intelligence) OR title:(ML) OR abstract:(ML) OR keyword:(ML) OR field_of_study:(ML) OR title:(machine learning) OR abstract:(machine learning) OR keyword:(machine learning) OR field_of_study:(machine learning) OR title:(train*) OR abstract:(train*) OR keyword:(train*) OR field_of_study:(train*))

**Branch #3: Title, Abstract, Keywords or Field of Study**

(title:(health*) OR abstract:(health*) OR keyword:(health*) OR field_of_study:(health*) OR title:(medic*) OR abstract:(medic*) OR keyword:(medic*) OR field_of_study:(medic*) OR title:(clinic*) OR abstract:(clinic*) OR keyword:(clinic*) OR field_of_study:(clinic*) OR title:(patient*) OR abstract:(patient*) OR keyword:(patient*) OR field_of_study:(patient*) OR title:(physician*) OR abstract:(physician*) OR keyword:(physician*) OR field_of_study:(physician*) OR title:(doctor*) OR abstract:(doctor*) OR keyword:(doctor*) OR field_of_study:(doctor*))

**Date-limited:** 2023-04-06 - 2024-03-28

Exclusion of “Book”, “Book chapter”, “Letter”, “Editorial”, “News”, “Review”, “Dataset”, “Component”

**No Full Text** (Segmentation without further searches) Excluding “Has Full Text”

<https://www.lens.org/lens/search/scholar/list?q=(title>:(%22distributed%22)%20OR%20abstract:(%22distributed%22)%20OR%20keyword:(%22distributed%22)%20OR%20field_of_study:(%22distributed%22)%20OR%20title:(%22federated%22)%20OR%20abstract:(%22federated%22)%20OR%20keyword:(%22federated%22)%20OR%20field_of_study:(%22federated%22)%20OR%20title:(%22decentrali*%22)%20OR%20abstract:(%22decentrali*%22)%20OR%20keyword:(%22decentrali*%22)%20OR%20field_of_study:(%22decentrali*%22)%20OR%20title:(%22centrali*%22)%20OR%20abstract:(%22centrali*%22)%20OR%20keyword:(%22centrali*%22)%20OR%20field_of_study:(%22centrali*%22)%20OR%20title:(%22blockchain%22)%20OR%20abstract:(%22blockchain%22)%20OR%20keyword:(%22blockchain%22)%20OR%20field_of_study:(%22blockchain%22)%20OR%20title:(%22multi-party%20computation%22)%20OR%20abstract:(%22multi-party%20computation%22)%20OR%20keyword:(%22multi-party%20computation%22)%20OR%20field_of_study:(%22multi-party%20computation%22))%20AND%20(title:(learn*)%20OR%20abstract:(learn*)%20OR%20keyword:(learn*)%20OR%20field_of_study:(learn*)%20OR%20title:(model*)%20OR%20abstract:(model*)%20OR%20keyword:(model*)%20OR%20field_of_study:(model*)%20OR%20title:(network*)%20OR%20abstract:(network*)%20OR%20keyword:(network*)%20OR%20field_of_study:(network*)%20OR%20title:(perceptron*)%20OR%20abstract:(perceptron*)%20OR%20keyword:(perceptron*)%20OR%20field_of_study:(perceptron*)%20OR%20title:(algorithm*)%20OR%20abstract:(algorithm*)%20OR%20keyword:(algorithm*)%20OR%20field_of_study:(algorithm*)%20OR%20title:(AI)%20OR%20abstract:(AI)%20OR%20keyword:(AI)%20OR%20field_of_study:(AI)%20OR%20title:(artificial%20intelligence)%20OR%20abstract:(artificial%20intelligence)%20OR%20keyword:(artificial%20intelligence)%20OR%20field_of_study:(artificial%20intelligence)%20OR%20title:(ML)%20OR%20abstract:(ML)%20OR%20keyword:(ML)%20OR%20field_of_study:(ML)%20OR%20title:(machine%20learning)%20OR%20abstract:(machine%20learning)%20OR%20keyword:(machine%20learning)%20OR%20field_of_study:(machine%20learning)%20OR%20title:(train*)%20OR%20abstract:(train*)%20OR%20keyword:(train*)%20OR%20field_of_study:(train*))%20AND%20(title:(health*)%20OR%20abstract:(health*)%20OR%20keyword:(health*)%20OR%20field_of_study:(health*)%20OR%20title:(medic*)%20OR%20abstract:(medic*)%20OR%20keyword:(medic*)%20OR%20field_of_study:(medic*)%20OR%20title:(clinic*)%20OR%20abstract:(clinic*)%20OR%20keyword:(clinic*)%20OR%20field_of_study:(clinic*)%20OR%20title:(patient*)%20OR%20abstract:(patient*)%20OR%20keyword:(patient*)%20OR%20field_of_study:(patient*)%20OR%20title:(physician*)%20OR%20abstract:(physician*)%20OR%20keyword:(physician*)%20OR%20field_of_study:(physician*)%20OR%20title:(doctor*)%20OR%20abstract:(doctor*)%20OR%20keyword:(doctor*)%20OR%20field_of_study:(doctor*))&p=0&n=10&s=_score&d=%2B&f=false&e=false&l=en&authorField=author&dateFilterField=publishedDate&orderBy=%2B_score&presentation=false&preview=true&stemmed=true&useAuthorId=false&publicationType.must=journal%20article&publicationType.must=unknown&publicationType.must=conference%20proceedings%20article&publicationType.must=dissertation&publicationType.must=preprint&publicationType.must=conference%20proceedings&publicationType.must=other&publicationType.must=report&publicationType.must=clinical%20trial&publicationType.must=reference%20entry&publicationType.must=journal%20issue&publicationType.must=journal&publicationType.must=journal%20volume&publicationType.must=libguide&publicationType.must=clinical%20study&publicationType.must=standard&publicationType.mustNot=component&publicationType.mustNot=dataset&publicationType.mustNot=book%20chapter&publicationType.mustNot=book&publicationType.mustNot=editorial&publicationType.mustNot=letter&publicationType.mustNot=review&publicationType.mustNot=news&publishedDate.from=2023-04-06&publishedDate.to=2024-03-28&hasFullText=false

Yield: 9,303

**Branch #4: Full Text**

(fulltext:AUROC OR ROC OR “receiver operating characteristic curve” OR F1 OR “Jensen Shannon” OR Jensen-Shannon OR sensitivity OR recall OR specificity OR accuracy OR precision OR “predictive value” OR Dice OR conversion OR performance)

<https://www.lens.org/lens/search/scholar/list?q=(title>:(%22distributed%22)%20OR%20abstract:(%22distributed%22)%20OR%20keyword:(%22distributed%22)%20OR%20field_of_study:(%22distributed%22)%20OR%20title:(%22federated%22)%20OR%20abstract:(%22federated%22)%20OR%20keyword:(%22federated%22)%20OR%20field_of_study:(%22federated%22)%20OR%20title:(%22decentrali*%22)%20OR%20abstract:(%22decentrali*%22)%20OR%20keyword:(%22decentrali*%22)%20OR%20field_of_study:(%22decentrali*%22)%20OR%20title:(%22centrali*%22)%20OR%20abstract:(%22centrali*%22)%20OR%20keyword:(%22centrali*%22)%20OR%20field_of_study:(%22centrali*%22)%20OR%20title:(%22blockchain%22)%20OR%20abstract:(%22blockchain%22)%20OR%20keyword:(%22blockchain%22)%20OR%20field_of_study:(%22blockchain%22)%20OR%20title:(%22multi-party%20computation%22)%20OR%20abstract:(%22multi-party%20computation%22)%20OR%20keyword:(%22multi-party%20computation%22)%20OR%20field_of_study:(%22multi-party%20computation%22))%20AND%20(title:(learn*)%20OR%20abstract:(learn*)%20OR%20keyword:(learn*)%20OR%20field_of_study:(learn*)%20OR%20title:(model*)%20OR%20abstract:(model*)%20OR%20keyword:(model*)%20OR%20field_of_study:(model*)%20OR%20title:(network*)%20OR%20abstract:(network*)%20OR%20keyword:(network*)%20OR%20field_of_study:(network*)%20OR%20title:(perceptron*)%20OR%20abstract:(perceptron*)%20OR%20keyword:(perceptron*)%20OR%20field_of_study:(perceptron*)%20OR%20title:(algorithm*)%20OR%20abstract:(algorithm*)%20OR%20keyword:(algorithm*)%20OR%20field_of_study:(algorithm*)%20OR%20title:(AI)%20OR%20abstract:(AI)%20OR%20keyword:(AI)%20OR%20field_of_study:(AI)%20OR%20title:(artificial%20intelligence)%20OR%20abstract:(artificial%20intelligence)%20OR%20keyword:(artificial%20intelligence)%20OR%20field_of_study:(artificial%20intelligence)%20OR%20title:(ML)%20OR%20abstract:(ML)%20OR%20keyword:(ML)%20OR%20field_of_study:(ML)%20OR%20title:(machine%20learning)%20OR%20abstract:(machine%20learning)%20OR%20keyword:(machine%20learning)%20OR%20field_of_study:(machine%20learning)%20OR%20title:(train*)%20OR%20abstract:(train*)%20OR%20keyword:(train*)%20OR%20field_of_study:(train*))%20AND%20(title:(health*)%20OR%20abstract:(health*)%20OR%20keyword:(health*)%20OR%20field_of_study:(health*)%20OR%20title:(medic*)%20OR%20abstract:(medic*)%20OR%20keyword:(medic*)%20OR%20field_of_study:(medic*)%20OR%20title:(clinic*)%20OR%20abstract:(clinic*)%20OR%20keyword:(clinic*)%20OR%20field_of_study:(clinic*)%20OR%20title:(patient*)%20OR%20abstract:(patient*)%20OR%20keyword:(patient*)%20OR%20field_of_study:(patient*)%20OR%20title:(physician*)%20OR%20abstract:(physician*)%20OR%20keyword:(physician*)%20OR%20field_of_study:(physician*)%20OR%20title:(doctor*)%20OR%20abstract:(doctor*)%20OR%20keyword:(doctor*)%20OR%20field_of_study:(doctor*))%20AND%20(fulltext:AUROC%20OR%20ROC%20OR%20%22receiver%20operating%20characteristic%20curve%22%20OR%20F1%20OR%20%22Jensen%20Shannon%22%20OR%20Jensen-Shannon%20OR%20sensitivity%20OR%20recall%20OR%20specificity%20OR%20accuracy%20OR%20precision%20OR%20%22predictive%20value%22%20OR%20Dice%20OR%20conversion%20OR%20performance)&p=0&n=10&s=date_published&d=-&f=false&e=false&l=en&authorField=author&dateFilterField=publishedDate&orderBy=-date_published&presentation=false&preview=true&stemmed=true&useAuthorId=false&publicationType.must=journal%20article&publicationType.must=unknown&publicationType.must=conference%20proceedings%20article&publicationType.must=preprint&publicationType.must=dissertation&publicationType.must=conference%20proceedings&publicationType.must=other&publicationType.must=report&publicationType.must=clinical%20trial&publicationType.must=journal%20issue&publicationType.must=reference%20entry&publicationType.must=journal%20volume&publicationType.must=journal&publicationType.must=standard&publicationType.mustNot=book%20chapter&publicationType.mustNot=book&publicationType.mustNot=component&publicationType.mustNot=dataset&publicationType.mustNot=letter&publicationType.mustNot=editorial&publicationType.mustNot=news&publicationType.mustNot=review&publishedDate.from=2023-04-06&publishedDate.to=2024-03-28&hasFullText=true

Yield: 680

##### LWW

**Query**: Validated

**Query Link:** Validated

Revised on 2024.03.20

**Extraction date:** 2024.03.28

Exportable to EndNote -> May need conversion: <https://www.bibtex.com/c/endnote-to-bibtex-converter/>

<https://lww.com/pages/results.aspx?txtKeywords=(health+OR+medical+OR+patient+OR+clinical+OR+clinic+OR+physician+OR+doctor)+AND+(distributed+OR+federated+OR+decentralized+OR+decentralised+OR+centralized+OR+centralised+OR+multi-party+computation+OR+blockchain)+AND+(learning+OR+model+OR+training+OR+tensor+OR+perceptron+OR+algorithm+OR+network+OR+AI+OR+artificial+intelligence+OR+ML+OR+machine+learning)>

Using filter “Last 12 Months”

##### medRxiv

**Query**: Validated

**Query Link:** Validated

Revised on 2024.03.20

**Extraction date:** 2024.03.28

Filter by “Publication Date” Remove duplicates from previous query

**Direct Link:**

- 2024 - 379

<https://www.medrxiv.org/search/%20abstract_title%3Afederated%252C%2Bdistributed%252C%2Bdecentrali%252A%252C%2Bmulti-party%2Bcomputation%252C%2Bmulti%2Bparty%2Bcomputation%252C%2Bcentrali%252A%20abstract_title_flags%3Amatch-any%20jcode%3Amedrxiv%20numresults%3A450%20sort%3Arelevance-rank%20format_result%3Astandard?facet%5Bpublication-date%5D%5B0%5D=2024>

- 2023 - 1,240

Sort by newest first Only cover relevant pages

<https://www.medrxiv.org/search/%20abstract_title%3Afederated%252C%2Bdistributed%252C%2Bdecentrali%252A%252C%2Bmulti-party%2Bcomputation%252C%2Bmulti%2Bparty%2Bcomputation%252C%2Bcentrali%252A%20abstract_title_flags%3Amatch-any%20jcode%3Amedrxiv%20numresults%3A450%20sort%3Arelevance-rank%20format_result%3Astandard?facet%5Bpublication-date%5D%5B0%5D=2023>

**Procedure:**

- Select “Add All Citations”
- Repeat for all other pages
- Go to “View Selected Citations”
- Export all results in an EndNote 8 format (xml)

##### Scopus

**Query**: Validated

**Query Link:** Validated

Revised on 2024.03.28

**Note:** Some extra exclusion keywords were added

**Extraction date:** 2024.03.28

Filter by document type

**Branch #1: Title or Abstract**

(TITLE-ABS(distributed OR federated OR decentrali* OR (multi-party computation) OR (multi party computation) OR centrali* OR blockchain )) AND (TITLE-ABS(learn* OR model* OR train* OR tensor* OR perceptron OR algorithm* OR network* OR AI OR (artificial intelligence) OR ML OR (machine learning))) AND (TITLE-ABS(health* OR medic* OR patient* OR clinic* OR physician* OR doctor*))

**Date Limit:** 2023-2024

Limit to: Article, Conference paper, Undefined

Exclude Keywords: Nonhuman, Animal, Animal Experiment, Animal Model, Mouse, Animal Tissue, Mice, Systematic Review, Rat, Animal Cell, “Disease Models, Animal”, Questionnaires, Rats, Animals, “Surveys and Questionnaires”,

Select “Try new version”

( TITLE-ABS ( distributed OR federated OR decentrali* OR ( multi-party AND computation ) OR ( multi AND party AND computation ) OR centrali* OR blockchain ) ) AND ( TITLE-ABS ( learn* OR model* OR train* OR tensor* OR perceptron OR algorithm* OR network* OR ai OR ( artificial AND intelligence ) OR ml OR ( machine AND learning ) ) ) AND ( TITLE-ABS ( health* OR medic* OR patient* OR clinic* OR physician* OR doctor* ) ) AND PUBYEAR > 2011 AND PUBYEAR < 2025 AND ( LIMIT-TO ( DOCTYPE , “ar” ) OR LIMIT-TO ( DOCTYPE , “cp” ) OR LIMIT-TO ( DOCTYPE , “Undefined” ) ) AND ( EXCLUDE ( EXACTKEYWORD , “Nonhuman” ) OR EXCLUDE ( EXACTKEYWORD , “Animals” ) OR EXCLUDE ( EXACTKEYWORD , “Animal” ) OR EXCLUDE ( EXACTKEYWORD , “Animal Experiment” ) OR EXCLUDE ( EXACTKEYWORD , “Animal Model” ) OR EXCLUDE ( EXACTKEYWORD , “Mouse” ) OR EXCLUDE ( EXACTKEYWORD , “Animal Tissue” ) OR EXCLUDE ( EXACTKEYWORD , “Mice” ) OR EXCLUDE ( EXACTKEYWORD , “Surveys And Questionnaires” ) OR EXCLUDE ( EXACTKEYWORD , “Systematic Review” ) OR EXCLUDE ( EXACTKEYWORD , “Rat” ) OR EXCLUDE ( EXACTKEYWORD , “Animal Cell” ) OR EXCLUDE ( EXACTKEYWORD , “Disease Models, Animal” ) OR EXCLUDE ( EXACTKEYWORD , “Questionnaires” ) OR EXCLUDE ( EXACTKEYWORD , “Rats” ) )

**Direct Link:**

<https://www.scopus.com/results/results.uri?sort=plf-f&src=s&sid=a4791c5e81f52e27d54c4db01796a7b4&sot=a&sdt=cl&cluster=scosubtype%2C%22ar%22%2Ct%2C%22cp%22%2Ct%2C%22Undefined%22%2Ct%2Bscoexactkeywords%2C%22Nonhuman%22%2Cf%2C%22Animals%22%2Cf%2C%22Animal%22%2Cf%2C%22Animal+Experiment%22%2Cf%2C%22Animal+Model%22%2Cf%2C%22Mouse%22%2Cf%2C%22Animal+Tissue%22%2Cf%2C%22Mice%22%2Cf%2C%22Surveys+And+Questionnaires%22%2Cf%2C%22Systematic+Review%22%2Cf%2C%22Rat%22%2Cf%2C%22Animal+Cell%22%2Cf%2C%22Disease+Models%2C+Animal%22%2Cf%2C%22Questionnaires%22%2Cf%2C%22Rats%22%2Cf&sessionSearchId=a4791c5e81f52e27d54c4db01796a7b4&origin=resultslist&editSaveSearch=&txGid=104d59dfe9b448b30fbe2d5767c369b3&featureToggles=FEATURE_DOCUMENT_RESULT_MICRO_UI%3A1&limit=10&s=%28+TITLE-ABS+%28+distributed+OR+federated+OR+decentrali*+OR+%28+multi-party+AND+computation+%29+OR+%28+multi+AND+party+AND+computation+%29+OR+centrali*+OR+blockchain+%29+%29+AND+%28+TITLE-ABS+%28+learn*+OR+model*+OR+train*+OR+tensor*+OR+perceptron+OR+algorithm*+OR+network*+OR+ai+OR+%28+artificial+AND+intelligence+%29+OR+ml+OR+%28+machine+AND+learning+%29+%29+%29+AND+%28+TITLE-ABS+%28+health*+OR+medic*+OR+patient*+OR+clinic*+OR+physician*+OR+doctor*+%29+%29+AND+PUBYEAR+%3E+2011+AND+PUBYEAR+%3C+2025&yearFrom=2023&yearTo=2024>

##### Springer Nature

**Query**: Validated

**Query Link:** Validated

Revised on 2024.03.28

**Extraction date:** 2024.03.28

Filter by “Article”, “Last 12 Months”

<https://forums.zotero.org/discussion/28442/mass-import-of-references-from-springer-link>

- Individual extraction of all search results
- Loop using SpringerLink API to extract meta-data
- Create .csv file to import to Rayyan

Example: <http://api.springernature.com/meta/v2/json?q=doi:10.1007/s10462-023-10417-3&api_key=59d958331507afaa2644390c0ea486a6>

(distributed OR federated OR decentrali* OR centrali* OR “multi-party computation” OR blockchain) AND (learn* OR model* OR train* OR tensor* OR perceptron OR algorithm* OR network* OR AI OR “artificial intelligence” OR ML OR “machine learning”) AND (health* OR medic* OR patient* OR clinc* OR physician* OR doctor*)

Add time limit (2023-2024) and other filters (Article, Conference Paper, Reference Work Entry, Conference Proceedings, Reference Work)

###### Distributed

<https://rd.springer.com/search?query=%28distributed+NEAR%2F2+%28learn*+model*+OR+train*+OR+tensor*+OR+perceptron+OR+algorithm*+OR+network*+OR+AI+OR+%22artificial+intelligence%22+OR+ML+OR+%22machine+learning%22%29%29+AND+%28health*+OR+medic*+OR+patient*+OR+clinc*+OR+physician*+OR+doctor*%29&facet-end-year=2024&showAll=true&date-facet-mode=between&facet-start-year=2023>

###### Federated

<https://rd.springer.com/search?query=%28federated+NEAR%2F2+%28learn*+model*+OR+train*+OR+tensor*+OR+perceptron+OR+algorithm*+OR+network*+OR+AI+OR+%22artificial+intelligence%22+OR+ML+OR+%22machine+learning%22%29%29+AND+%28health*+OR+medic*+OR+patient*+OR+clinc*+OR+physician*+OR+doctor*%29&facet-end-year=2024&showAll=true&date-facet-mode=between&facet-start-year=2023>

###### Blockchain

<https://rd.springer.com/search?query=%28blockchain+NEAR%2F2+%28learn*+model*+OR+train*+OR+tensor*+OR+perceptron+OR+algorithm*+OR+network*+OR+AI+OR+%22artificial+intelligence%22+OR+ML+OR+%22machine+learning%22%29%29+AND+%28health*+OR+medic*+OR+patient*+OR+clinc*+OR+physician*+OR+doctor*%29&facet-end-year=2024&showAll=true&date-facet-mode=between&facet-start-year=2023>

###### Decentrali*

<https://rd.springer.com/search?query=%28decentrali*+NEAR%2F2+%28learn*+model*+OR+train*+OR+tensor*+OR+perceptron+OR+algorithm*+OR+network*+OR+AI+OR+%22artificial+intelligence%22+OR+ML+OR+%22machine+learning%22%29%29+AND+%28health*+OR+medic*+OR+patient*+OR+clinc*+OR+physician*+OR+doctor*%29&facet-end-year=2024&showAll=true&date-facet-mode=between&facet-start-year=2023>

###### Centrali*

<https://rd.springer.com/search?query=%28centrali*+NEAR%2F2+%28learn*+model*+OR+train*+OR+tensor*+OR+perceptron+OR+algorithm*+OR+network*+OR+AI+OR+%22artificial+intelligence%22+OR+ML+OR+%22machine+learning%22%29%29+AND+%28health*+OR+medic*+OR+patient*+OR+clinc*+OR+physician*+OR+doctor*%29&facet-end-year=2024&showAll=true&date-facet-mode=between&facet-start-year=2023>

###### MPC

<https://rd.springer.com/search?query=%28%22multi-party+computation%22+NEAR%2F2+%28learn*+model*+OR+train*+OR+tensor*+OR+perceptron+OR+algorithm*+OR+network*+OR+AI+OR+%22artificial+intelligence%22+OR+ML+OR+%22machine+learning%22%29%29+AND+%28health*+OR+medic*+OR+patient*+OR+clinc*+OR+physician*+OR+doctor*%29&facet-end-year=2024&showAll=true&date-facet-mode=between&facet-start-year=2023>

##### Web of Science

**Query**: Validated

**Query Link:** Validated

Revised on 2024.03.20

**Extraction date:** 2024.03.28

Filter by “Article”

Compare with previous results

Batch export of 1.000 results

**Branch #1 & #2 (NEAR/2): Title or Abstract**

(TI=((distributed NEAR/2 learn*) OR (distributed NEAR/2 model*) OR (distributed NEAR/2 train*) OR (distributed NEAR/2 tensor*) OR (distributed NEAR/2 perceptron) OR (distributed NEAR/2 algorithm*) OR (distributed NEAR/2 network*) OR (distributed NEAR/2 AI) OR (distributed NEAR/2 “artificial intelligence”) OR (distributed NEAR/2 ML) OR (distributed NEAR/2 “machine learning”) OR (federated NEAR/2 learn*) OR (federated NEAR/2 model*) OR (federated NEAR/2 train*) OR (federated NEAR/2 tensor*) OR (federated NEAR/2 perceptron) OR (federated NEAR/2 algorithm*) OR (federated NEAR/2 network*) OR (federated NEAR/2 AI) OR (federated NEAR/2 “artificial intelligence”) OR (federated NEAR/2 ML) OR (federated NEAR/2 “machine learning”) OR (decentrali* NEAR/2 learn*) OR (decentrali* NEAR/2 model*) OR (decentrali* NEAR/2 train*) OR (decentrali* NEAR/2 tensor*) OR (decentrali* NEAR/2 perceptron) OR (decentrali* NEAR/2 algorithm*) OR (decentrali* NEAR/2 network*) OR (decentrali* NEAR/2 AI) OR (decentrali* NEAR/2 “artificial intelligence”) OR (decentrali* NEAR/2 ML) OR (decentrali* NEAR/2 “machine learning”) OR (centrali* NEAR/2 learn*) OR (centrali* NEAR/2 model*) OR (centrali* NEAR/2 train*) OR (centrali* NEAR/2 tensor*) OR (centrali* NEAR/2 perceptron) OR (centrali* NEAR/2 algorithm*) OR (centrali* NEAR/2 network*) OR (centrali* NEAR/2 AI) OR (centrali* NEAR/2 “artificial intelligence”) OR (centrali* NEAR/2 ML) OR (centrali* NEAR/2 “machine learning”) OR (“multi-party computation” NEAR/2 learn*) OR (“multi-party computation” NEAR/2 model*) OR (“multi-party computation” NEAR/2 train*) OR (“multi-party computation” NEAR/2 tensor*) OR (“multi-party computation” NEAR/2 perceptron) OR (“multi-party computation” NEAR/2 algorithm*) OR (“multi-party computation” NEAR/2 network*) OR (“multi-party computation” NEAR/2 AI) OR (“multi-party computation” NEAR/2 “artificial intelligence”) OR (“multi-party computation” NEAR/2 ML) OR (“multi-party computation” NEAR/2 “machine learning”) OR (blockchain NEAR/2 learn*) OR (blockchain NEAR/2 model*) OR (blockchain NEAR/2 train*) OR (blockchain NEAR/2 tensor*) OR (blockchain NEAR/2 perceptron) OR (blockchain NEAR/2 algorithm*) OR (blockchain NEAR/2 network*) OR (blockchain NEAR/2 AI) OR (blockchain NEAR/2 “artificial intelligence”) OR (blockchain NEAR/2 ML) OR (blockchain NEAR/2 “machine learning”))) OR (AB=((distributed NEAR/2 learn*) OR (distributed NEAR/2 model*) OR (distributed NEAR/2 train*) OR (distributed NEAR/2 tensor*) OR (distributed NEAR/2 perceptron) OR (distributed NEAR/2 algorithm*) OR (distributed NEAR/2 network*) OR (distributed NEAR/2 AI) OR (distributed NEAR/2 “artificial intelligence”) OR (distributed NEAR/2 ML) OR (distributed NEAR/2 “machine learning”) OR (federated NEAR/2 learn*) OR (federated NEAR/2 model*) OR (federated NEAR/2 train*) OR (federated NEAR/2 tensor*) OR (federated NEAR/2 perceptron) OR (federated NEAR/2 algorithm*) OR (federated NEAR/2 network*) OR (federated NEAR/2 AI) OR (federated NEAR/2 “artificial intelligence”) OR (federated NEAR/2 ML) OR (federated NEAR/2 “machine learning”) OR (decentrali* NEAR/2 learn*) OR (decentrali* NEAR/2 model*) OR (decentrali* NEAR/2 train*) OR (decentrali* NEAR/2 tensor*) OR (decentrali* NEAR/2 perceptron) OR (decentrali* NEAR/2 algorithm*) OR (decentrali* NEAR/2 network*) OR (decentrali* NEAR/2 AI) OR (decentrali* NEAR/2 “artificial intelligence”) OR (decentrali* NEAR/2 ML) OR (decentrali* NEAR/2 “machine learning”) OR (centrali* NEAR/2 learn*) OR (centrali* NEAR/2 model*) OR (centrali* NEAR/2 train*) OR (centrali* NEAR/2 tensor*) OR (centrali* NEAR/2 perceptron) OR (centrali* NEAR/2 algorithm*) OR (centrali* NEAR/2 network*) OR (centrali* NEAR/2 AI) OR (centrali* NEAR/2 “artificial intelligence”) OR (centrali* NEAR/2 ML) OR (centrali* NEAR/2 “machine learning”) OR (“multi-party computation” NEAR/2 learn*) OR (“multi-party computation” NEAR/2 model*) OR (“multi-party computation” NEAR/2 train*) OR (“multi-party computation” NEAR/2 tensor*) OR (“multi-party computation” NEAR/2 perceptron) OR (“multi-party computation” NEAR/2 algorithm*) OR (“multi-party computation” NEAR/2 network*) OR (“multi-party computation” NEAR/2 AI) OR (“multi-party computation” NEAR/2 “artificial intelligence”) OR (“multi-party computation” NEAR/2 ML) OR (“multi-party computation” NEAR/2 “machine learning”) OR (blockchain NEAR/2 learn*) OR (blockchain NEAR/2 model*) OR (blockchain NEAR/2 train*) OR (blockchain NEAR/2 tensor*) OR (blockchain NEAR/2 perceptron) OR (blockchain NEAR/2 algorithm*) OR (blockchain NEAR/2 network*) OR (blockchain NEAR/2 AI) OR (blockchain NEAR/2 “artificial intelligence”) OR (blockchain NEAR/2 ML) OR (blockchain NEAR/2 “machine learning”)))

**Branch #3: Title or Abstract**

((TI=(health* OR medic* OR patient* OR clinic* OR physician* OR doctor*)) OR (AB=(health* OR medic* OR patient* OR clinic* OR physician* OR doctor*)))

**Date-limited:** 2023-2024

**Document Types:** “Article”, “Proceeding Paper”, “Early Access”, “Meeting Abstract”, “Correction”

Yield: 1,505

**Query:**

<https://www.webofscience.com/wos/woscc/summary/5cd49d11-47ac-45ed-80c2-71eb573c3094-d6ffa1d2/date-ascending/1>

((TI=((distributed NEAR/2 learn*) OR (distributed NEAR/2 model*) OR (distributed NEAR/2 train*) OR (distributed NEAR/2 tensor*) OR (distributed NEAR/2 perceptron) OR (distributed NEAR/2 algorithm*) OR (distributed NEAR/2 network*) OR (distributed NEAR/2 AI) OR (distributed NEAR/2 “artificial intelligence”) OR (distributed NEAR/2 ML) OR (distributed NEAR/2 “machine learning”) OR (federated NEAR/2 learn*) OR (federated NEAR/2 model*) OR (federated NEAR/2 train*) OR (federated NEAR/2 tensor*) OR (federated NEAR/2 perceptron) OR (federated NEAR/2 algorithm*) OR (federated NEAR/2 network*) OR (federated NEAR/2 AI) OR (federated NEAR/2 “artificial intelligence”) OR (federated NEAR/2 ML) OR (federated NEAR/2 “machine learning”) OR (decentrali* NEAR/2 learn*) OR (decentrali* NEAR/2 model*) OR (decentrali* NEAR/2 train*) OR (decentrali* NEAR/2 tensor*) OR (decentrali* NEAR/2 perceptron) OR (decentrali* NEAR/2 algorithm*) OR (decentrali* NEAR/2 network*) OR (decentrali* NEAR/2 AI) OR (decentrali* NEAR/2 “artificial intelligence”) OR (decentrali* NEAR/2 ML) OR (decentrali* NEAR/2 “machine learning”) OR (centrali* NEAR/2 learn*) OR (centrali* NEAR/2 model*) OR (centrali* NEAR/2 train*) OR (centrali* NEAR/2 tensor*) OR (centrali* NEAR/2 perceptron) OR (centrali* NEAR/2 algorithm*) OR (centrali* NEAR/2 network*) OR (centrali* NEAR/2 AI) OR (centrali* NEAR/2 “artificial intelligence”) OR (centrali* NEAR/2 ML) OR (centrali* NEAR/2 “machine learning”) OR (“multi-party computation” NEAR/2 learn*) OR (“multi-party computation” NEAR/2 model*) OR (“multi-party computation” NEAR/2 train*) OR (“multi-party computation” NEAR/2 tensor*) OR (“multi-party computation” NEAR/2 perceptron) OR (“multi-party computation” NEAR/2 algorithm*) OR (“multi-party computation” NEAR/2 network*) OR (“multi-party computation” NEAR/2 AI) OR (“multi-party computation” NEAR/2 “artificial intelligence”) OR (“multi-party computation” NEAR/2 ML) OR (“multi-party computation” NEAR/2 “machine learning”) OR (blockchain NEAR/2 learn*) OR (blockchain NEAR/2 model*) OR (blockchain NEAR/2 train*) OR (blockchain NEAR/2 tensor*) OR (blockchain NEAR/2 perceptron) OR (blockchain NEAR/2 algorithm*) OR (blockchain NEAR/2 network*) OR (blockchain NEAR/2 AI) OR (blockchain NEAR/2 “artificial intelligence”) OR (blockchain NEAR/2 ML) OR (blockchain NEAR/2 “machine learning”))) OR (AB=((distributed NEAR/2 learn*) OR (distributed NEAR/2 model*) OR (distributed NEAR/2 train*) OR (distributed NEAR/2 tensor*) OR (distributed NEAR/2 perceptron) OR (distributed NEAR/2 algorithm*) OR (distributed NEAR/2 network*) OR (distributed NEAR/2 AI) OR (distributed NEAR/2 “artificial intelligence”) OR (distributed NEAR/2 ML) OR (distributed NEAR/2 “machine learning”) OR (federated NEAR/2 learn*) OR (federated NEAR/2 model*) OR (federated NEAR/2 train*) OR (federated NEAR/2 tensor*) OR (federated NEAR/2 perceptron) OR (federated NEAR/2 algorithm*) OR (federated NEAR/2 network*) OR (federated NEAR/2 AI) OR (federated NEAR/2 “artificial intelligence”) OR (federated NEAR/2 ML) OR (federated NEAR/2 “machine learning”) OR (decentrali* NEAR/2 learn*) OR (decentrali* NEAR/2 model*) OR (decentrali* NEAR/2 train*) OR (decentrali* NEAR/2 tensor*) OR (decentrali* NEAR/2 perceptron) OR (decentrali* NEAR/2 algorithm*) OR (decentrali* NEAR/2 network*) OR (decentrali* NEAR/2 AI) OR (decentrali* NEAR/2 “artificial intelligence”) OR (decentrali* NEAR/2 ML) OR (decentrali* NEAR/2 “machine learning”) OR (centrali* NEAR/2 learn*) OR (centrali* NEAR/2 model*) OR (centrali* NEAR/2 train*) OR (centrali* NEAR/2 tensor*) OR (centrali* NEAR/2 perceptron) OR (centrali* NEAR/2 algorithm*) OR (centrali* NEAR/2 network*) OR (centrali* NEAR/2 AI) OR (centrali* NEAR/2 “artificial intelligence”) OR (centrali* NEAR/2 ML) OR (centrali* NEAR/2 “machine learning”) OR (“multi-party computation” NEAR/2 learn*) OR (“multi-party computation” NEAR/2 model*) OR (“multi-party computation” NEAR/2 train*) OR (“multi-party computation” NEAR/2 tensor*) OR (“multi-party computation” NEAR/2 perceptron) OR (“multi-party computation” NEAR/2 algorithm*) OR (“multi-party computation” NEAR/2 network*) OR (“multi-party computation” NEAR/2 AI) OR (“multi-party computation” NEAR/2 “artificial intelligence”) OR (“multi-party computation” NEAR/2 ML) OR (“multi-party computation” NEAR/2 “machine learning”) OR (blockchain NEAR/2 learn*) OR (blockchain NEAR/2 model*) OR (blockchain NEAR/2 train*) OR (blockchain NEAR/2 tensor*) OR (blockchain NEAR/2 perceptron) OR (blockchain NEAR/2 algorithm*) OR (blockchain NEAR/2 network*) OR (blockchain NEAR/2 AI) OR (blockchain NEAR/2 “artificial intelligence”) OR (blockchain NEAR/2 ML) OR (blockchain NEAR/2 “machine learning”))) ) AND (((TI=(health* OR medic* OR patient* OR clinic* OR physician* OR doctor*)) OR (AB=(health* OR medic* OR patient* OR clinic* OR physician* OR doctor*))))

##### Wiley

Query 1

**Branch #1: Title**

distributed OR federated OR decentrali* OR centrali* OR multi-party computation OR blockchain

**Branch #2: Title**

learn* OR model* OR train* OR tensor* OR perceptron OR algorithm* OR network* OR AI OR “artificial intelligence” OR ML OR “machine learning”

**Branch #3: Anywhere** health* OR medic* OR patient* OR clinic* OR physician* OR doctor*

Limited to “Journals” Limited to Date

Query 2

**Branch #1: Abstract

distributed OR federated OR decentrali* OR centrali* OR multi-party computation OR blockchain

**Branch #2: Abstract**

learn* OR model* OR train* OR tensor* OR perceptron OR algorithm* OR network* OR AI OR “artificial intelligence” OR ML OR “machine learning”

**Branch #3: Anywhere** health* OR medic* OR patient* OR clinic* OR physician* OR doctor*

Limited to “Journals” Limited to Date

Direct Link (Two Queries):

<https://onlinelibrary.wiley.com/action/doSearch?PubType=journal&field1=Title&field2=Title&field3=AllField&text1=distributed+OR+federated+OR+decentrali*+OR+centrali*+OR+multi-party+computation+OR+blockchain&text2=learn*+OR+model*+OR+train*+OR+tensor*+OR+perceptron+OR+algorithm*+OR+network*+OR+AI+OR+%22artificial+intelligence%22+OR+ML+OR+%22machine+learning%22&text3=health*+OR+medic*+OR+patient*+OR+clinic*+OR+physician*+OR+doctor*&startPage=&Ppub=%5B20230320%20TO%20202403282359%5D>

Using filter “Last 12 Months”

Yield = 13

<https://onlinelibrary.wiley.com/action/doSearch?PubType=journal&field1=Abstract&field2=Abstract&field3=AllField&text1=distributed+OR+federated+OR+decentrali*+OR+centrali*+OR+multi-party+computation+OR+blockchain&text2=learn*+OR+model*+OR+train*+OR+tensor*+OR+perceptron+OR+algorithm*+OR+network*+OR+AI+OR+%22artificial+intelligence%22+OR+ML+OR+%22machine+learning%22&text3=health*+OR+medic*+OR+patient*+OR+clinic*+OR+physician*+OR+doctor*&startPage=&Ppub=%5B20230320%20TO%20202403282359%5D>

Using filter “Last 12 Months”

Yield = 785

**Procedure:**

- Choose filters (“Journals”, anos), in the sidebar
- Add “&pageSize=500” to the URL and reload page
- Hit the export citations button
- Open javascript console (F12), paste “$(":checkbox").prop("checked", true)” and run
- Unselect and select again any title
- Continue and export as BibTex. Place BibTex files in the Wiley folder
- Repeat for remaining pages (~ # of results/500 pages)
- Run wiley.py

## Results Filtration

As some indispensable terms are very prevalent in publications, such as “model” and “distribution,” increasing the relative useful yield of the query and the number of studies retrieved, we conducted a processing task to filter irrelevant studies.

To do so, using RegEx code in R, we simulated a “within” operator. It was developed to only capture studies in which the term referring to the model architecture (group A) and the one referring to the model synonym (group B) have no more than 2 other terms separating them. Finally, while this process does not perfectly compensate for the limitation in the databases search features and variation, it is expected to offset the most significant differences and not significantly compromise the pursuit of relevant primary papers.

## Duplicates removal

Before the screening phase, duplicate results were removed according to the following criteria: exactly the same lowercase DOI link, exactly the same title-abstract pair after trimming whitespaces and converting all text to lowercase, differing only in a full-stop in the title or abstract.

## Additional Considerations

While it was originally intended to conduct a single group of queries for this review, due to delays in the articles classification and data retrieval, it was considered appropriate to repeat the search strategy. The second query moment was able to cover almost a year of new evidence that would become an invaluable addition to this work. In addition, duplicate results (ie., articles retrieved for both query moments) were removed from the selection process.

Given the heterogeneity of the search engines used several different query procedures were adopted. The approaches for each source, in both moments, are presented in the “Availability of data, code and other materials” section. This documentation includes the exact search strings used, a URL for the query and/or results (if possible), and other details, such as filters applied.

## Main Changes to Protocol

The main alterations were made to the data collection process, the appraisal of the included articles and reporting bias assessments. First, some of the variables for data collection were removed due to very inconsistent reporting (e.g., number of observations, number of model variables, data volume, localization of original data holders, physical location of the data analyses, ethics and legal permissions, specific data format and conversion processes, advanced details on the model architecture, model development orchestration details and specific reason for using decentralized approaches, as well as reported challenges and limitations), being difficult to standardize across studies (e.g., PICO question), or being of little relevance (e.g., research institution country or countries). Moreover, the reporting of specific dimensions of privacy cost and resource consumption was distilled into a single parameter. Second, the application of the CHARMS and PROBAST tools was considered too demanding for the quality of the data reported by primary articles included, as most did not feature a traditional clinical trial design, used a pre-existing dataset without detailed description of the data used, featured a proof of concept nature or did not report the predictors and outcomes definitions and assessments. Additionally, it was decided to abandon direct contacts with corresponding authors of the included primary articles due to the onerous process and the lack of meta-analysis. In addition, although the search strategy did not change, a second moment for further evidence retrieval was added. Minor changes were made information sources, removing the organized additional articles retrieval. While various solicitations were made, no article provided was either outside the list of appraised studies or relevant for the eligibility criteria. Critical parts such as aims and objectives, eligibility criteria and selection process were unchanged from the initial proposal.

## Data Items

Regarding general article information, the following data items were collected: title, abstract, authorship, scientific journal of publication, year of publication, link. Moreover, each article was assessed in terms of the “Transparent Reporting of a multivariable prediction model for Individual Prognosis or Diagnosis” (TRIPOD) statement type^46^ of the featured decentralized learning models and whether they follow a federated learning architecture. For a given study, it was recorded whether the data and code used were reported as well as the circumstances under which they are available.

Within each article, for every individual decentralized model presented, data was extracted in terms of their specific learning architecture (e.g., federated learning, swarm learning), their clinical application, and the clinical domain (and broader clinical domain) to which they were applied. As far as the data they used, we collected the names of the datasets used (if available), whether the data was real, synthetic or both, whether data was collected primarily for the study or if it was used secondarily, the number of datasets used, the type of datasets used (e.g., healthcare information systems data) and the type of data used (e.g., X-ray). In addition, it was recorded if additional privacy preserving techniques were employed (i.e., encryption, differential privacy or noise, data augmentation, or other) as well as whether some development or application costs were quantified (e.g., computation time, energy consumption, privacy compromise).

For each decentralized learning model, we recorded information on the performance comparison with their non-decentralized learning counterparts. We identified the different model natures (i.e., decentralized, local and centralized) and, for a given performance metric, we identified the value (or interval) reported, alongside the uncertainty metric adopted (i.e., confidence interval or standard deviation), as well as its respective value. Whenever applicable, information was collected on the type of statistical comparison between the values, between decentralized and non-decentralized performances, as well as the value of the statistical test and its interpretation.

As often there were many variations of models to compare, e.g., many different local models. Moreover, permutations of different comparisons were collected to reflect as much of the information reported as possible.

No assumptions will be made regarding missing or unclear information—those findings will be reported as such. Each article and decentralized model were given a unique identifier for data processing purposes.


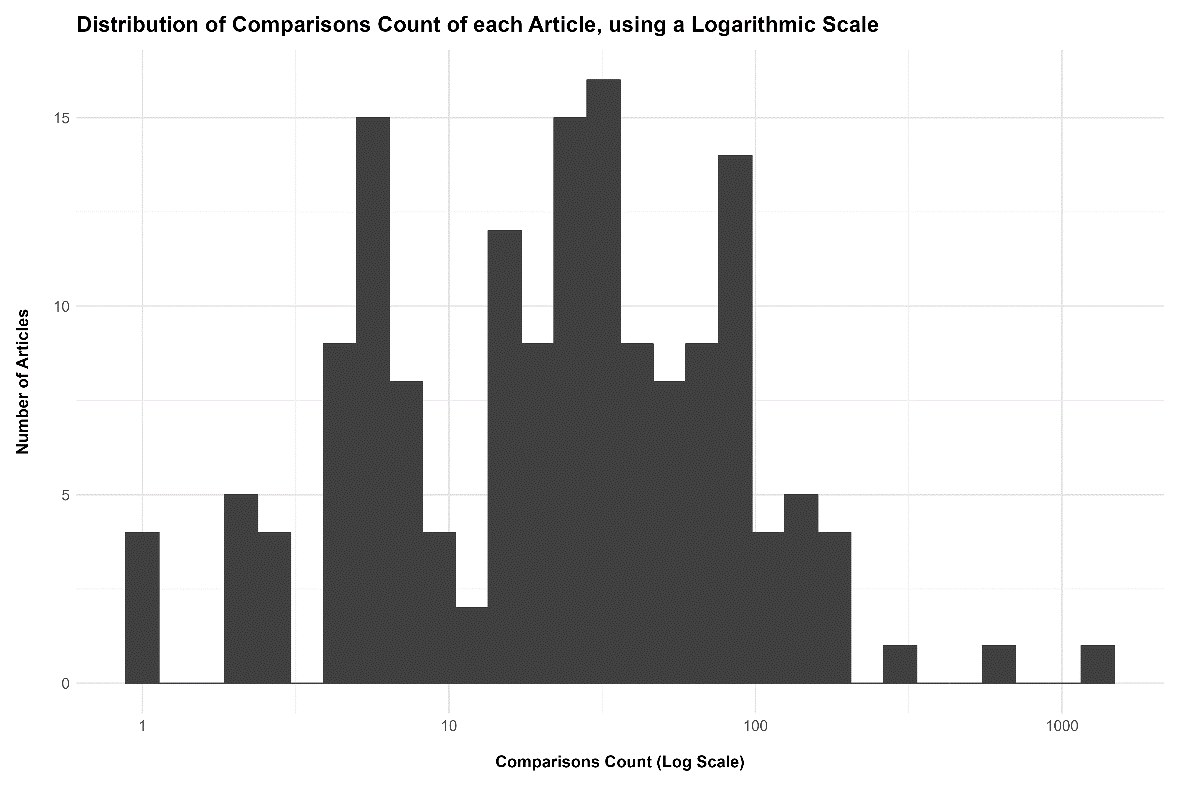


Supplementary Figure 1 - Distribution of comparisons counts of each article, using a logarithmic scale

## Performance Analyses

These figures present detailed distributions of performance differences between decentralized and non-decentralized learning approaches across all metrics. Histograms show the percentage and absolute number of comparisons at each performance difference level (x-axis: non-decentralized minus decentralized values). Negative values indicate decentralized superiority; positive values favour non-decentralized approaches. These distributions complement main text summary statistics and reveal the full range of performance variations underlying aggregate findings.


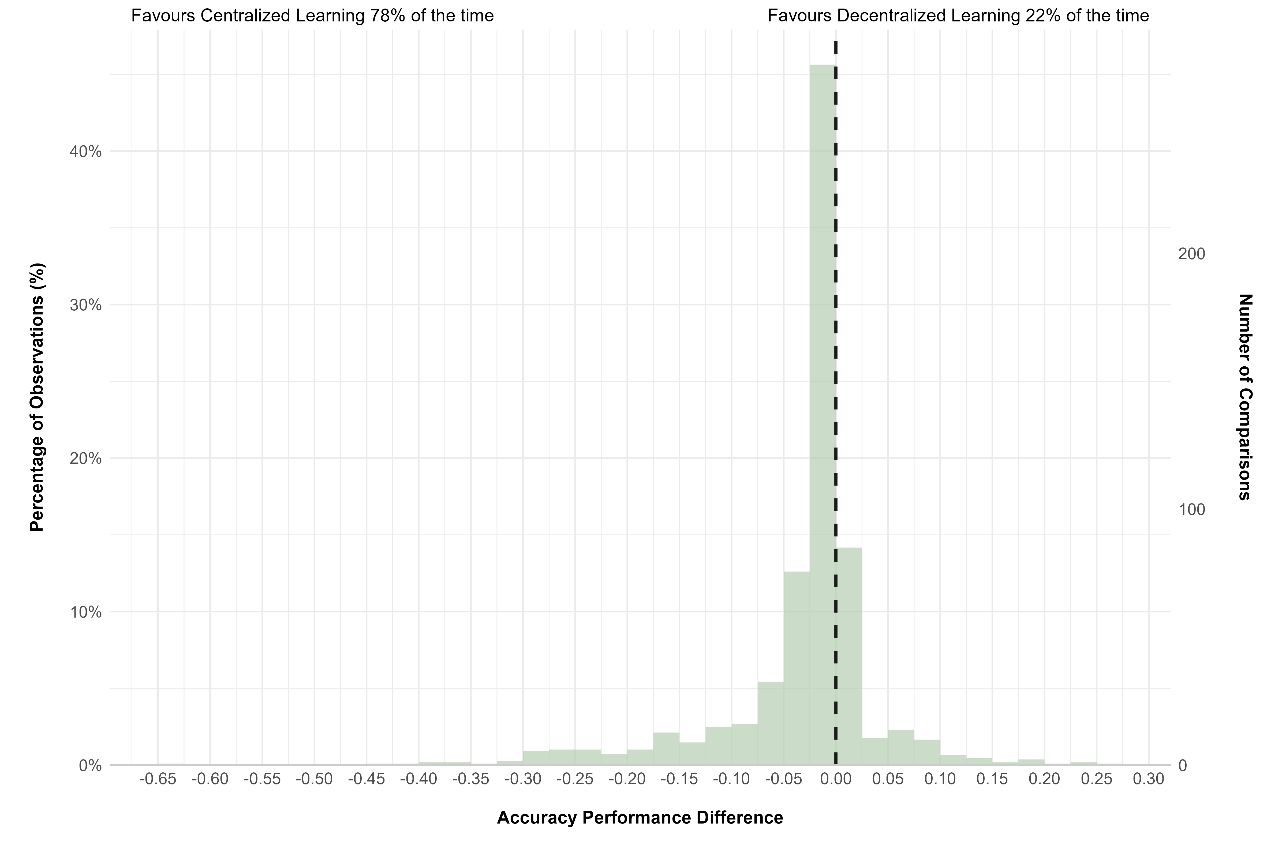


Supplementary Figure 2 - Distribution of Individual Model Performance Differences - Across Accuracy (Comparing Decentralized Learning versus Centralized Learning). Based on 1089 observations extracted from 212 models of 66 studies. Summary Results: Bootstrapped 95% CI: (-0.0342, -0.025 | 25th Percentile = -0.0364 | 75th Percentile = 0.0000 | Mean difference: -0.0295. Note: Dashed vertical line indicates no difference in performance between compared approaches.


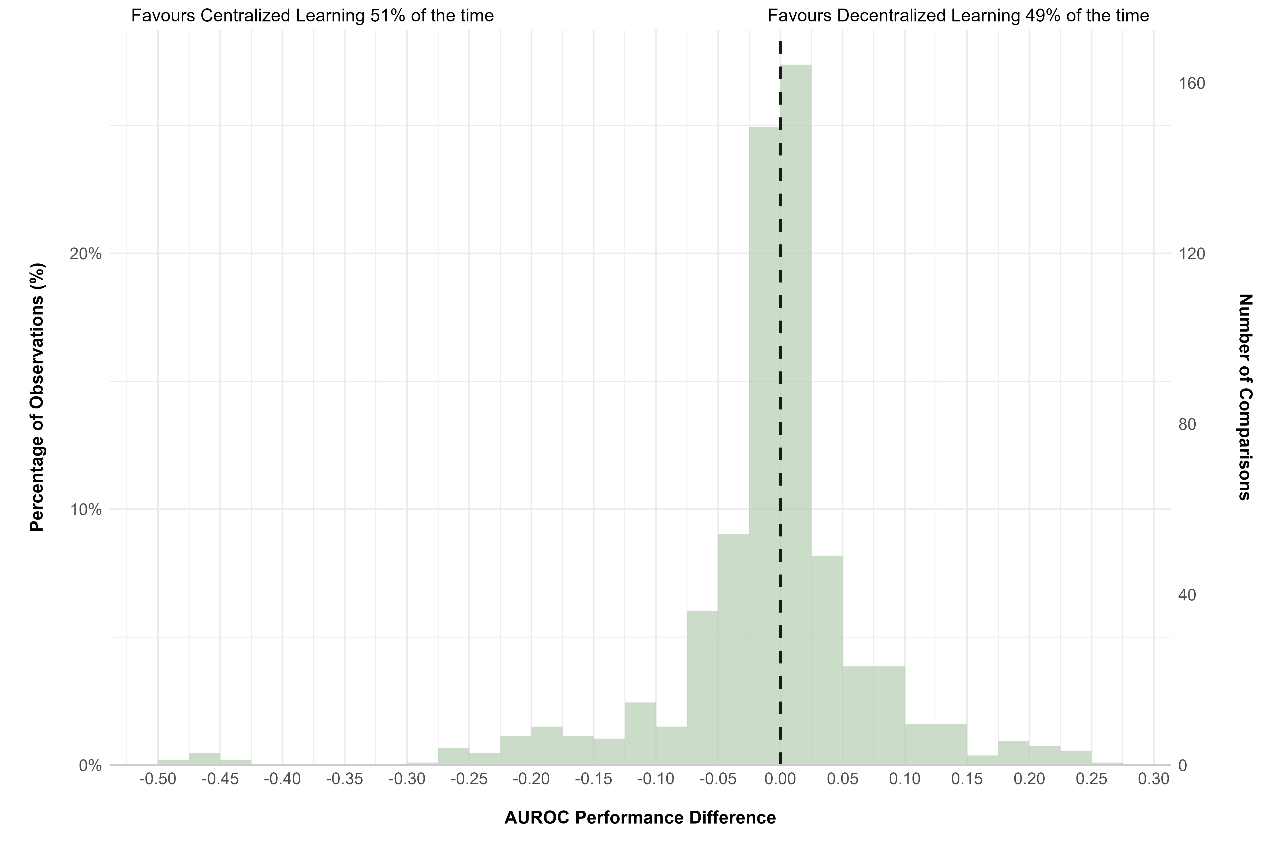


Supplementary Figure 3 - Distribution of Individual Model Performance Differences - Across AUROC (Comparing Decentralized Learning versus Centralized Learning). Based on 1063 observations extracted from 212 models of 42 studies. Summary Results: Bootstrapped 95% CI: (-0.0128, -0.0027 | 25th Percentile = -0.0287 | 75th Percentile = 0.0190 | Mean difference: -0.0076. Note: Dashed vertical line indicates no difference in performance between compared approaches.


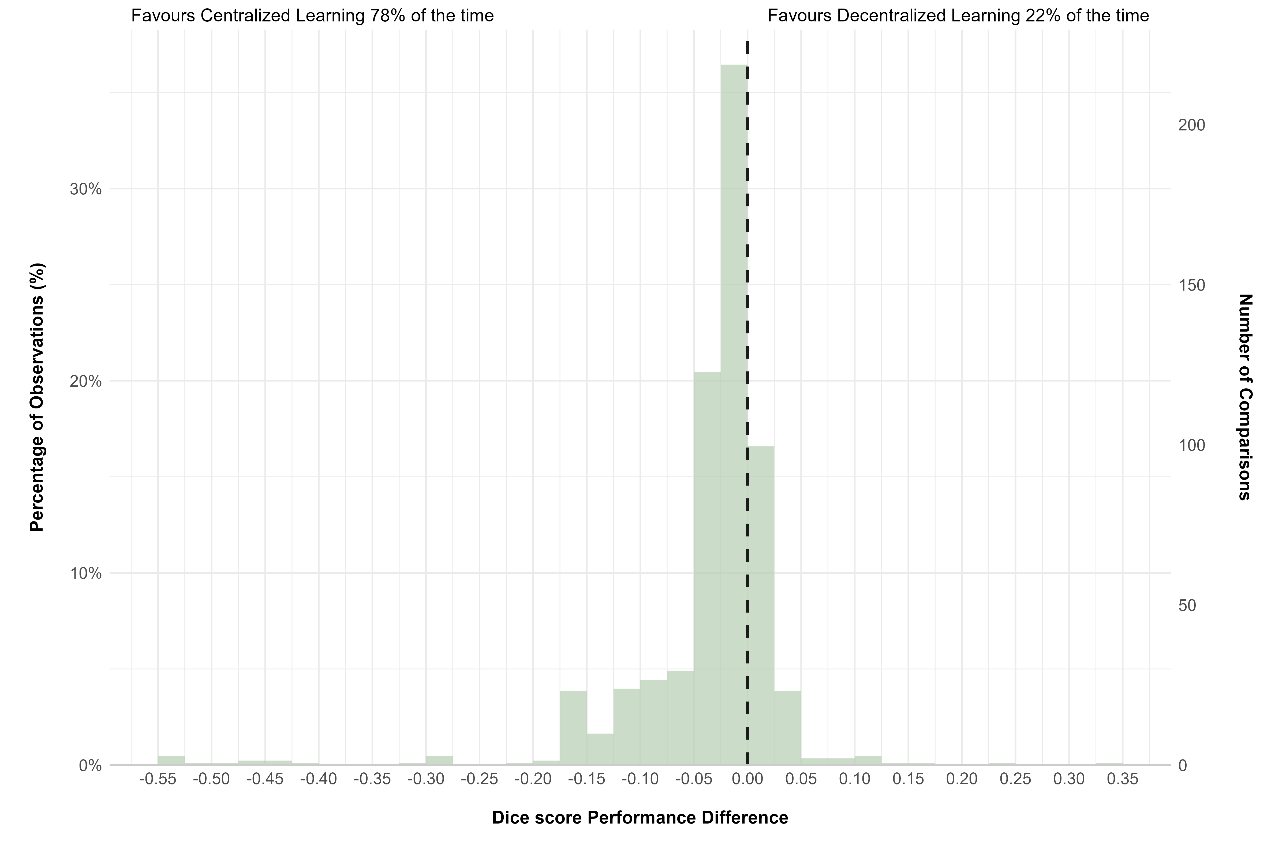


Supplementary Figure 4 - Distribution of Individual Model Performance Differences - Across Dice score (Comparing Decentralized Learning versus Centralized Learning). Based on 856 observations extracted from 127 models of 24 studies. Summary Results: Bootstrapped 95% CI: (-0.0393, -0.0294 | 25th Percentile = -0.0411 | 75th Percentile = -0.0021 | Mean difference: -0.0342. Note: Dashed vertical line indicates no difference in performance between compared approaches.


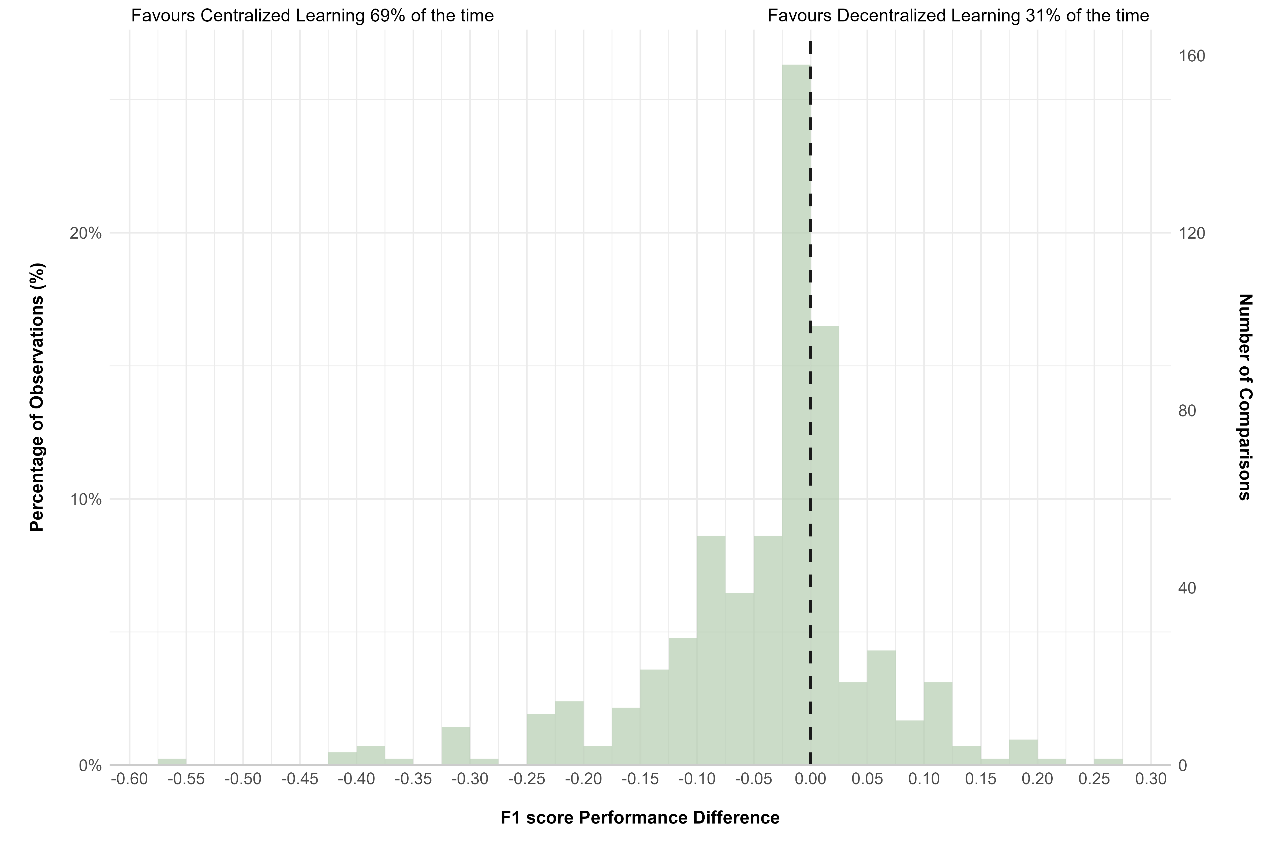


Supplementary Figure 5 - Distribution of Individual Model Performance Differences - Across F1 score (Comparing Decentralized Learning versus Centralized Learning). Based on 420 observations extracted from 98 models of 32 studies. Summary Results: Bootstrapped 95% CI: (-0.0488, -0.03 | 25th Percentile = -0.0825 | 75th Percentile = 0.0034 | Mean difference: -0.0393. Note: Dashed vertical line indicates no difference in performance between compared approaches.


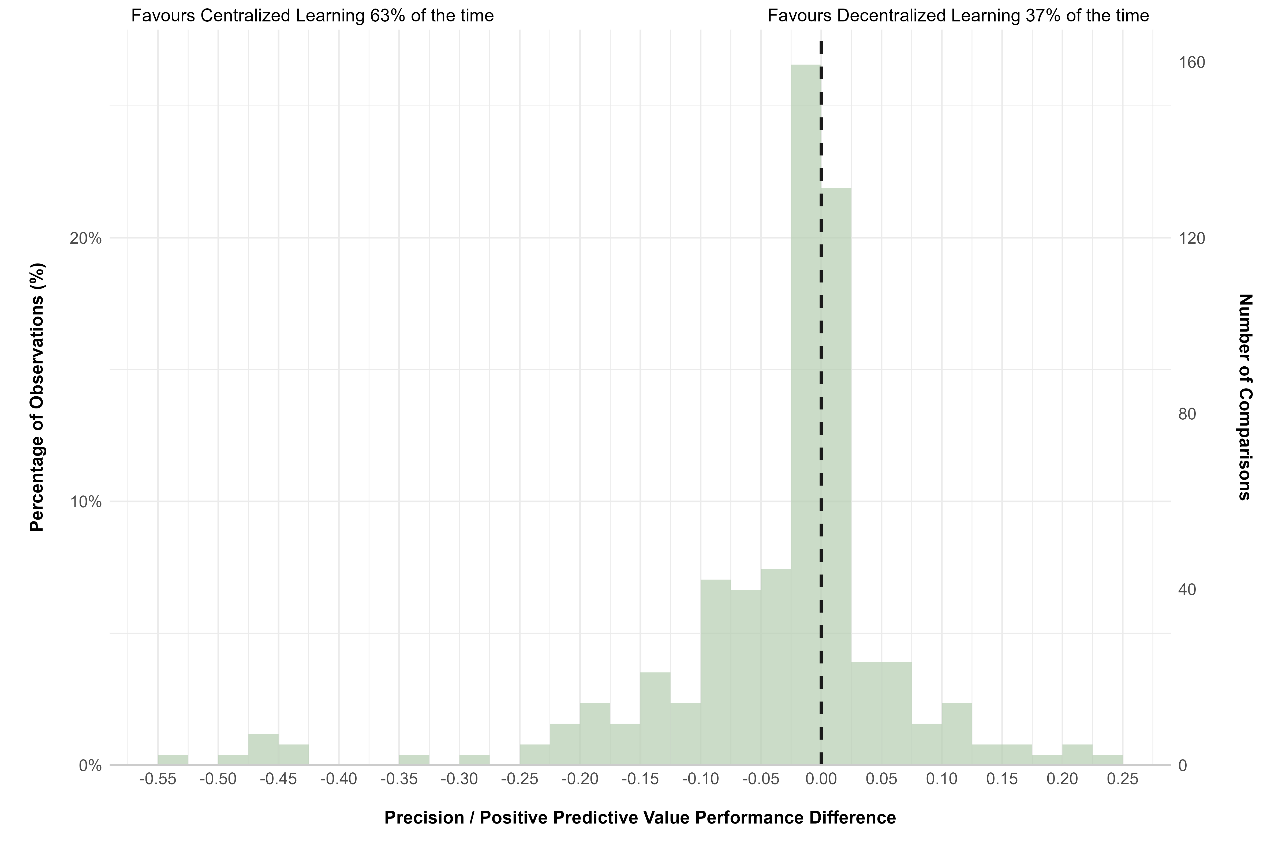


Supplementary Figure 6 - Distribution of Individual Model Performance Differences - Across Precision / Positive Predictive Value (Comparing Decentralized Learning versus Centralized Learning). Based on 258 observations extracted from 80 models of 29 studies. Summary Results: Bootstrapped 95% CI: (-0.0475, -0.0214 | 25th Percentile = -0.0640 | 75th Percentile = 0.0080 | Mean difference: -0.0343. Note: Dashed vertical line indicates no difference in performance between compared approaches.


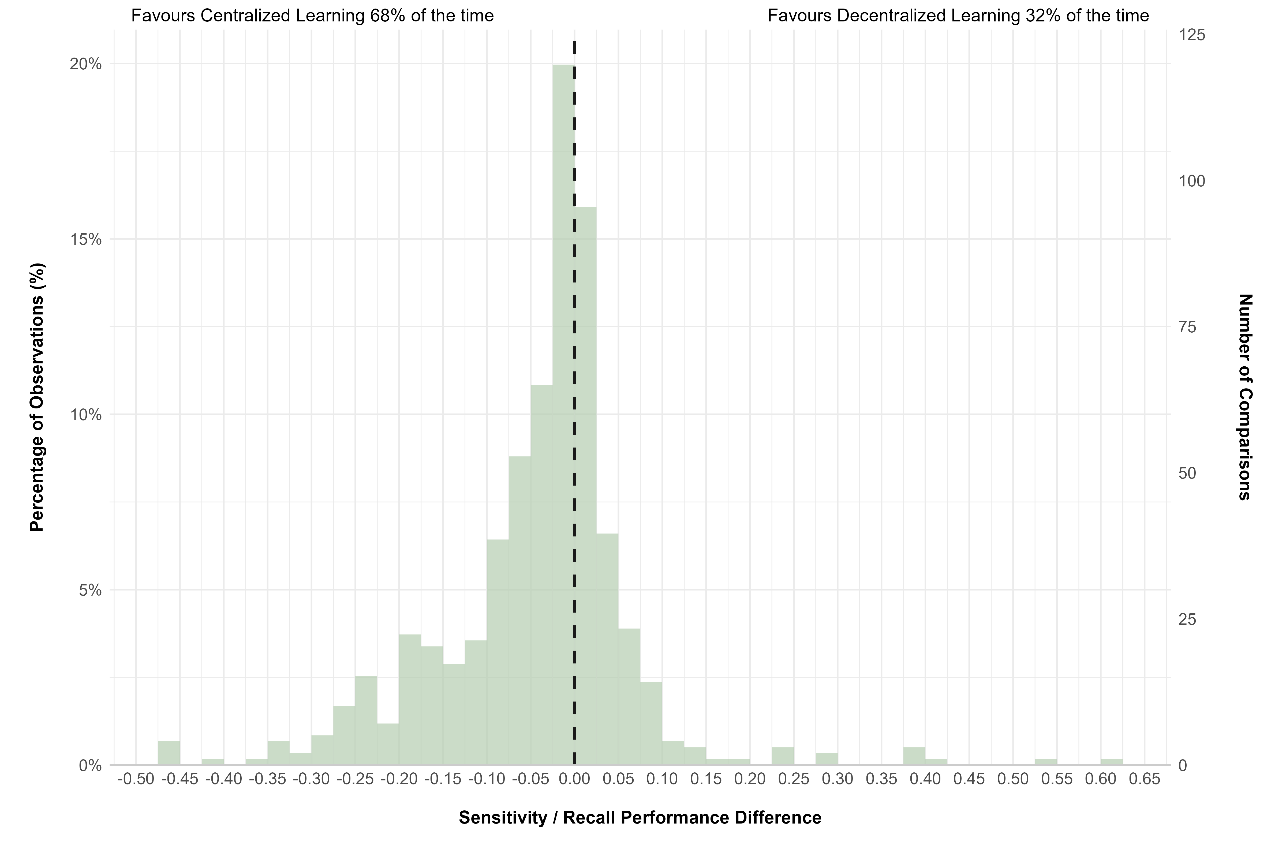


Supplementary Figure 7 - Distribution of Individual Model Performance Differences - Across Sensitivity / Recall (Comparing Decentralized Learning versus Centralized Learning). Based on 593 observations extracted from 120 models of 42 studies. Summary Results: Bootstrapped 95% CI: (-0.051, -0.0331 | 25th Percentile = -0.0880 | 75th Percentile = 0.0100 | Mean difference: -0.0421. Note: Dashed vertical line indicates no difference in performance between compared approaches.


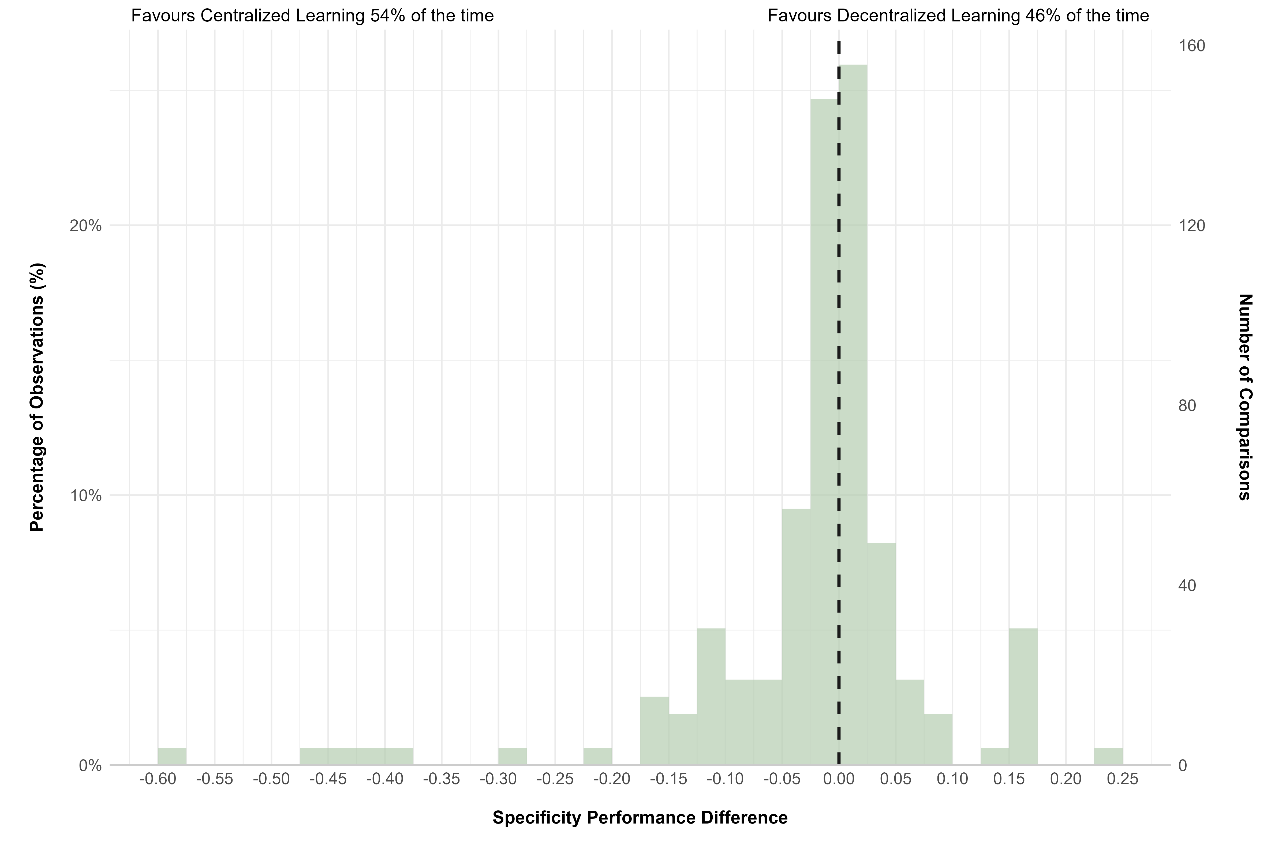


Supplementary Figure 8 - Distribution of Individual Model Performance Differences - Across Specificity (Comparing Decentralized Learning versus Centralized Learning). Based on 160 observations extracted from 46 models of 20 studies. Summary Results: Bootstrapped 95% CI: (-0.037, -0.0037 | 25th Percentile = -0.0295 | 75th Percentile = 0.0113 | Mean difference: -0.0198. Note: Dashed vertical line indicates no difference in performance between compared approaches.


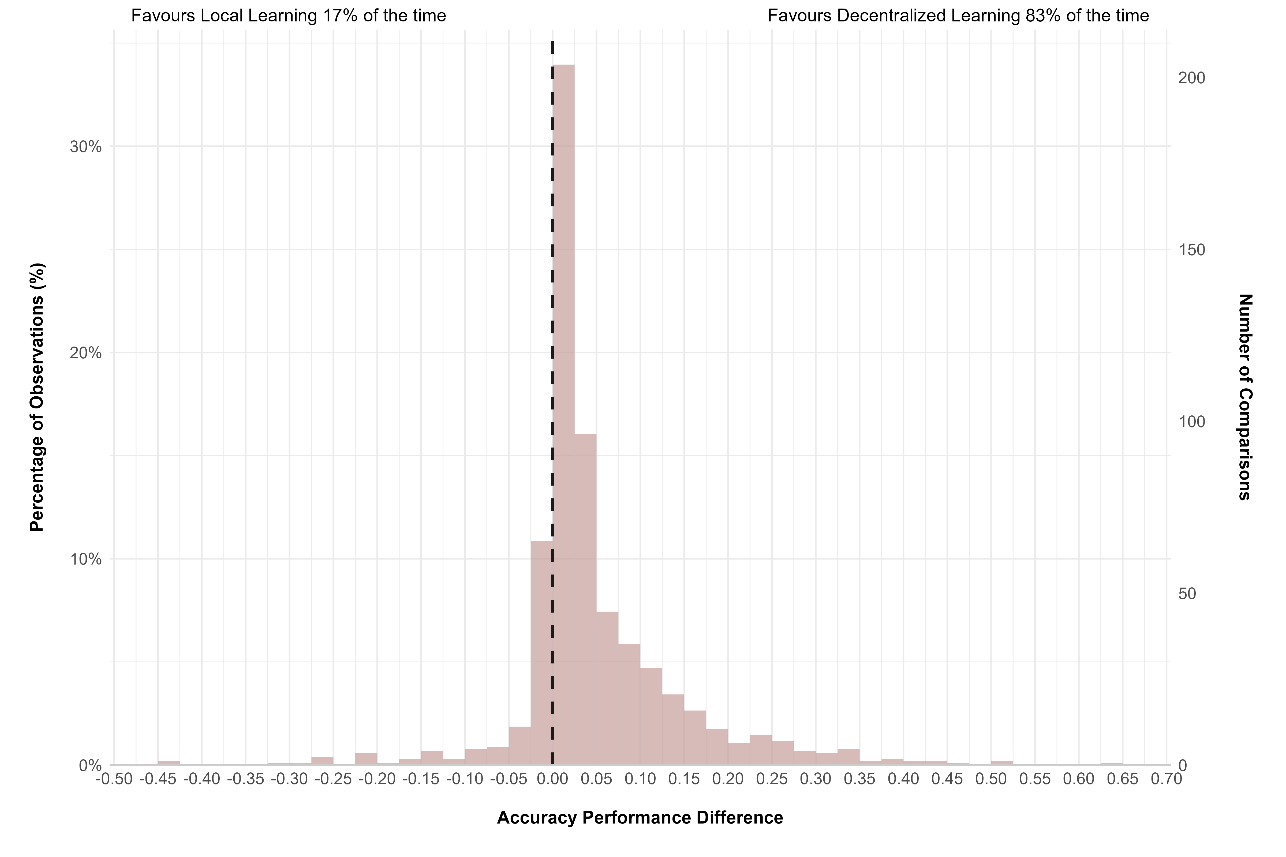


Supplementary Figure 9 - Distribution of Individual Model Performance Differences - Across Accuracy (Comparing Decentralized Learning versus Local Learning). Based on 1024 observations extracted from 139 models of 46 studies. Summary Results: Bootstrapped 95% CI: (0.0438, 0.0555 | 25th Percentile = 0.0042 | 75th Percentile = 0.0772 | Mean difference: 0.0497. Note: Dashed vertical line indicates no difference in performance between compared approaches.


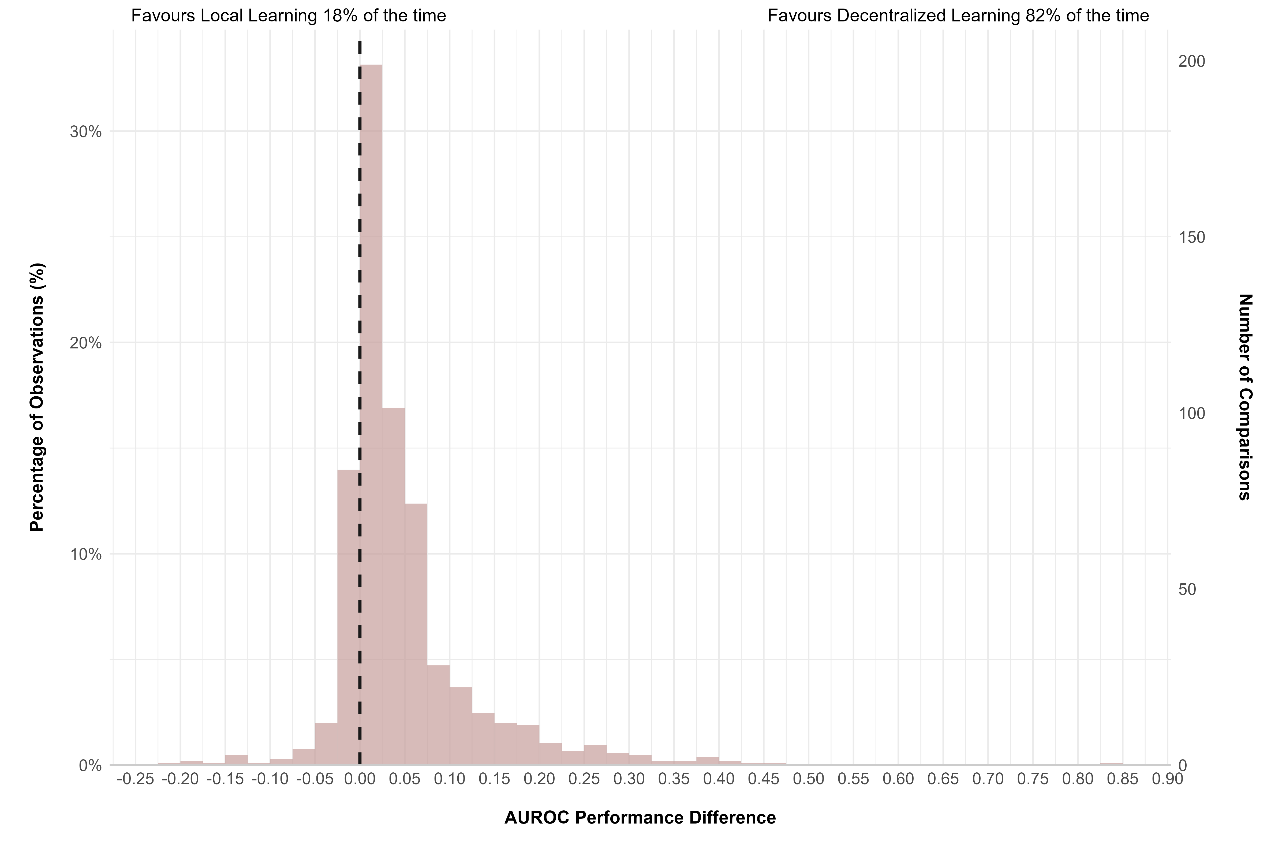


Supplementary Figure 10 - Distribution of Individual Model Performance Differences - Across AUROC (Comparing Decentralized Learning versus Local Learning). Based on 1059 observations extracted from 140 models of 39 studies. Summary Results: Bootstrapped 95% CI: (0.0419, 0.0515 | 25th Percentile = 0.0033 | 75th Percentile = 0.0608 | Mean difference: 0.0466. Note: Dashed vertical line indicates no difference in performance between compared approaches.


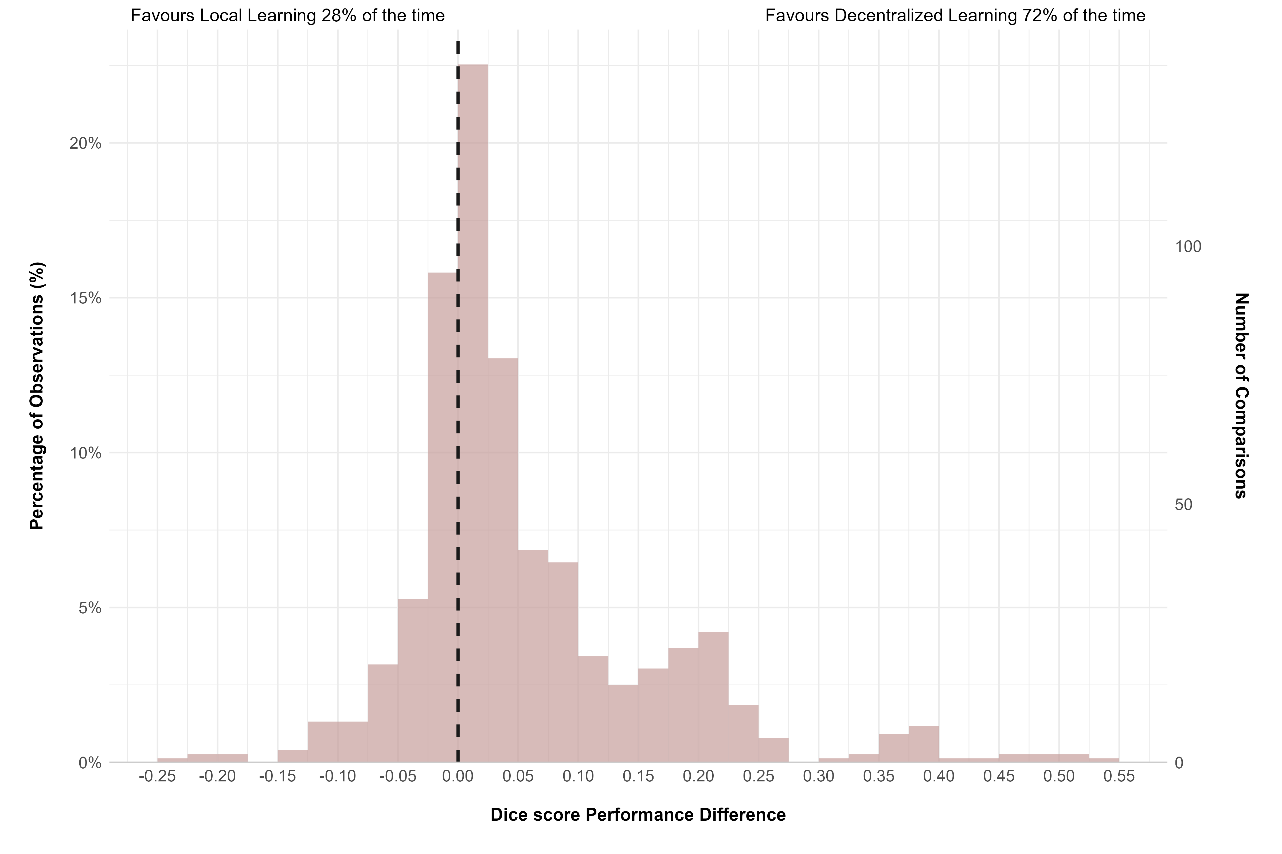


Supplementary Figure 11 - Distribution of Individual Model Performance Differences - Across Dice score (Comparing Decentralized Learning versus Local Learning). Based on 759 observations extracted from 74 models of 18 studies. Summary Results: Bootstrapped 95% CI: (0.0501, 0.0651 | 25th Percentile = -0.0020 | 75th Percentile = 0.0935 | Mean difference: 0.0573. Note: Dashed vertical line indicates no difference in performance between compared approaches.


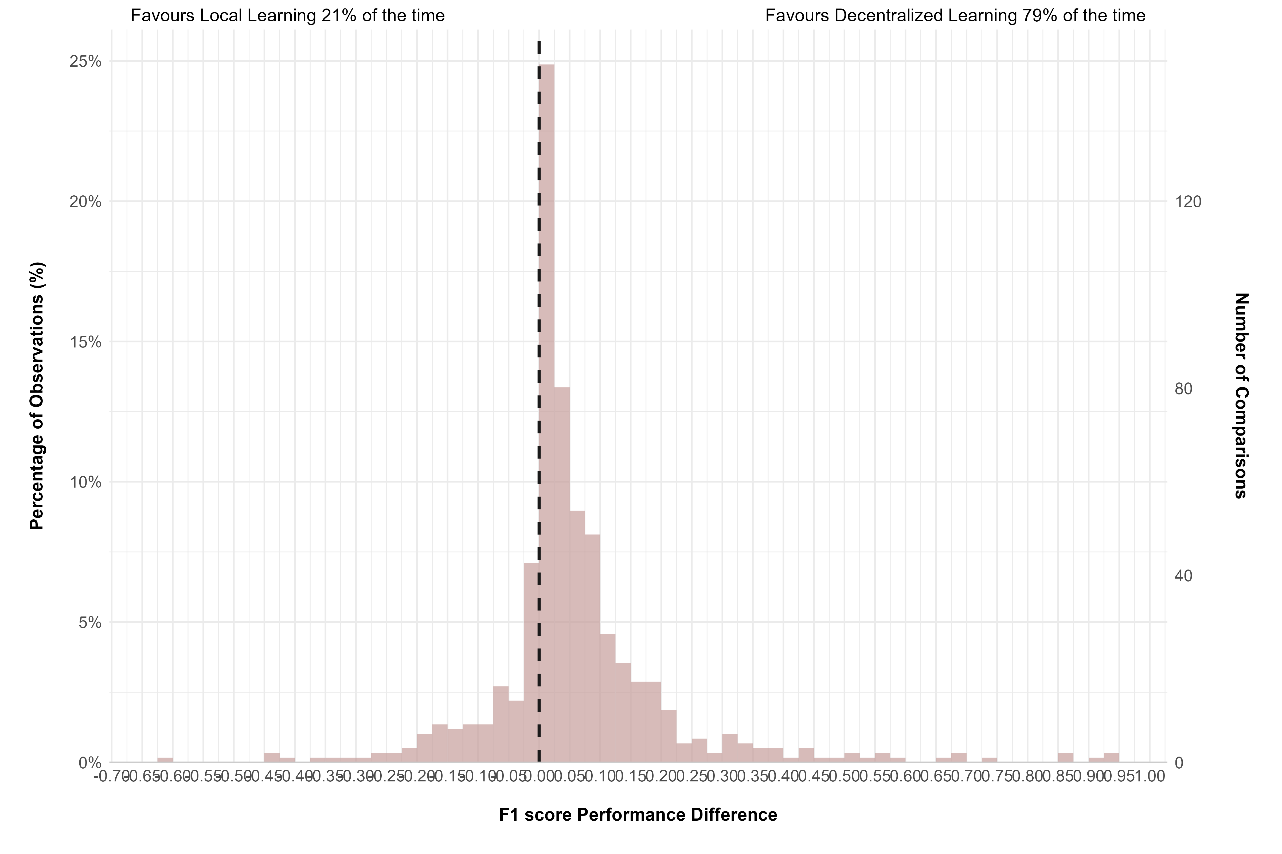


Supplementary Figure 12 - Distribution of Individual Model Performance Differences - Across F1 score (Comparing Decentralized Learning versus Local Learning). Based on 593 observations extracted from 74 models of 27 studies. Summary Results: Bootstrapped 95% CI: (0.0489, 0.0745 | 25th Percentile = 0.0040 | 75th Percentile = 0.0955 | Mean difference: 0.0614. Note: Dashed vertical line indicates no difference in performance between compared approaches.


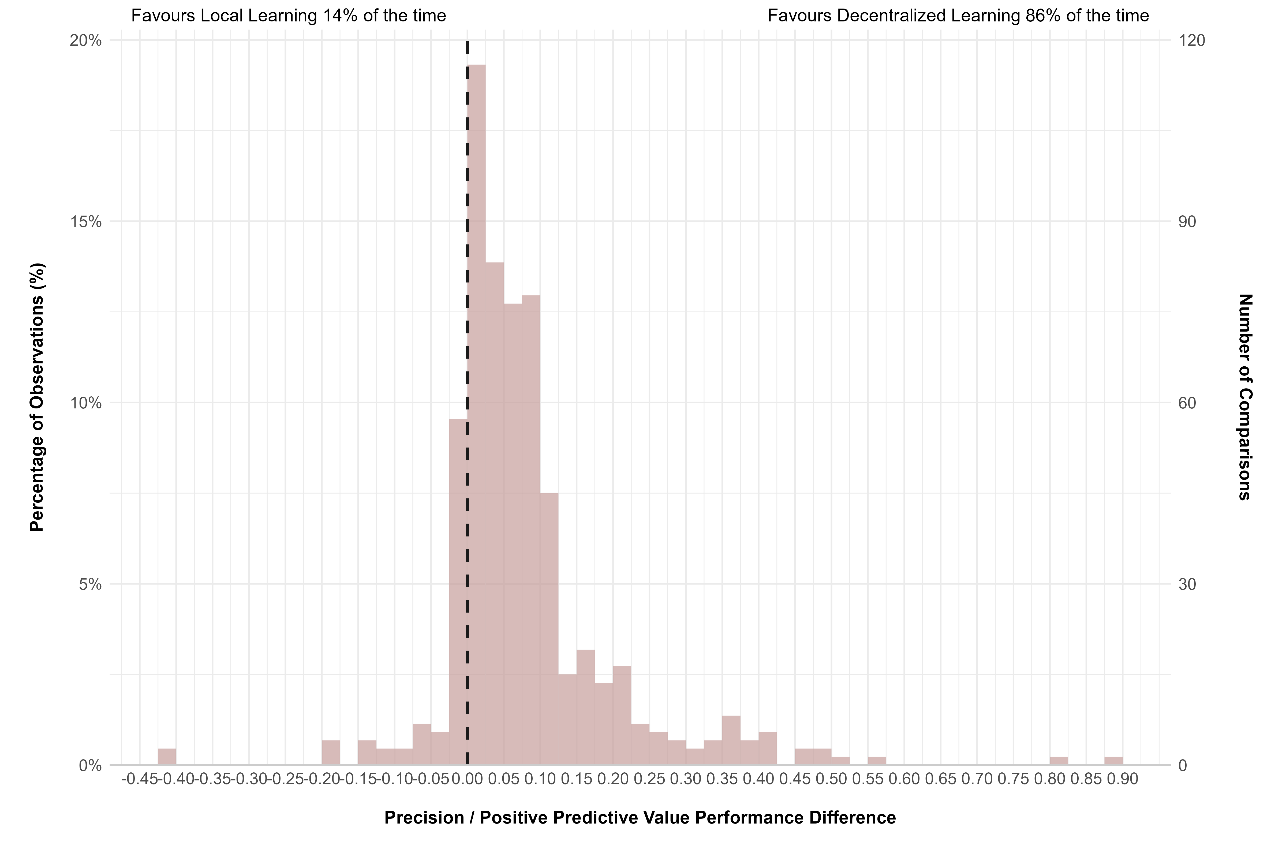


Supplementary Figure 13 - Distribution of Individual Model Performance Differences - Across Precision / Positive Predictive Value (Comparing Decentralized Learning versus Local Learning). Based on 442 observations extracted from 60 models of 23 studies. Summary Results: Bootstrapped 95% CI: (0.0689, 0.0918 | 25th Percentile = 0.0120 | 75th Percentile = 0.1090 | Mean difference: 0.0801. Note: Dashed vertical line indicates no difference in performance between compared approaches.


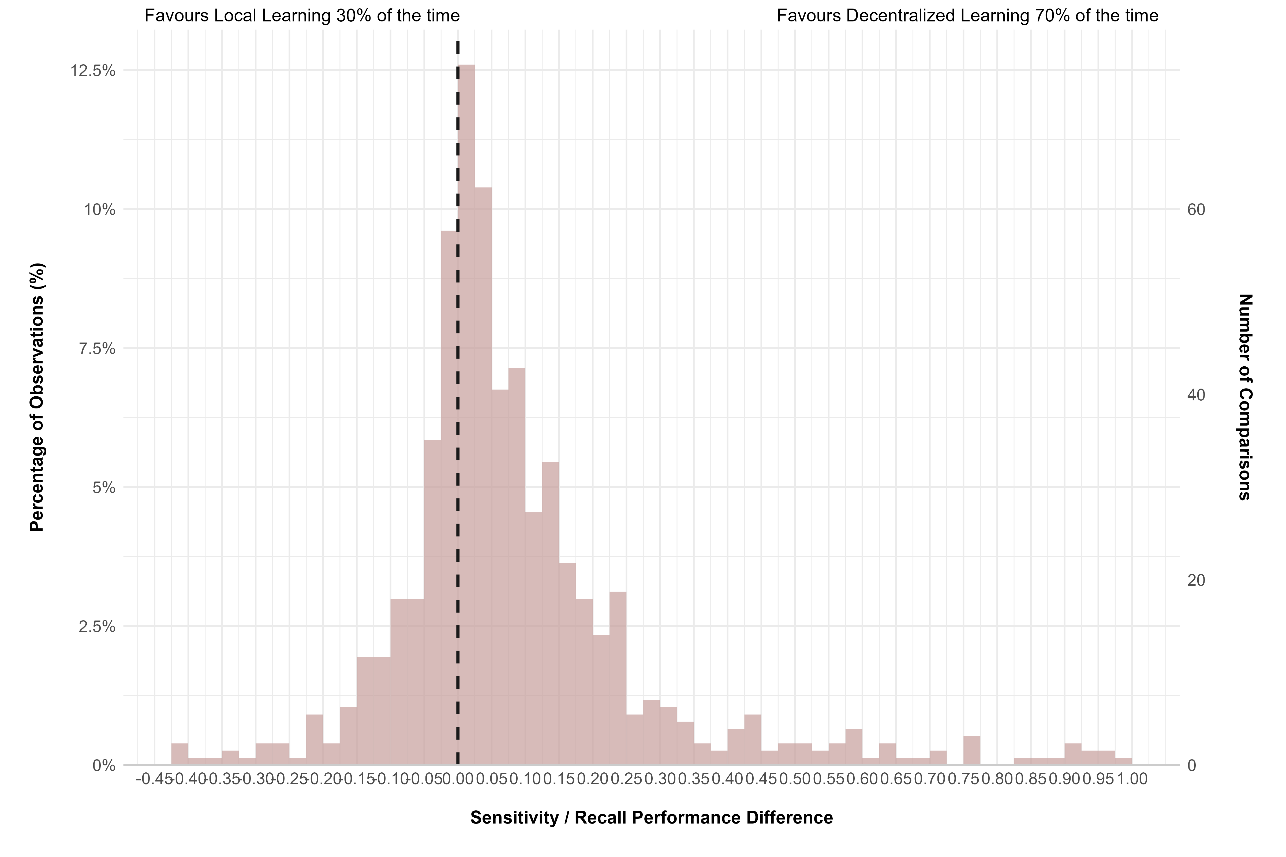


Supplementary Figure 14 - Distribution of Individual Model Performance Differences - Across Sensitivity / Recall (Comparing Decentralized Learning versus Local Learning). Based on 772 observations extracted from 96 models of 35 studies. Summary Results: Bootstrapped 95% CI: (0.0736, 0.1005 | 25th Percentile = -0.0093 | 75th Percentile = 0.1427 | Mean difference: 0.0869. Note: Dashed vertical line indicates no difference in performance between compared approaches.


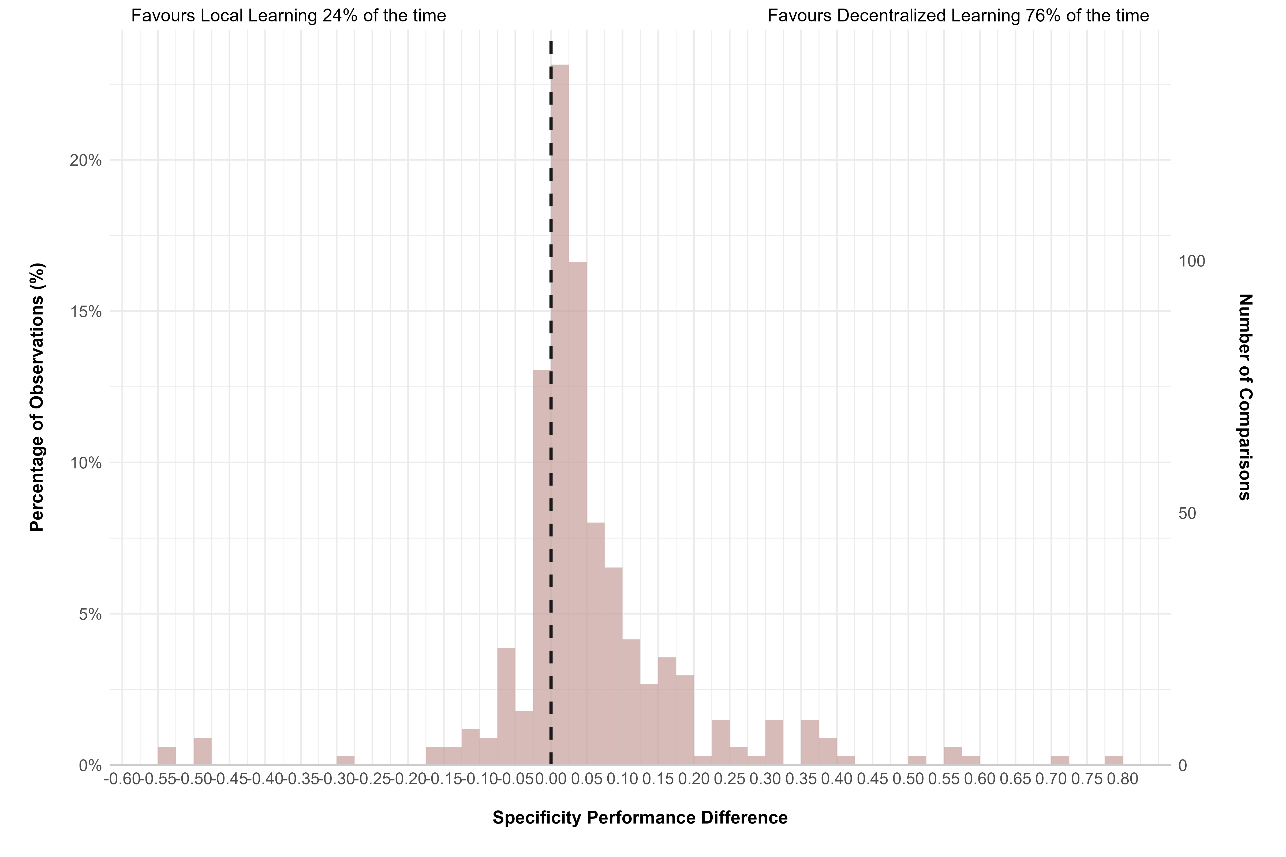


Supplementary Figure 15 - Distribution of Individual Model Performance Differences - Across Specificity (Comparing Decentralized Learning versus Local Learning). Based on 339 observations extracted from 47 models of 16 studies. Summary Results: Bootstrapped 95% CI: (0.0395, 0.0692 | 25th Percentile = 0.0021 | 75th Percentile = 0.0898 | Mean difference: 0.0541. Note: Dashed vertical line indicates no difference in performance between compared approaches.


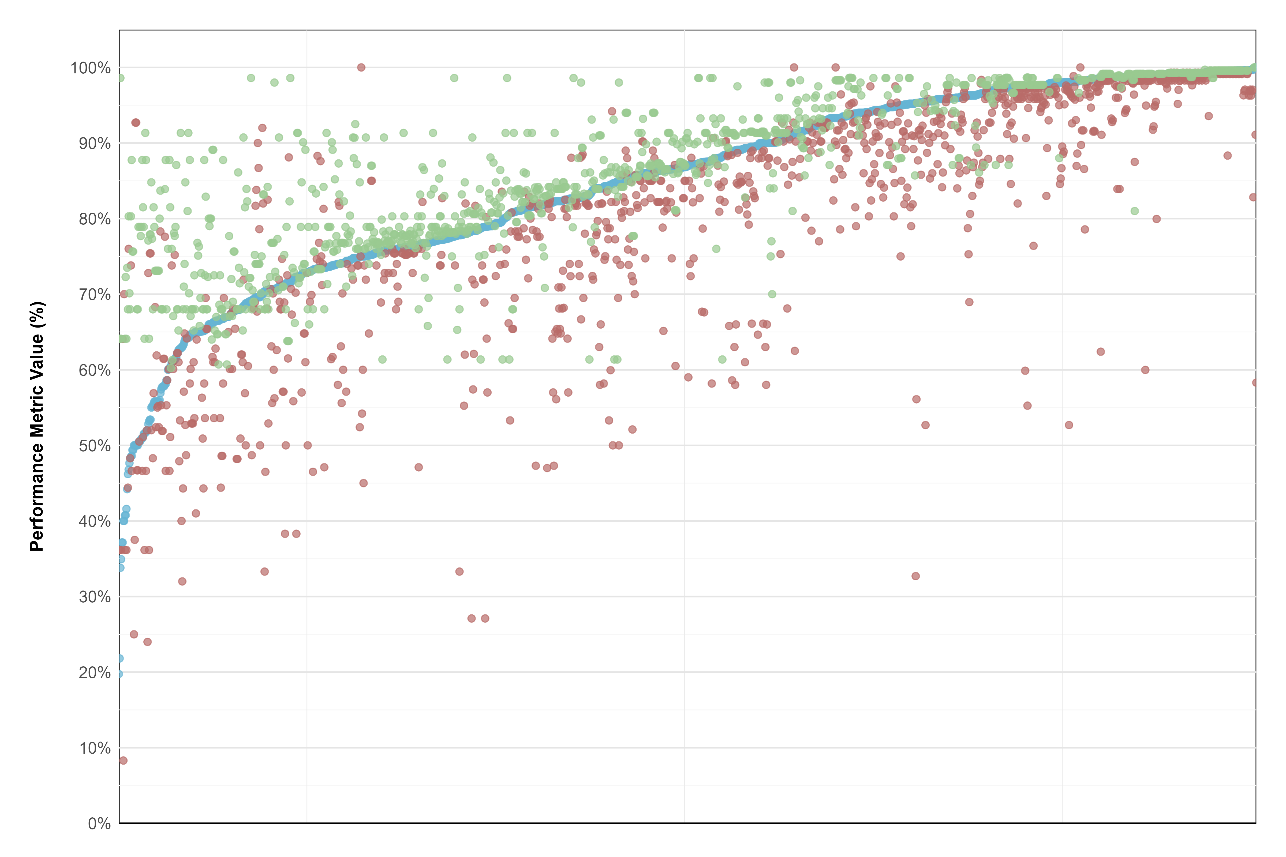


Supplementary Figure 16 - Aggregate Distribution of Individual Model Performance Differences - Across Accuracy (Comparing Centralized Learning versus Decentralized Learning versus Local Learning). Green dots, blue dots and red dots represent the performance of centralized models, decentralized models and local models, respectively. Green and red dots are vertically aligned with the corresponding blue dot of a specific performance comparison. Based on 1507 observations from 265 models and 86 studies. Note: Values ordered by the decentralized values of each comparison.


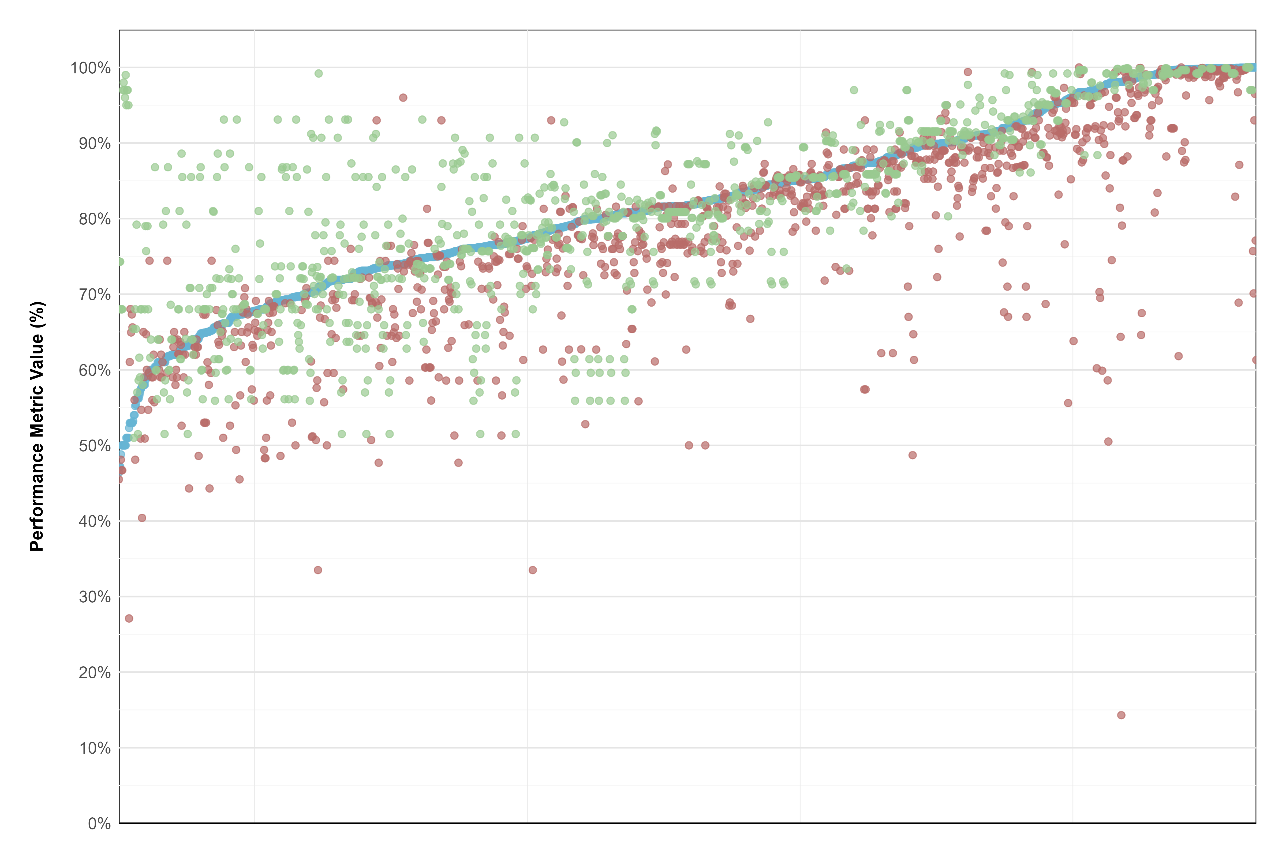


Supplementary Figure 17 - Aggregate Distribution of Individual Model Performance Differences - Across AUROC (Comparing Centralized Learning versus Decentralized Learning versus Local Learning). Green dots, blue dots and red dots represent the performance of centralized models, decentralized models and local models, respectively. Green and red dots are vertically aligned with the corresponding blue dot of a specific performance comparison. Based on 1669 observations from 266 models and 58 studies. Note: Values ordered by the decentralized values of each comparison.


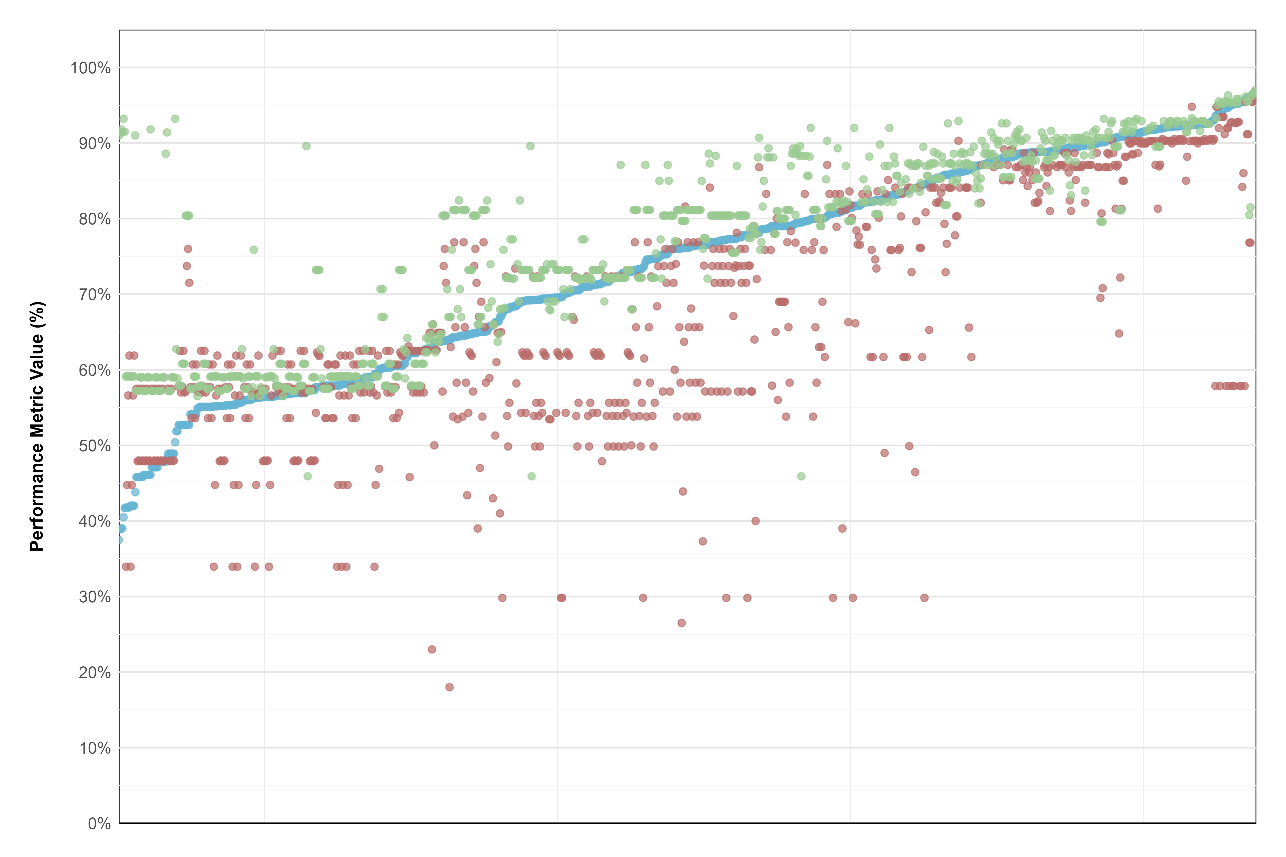


Supplementary Figure 18 - Aggregate Distribution of Individual Model Performance Differences - Across Dice score (Comparing Centralized Learning versus Decentralized Learning versus Local Learning). Green dots, blue dots and red dots represent the performance of centralized models, decentralized models and local models, respectively. Green and red dots are vertically aligned with the corresponding blue dot of a specific performance comparison. Based on 971 observations from 149 models and 29 studies. Note: Values ordered by the decentralized values of each comparison.


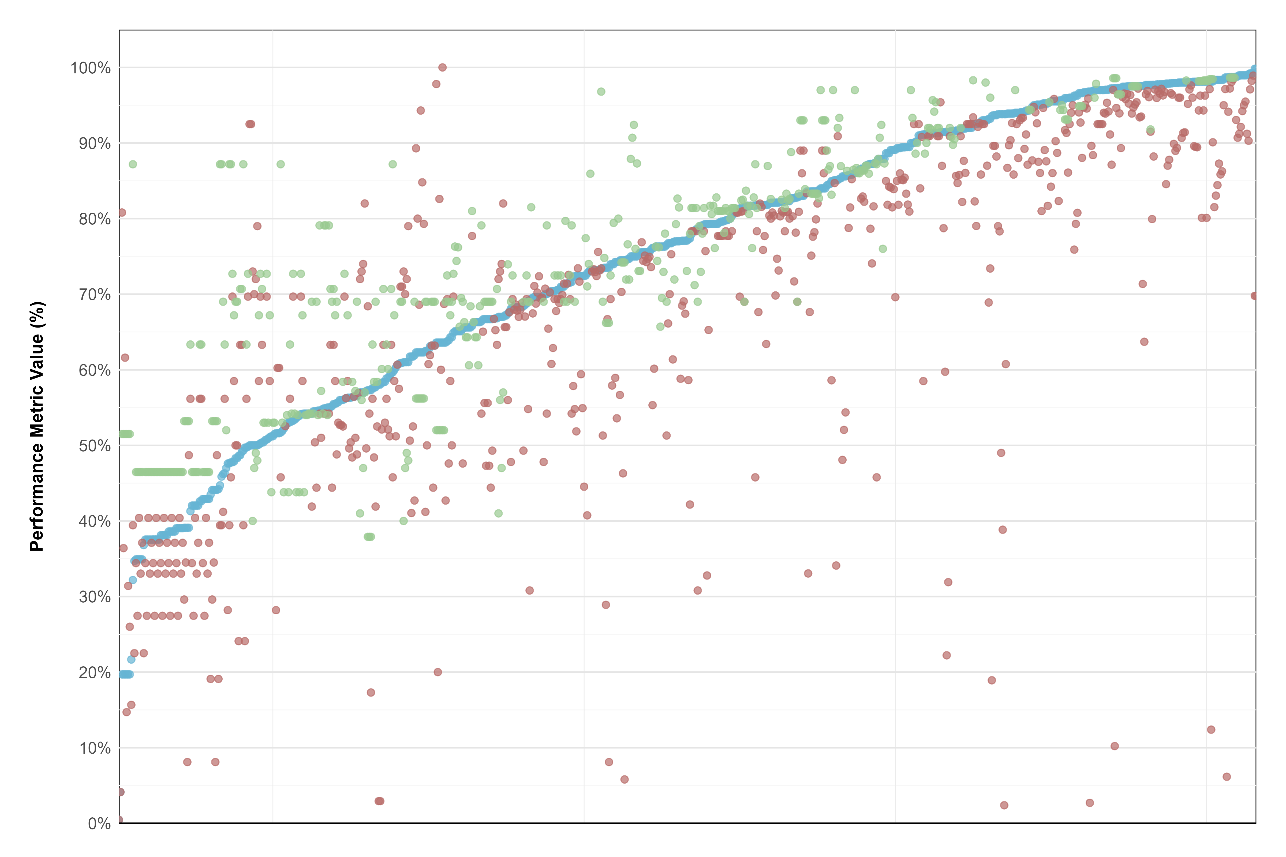


Supplementary Figure 19 - Aggregate Distribution of Individual Model Performance Differences - Across F1 score (Comparing Centralized Learning versus Decentralized Learning versus Local Learning). Green dots, blue dots and red dots represent the performance of centralized models, decentralized models and local models, respectively. Green and red dots are vertically aligned with the corresponding blue dot of a specific performance comparison. Based on 732 observations from 119 models and 44 studies. Note: Values ordered by the decentralized values of each comparison.


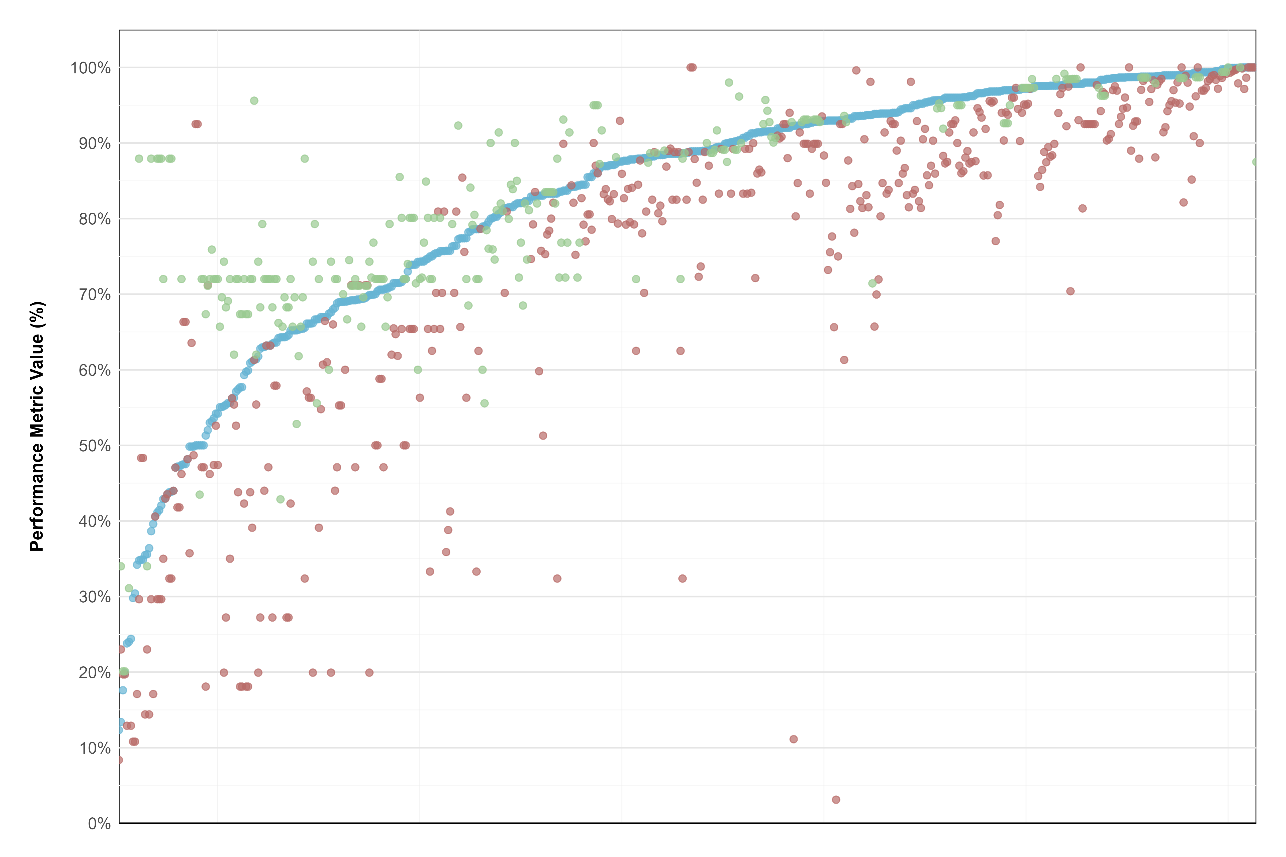


Supplementary Figure 20 - Aggregate Distribution of Individual Model Performance Differences - Across Precision / Positive Predictive Value (Comparing Centralized Learning versus Decentralized Learning versus Local Learning). Green dots, blue dots and red dots represent the performance of centralized models, decentralized models and local models, respectively. Green and red dots are vertically aligned with the corresponding blue dot of a specific performance comparison. Based on 564 observations from 108 models and 42 studies. Note: Values ordered by the decentralized values of each comparison.


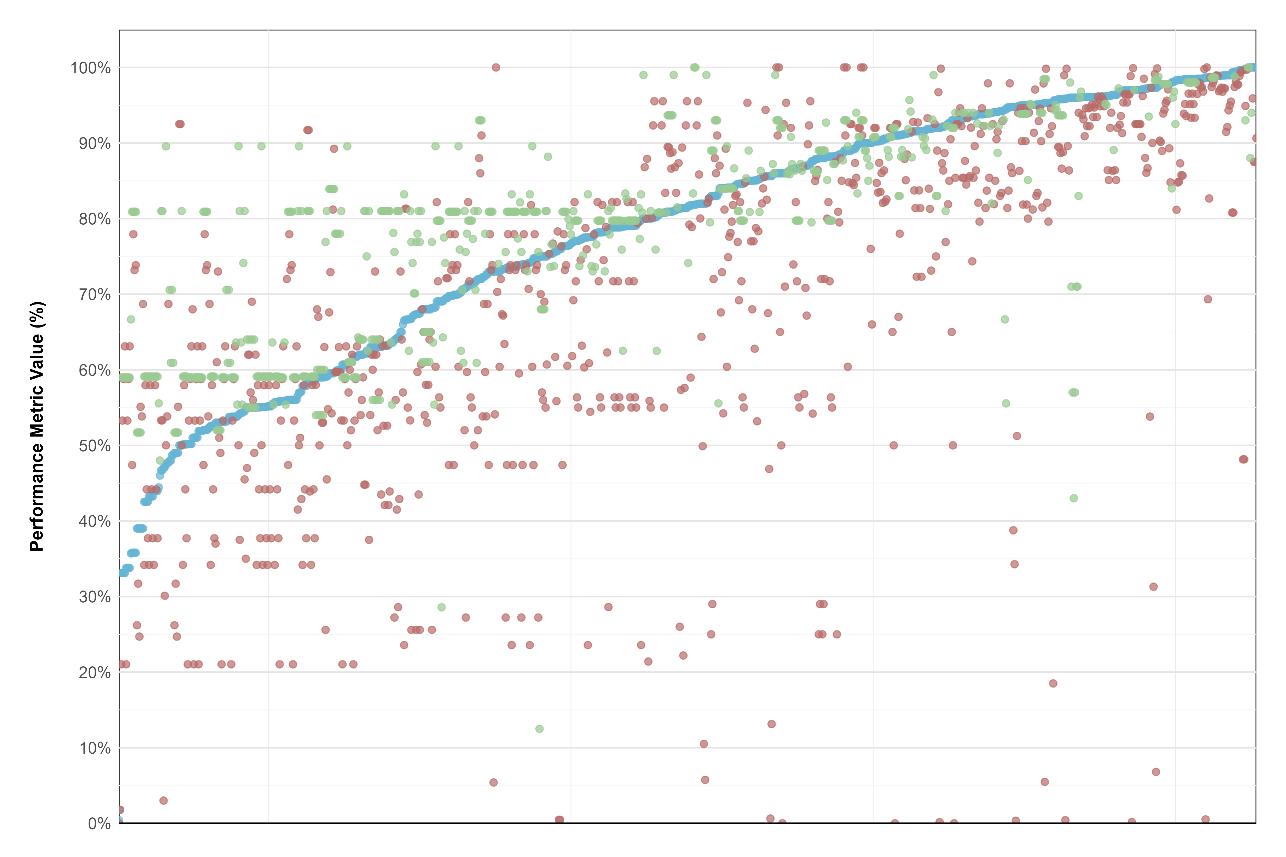


Supplementary Figure 21 - Aggregate Distribution of Individual Model Performance Differences - Across Sensitivity / Recall (Comparing Centralized Learning versus Decentralized Learning versus Local Learning). Green dots, blue dots and red dots represent the performance of centralized models, decentralized models and local models, respectively. Green and red dots are vertically aligned with the corresponding blue dot of a specific performance comparison. Based on 942 observations from 165 models and 60 studies. Note: Values ordered by the decentralized values of each comparison.


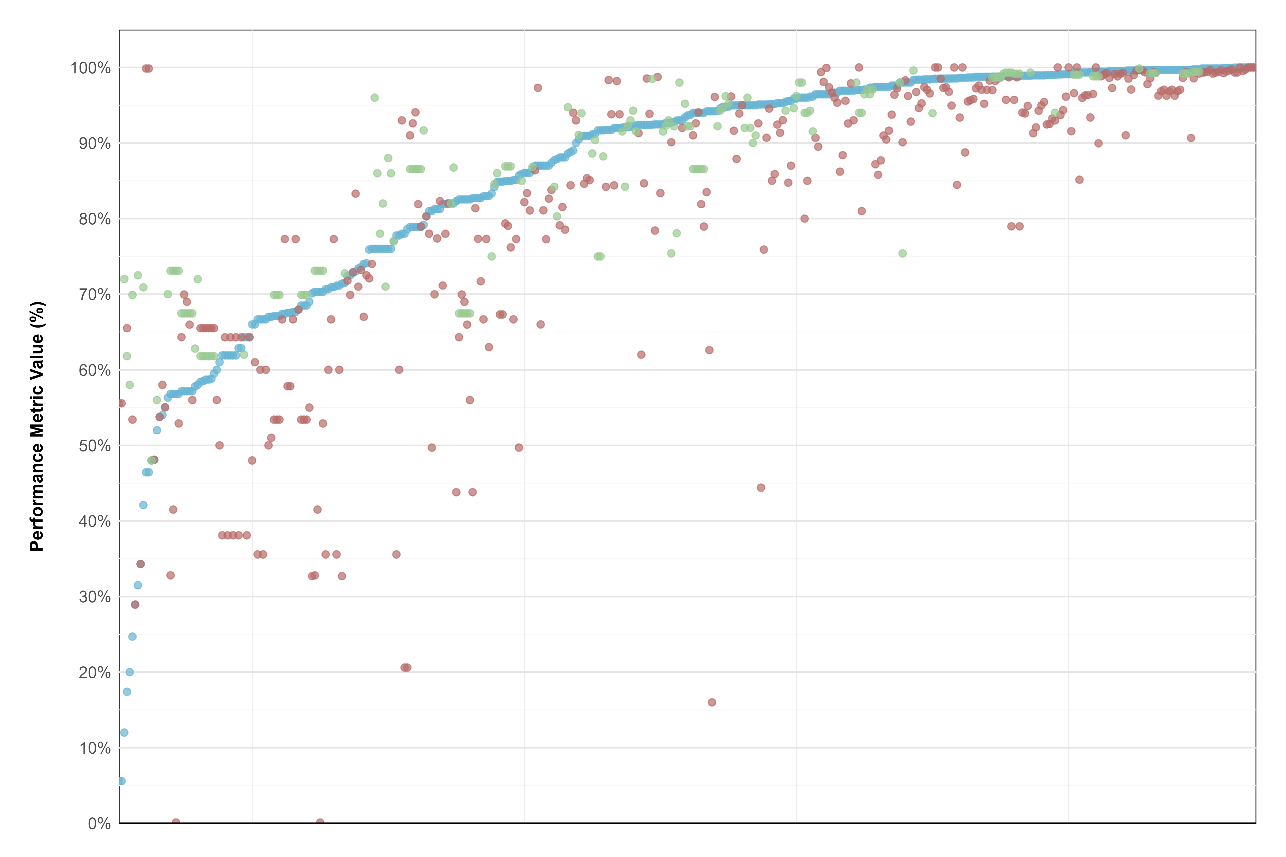


Supplementary Figure 22 - Aggregate Distribution of Individual Model Performance Differences - Across Specificity (Comparing Centralized Learning versus Decentralized Learning versus Local Learning). Green dots, blue dots and red dots represent the performance of centralized models, decentralized models and local models, respectively. Green and red dots are vertically aligned with the corresponding blue dot of a specific performance comparison. Based on 419 observations from 77 models and 30 studies. Note: Values ordered by the decentralized values of each comparison.

## Performance Analyses with Type of Data Origin

To facilitate the distinction between primary and secondary data collection, we added different graphical representations of some of the presented analyses. In particular, labelling sections in the existing histograms and points on plots to further detail their origin. Summary statistics were also added to quantify the percentage of each data collection type in a given analysis.


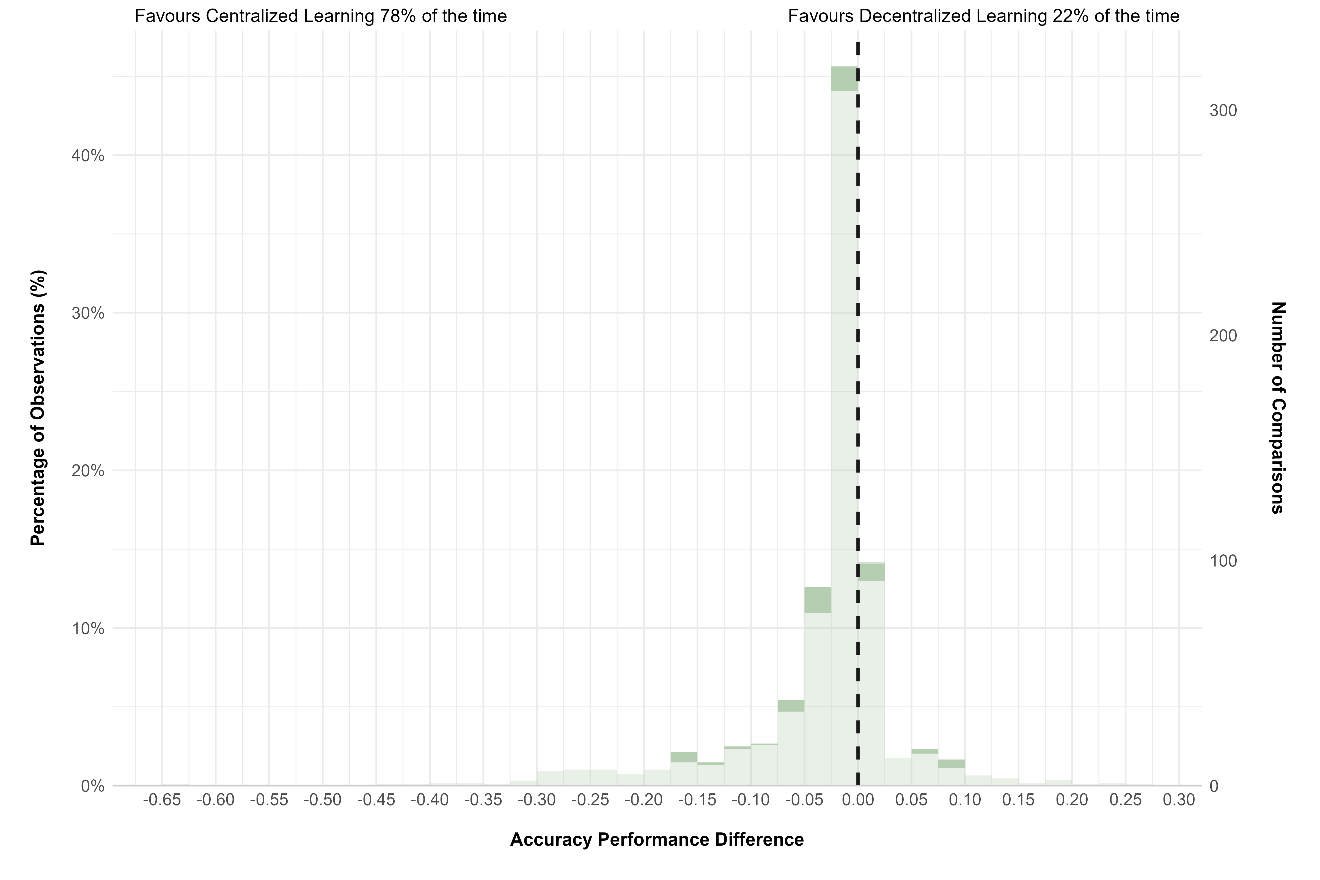


Supplementary Figure 23 - Distribution of Individual Model Performance Differences - Across Accuracy (Comparing Decentralized Learning versus Centralized Learning), by nature of data collection. Dark green, very light green and light green represent models using Primary data collection, Secondary data collection or Both, respectively. Based on 1089 observations extracted from 212 models of 66 studies. Summary Results: 76 comparisons based on Primary data collection | 25th Percentile = -0.0364 | 75th Percentile = 0.0000. Note: Dashed vertical line indicates no difference in performance between compared approaches.


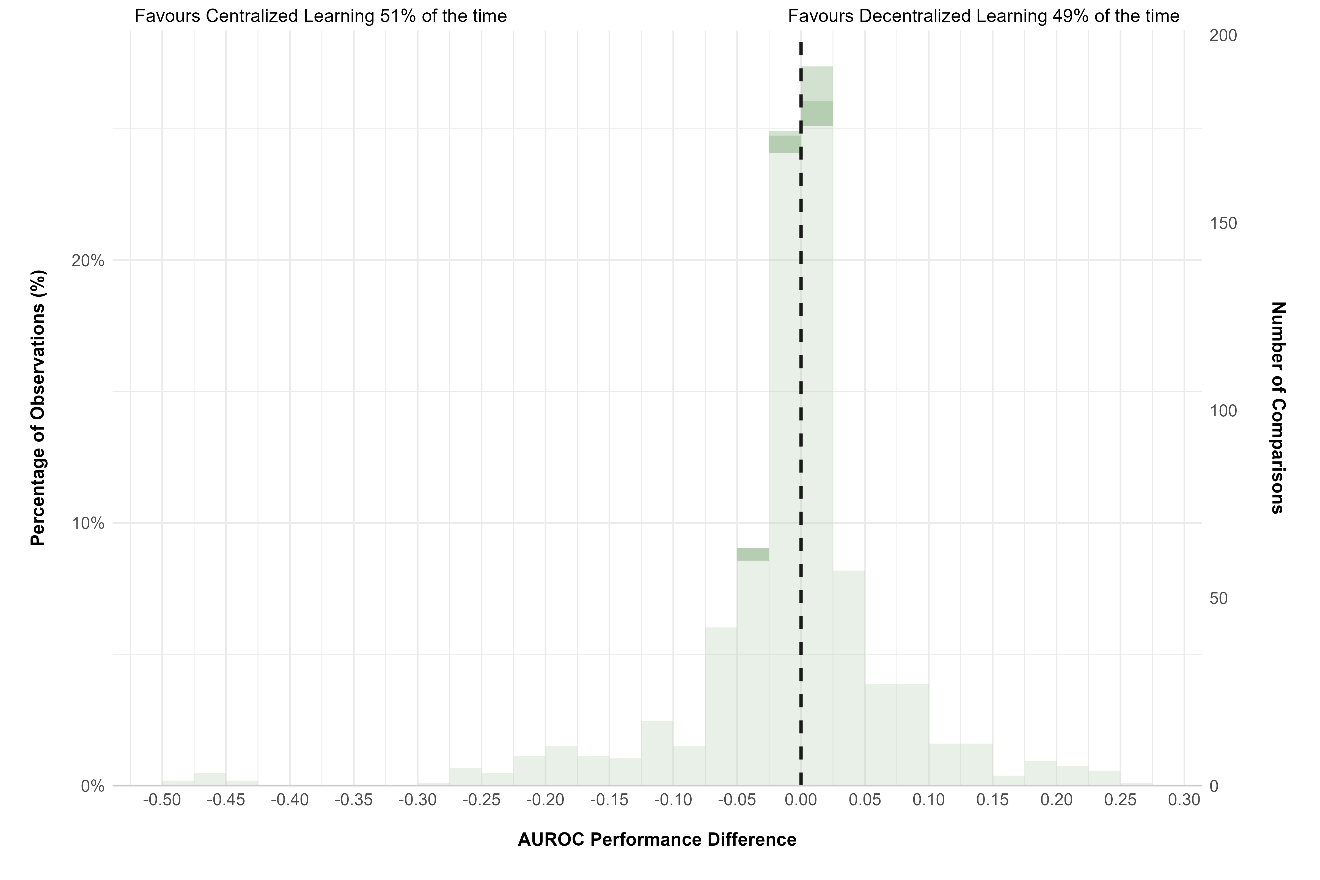


Supplementary Figure 24 - Distribution of Individual Model Performance Differences - Across AUROC (Comparing Decentralized Learning versus Centralized Learning), by nature of data collection. Dark green, very light green and light green represent models using Primary data collection, Secondary data collection or Both, respectively. Based on 1063 observations extracted from 212 models of 42 studies. Summary Results: 22 comparisons based on Primary data collection | 25th Percentile = -0.0287 | 75th Percentile = 0.0190. Note: Dashed vertical line indicates no difference in performance between compared approaches.


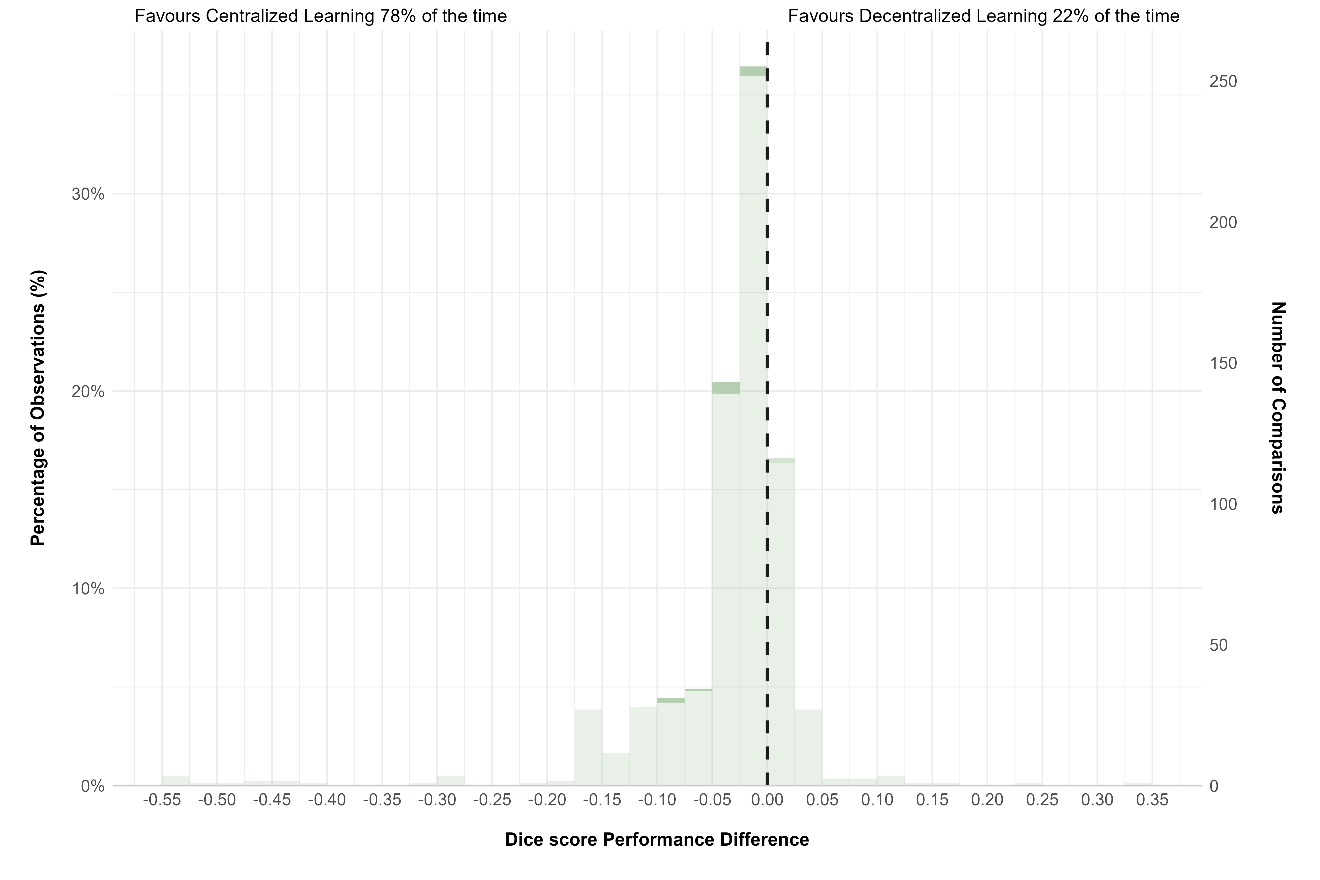


Supplementary Figure 25 - Distribution of Individual Model Performance Differences - Across Dice score (Comparing Decentralized Learning versus Centralized Learning), by nature of data collection. Dark green, very light green and light green represent models using Primary data collection, Secondary data collection or Both, respectively. Based on 856 observations extracted from 127 models of 24 studies. Summary Results: 12 comparisons based on Primary data collection | 25th Percentile = -0.0411 | 75th Percentile = -0.0021. Note: Dashed vertical line indicates no difference in performance between compared approaches.


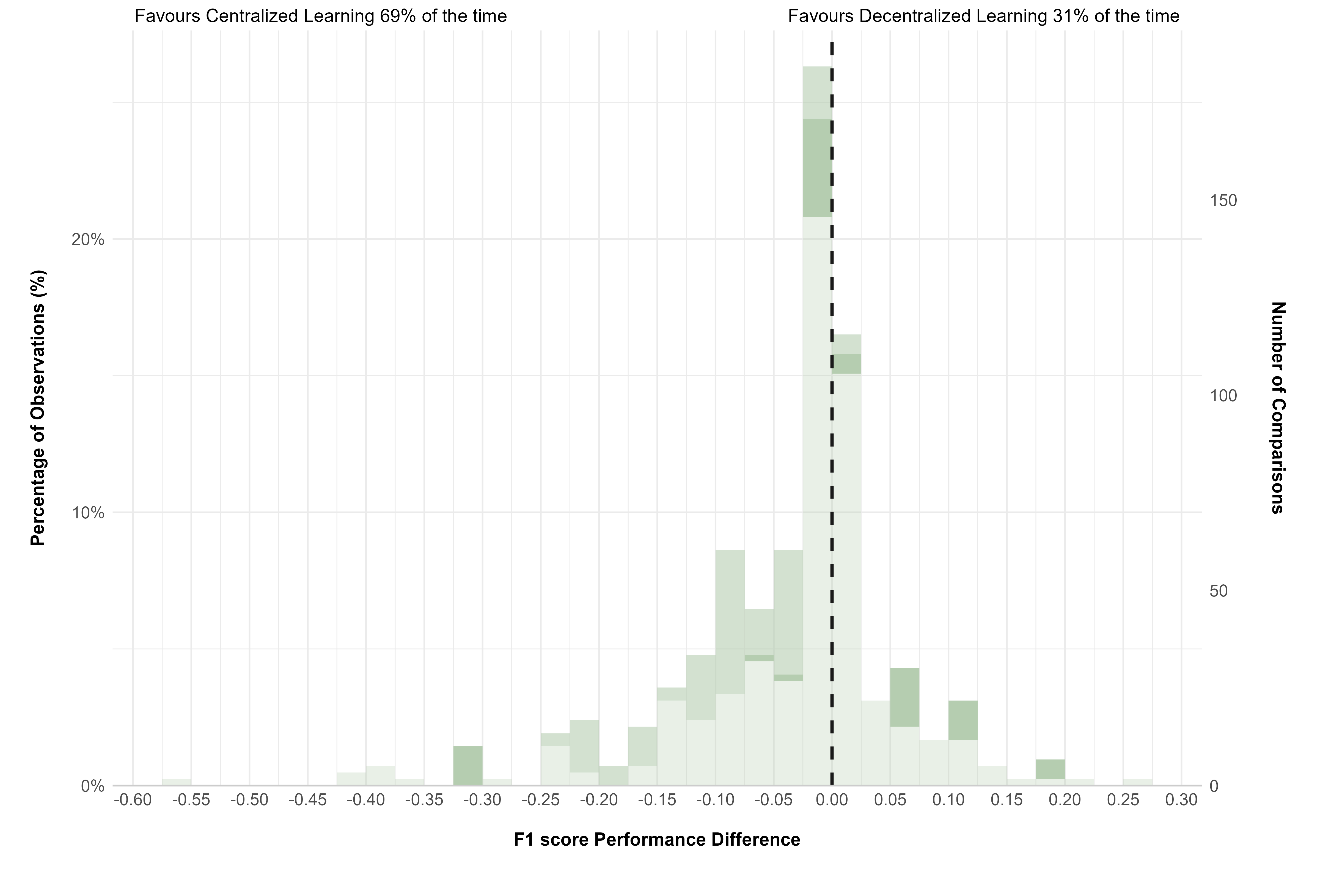


Supplementary Figure 26 - Distribution of Individual Model Performance Differences - Across F1 score (Comparing Decentralized Learning versus Centralized Learning), by nature of data collection. Dark green, very light green and light green represent models using Primary data collection, Secondary data collection or Both, respectively. Based on 420 observations extracted from 98 models of 32 studies. Summary Results: 44 comparisons based on Primary data collection | 25th Percentile = -0.0825 | 75th Percentile = 0.0034. Note: Dashed vertical line indicates no difference in performance between compared approaches.


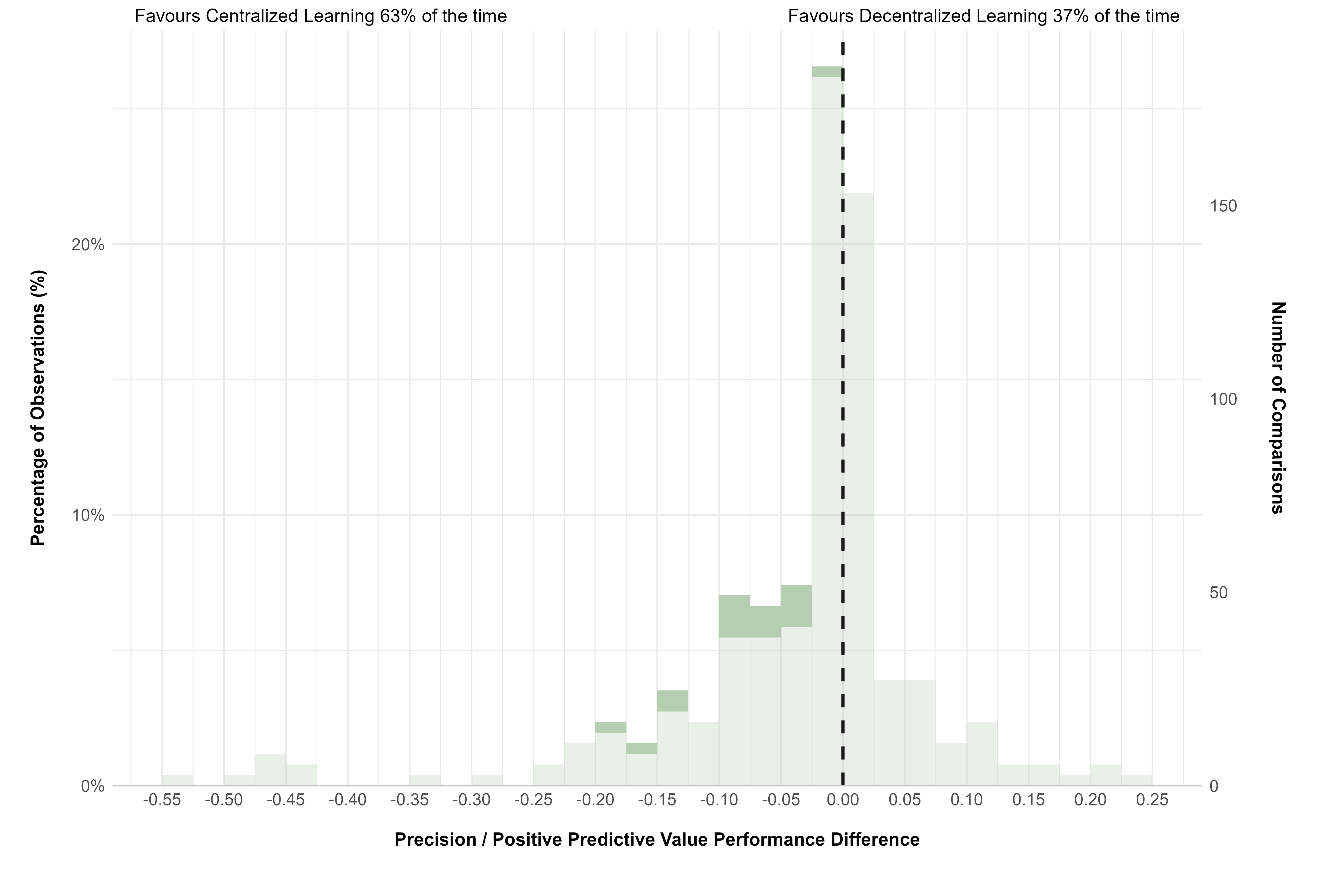


Supplementary Figure 27 - Distribution of Individual Model Performance Differences - Across Precision / Positive Predictive Value (Comparing Decentralized Learning versus Centralized Learning), by nature of data collection. Dark green, very light green and light green represent models using Primary data collection, Secondary data collection or Both, respectively. Based on 258 observations extracted from 80 models of 29 studies. Summary Results: 16 comparisons based on Primary data collection | 25th Percentile = -0.0640 | 75th Percentile = 0.0080. Note: Dashed vertical line indicates no difference in performance between compared approaches.


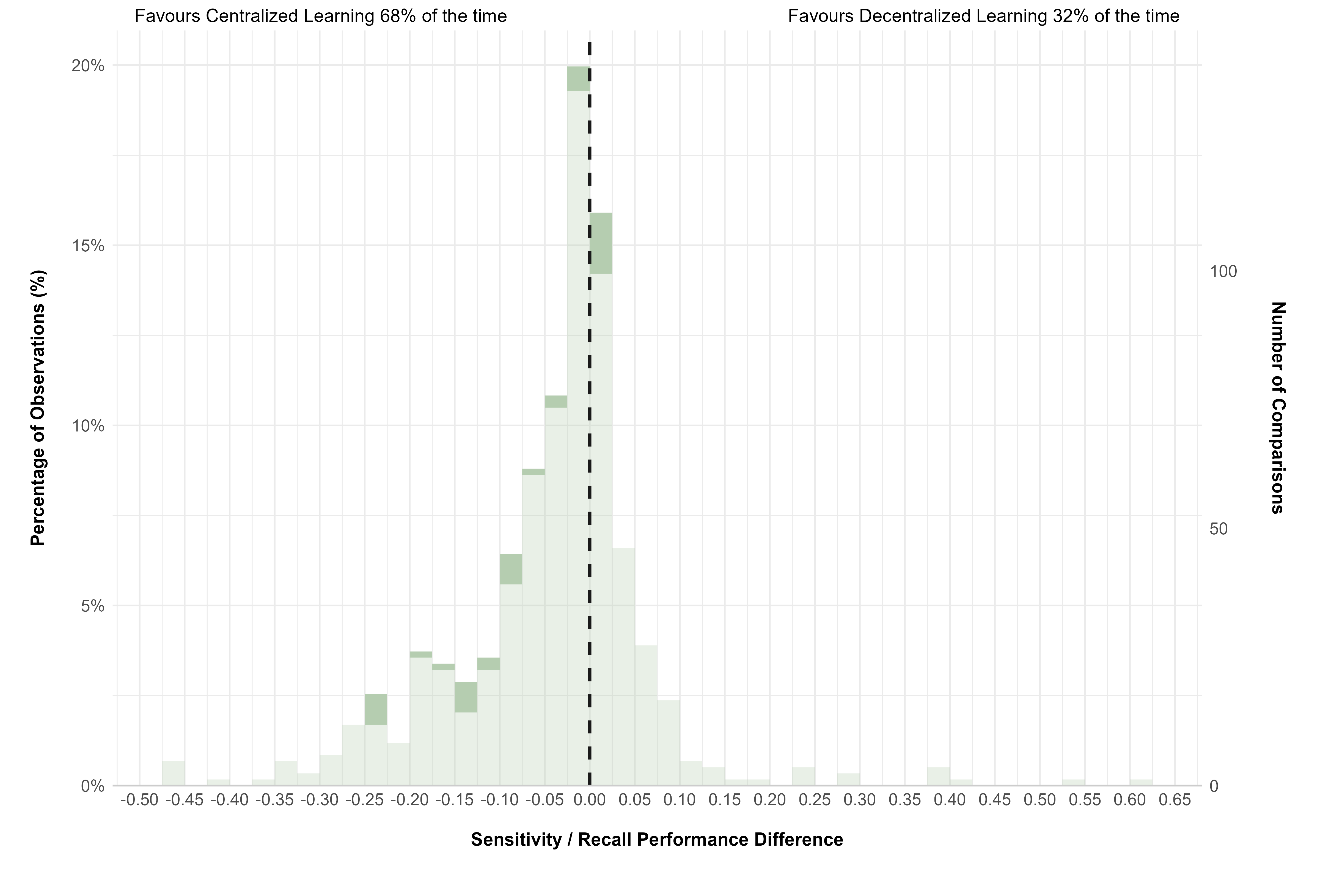


Supplementary Figure 28 - Distribution of Individual Model Performance Differences - Across Sensitivity / Recall (Comparing Decentralized Learning versus Centralized Learning), by nature of data collection. Dark green, very light green and light green represent models using Primary data collection, Secondary data collection or Both, respectively. Based on 593 observations extracted from 120 models of 42 studies. Summary Results: 36 comparisons based on Primary data collection | 25th Percentile = -0.0880 | 75th Percentile = 0.0100. Note: Dashed vertical line indicates no difference in performance between compared approaches.


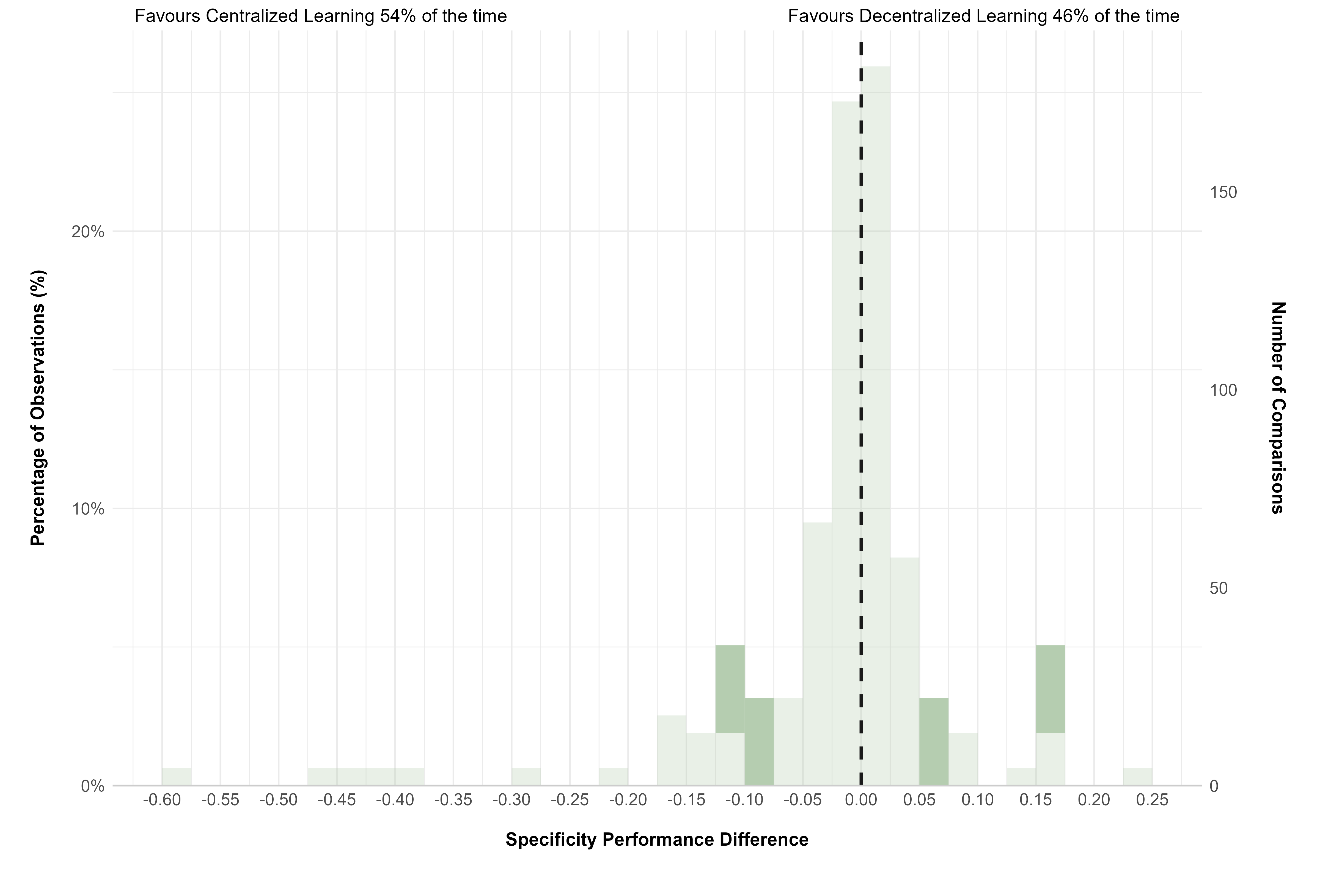


Supplementary Figure 29 - Distribution of Individual Model Performance Differences - Across Specificity (Comparing Decentralized Learning versus Centralized Learning), by nature of data collection. Dark green, very light green and light green represent models using Primary data collection, Secondary data collection or Both, respectively. Based on 160 observations extracted from 46 models of 20 studies. Summary Results: 20 comparisons based on Primary data collection | 25th Percentile = -0.0295 | 75th Percentile = 0.0113. Note: Dashed vertical line indicates no difference in performance between compared approaches.


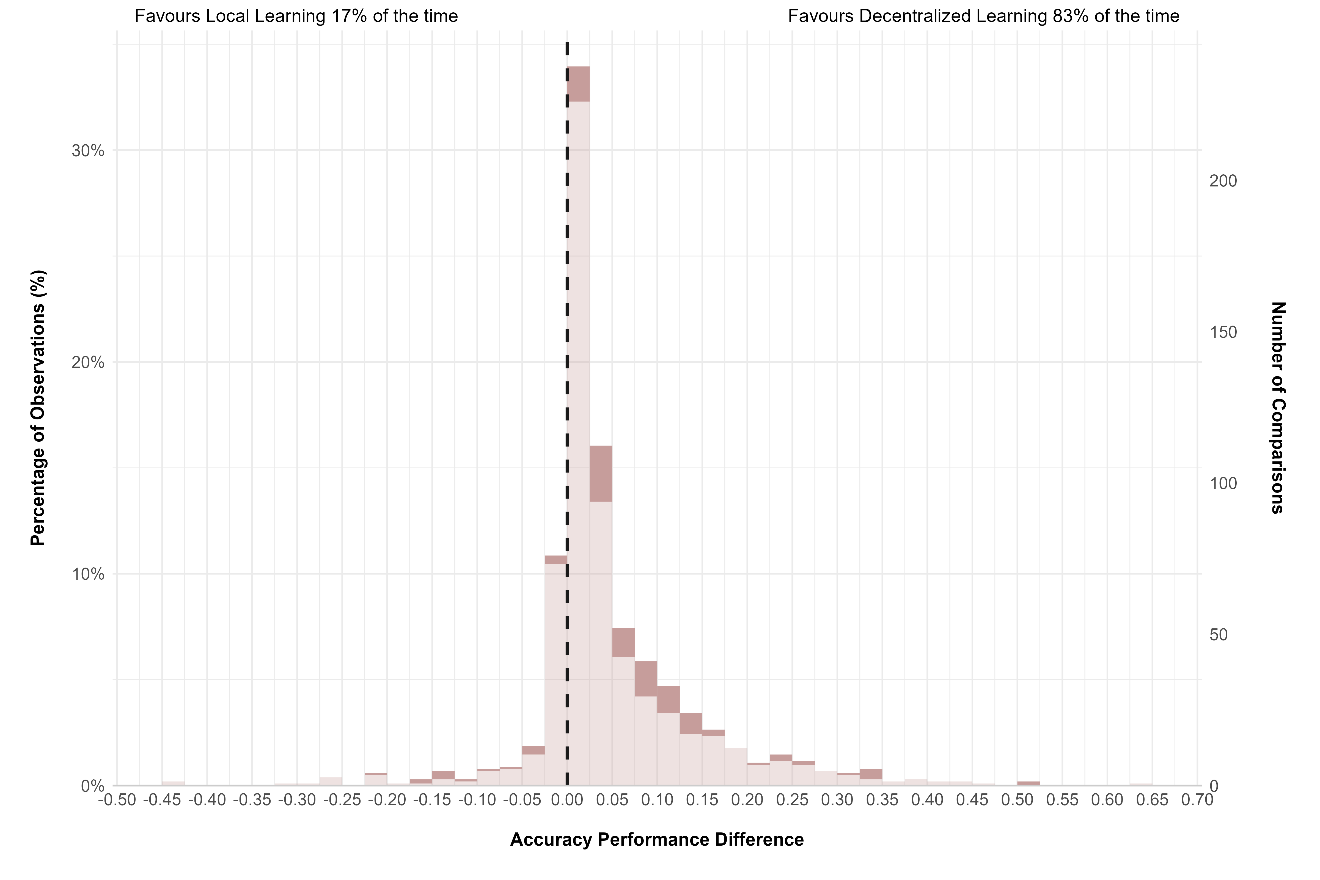


Supplementary Figure 30 - Distribution of Individual Model Performance Differences - Across Accuracy (Comparing Decentralized Learning versus Local Learning), by nature of data collection. Dark green, very light green and light green represent models using Primary data collection, Secondary data collection or Both, respectively. Based on 1024 observations extracted from 139 models of 46 studies. Summary Results: 133 comparisons based on Primary data collection | 25th Percentile = 0.0042 | 75th Percentile = 0.0772. Note: Dashed vertical line indicates no difference in performance between compared approaches.


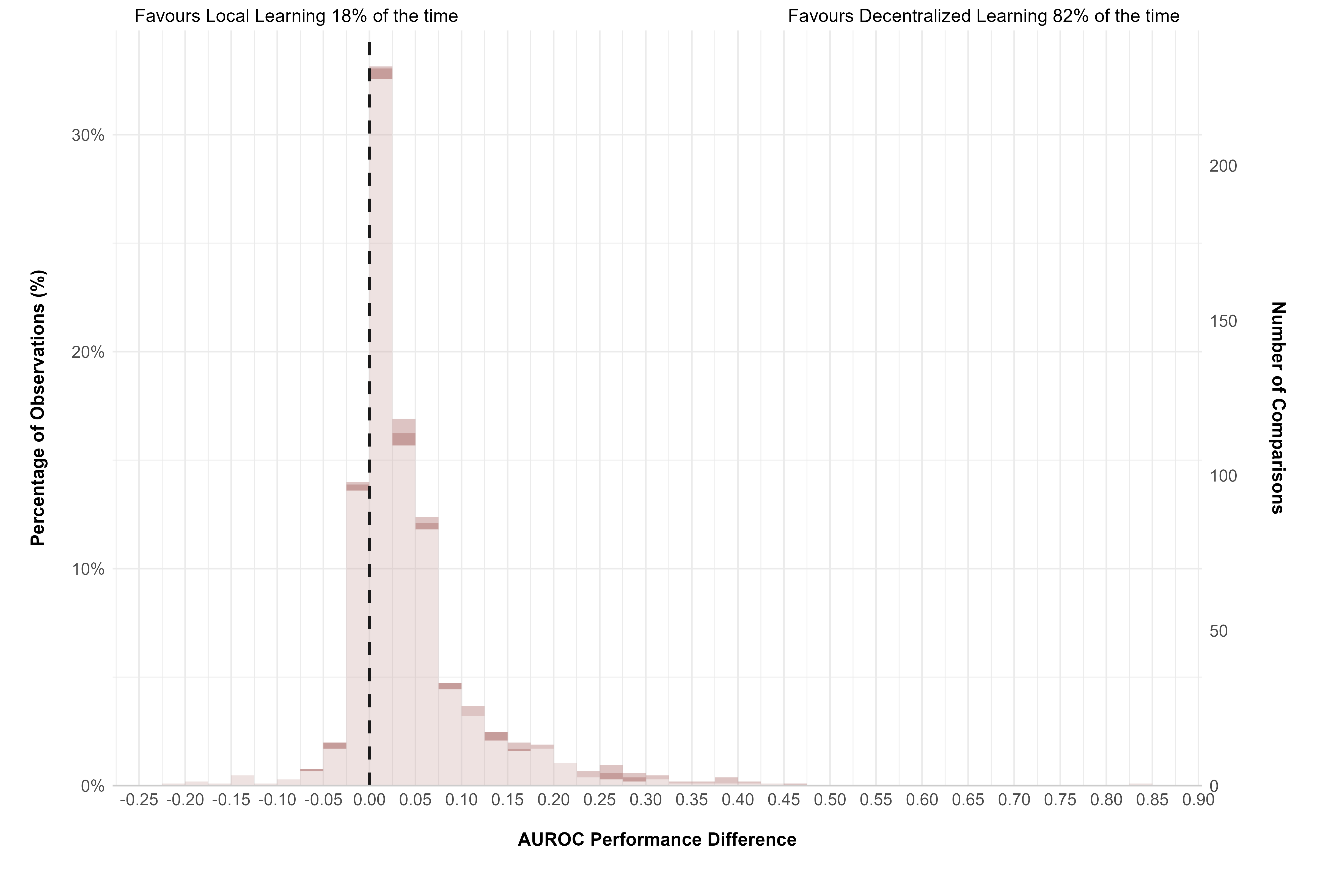


Supplementary Figure 31 - Distribution of Individual Model Performance Differences - Across AUROC (Comparing Decentralized Learning versus Local Learning), by nature of data collection. Dark green, very light green and light green represent models using Primary data collection, Secondary data collection or Both, respectively. Based on 1059 observations extracted from 140 models of 39 studies. Summary Results: 34 comparisons based on Primary data collection | 25th Percentile = 0.0033 | 75th Percentile = 0.0608. Note: Dashed vertical line indicates no difference in performance between compared approaches.


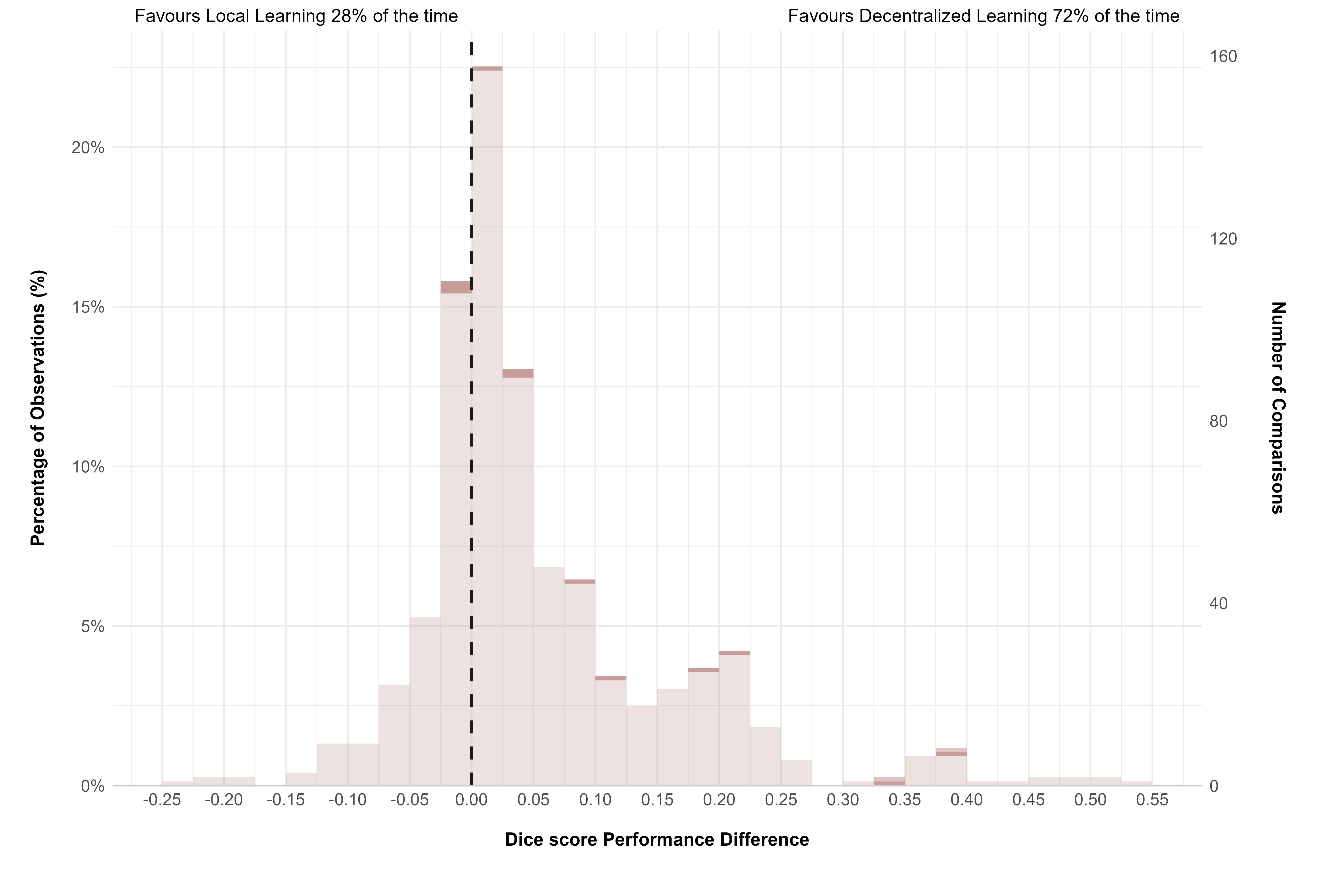


Supplementary Figure 32 - Distribution of Individual Model Performance Differences - Across Dice score (Comparing Decentralized Learning versus Local Learning), by nature of data collection. Dark green, very light green and light green represent models using Primary data collection, Secondary data collection or Both, respectively. Based on 759 observations extracted from 74 models of 18 studies. Summary Results: 12 comparisons based on Primary data collection | 25th Percentile = -0.0020 | 75th Percentile = 0.0935. Note: Dashed vertical line indicates no difference in performance between compared approaches.


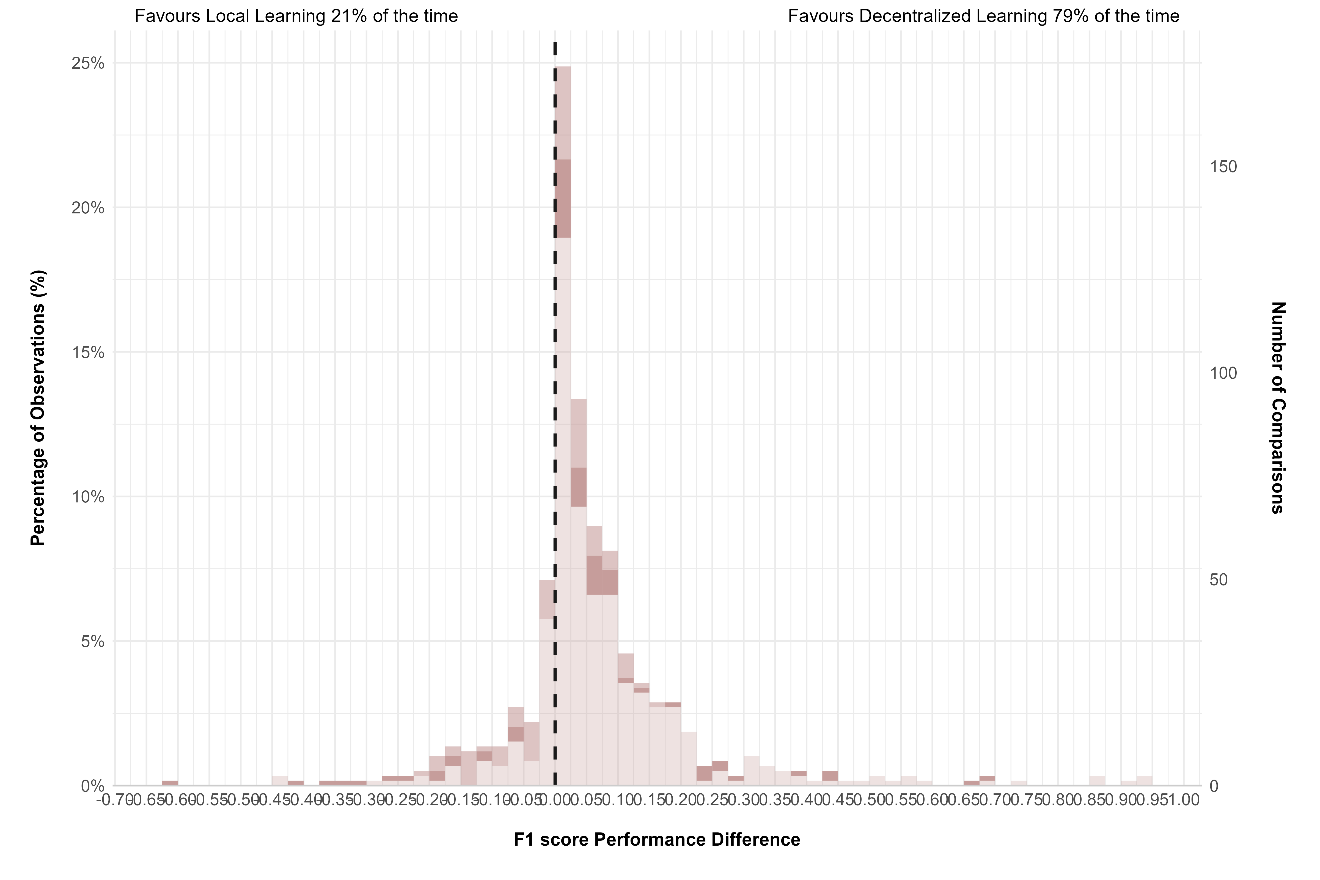


Supplementary Figure 33 - Distribution of Individual Model Performance Differences - Across F1 score (Comparing Decentralized Learning versus Local Learning), by nature of data collection. Dark green, very light green and light green represent models using Primary data collection, Secondary data collection or Both, respectively. Based on 593 observations extracted from 74 models of 27 studies. Summary Results: 67 comparisons based on Primary data collection | 25th Percentile = 0.0040 | 75th Percentile = 0.0955. Note: Dashed vertical line indicates no difference in performance between compared approaches.


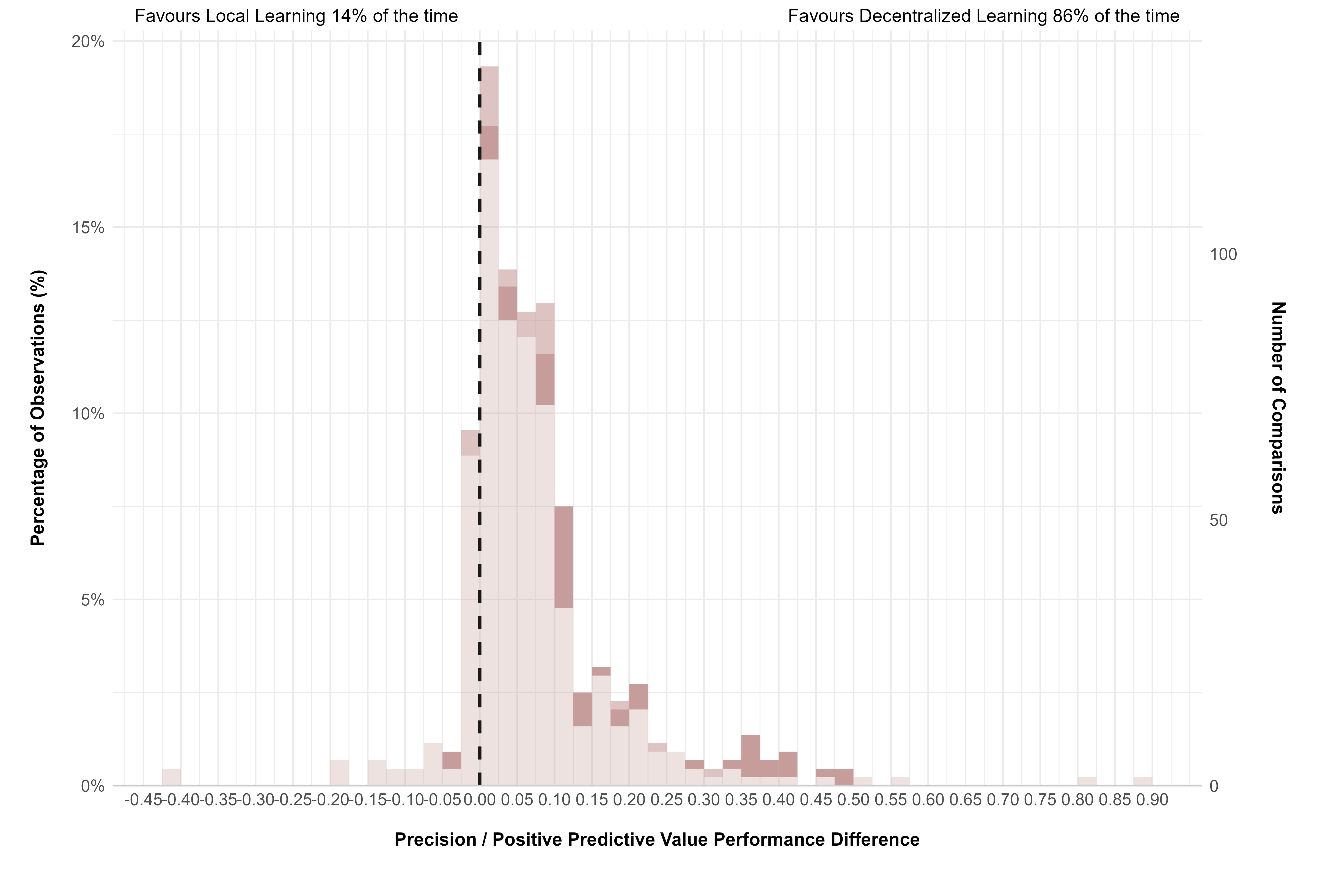


Supplementary Figure 34 - Distribution of Individual Model Performance Differences - Across Precision / Positive Predictive Value (Comparing Decentralized Learning versus Local Learning), by nature of data collection. Dark green, very light green and light green represent models using Primary data collection, Secondary data collection or Both, respectively. Based on 442 observations extracted from 60 models of 23 studies. Summary Results: 53 comparisons based on Primary data collection | 25th Percentile = 0.0120 | 75th Percentile = 0.1090. Note: Dashed vertical line indicates no difference in performance between compared approaches.


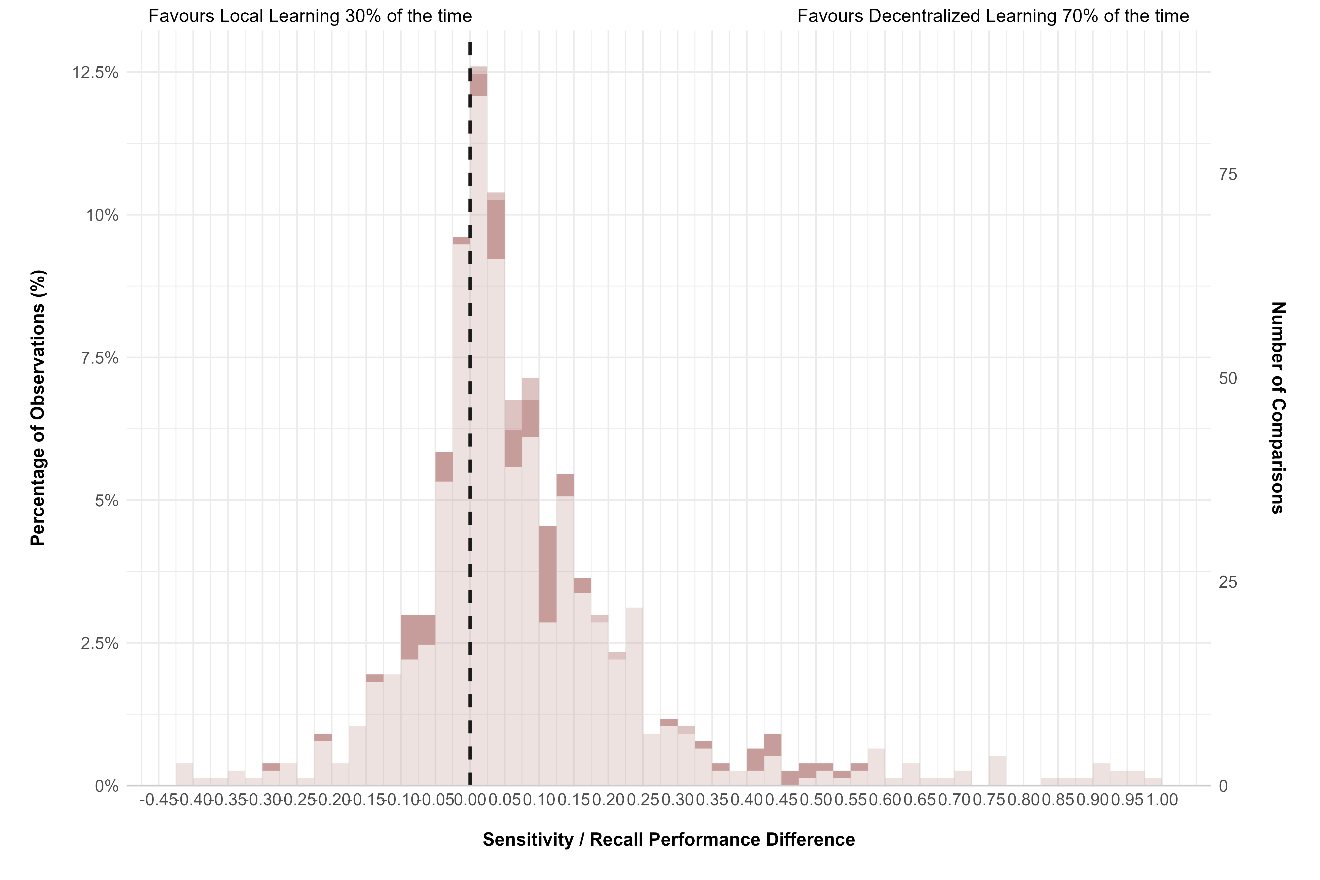


Supplementary Figure 35 - Distribution of Individual Model Performance Differences - Across Sensitivity / Recall (Comparing Decentralized Learning versus Local Learning), by nature of data collection. Dark green, very light green and light green represent models using Primary data collection, Secondary data collection or Both, respectively. Based on 772 observations extracted from 96 models of 35 studies. Summary Results: 73 comparisons based on Primary data collection | 25th Percentile = -0.0093 | 75th Percentile = 0.1427. Note: Dashed vertical line indicates no difference in performance between compared approaches.


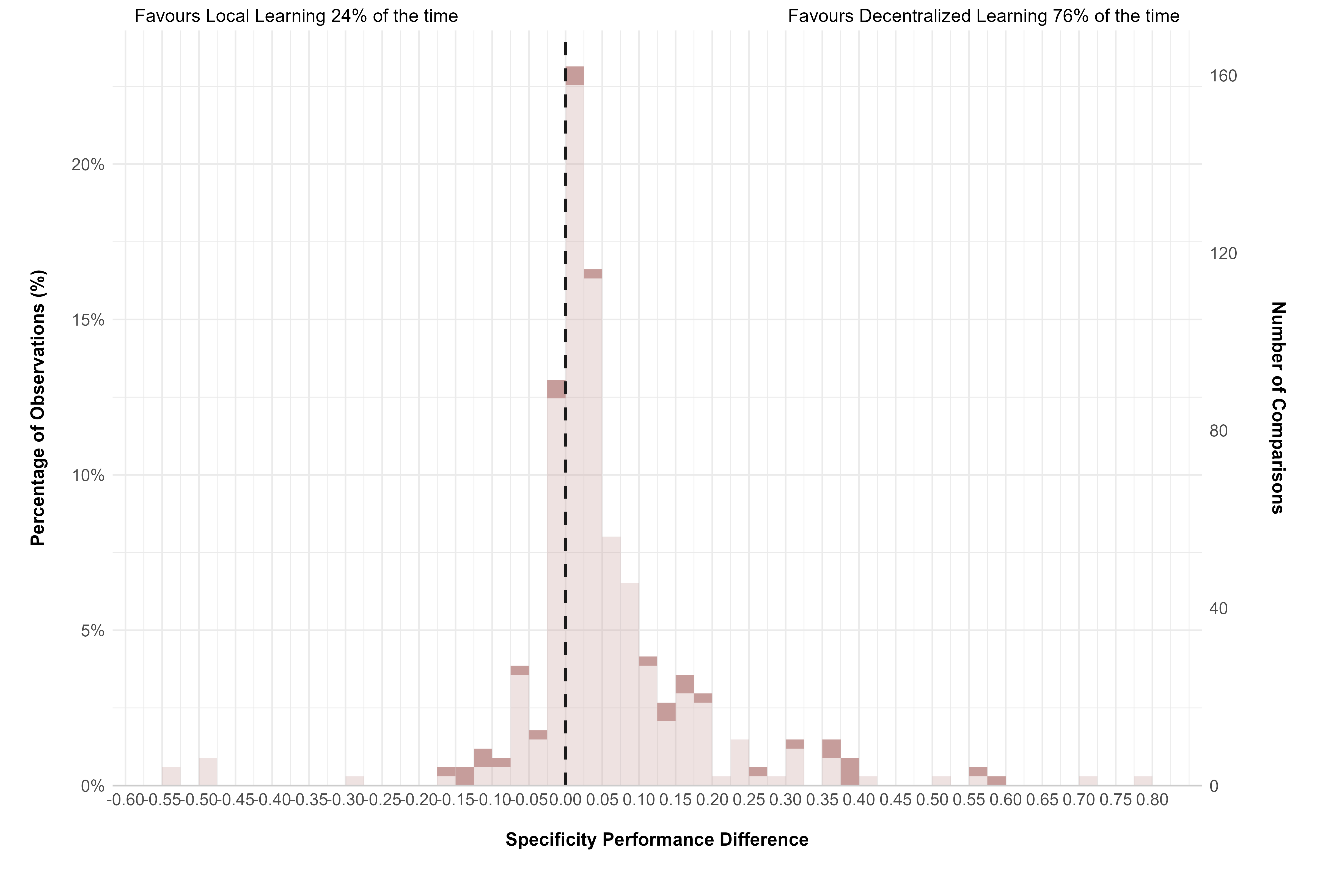


Supplementary Figure 36 - Distribution of Individual Model Performance Differences - Across Specificity (Comparing Decentralized Learning versus Local Learning), by nature of data collection. Dark green, very light green and light green represent models using Primary data collection, Secondary data collection or Both, respectively. Based on 339 observations extracted from 47 models of 16 studies. Summary Results: 28 comparisons based on Primary data collection | 25th Percentile = 0.0021 | 75th Percentile = 0.0898. Note: Dashed vertical line indicates no difference in performance between compared approaches.

## Detailed Absolute and Relative Differences Analysis

### Centralized Models Comparisons


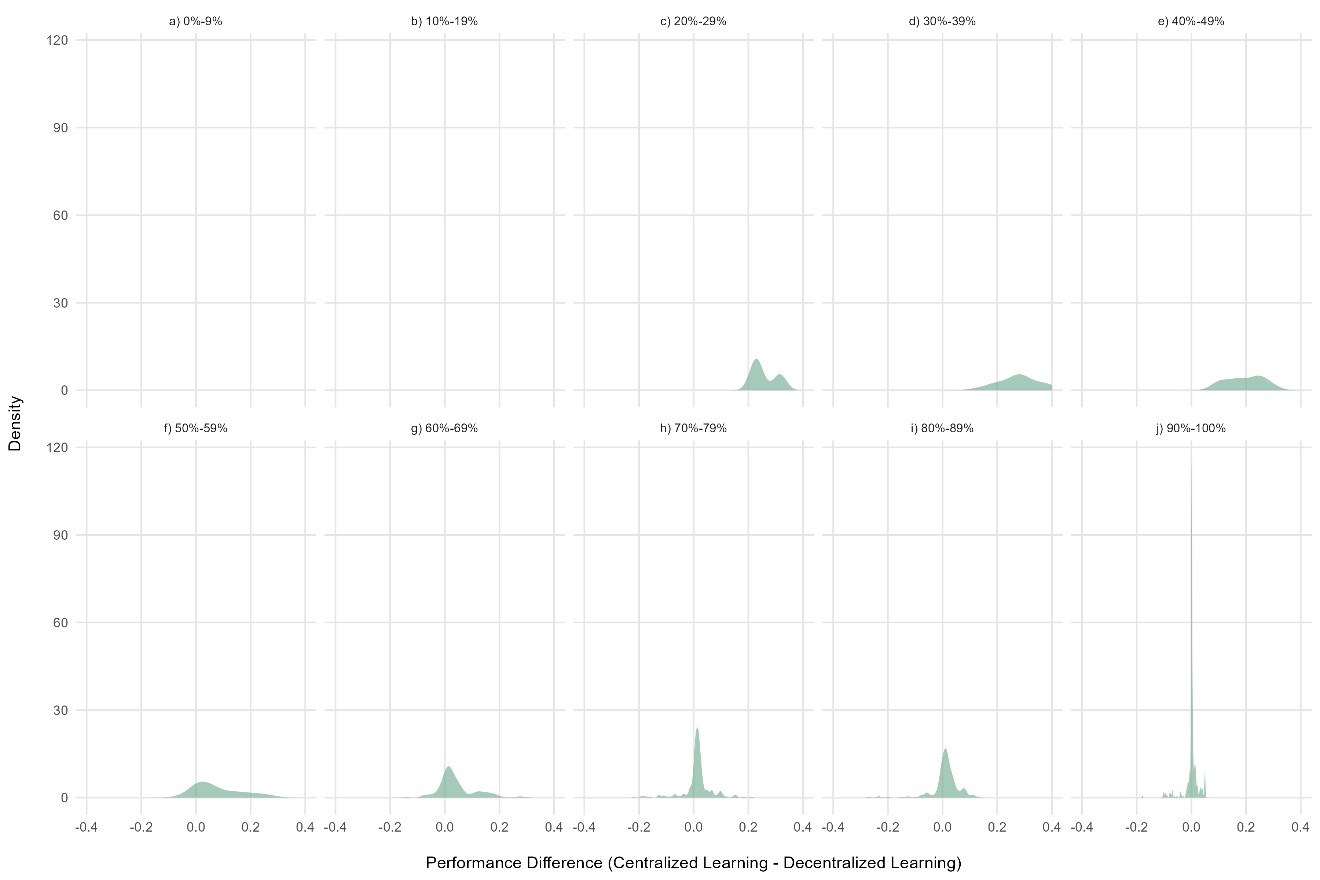


Supplementary Figure 37 - Distribution of Absolute Performance Differences by Decentralized Learning Value Percentiles in Accuracy, for percentiles a) [0%-10%[, b) [10%-20%[, c) [20%-30% [, d) [30%-40% [, e) [40%-50% [, f) [50%-60% [, g) [60%-70% [, h) [70%-80% [, i) [80%-90% [, j) [90%-100%].


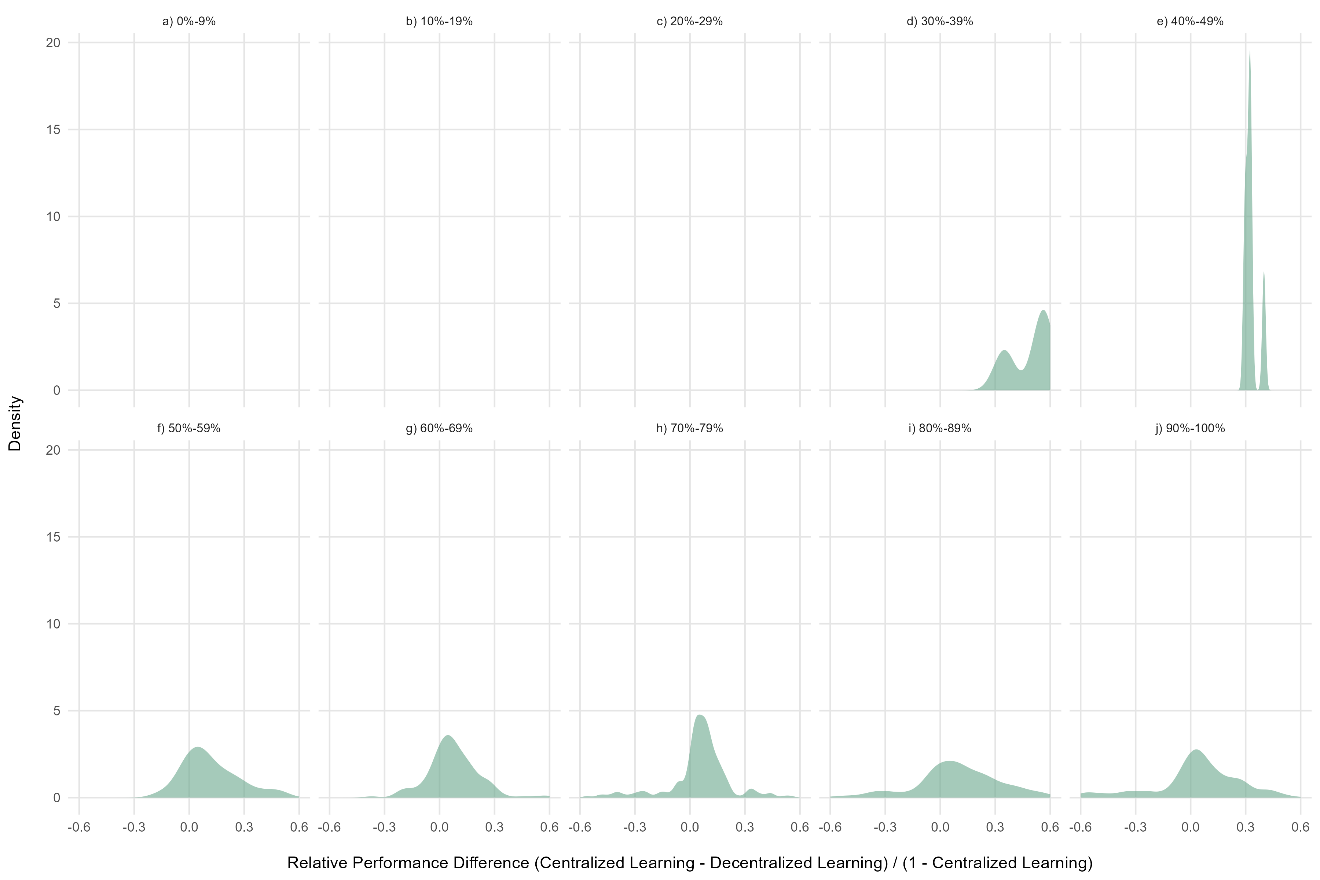


Supplementary Figure 38 - Distribution of Relative Performance Differences by Decentralized Learning Value Percentiles in Accuracy, for percentiles a) [0%-10%[, b) [10%-20%[, c) [20%-30% [, d) [30%-40% [, e) [40%-50% [, f) [50%-60% [, g) [60%-70% [, h) [70%-80% [, i) [80%-90% [, j) [90%-100%].


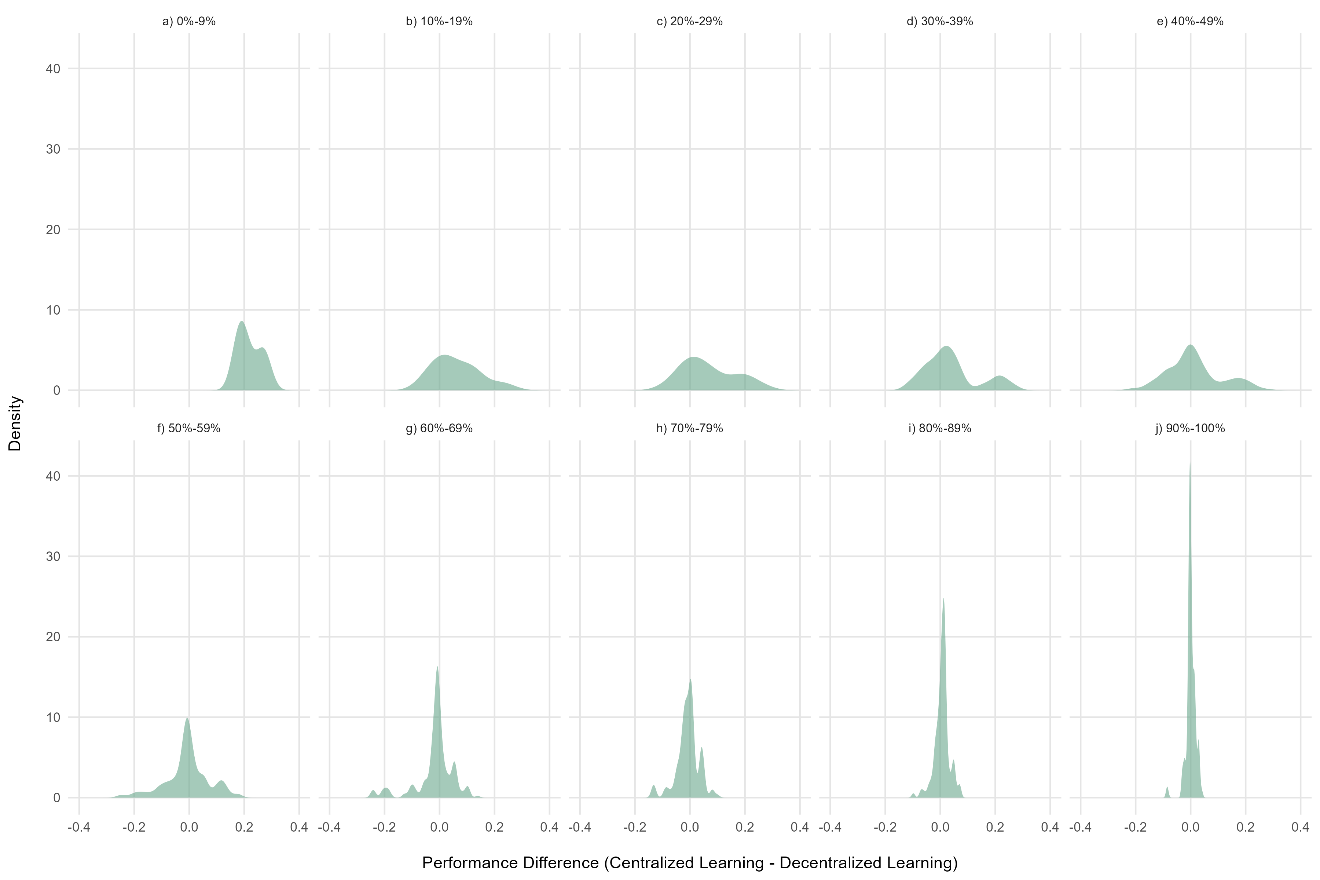


Supplementary Figure 39 - Distribution of Absolute Performance Differences by Decentralized Learning Value Percentiles in AUROC, for percentiles a) [0%-10%[, b) [10%-20%[, c) [20%-30% [, d) [30%-40% [, e) [40%-50% [, f) [50%-60% [, g) [60%-70% [, h) [70%-80% [, i) [80%-90% [, j) [90%-100%].


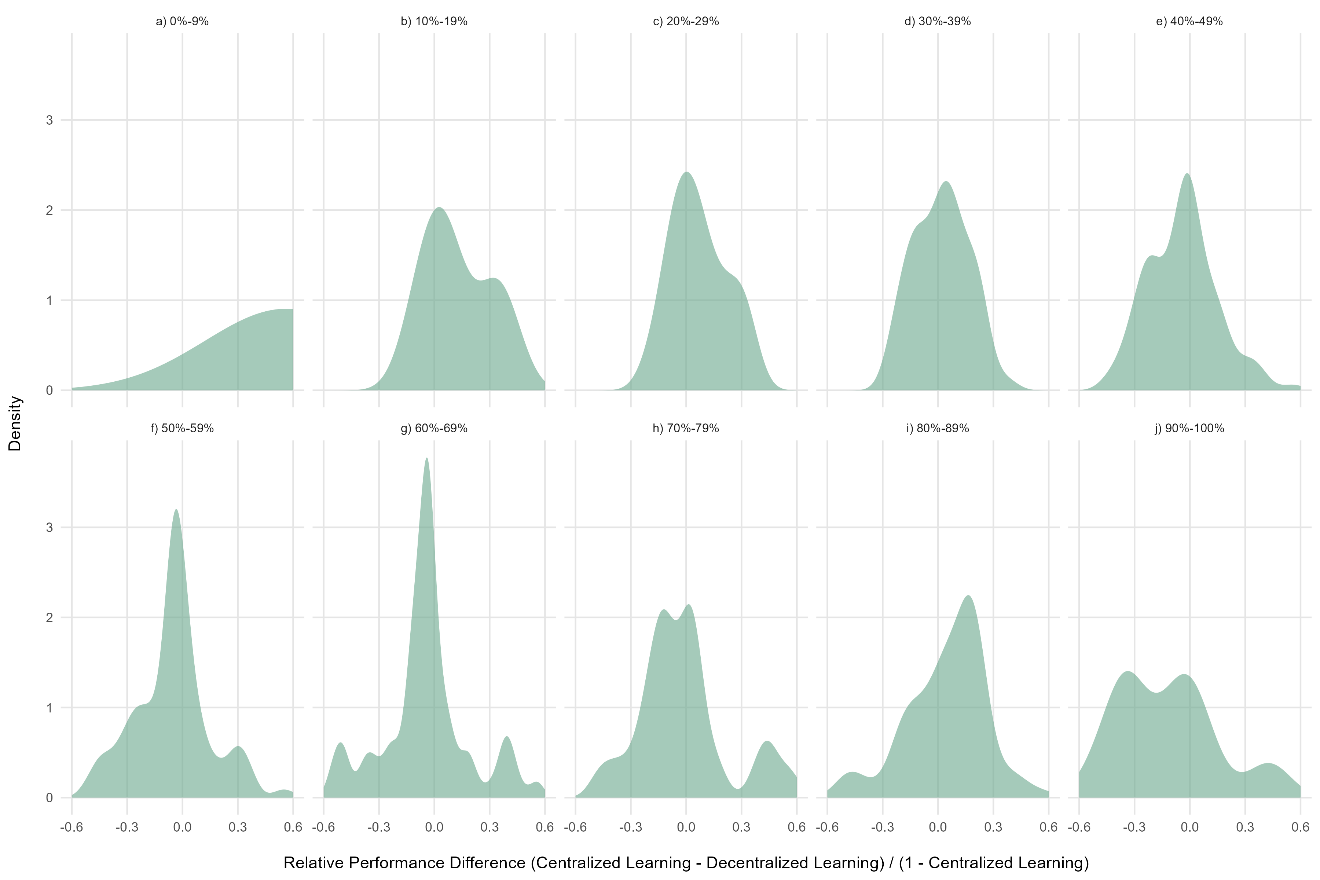


Supplementary Figure 40 - Distribution of Relative Performance Differences by Decentralized Learning Value Percentiles in AUROC, for percentiles a) [0%-10%[, b) [10%-20%[, c) [20%-30% [, d) [30%-40% [, e) [40%-50% [, f) [50%-60% [, g) [60%-70% [, h) [70%-80% [, i) [80%-90% [, j) [90%-100%].


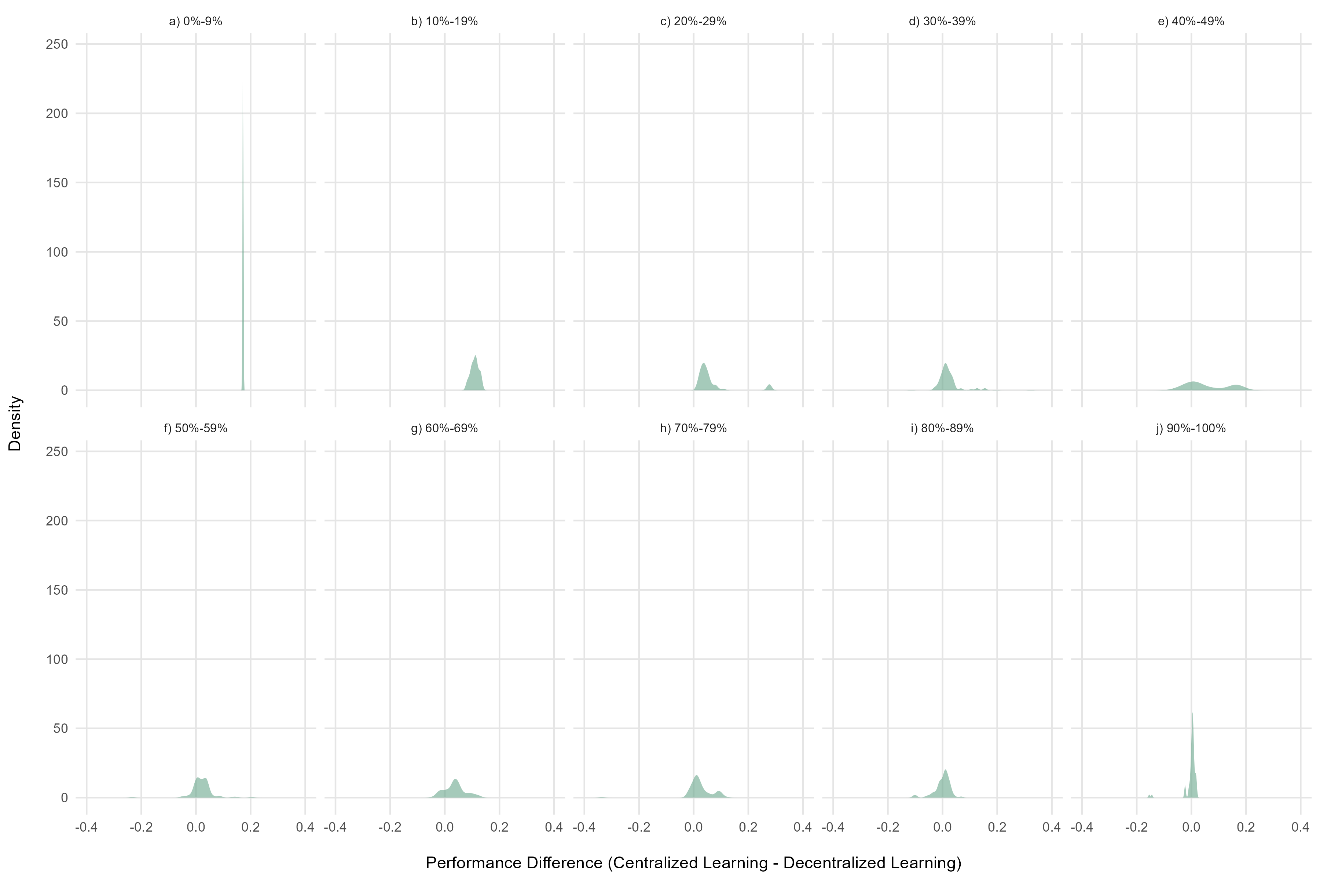


Supplementary Figure 41 - Distribution of Absolute Performance Differences by Decentralized Learning Value Percentiles in Dice score, for percentiles a) [0%-10%[, b) [10%-20%[, c) [20%-30% [, d) [30%-40% [, e) [40%-50% [, f) [50%-60% [, g) [60%-70% [, h) [70%-80% [, i) [80%-90% [, j) [90%-100%].


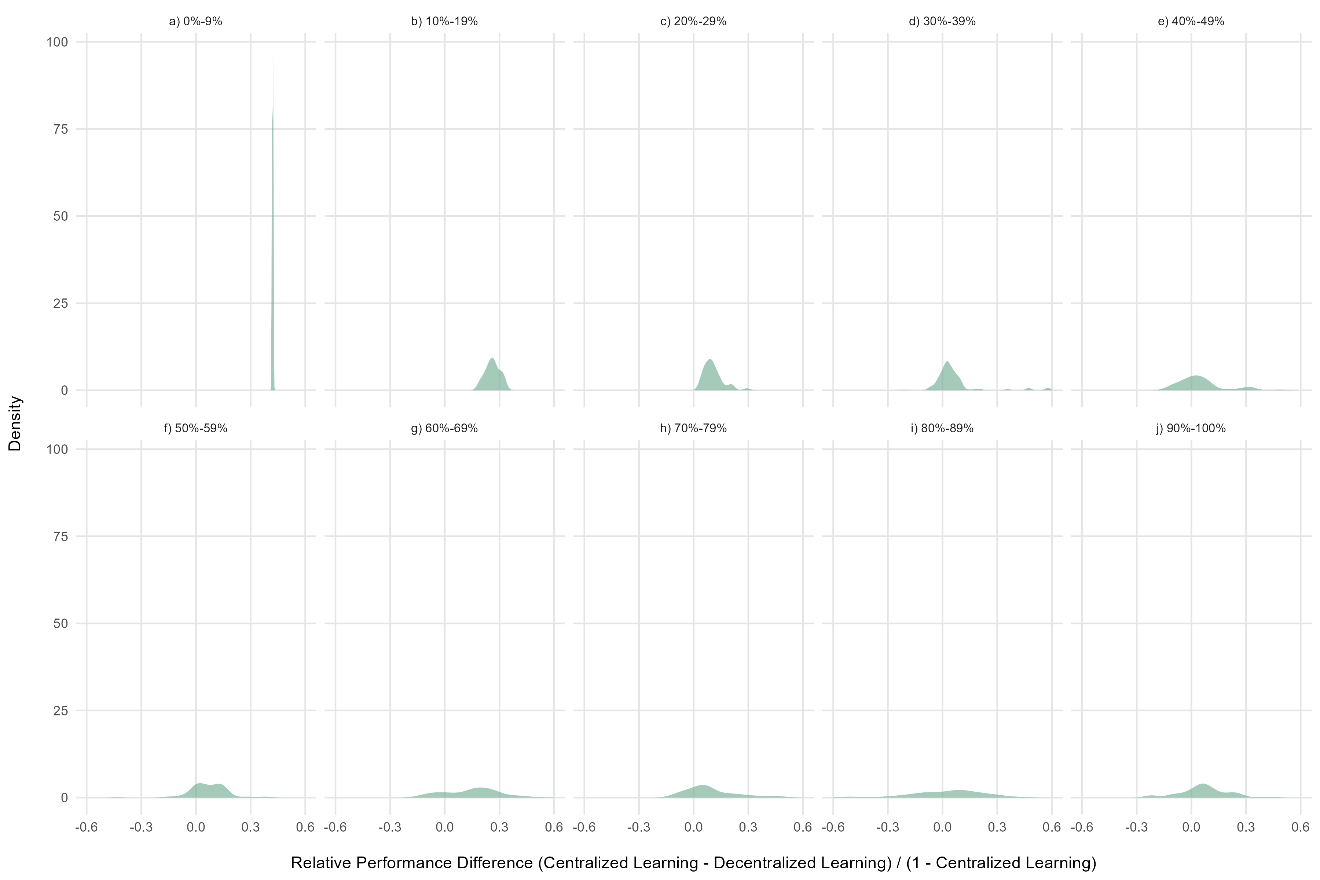


Supplementary Figure 42 - Distribution of Relative Performance Differences by Decentralized Learning Value Percentiles in Dice score, for percentiles a) [0%-10%[, b) [10%-20%[, c) [20%-30% [, d) [30%-40% [, e) [40%-50% [, f) [50%-60% [, g) [60%-70% [, h) [70%-80% [, i) [80%-90% [, j) [90%-100%].


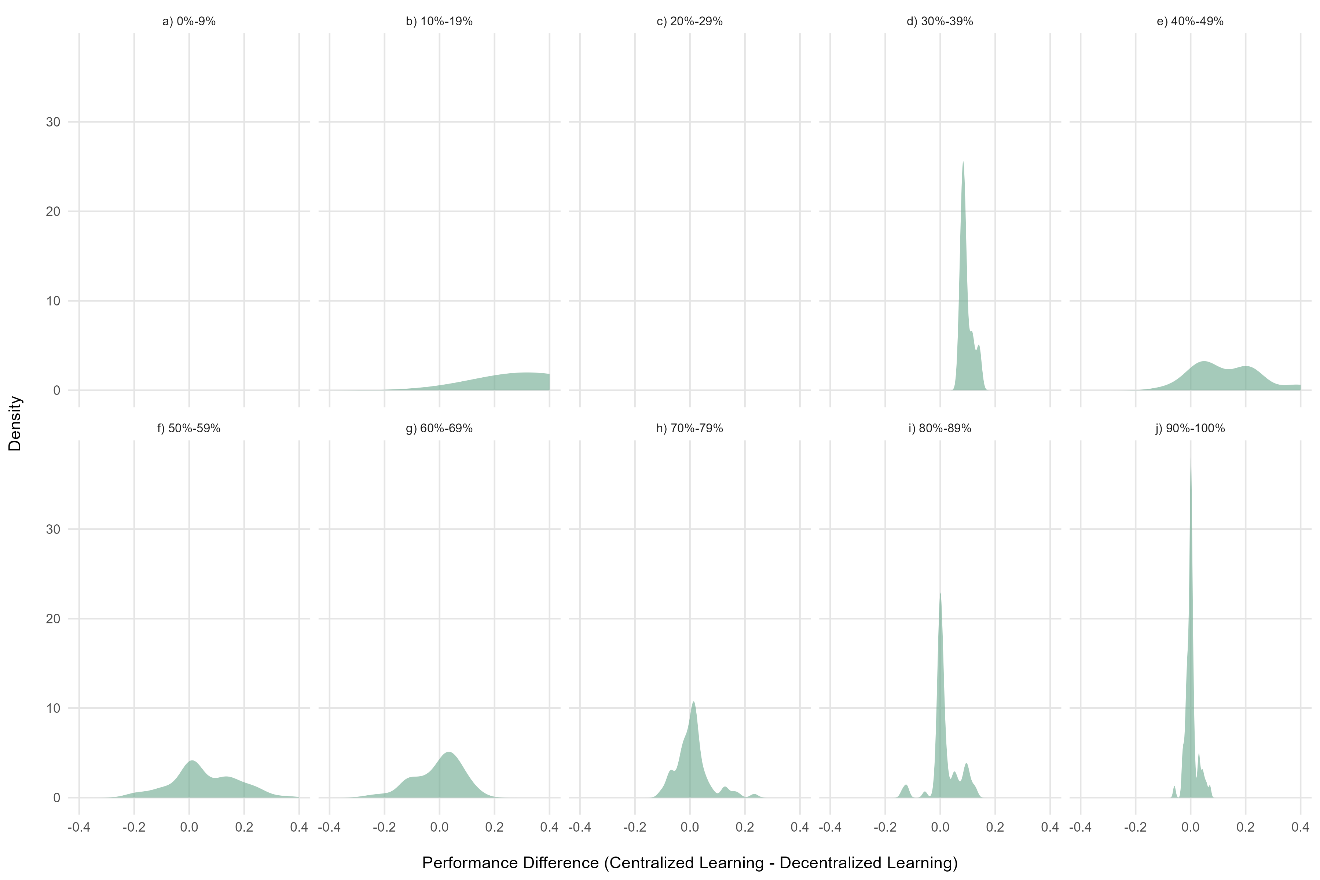


Supplementary Figure 43 - Distribution of Absolute Performance Differences by Decentralized Learning Value Percentiles in F1 score, for percentiles a) [0%-10%[, b) [10%-20%[, c) [20%-30% [, d) [30%-40% [, e) [40%-50% [, f) [50%-60% [, g) [60%-70% [, h) [70%-80% [, i) [80%-90% [, j) [90%-100%].


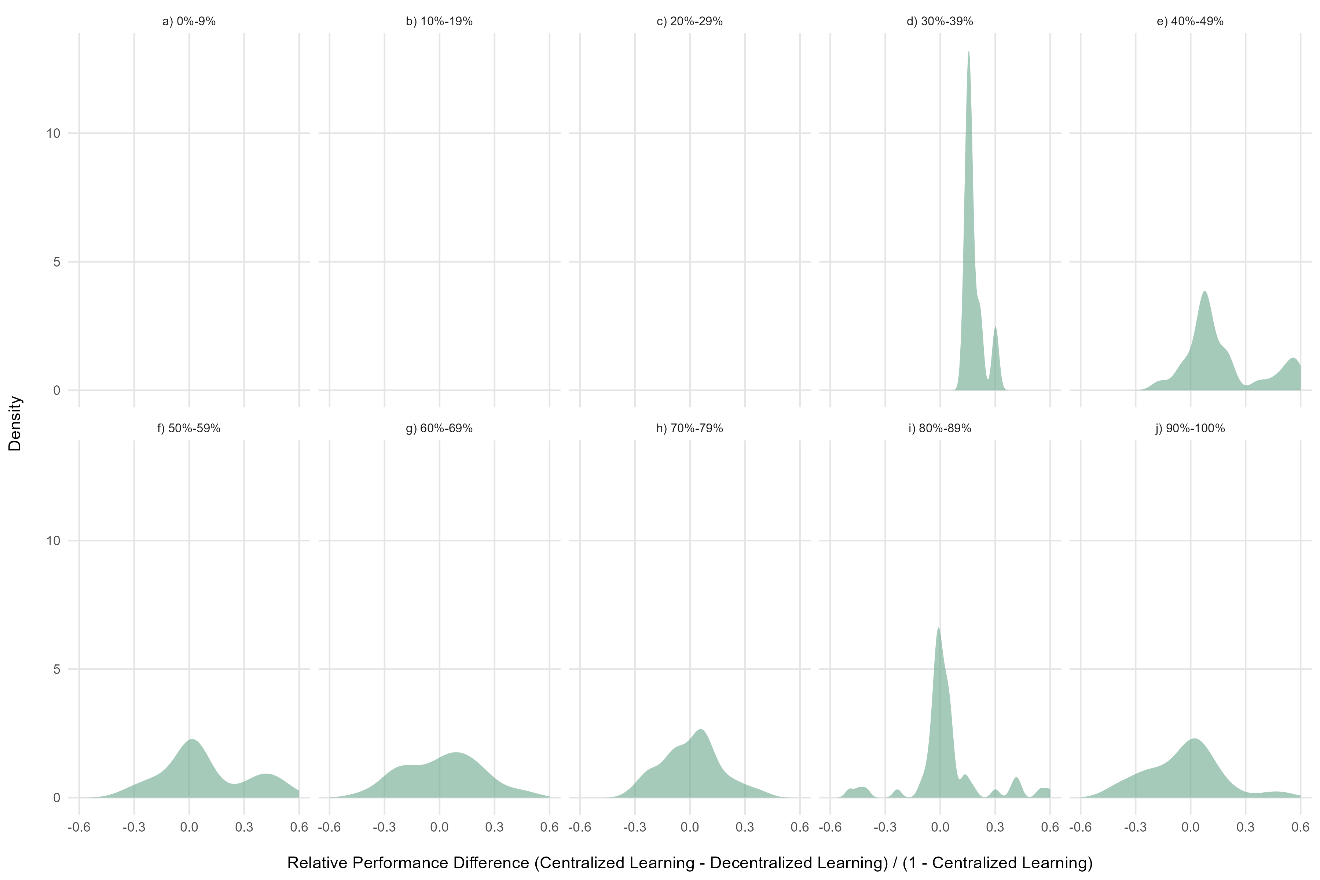


Supplementary Figure 44 - Distribution of Relative Performance Differences by Decentralized Learning Value Percentiles in F1 score, for percentiles a) [0%-10%[, b) [10%-20%[, c) [20%-30% [, d) [30%-40% [, e) [40%-50% [, f) [50%-60% [, g) [60%-70% [, h) [70%-80% [, i) [80%-90% [, j) [90%-100%].


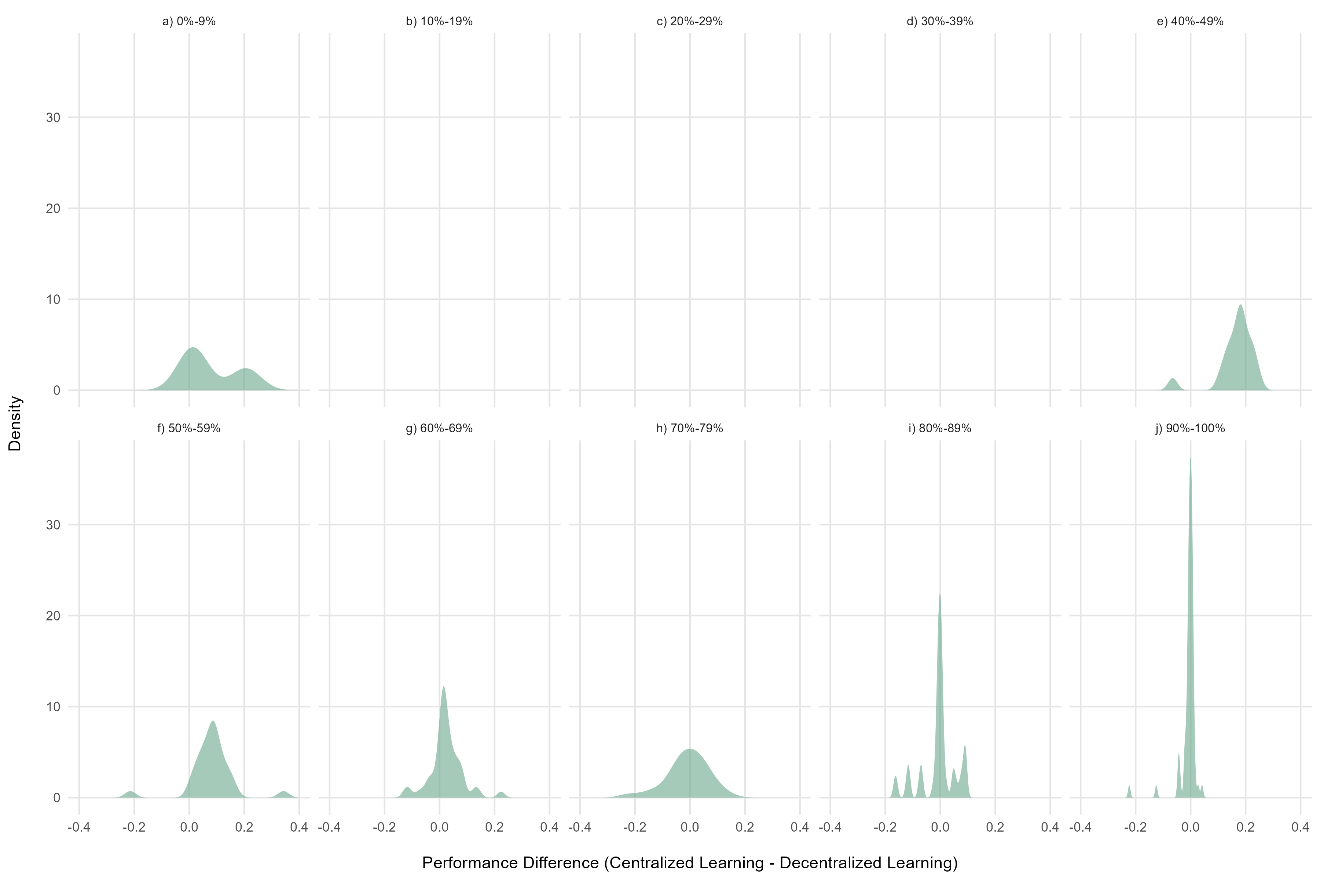


Supplementary Figure 45 - Distribution of Absolute Performance Differences by Decentralized Learning Value Percentiles in Precision / Positive Predictive Value, for percentiles a) [0%-10%[, b) [10%-20%[, c) [20%-30% [, d) [30%-40% [, e) [40%-50% [, f) [50%-60% [, g) [60%-70% [, h) [70%-80% [, i) [80%-90% [, j) [90%-100%].


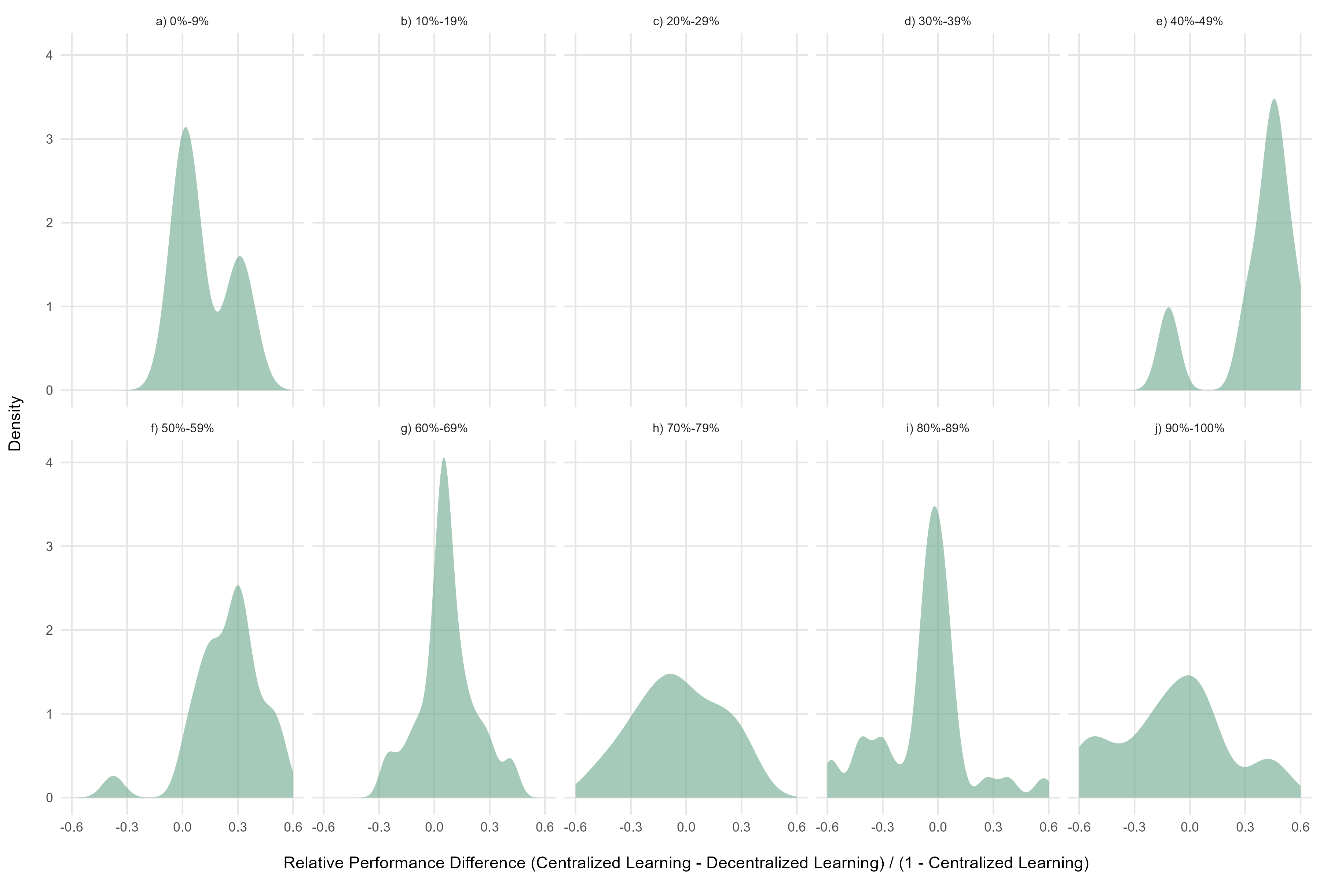


Supplementary Figure 46 - Distribution of Relative Performance Differences by Decentralized Learning Value Percentiles in Precision / Positive Predictive Value, for percentiles a) [0%-10%[, b) [10%-20%[, c) [20%-30% [, d) [30%-40% [, e) [40%-50% [, f) [50%-60% [, g) [60%-70% [, h) [70%-80% [, i) [80%-90% [, j) [90%-100%].


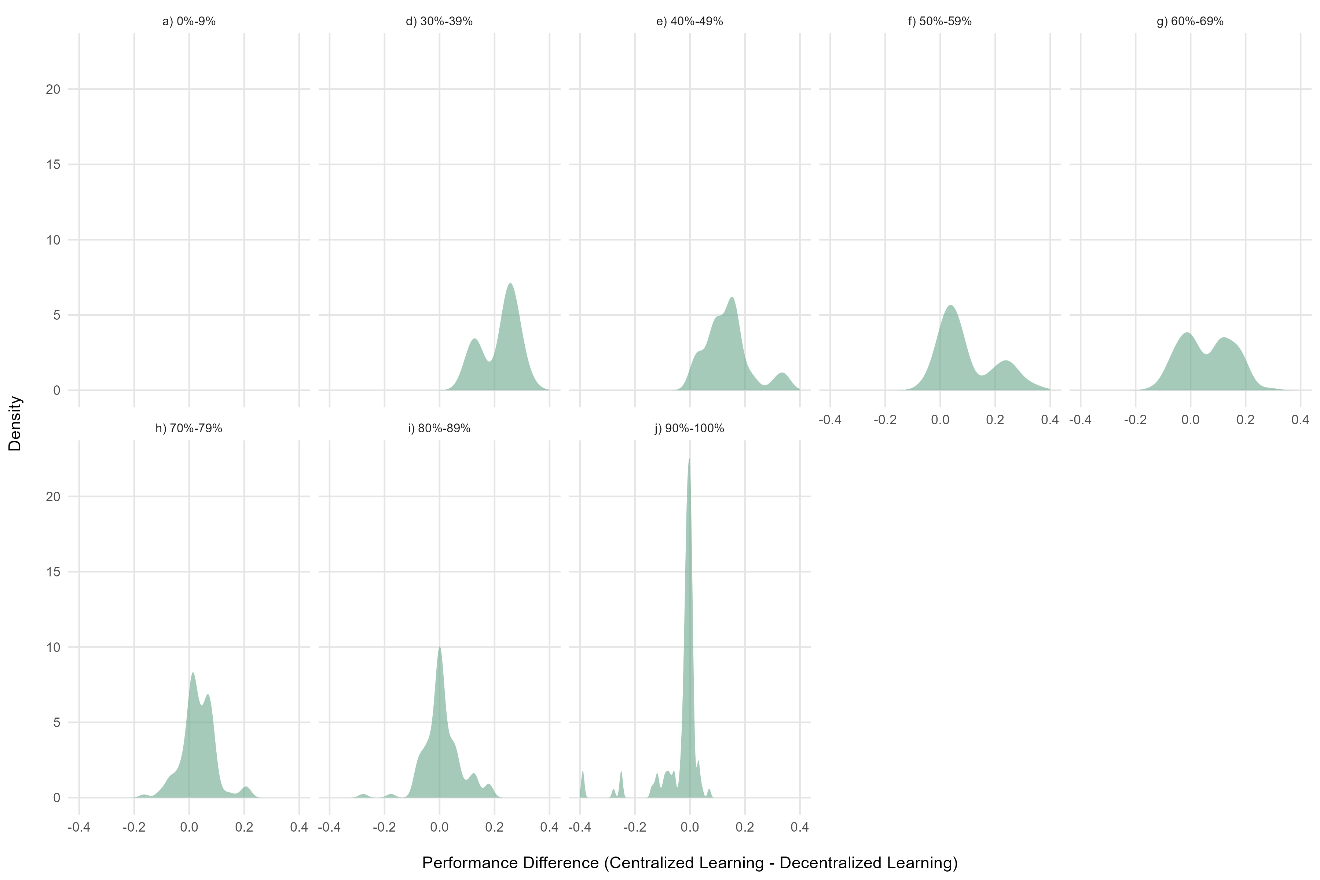


Supplementary Figure 47 - Distribution of Absolute Performance Differences by Decentralized Learning Value Percentiles in Sensitivity / Recall, for percentiles a) [0%-10%[, b) [10%-20%[, c) [20%-30% [, d) [30%-40% [, e) [40%-50% [, f) [50%-60% [, g) [60%-70% [, h) [70%-80% [, i) [80%-90% [, j) [90%-100%].


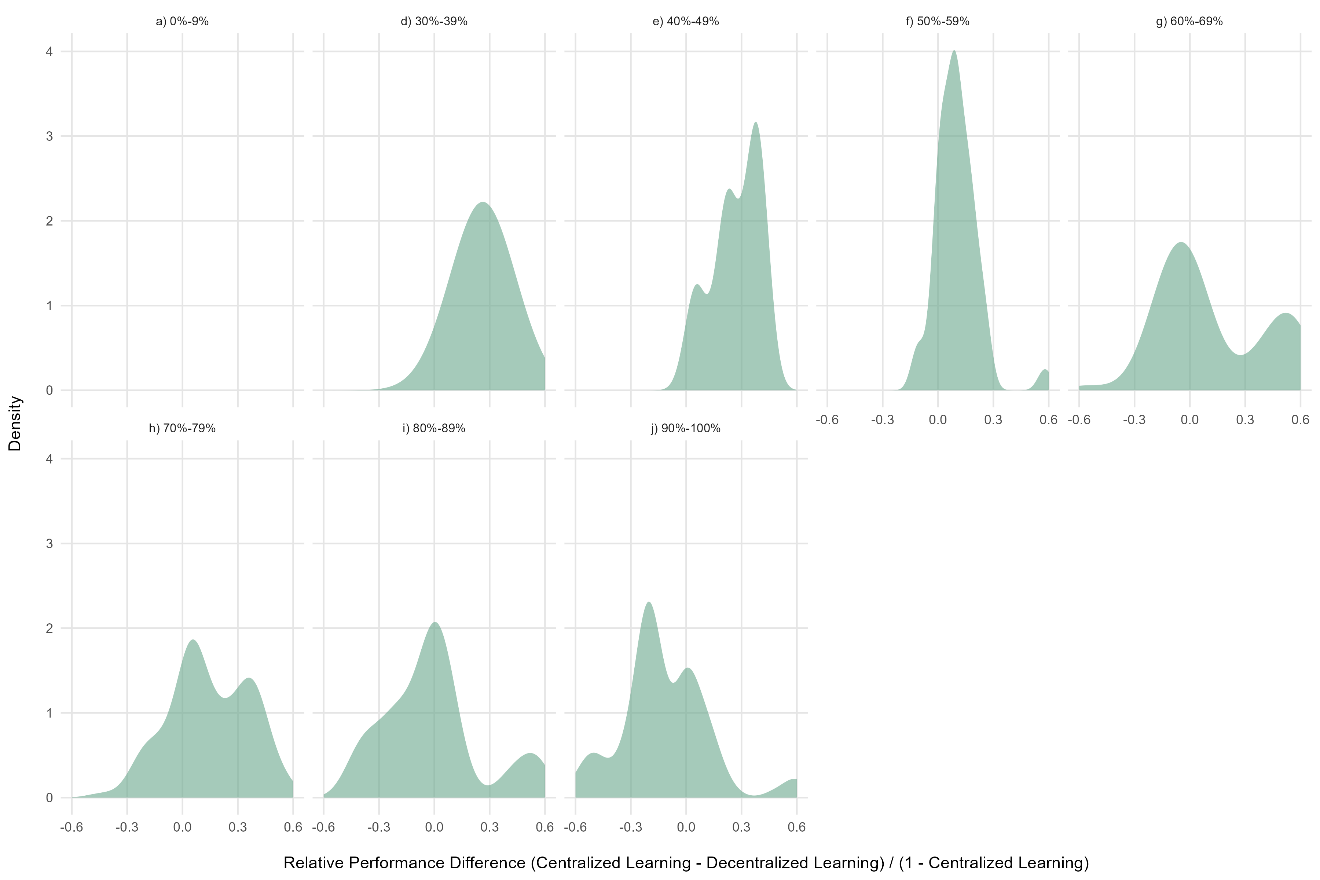


Supplementary Figure 48 - Distribution of Relative Performance Differences by Decentralized Learning Value Percentiles in Sensitivity / Recall, for percentiles a) [0%-10%[, b) [10%-20%[, c) [20%-30% [, d) [30%-40% [, e) [40%-50% [, f) [50%-60% [, g) [60%-70% [, h) [70%-80% [, i) [80%-90% [, j) [90%-100%].


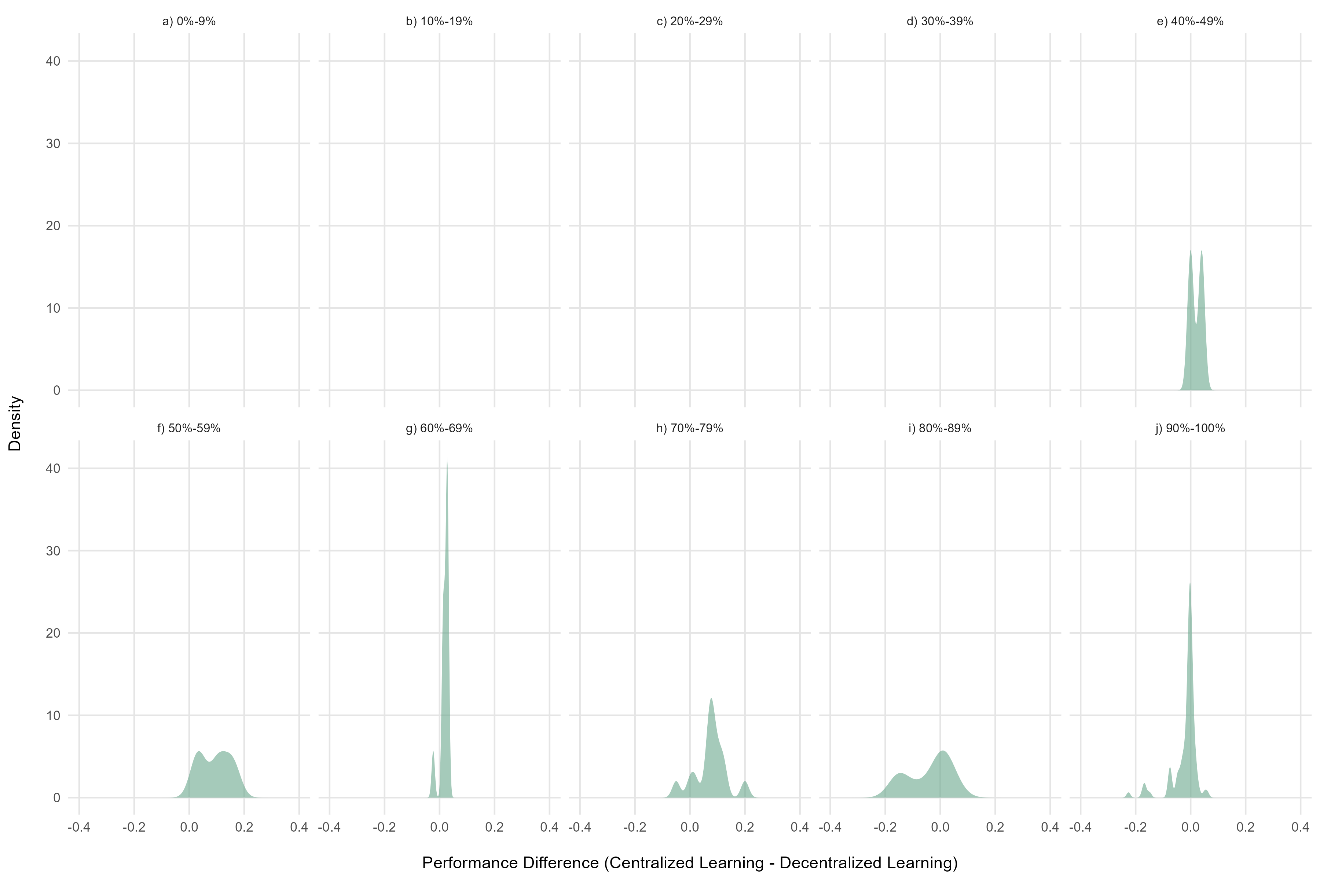


Supplementary Figure 49 - Distribution of Absolute Performance Differences by Decentralized Learning Value Percentiles in Specificity, for percentiles a) [0%-10%[, b) [10%-20%[, c) [20%-30% [, d) [30%-40% [, e) [40%-50% [, f) [50%-60% [, g) [60%-70% [, h) [70%-80% [, i) [80%-90% [, j) [90%-100%].


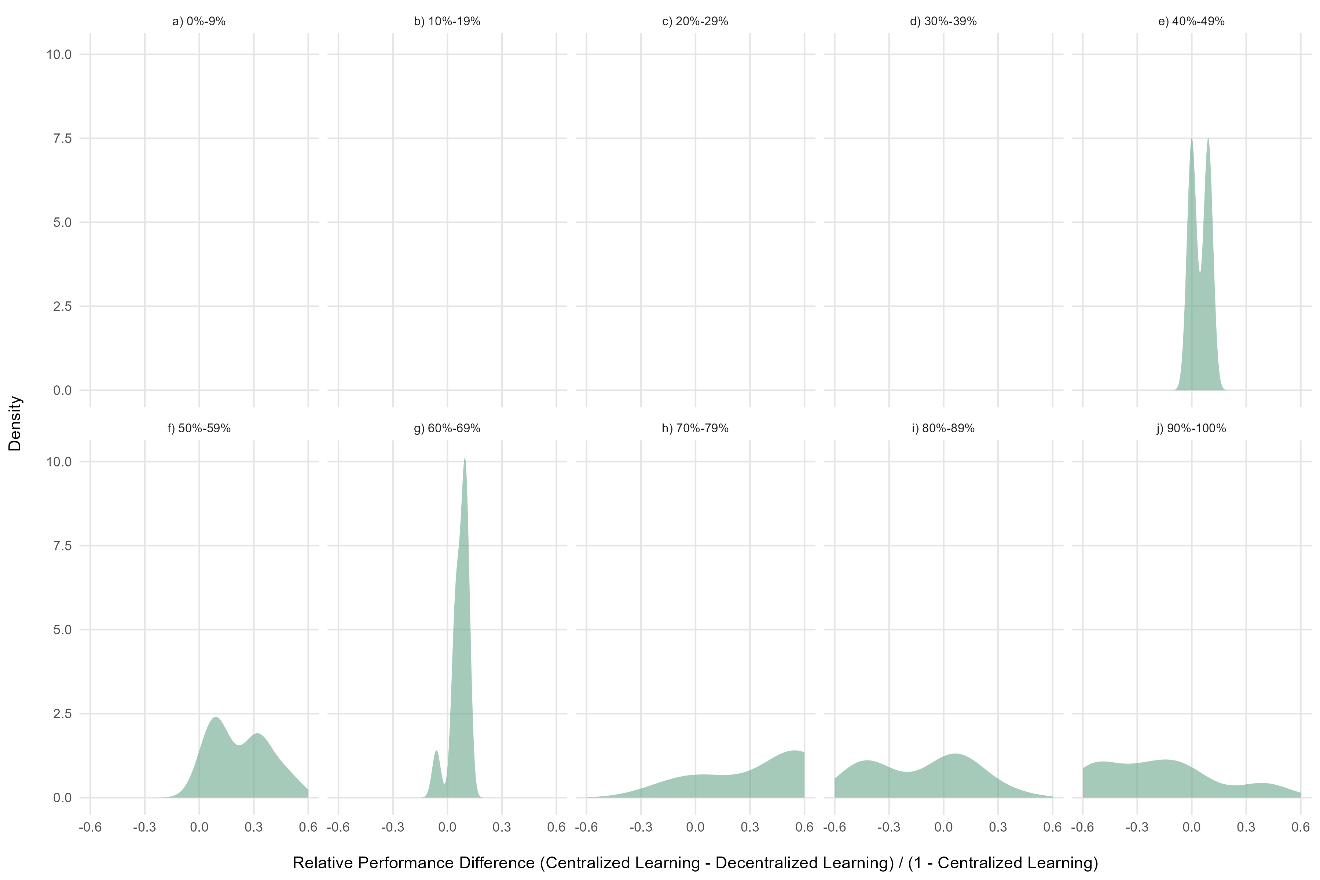


Supplementary Figure 50 - Distribution of Relative Performance Differences by Decentralized Learning Value Percentiles in Specificity, for percentiles a) [0%-10%[, b) [10%-20%[, c) [20%-30% [, d) [30%-40% [, e) [40%-50% [, f) [50%-60% [, g) [60%-70% [, h) [70%-80% [, i) [80%-90% [, j) [90%-100%].

### Local Models Comparisons


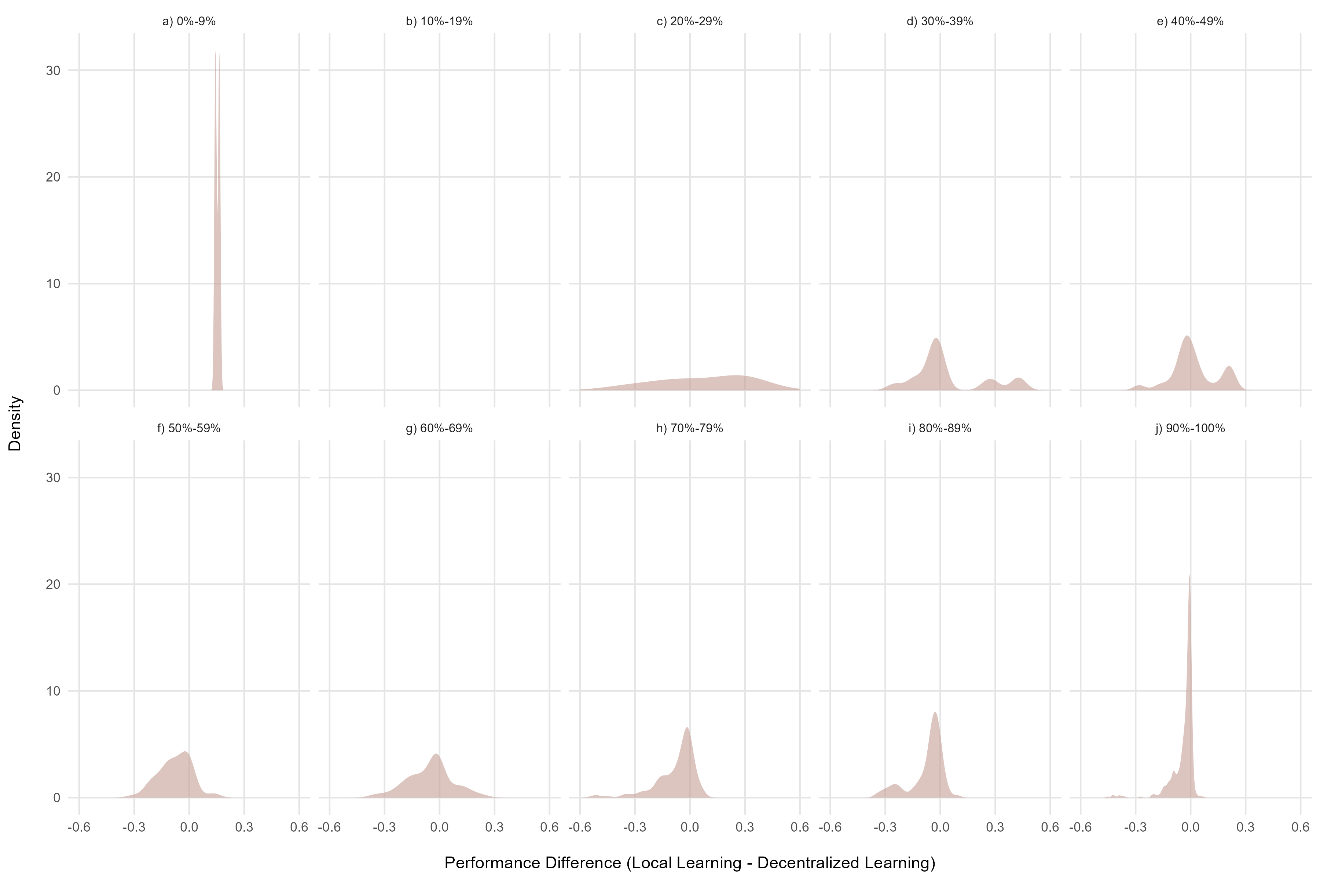


Supplementary Figure 51 - Distribution of Absolute Performance Differences by Decentralized Learning Value Percentiles in Accuracy, for percentiles a) [0%-10%[, b) [10%-20%[, c) [20%-30% [, d) [30%-40% [, e) [40%-50% [, f) [50%-60% [, g) [60%-70% [, h) [70%-80% [, i) [80%-90% [, j) [90%-100%].


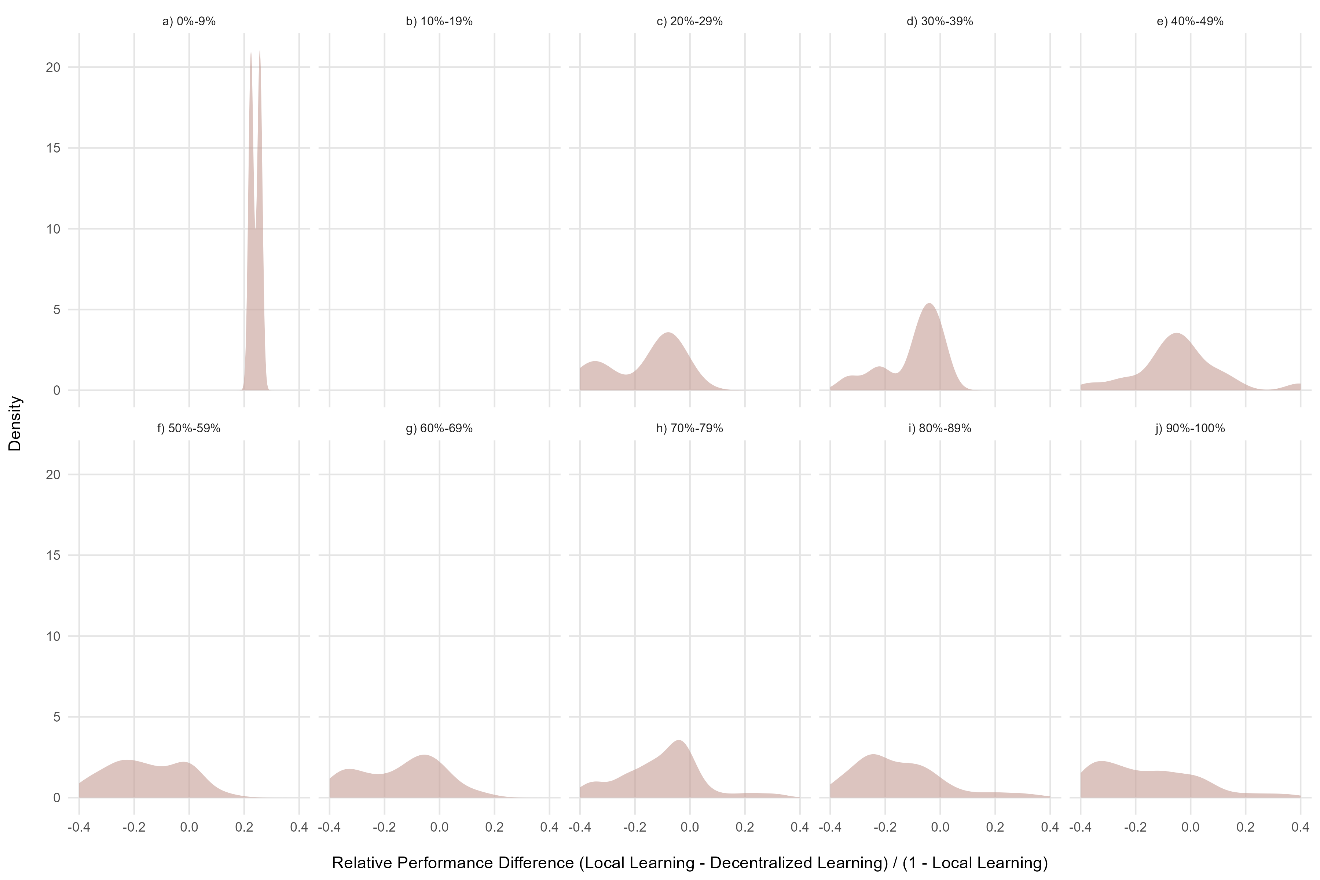


Supplementary Figure 52 - Distribution of Relative Performance Differences by Decentralized Learning Value Percentiles in Accuracy, for percentiles a) [0%-10%[, b) [10%-20%[, c) [20%-30% [, d) [30%-40% [, e) [40%-50% [, f) [50%-60% [, g) [60%-70% [, h) [70%-80% [, i) [80%-90% [, j) [90%-100%].


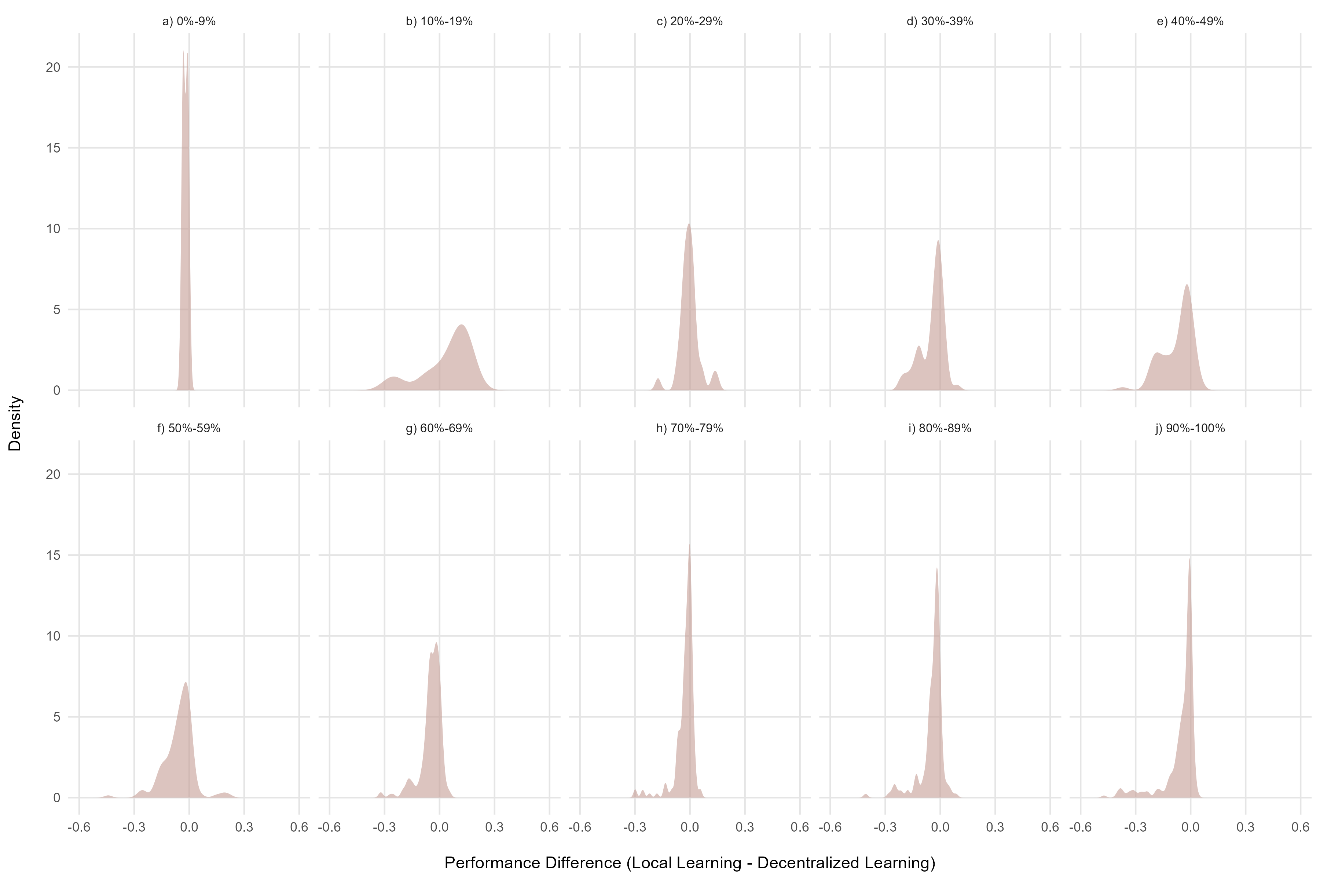


Supplementary Figure 53 - Distribution of Absolute Performance Differences by Decentralized Learning Value Percentiles in AUROC, for percentiles a) [0%-10%[, b) [10%-20%[, c) [20%-30% [, d) [30%-40% [, e) [40%-50% [, f) [50%-60% [, g) [60%-70% [, h) [70%-80% [, i) [80%-90% [, j) [90%-100%].


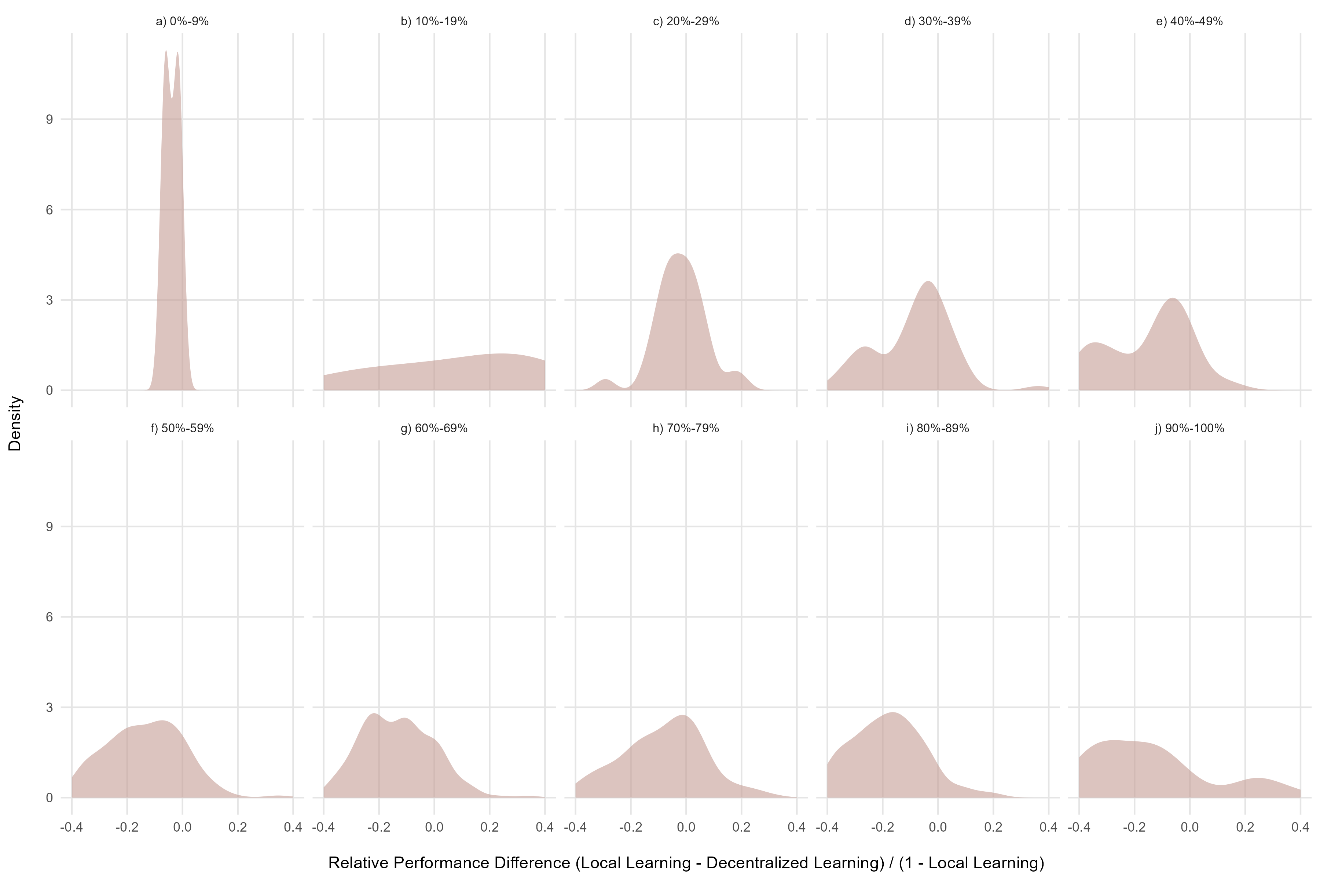


Supplementary Figure 54 - Distribution of Relative Performance Differences by Decentralized Learning Value Percentiles in AUROC, for percentiles a) [0%-10%[, b) [10%-20%[, c) [20%-30% [, d) [30%-40% [, e) [40%-50% [, f) [50%-60% [, g) [60%-70% [, h) [70%-80% [, i) [80%-90% [, j) [90%-100%].


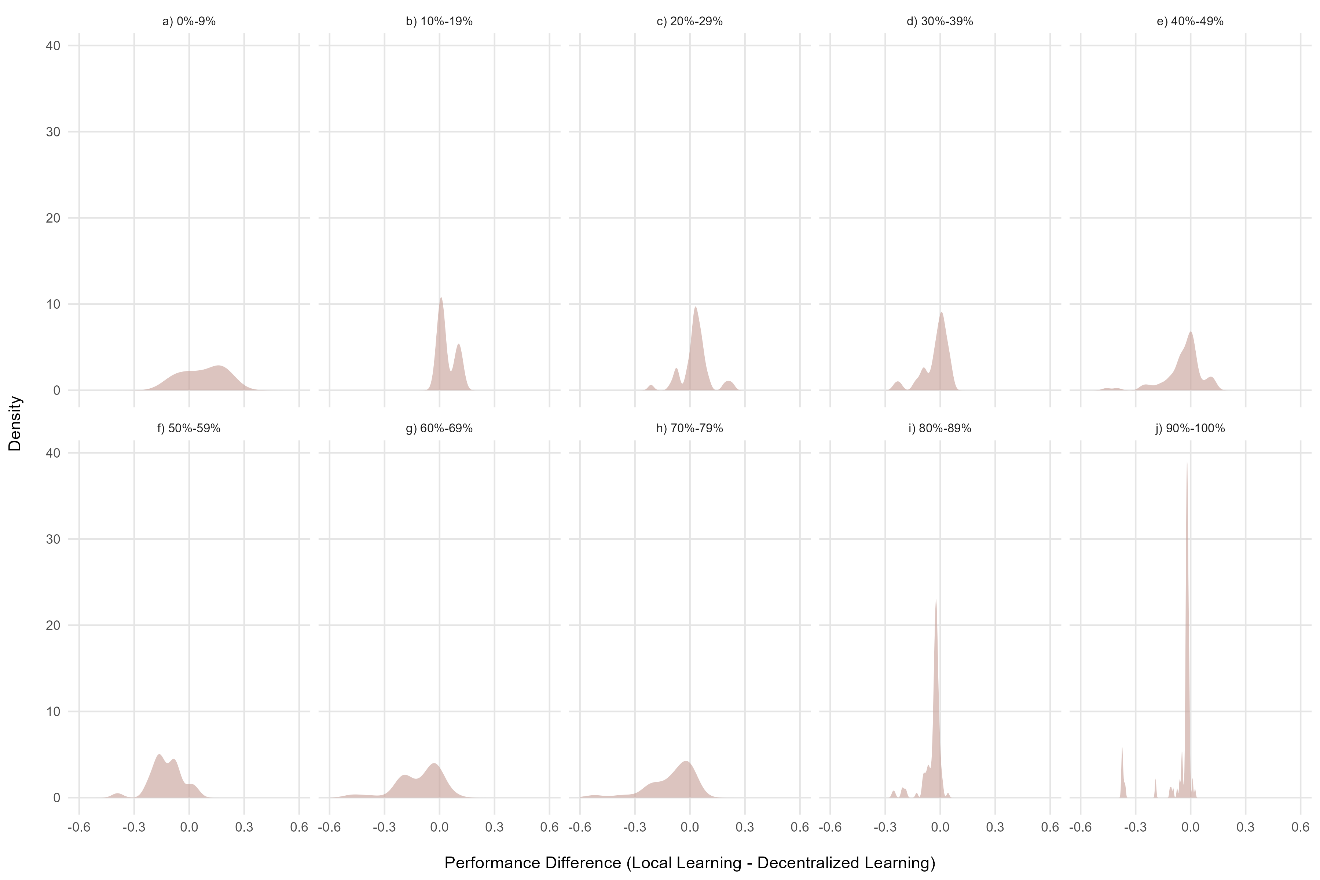


Supplementary Figure 55 - Distribution of Absolute Performance Differences by Decentralized Learning Value Percentiles in Dice score, for percentiles a) [0%-10%[, b) [10%-20%[, c) [20%-30% [, d) [30%-40% [, e) [40%-50% [, f) [50%-60% [, g) [60%-70% [, h) [70%-80% [, i) [80%-90% [, j) [90%-100%].


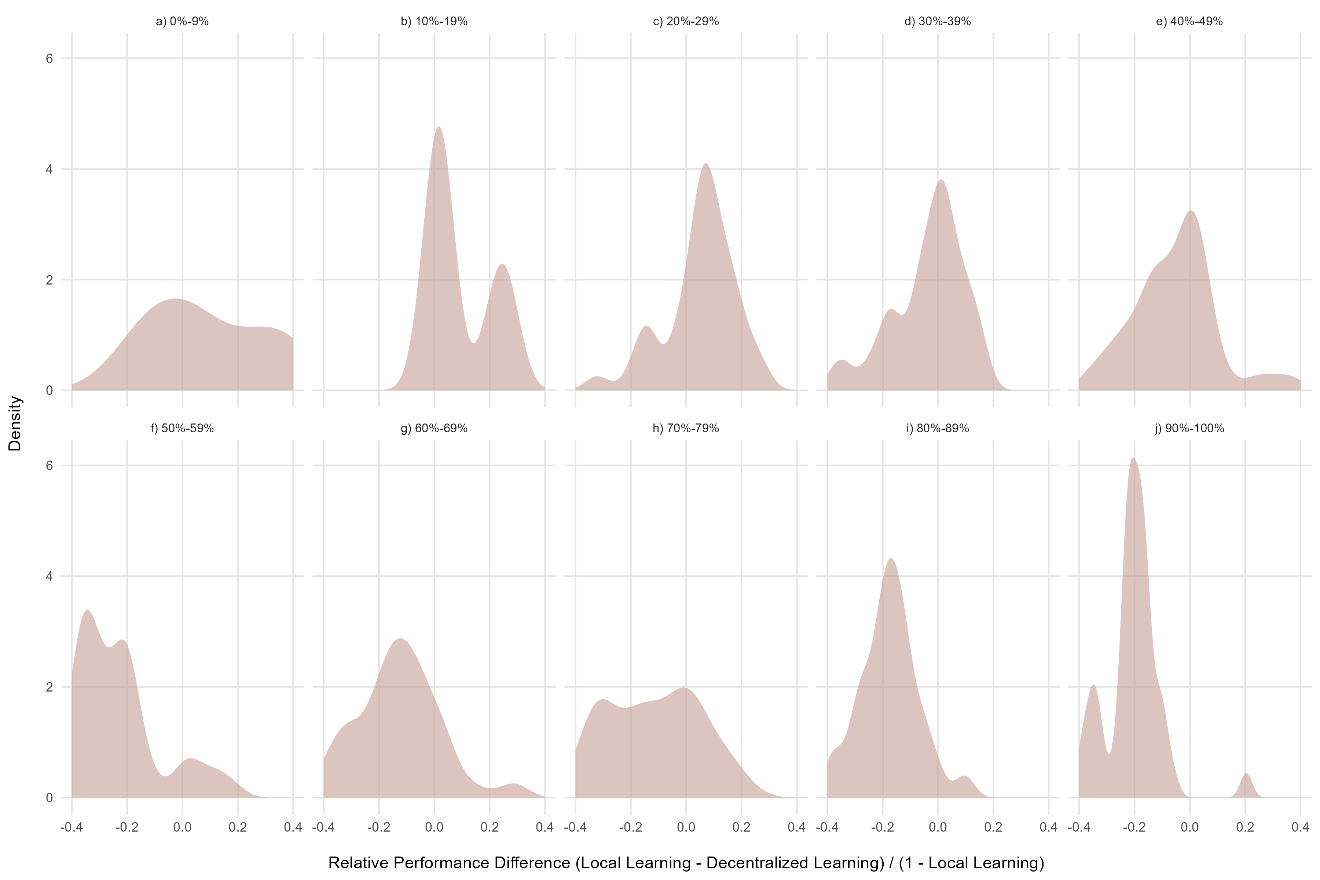


Supplementary Figure 56 - Distribution of Relative Performance Differences by Decentralized Learning Value Percentiles in Dice score, for percentiles a) [0%-10%[, b) [10%-20%[, c) [20%-30% [, d) [30%-40% [, e) [40%-50% [, f) [50%-60% [, g) [60%-70% [, h) [70%-80% [, i) [80%-90% [, j) [90%-100%].


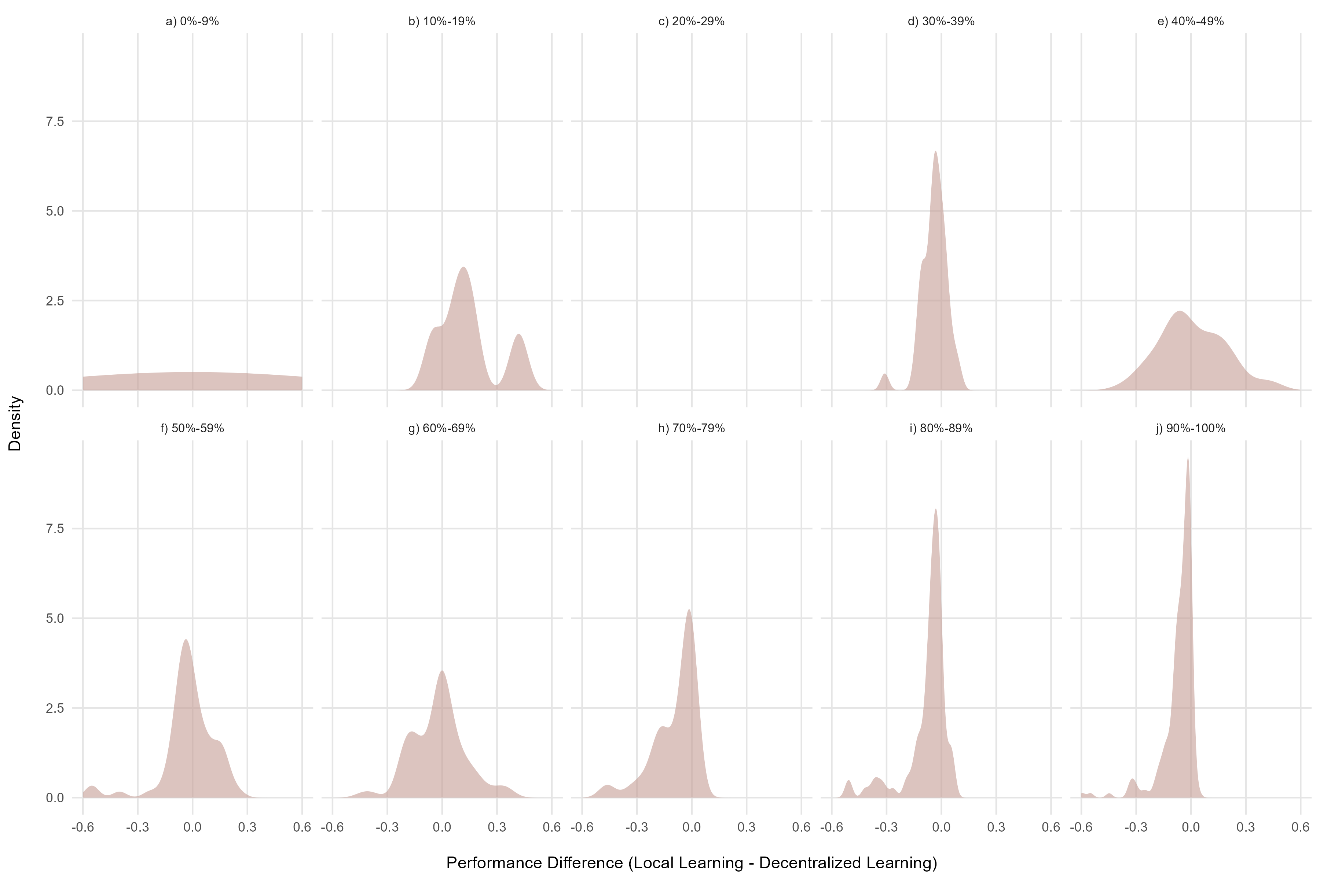


Supplementary Figure 57 - Distribution of Absolute Performance Differences by Decentralized Learning Value Percentiles in F1 score, for percentiles a) [0%-10%[, b) [10%-20%[, c) [20%-30% [, d) [30%-40% [, e) [40%-50% [, f) [50%-60% [, g) [60%-70% [, h) [70%-80% [, i) [80%-90% [, j) [90%-100%].


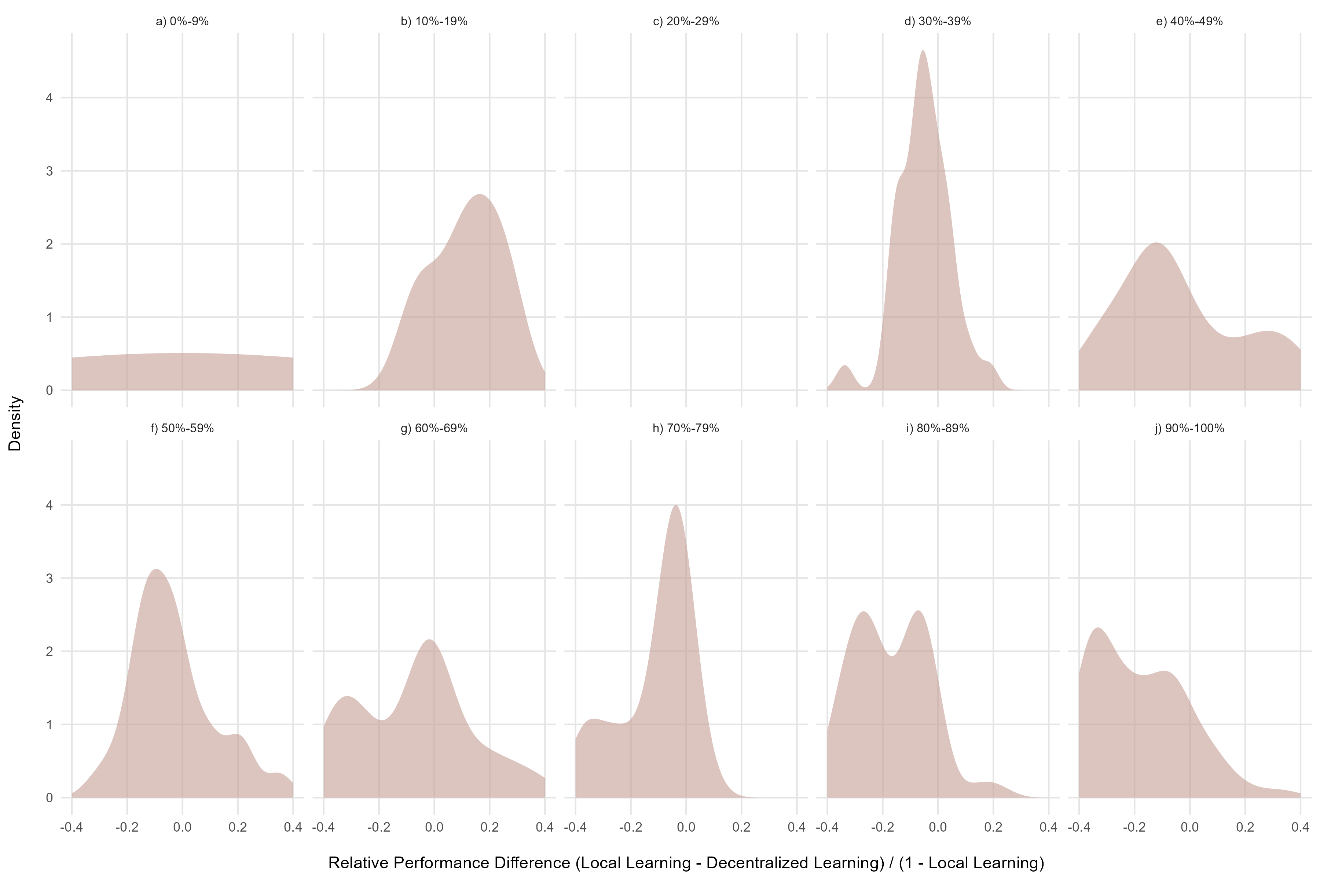


Supplementary Figure 58 - Distribution of Relative Performance Differences by Decentralized Learning Value Percentiles in F1 score, for percentiles a) [0%-10%[, b) [10%-20%[, c) [20%-30% [, d) [30%-40% [, e) [40%-50% [, f) [50%-60% [, g) [60%-70% [, h) [70%-80% [, i) [80%-90% [, j) [90%-100%].


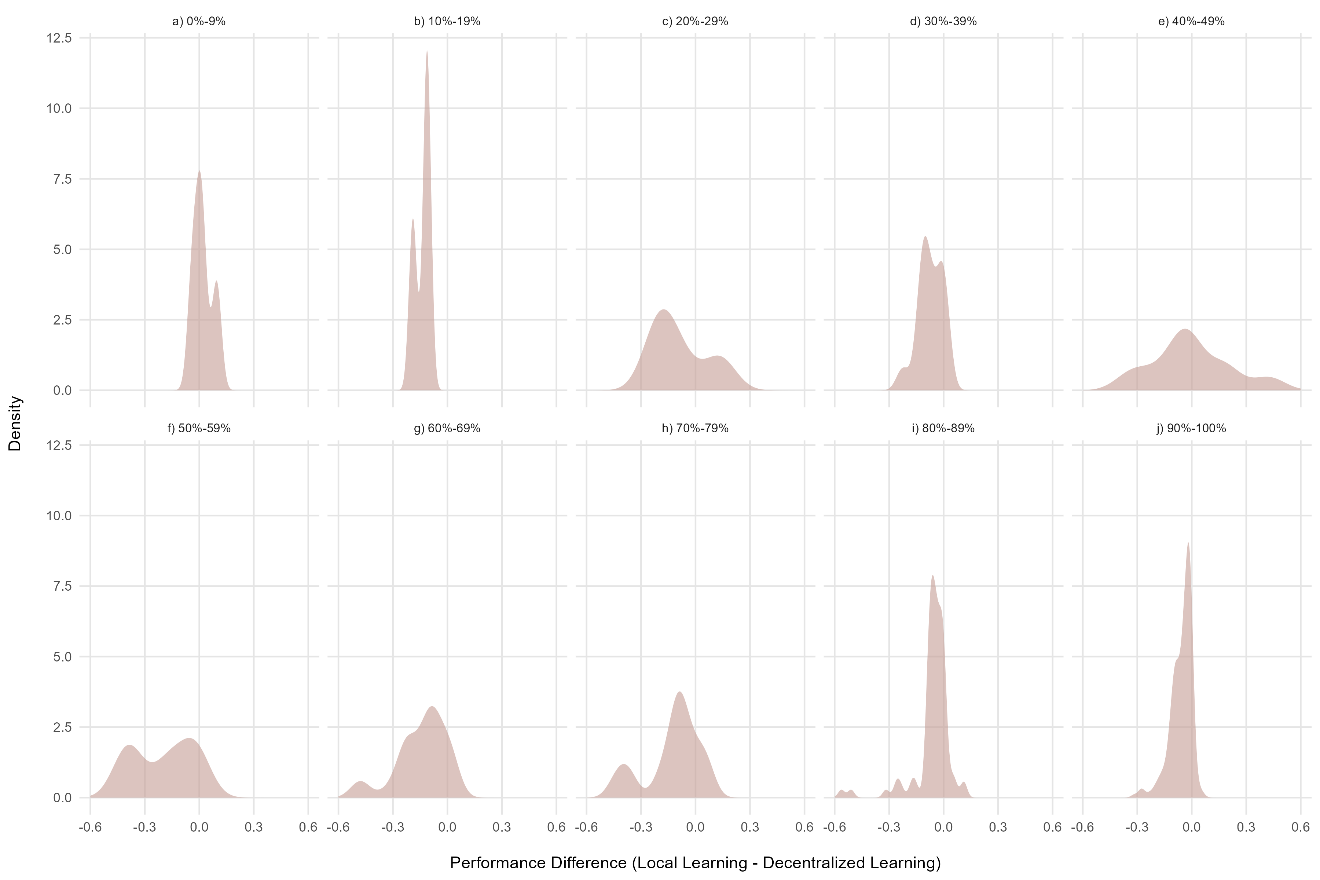


Supplementary Figure 59 - Distribution of Absolute Performance Differences by Decentralized Learning Value Percentiles in Precision / Positive Predictive Value, for percentiles a) [0%-10%[, b) [10%-20%[, c) [20%-30% [, d) [30%-40% [, e) [40%-50% [, f) [50%-60% [, g) [60%-70% [, h) [70%-80% [, i) [80%-90% [, j) [90%-100%].


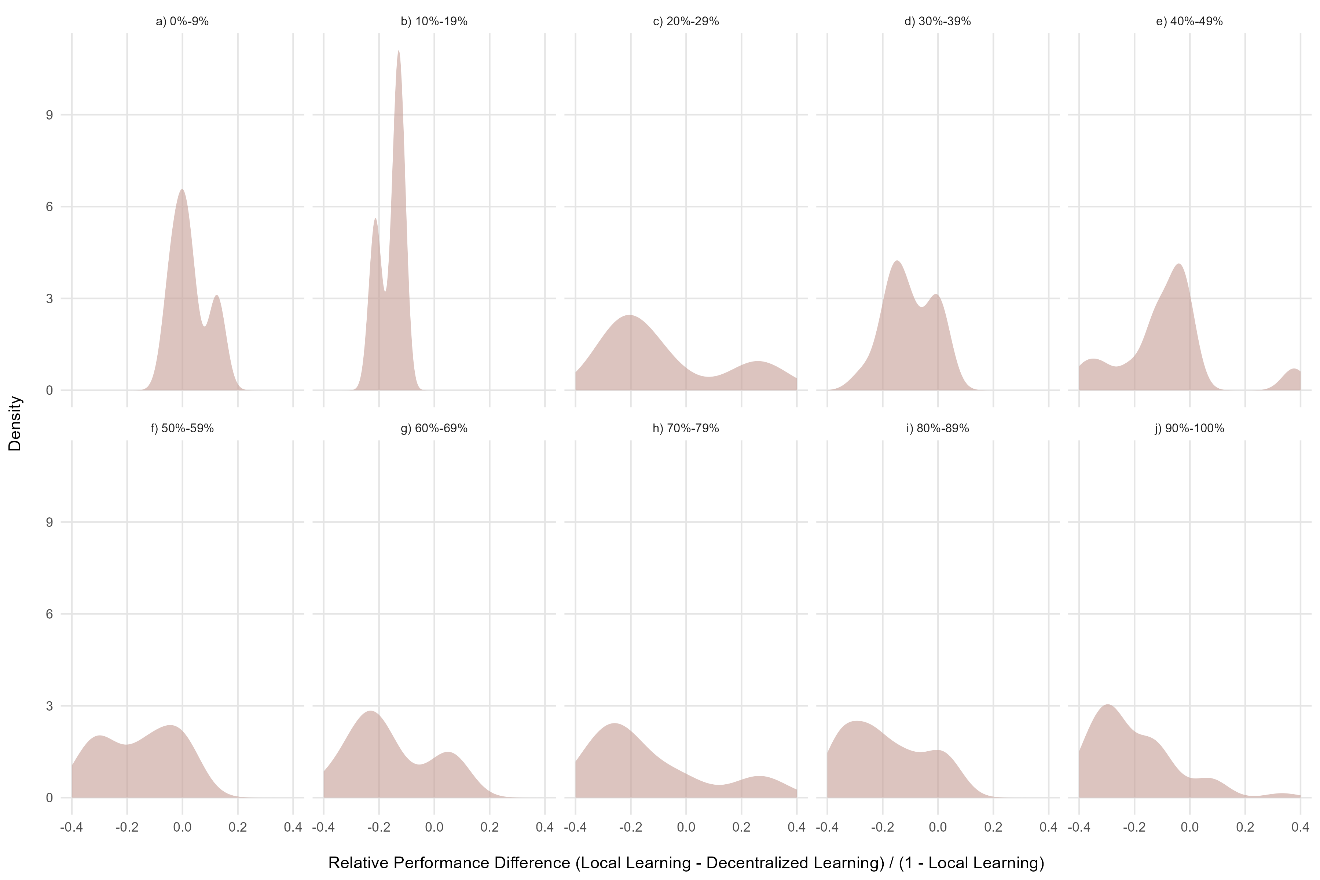


Supplementary Figure 60 - Distribution of Relative Performance Differences by Decentralized Learning Value Percentiles in Precision / Positive Predictive Value, for percentiles a) [0%-10%[, b) [10%-20%[, c) [20%-30% [, d) [30%-40% [, e) [40%-50% [, f) [50%-60% [, g) [60%-70% [, h) [70%-80% [, i) [80%-90% [, j) [90%-100%].


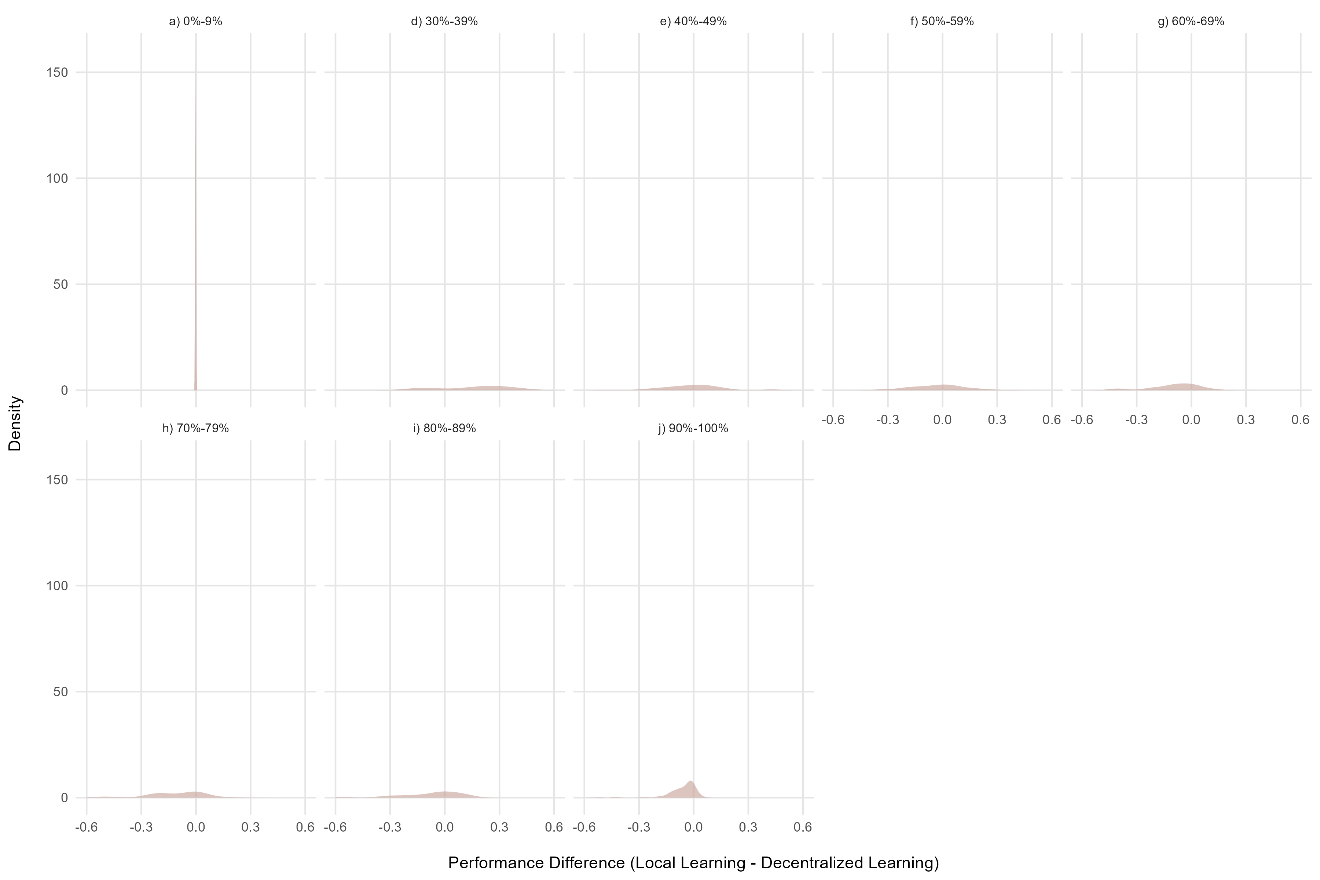


Supplementary Figure 61 - Distribution of Absolute Performance Differences by Decentralized Learning Value Percentiles in Sensitivity / Recall, for percentiles a) [0%-10%[, b) [10%-20%[, c) [20%-30% [, d) [30%-40% [, e) [40%-50% [, f) [50%-60% [, g) [60%-70% [, h) [70%-80% [, i) [80%-90% [, j) [90%-100%].


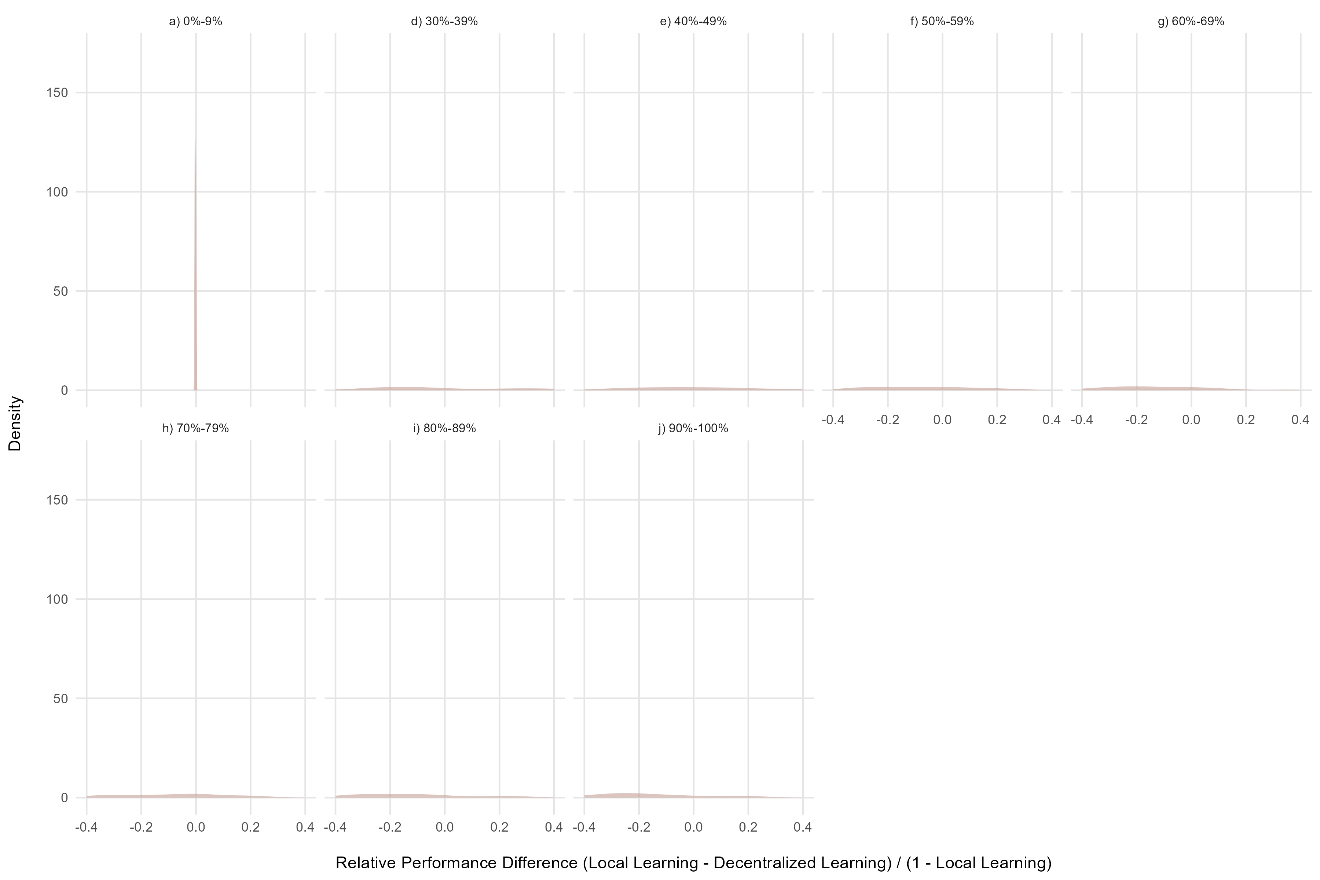


Supplementary Figure 62 - Distribution of Relative Performance Differences by Decentralized Learning Value Percentiles in Sensitivity / Recall, for percentiles a) [0%-10%[, b) [10%-20%[, c) [20%-30% [, d) [30%-40% [, e) [40%-50% [, f) [50%-60% [, g) [60%-70% [, h) [70%-80% [, i) [80%-90% [, j) [90%-100%].


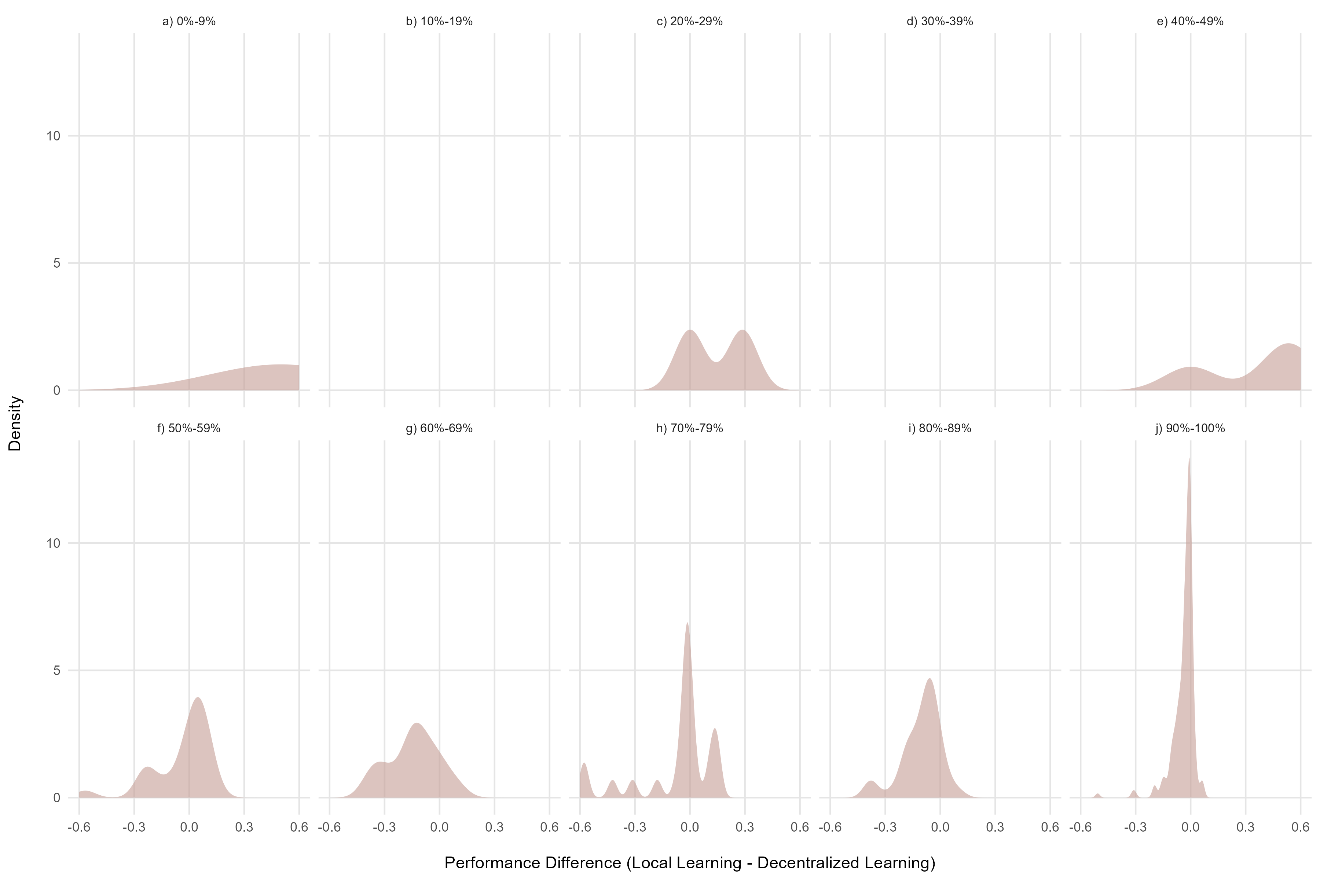


Supplementary Figure 63 - Distribution of Absolute Performance Differences by Decentralized Learning Value Percentiles in Specificity, for percentiles a) [0%-10%[, b) [10%-20%[, c) [20%-30% [, d) [30%-40% [, e) [40%-50% [, f) [50%-60% [, g) [60%-70% [, h) [70%-80% [, i) [80%-90% [, j) [90%-100%].


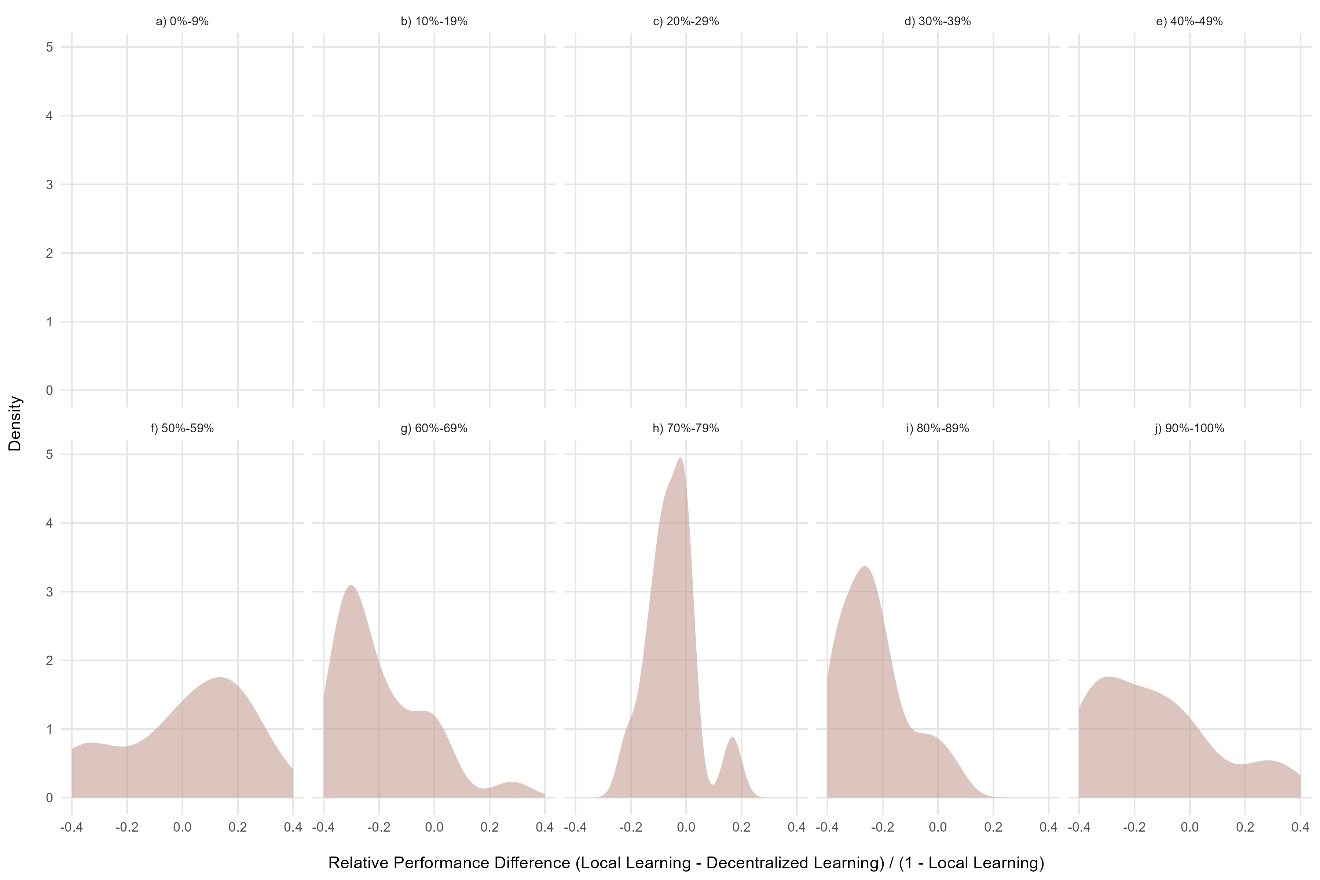


Supplementary Figure 64 - Distribution of Relative Performance Differences by Decentralized Learning Value Percentiles in Specificity, for percentiles a) [0%-10%[, b) [10%-20%[, c) [20%-30% [, d) [30%-40% [, e) [40%-50% [, f) [50%-60% [, g) [60%-70% [, h) [70%-80% [, i) [80%-90% [, j) [90%-100%].

## Sensitivity Analyses


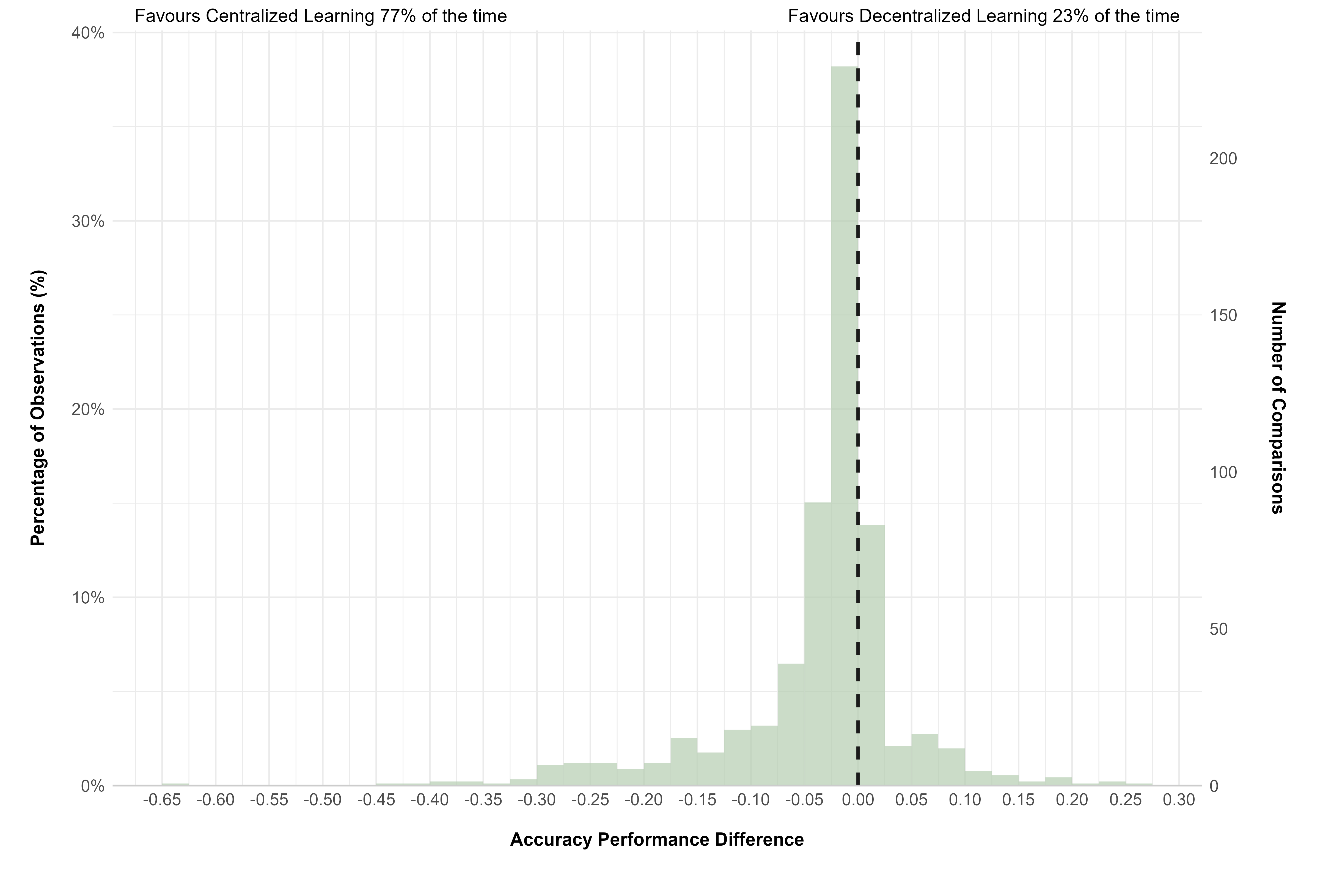


Supplementary Figure 65 - Sensitivity Analysis of Distribution of Individual Model Performance Differences - Across Accuracy (Comparing Decentralized Learning versus Centralized Learning). Based on 1089 observations extracted from 212 models of 66 studies. Summary Results: 25th Percentile = -0.0364 | 75th Percentile = 0.0000 | Mean difference: -0.0012. Note: Dashed vertical line indicates no difference in performance between compared approaches.


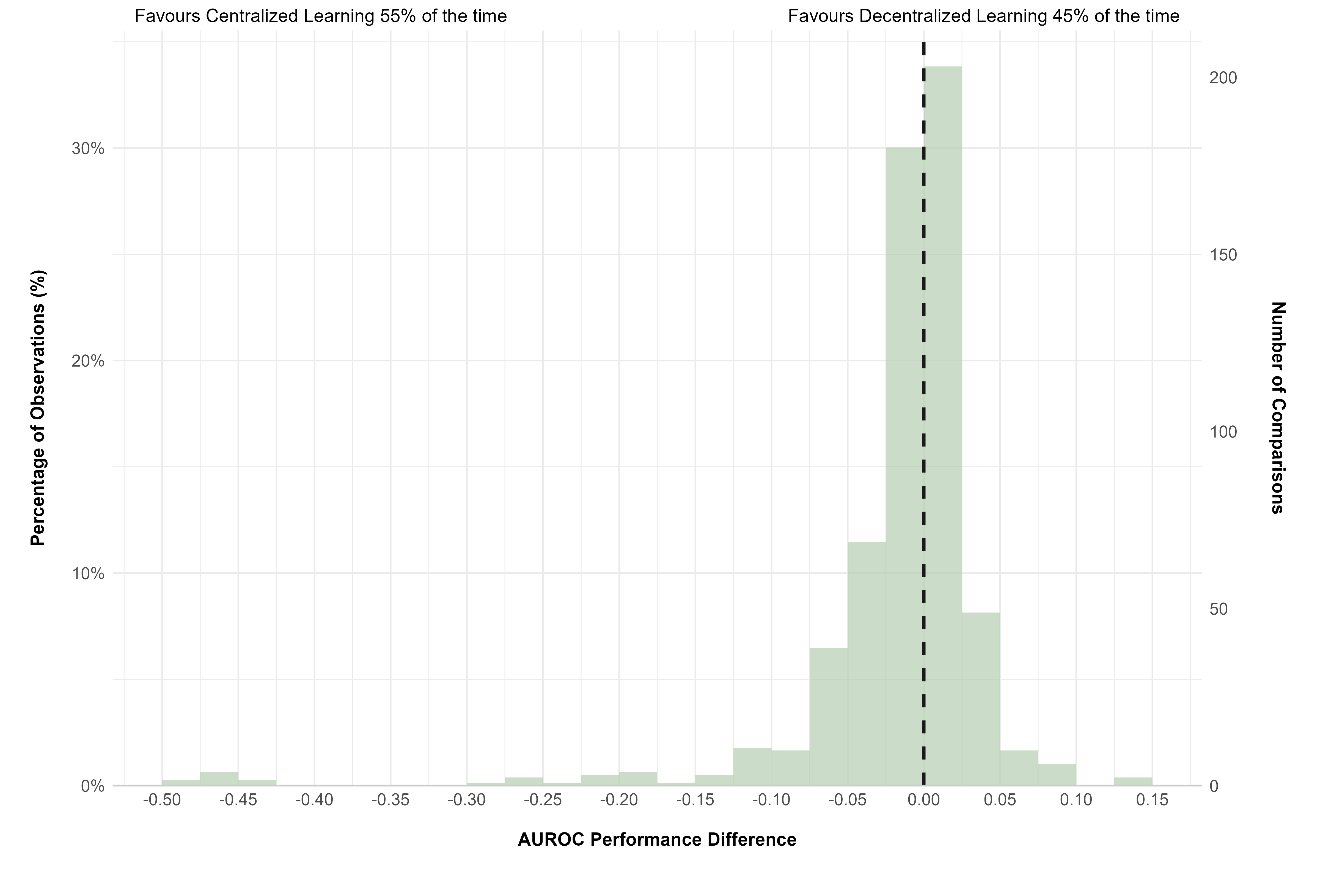


Supplementary Figure 66 - Sensitivity Analysis of Distribution of Individual Model Performance Differences - Across AUROC (Comparing Decentralized Learning versus Centralized Learning). Based on 1063 observations extracted from 212 models of 42 studies. Summary Results: 25th Percentile = -0.0287 | 75th Percentile = 0.0190 | Mean difference: -0.0012. Note: Dashed vertical line indicates no difference in performance between compared approaches.


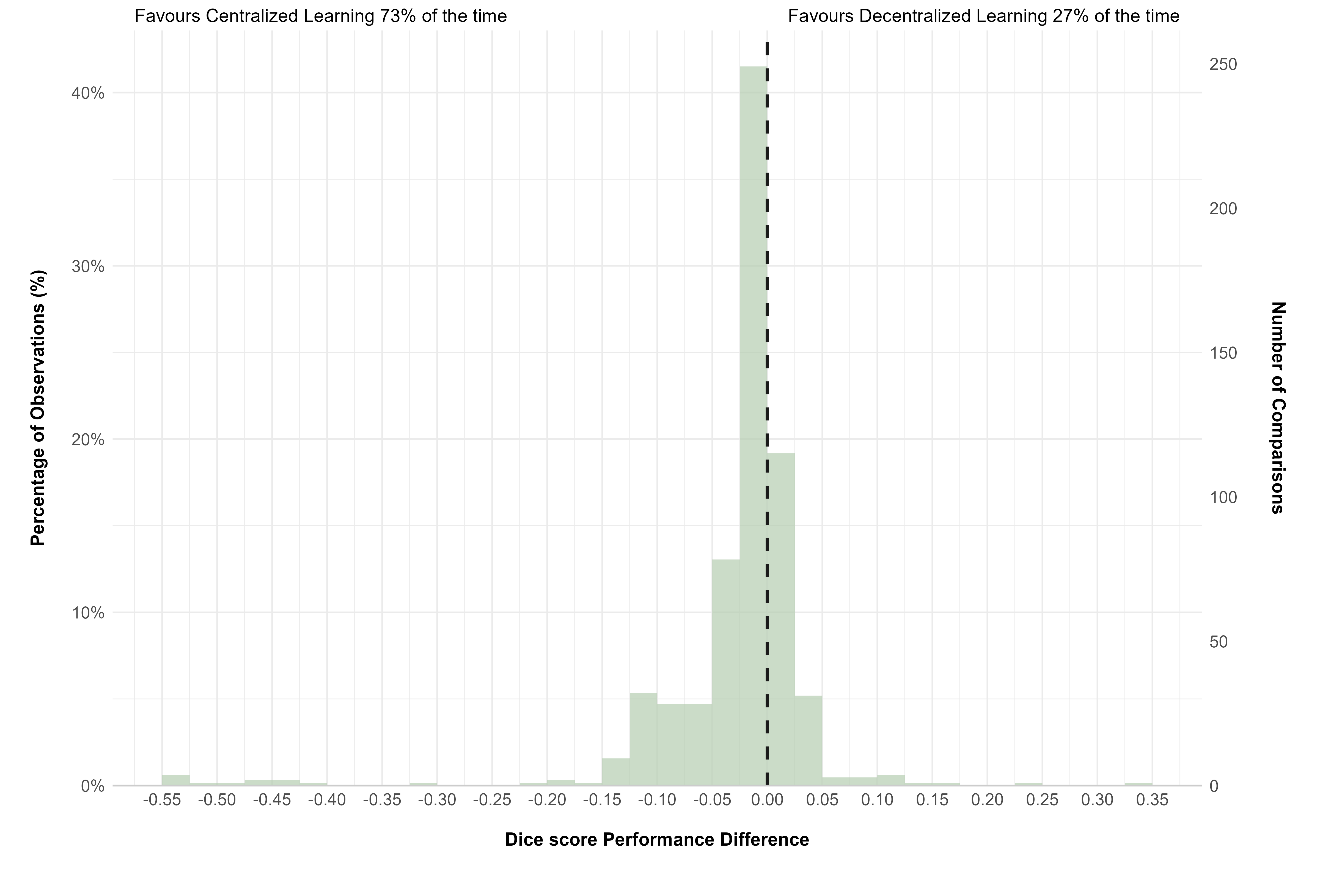


Supplementary Figure 67 - Sensitivity Analysis of Distribution of Individual Model Performance Differences - Across Dice score (Comparing Decentralized Learning versus Centralized Learning). Based on 856 observations extracted from 127 models of 24 studies. Summary Results: 25th Percentile = -0.0411 | 75th Percentile = -0.0021 | Mean difference: -0.0012. Note: Dashed vertical line indicates no difference in performance between compared approaches.


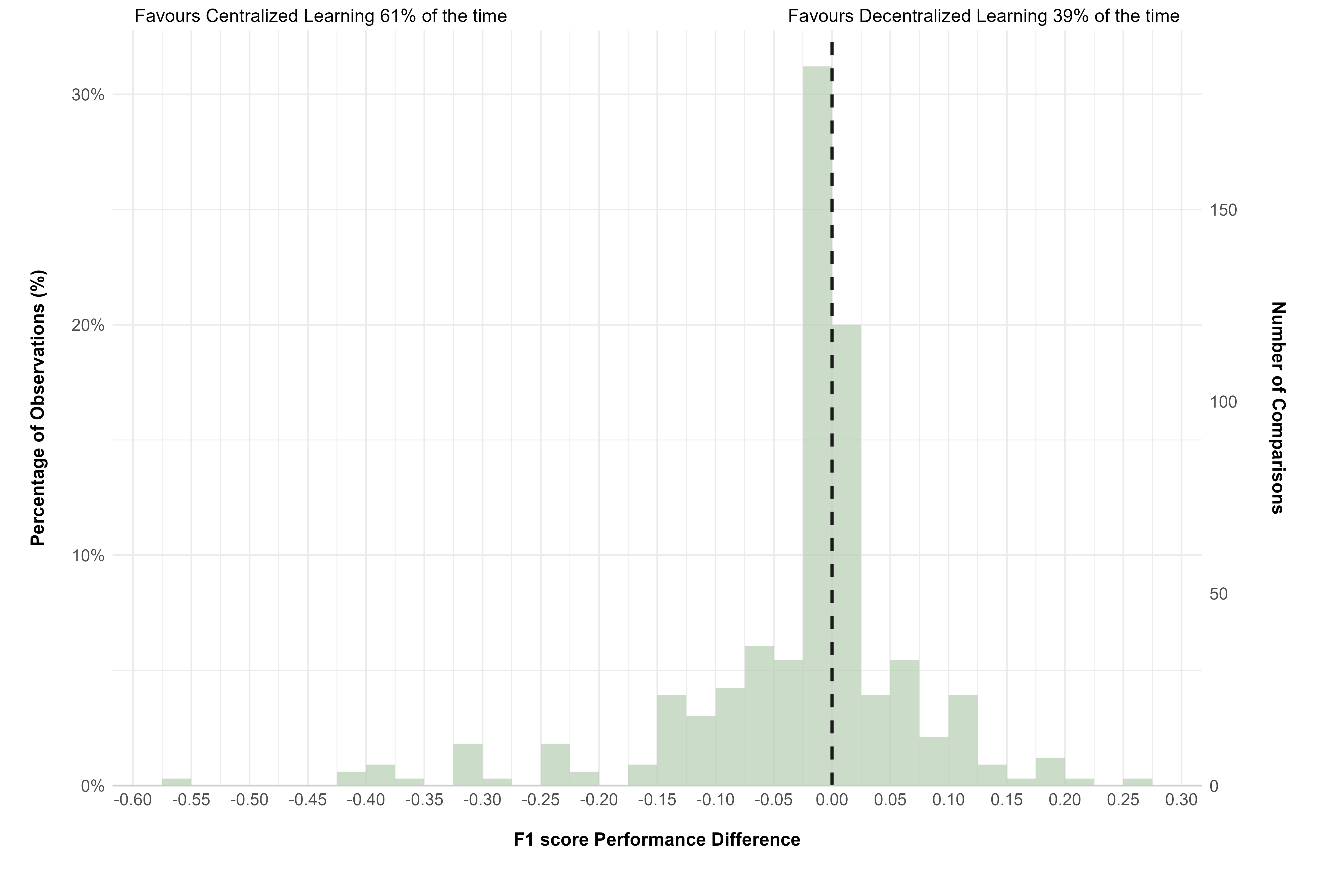


Supplementary Figure 68 - Sensitivity Analysis of Distribution of Individual Model Performance Differences - Across F1 score (Comparing Decentralized Learning versus Centralized Learning). Based on 420 observations extracted from 98 models of 32 studies. Summary Results: 25th Percentile = -0.0825 | 75th Percentile = 0.0034 | Mean difference: -0.0012. Note: Dashed vertical line indicates no difference in performance between compared approaches.


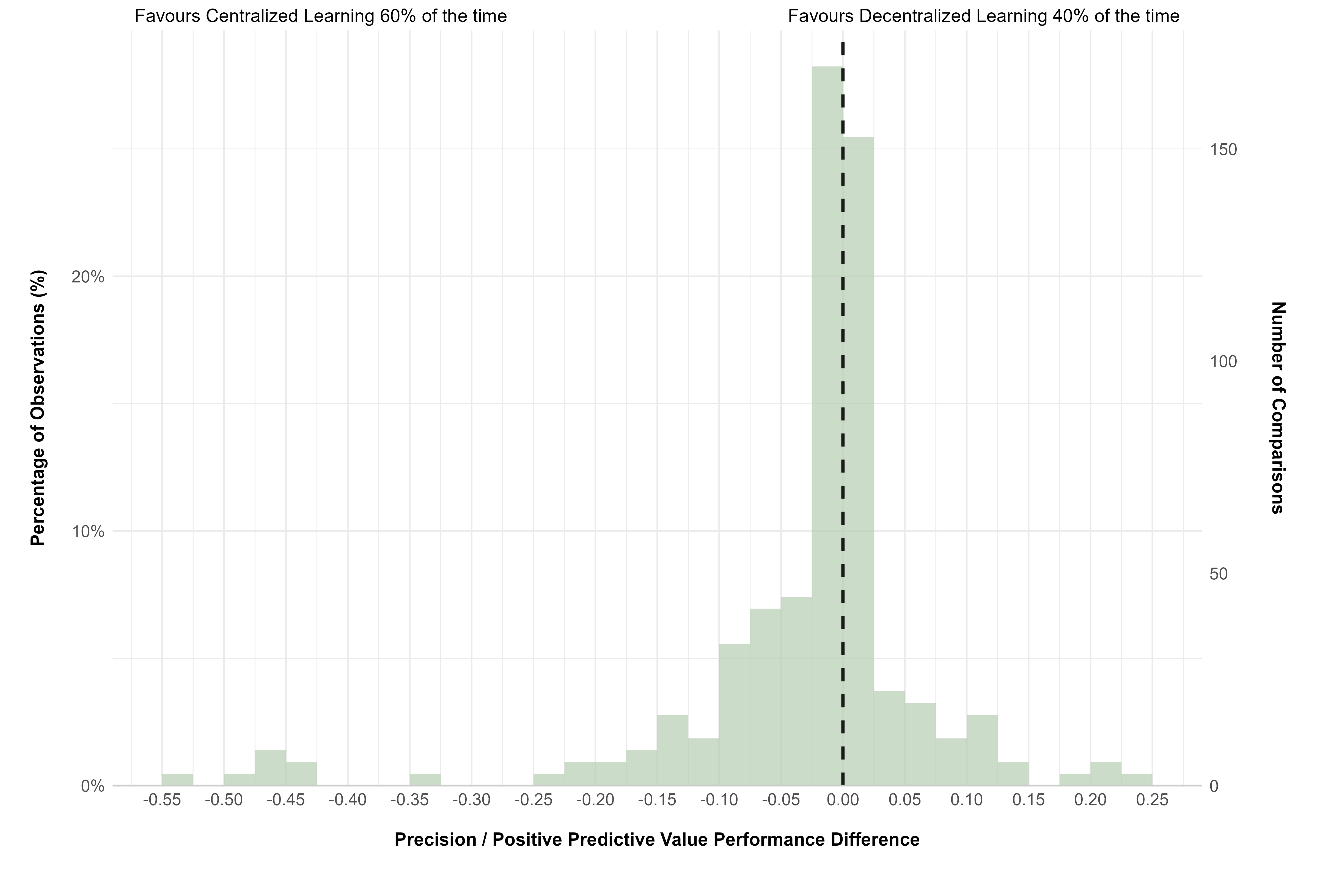


Supplementary Figure 69 - Sensitivity Analysis of Distribution of Individual Model Performance Differences - Across Precision / Positive Predictive Value (Comparing Decentralized Learning versus Centralized Learning). Based on 258 observations extracted from 80 models of 29 studies. Summary Results: 25th Percentile = -0.0640 | 75th Percentile = 0.0080 | Mean difference: -0.0012. Note: Dashed vertical line indicates no difference in performance between compared approaches.


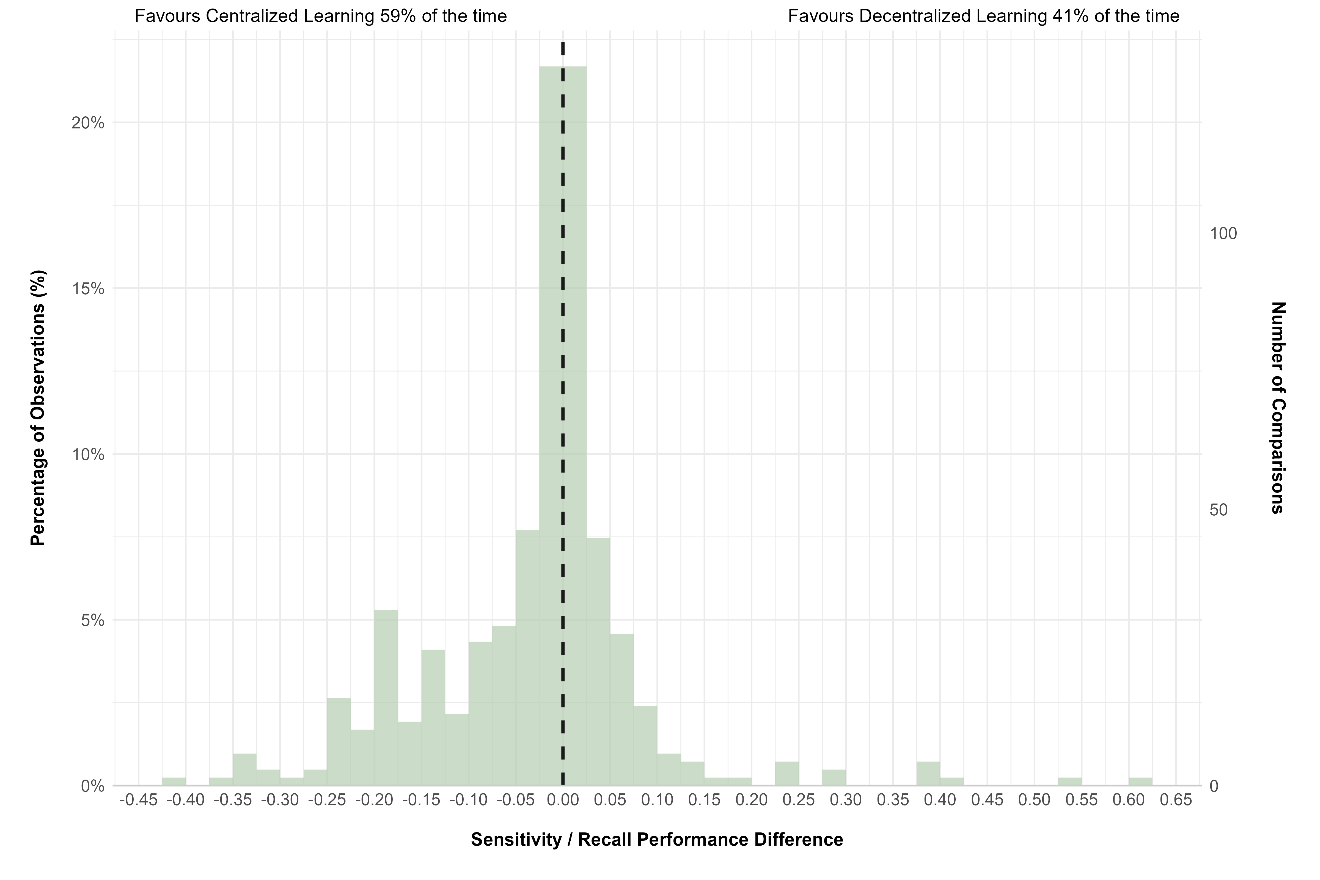


Supplementary Figure 70 - Sensitivity Analysis of Distribution of Individual Model Performance Differences - Across Sensitivity / Recall (Comparing Decentralized Learning versus Centralized Learning). Based on 593 observations extracted from 120 models of 42 studies. Summary Results: 25th Percentile = -0.0880 | 75th Percentile = 0.0100 | Mean difference: -0.0012. Note: Dashed vertical line indicates no difference in performance between compared approaches.


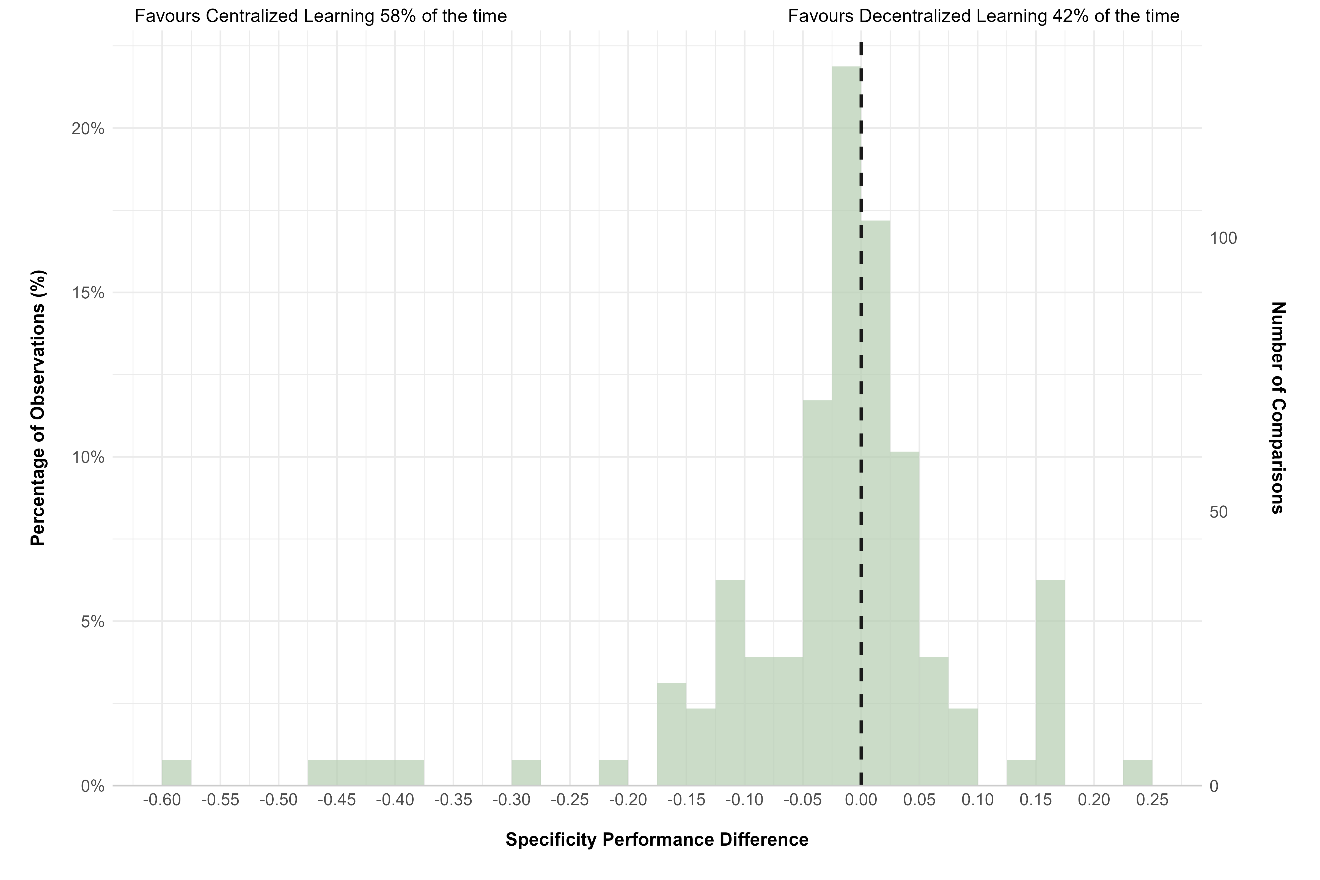


Supplementary Figure 71 - Sensitivity Analysis of Distribution of Individual Model Performance Differences - Across Specificity (Comparing Decentralized Learning versus Centralized Learning). Based on 160 observations extracted from 46 models of 20 studies. Summary Results: 25th Percentile = -0.0295 | 75th Percentile = 0.0113 | Mean difference: -0.0012. Note: Dashed vertical line indicates no difference in performance between compared approaches.


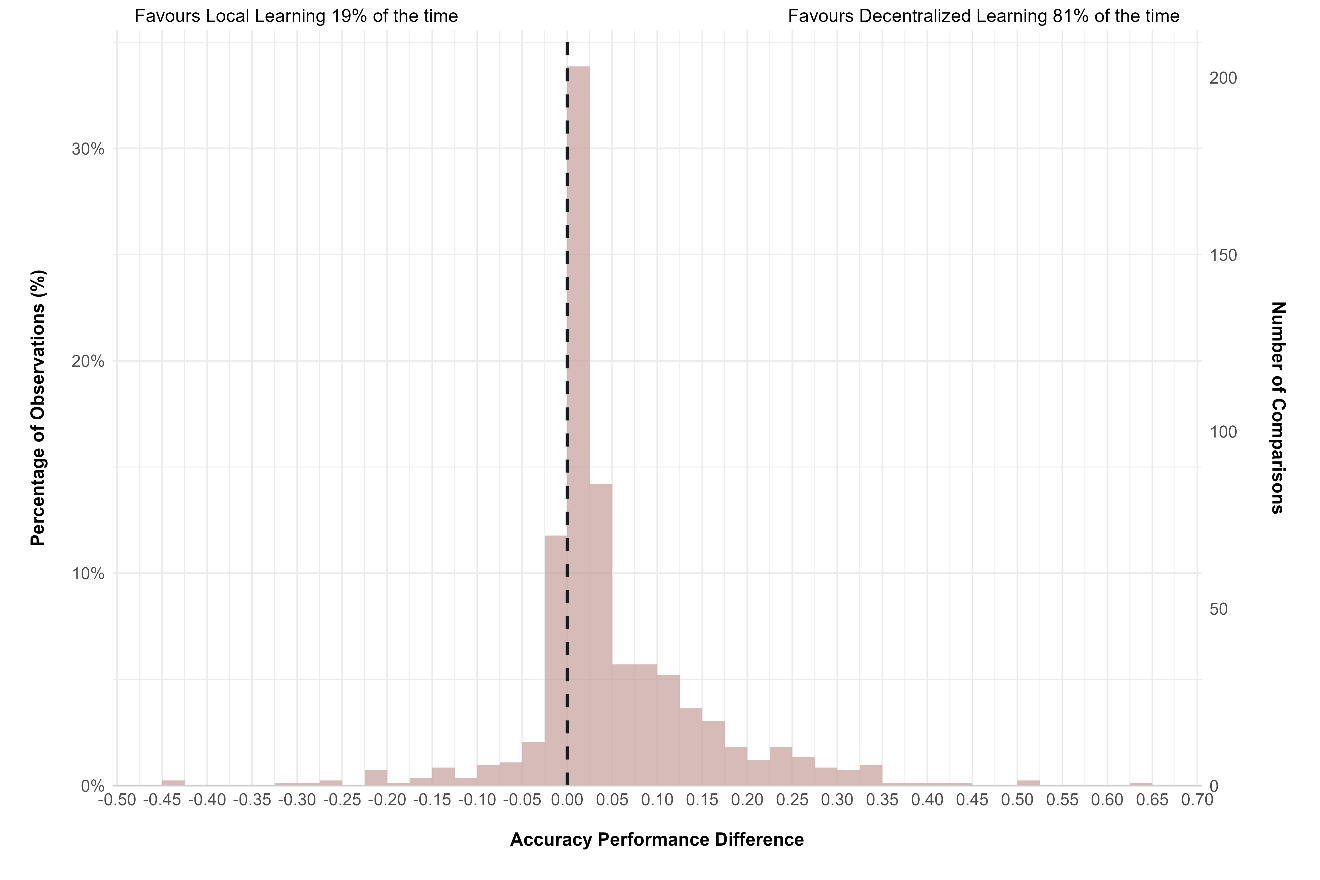


Supplementary Figure 72 - Sensitivity Analysis of Distribution of Individual Model Performance Differences - Across Accuracy (Comparing Decentralized Learning versus Local Learning). Based on 1024 observations extracted from 139 models of 46 studies. Summary Results: 25th Percentile = 0.0042 | 75th Percentile = 0.0772 | Mean difference: -0.0012. Note: Dashed vertical line indicates no difference in performance between compared approaches.


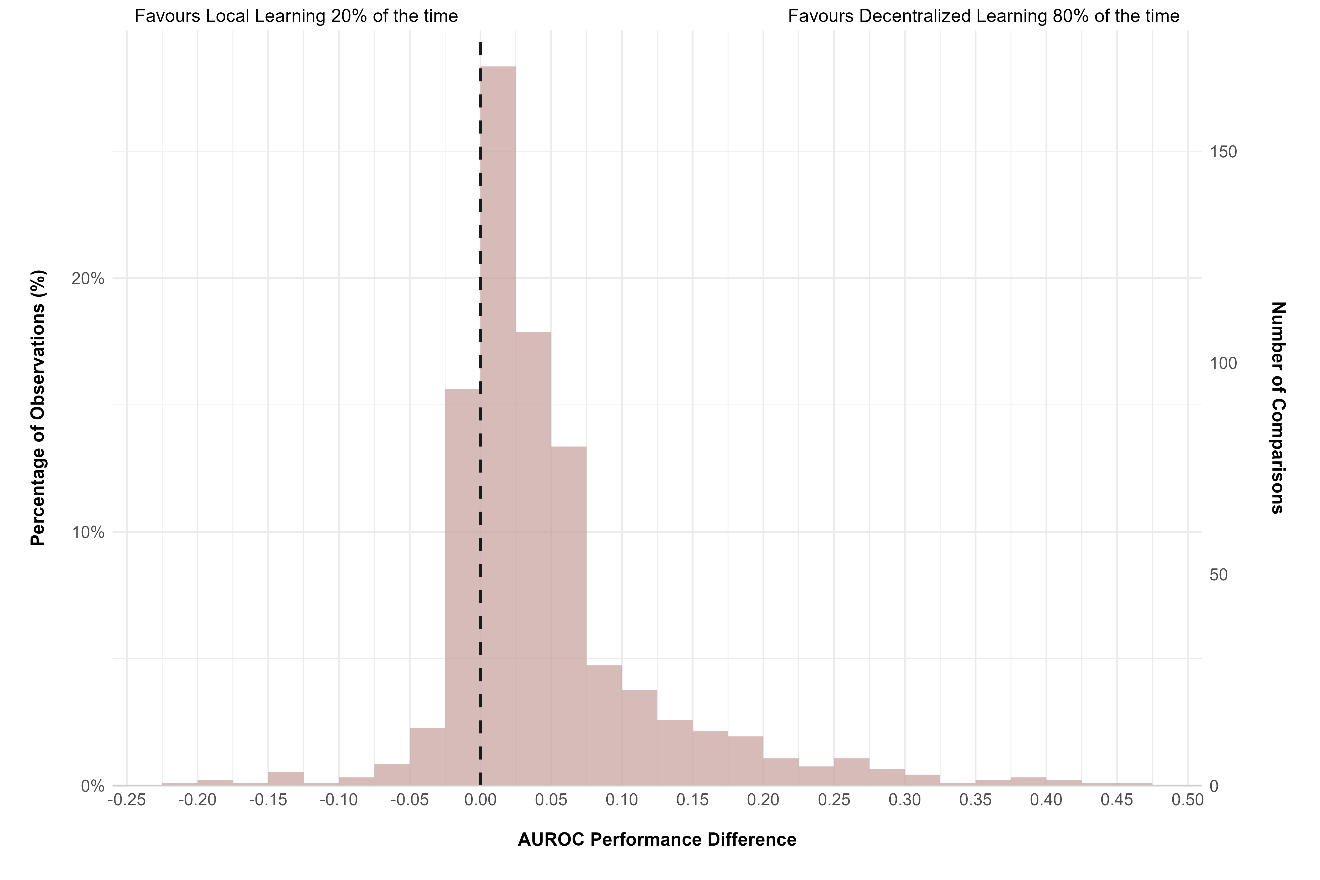


Supplementary Figure 73 - Sensitivity Analysis of Distribution of Individual Model Performance Differences - Across AUROC (Comparing Decentralized Learning versus Local Learning). Based on 1059 observations extracted from 140 models of 39 studies. Summary Results: 25th Percentile = 0.0033 | 75th Percentile = 0.0608 | Mean difference: -0.0012. Note: Dashed vertical line indicates no difference in performance between compared approaches.


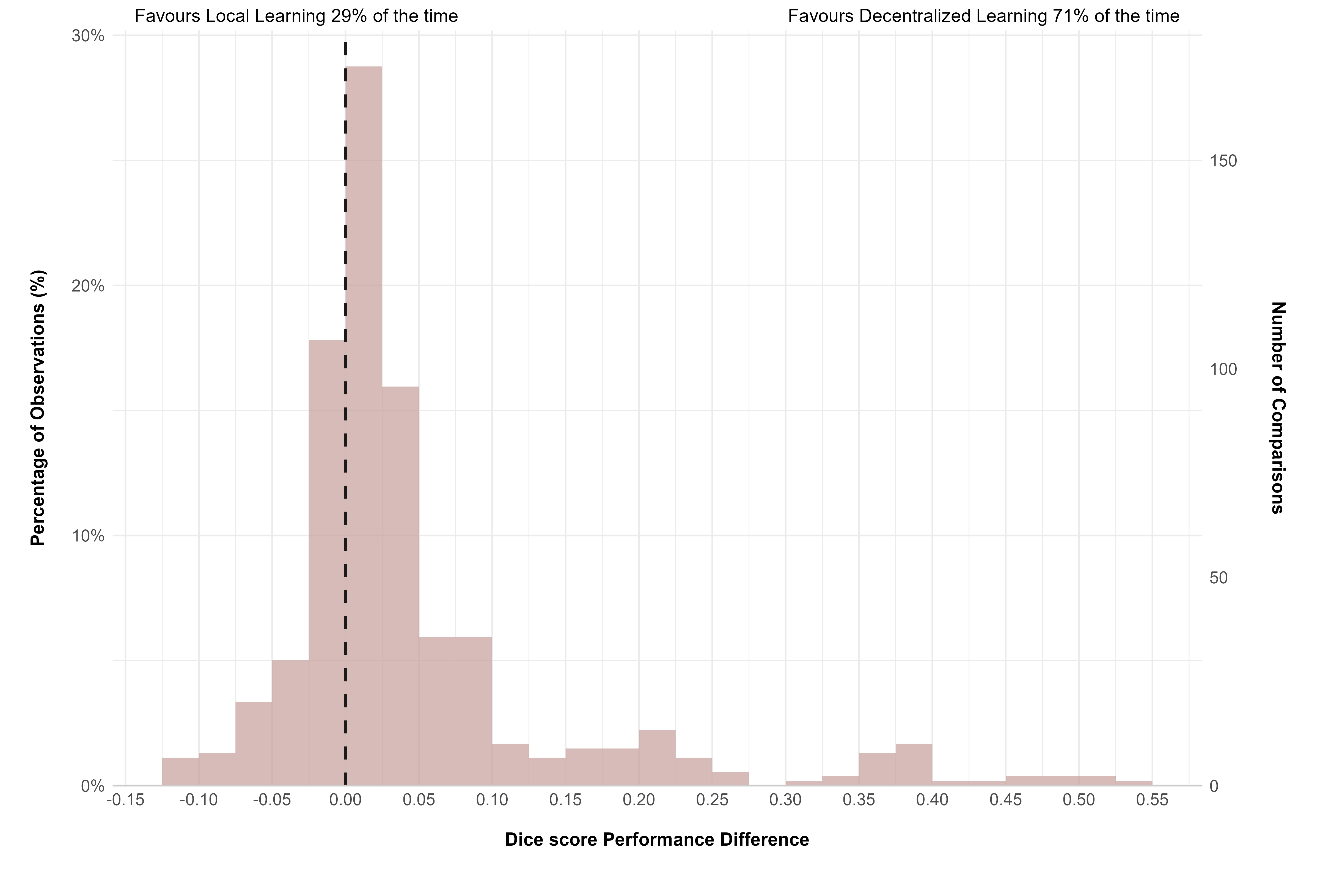


Supplementary Figure 74 - Sensitivity Analysis of Distribution of Individual Model Performance Differences - Across Dice score (Comparing Decentralized Learning versus Local Learning). Based on 759 observations extracted from 74 models of 18 studies. Summary Results: 25th Percentile = -0.0020 | 75th Percentile = 0.0935 | Mean difference: -0.0012. Note: Dashed vertical line indicates no difference in performance between compared approaches.


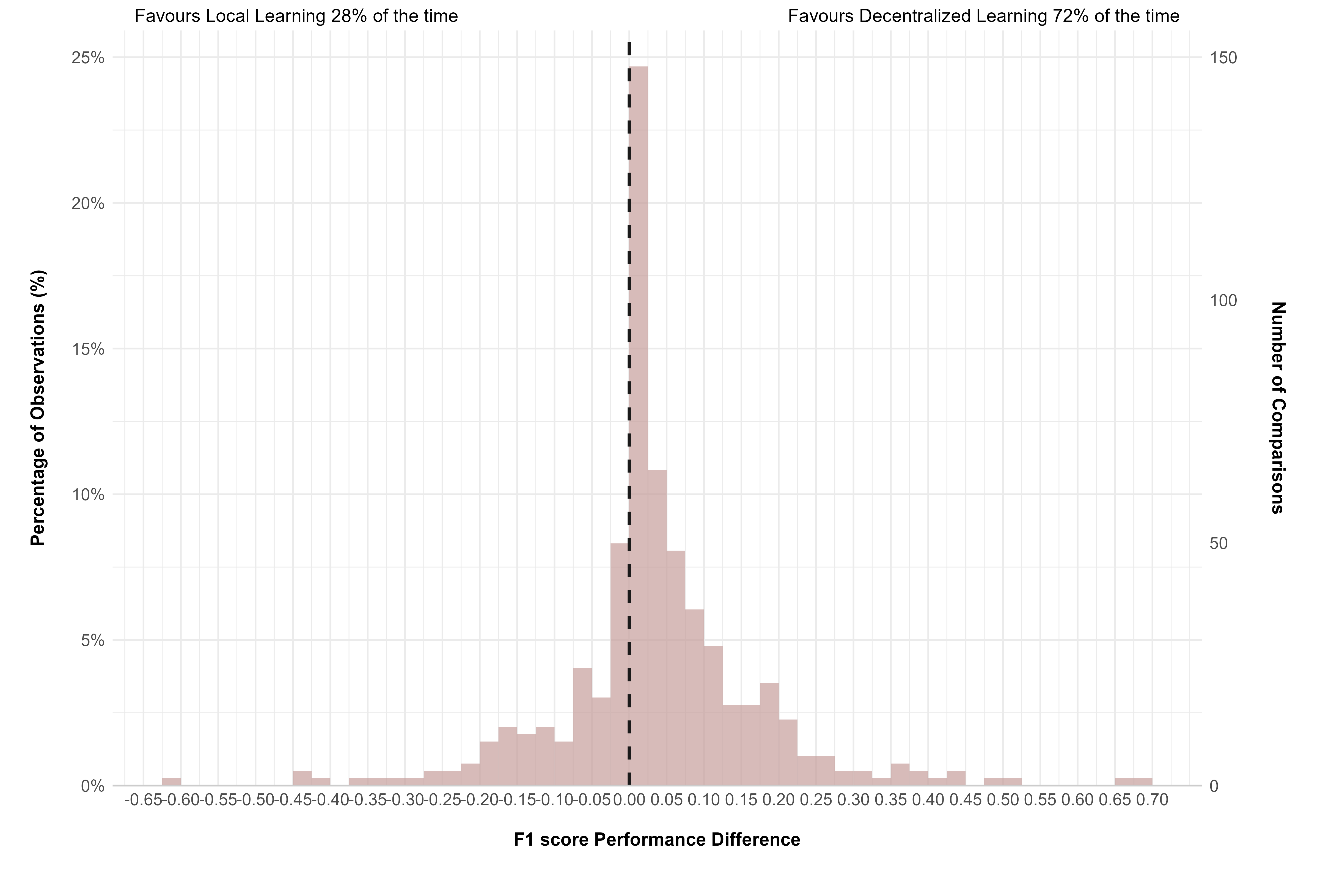


Supplementary Figure 75 - Sensitivity Analysis of Distribution of Individual Model Performance Differences - Across F1 score (Comparing Decentralized Learning versus Local Learning). Based on 593 observations extracted from 74 models of 27 studies. Summary Results: 25th Percentile = 0.0040 | 75th Percentile = 0.0955 | Mean difference: -0.0012. Note: Dashed vertical line indicates no difference in performance between compared approaches.


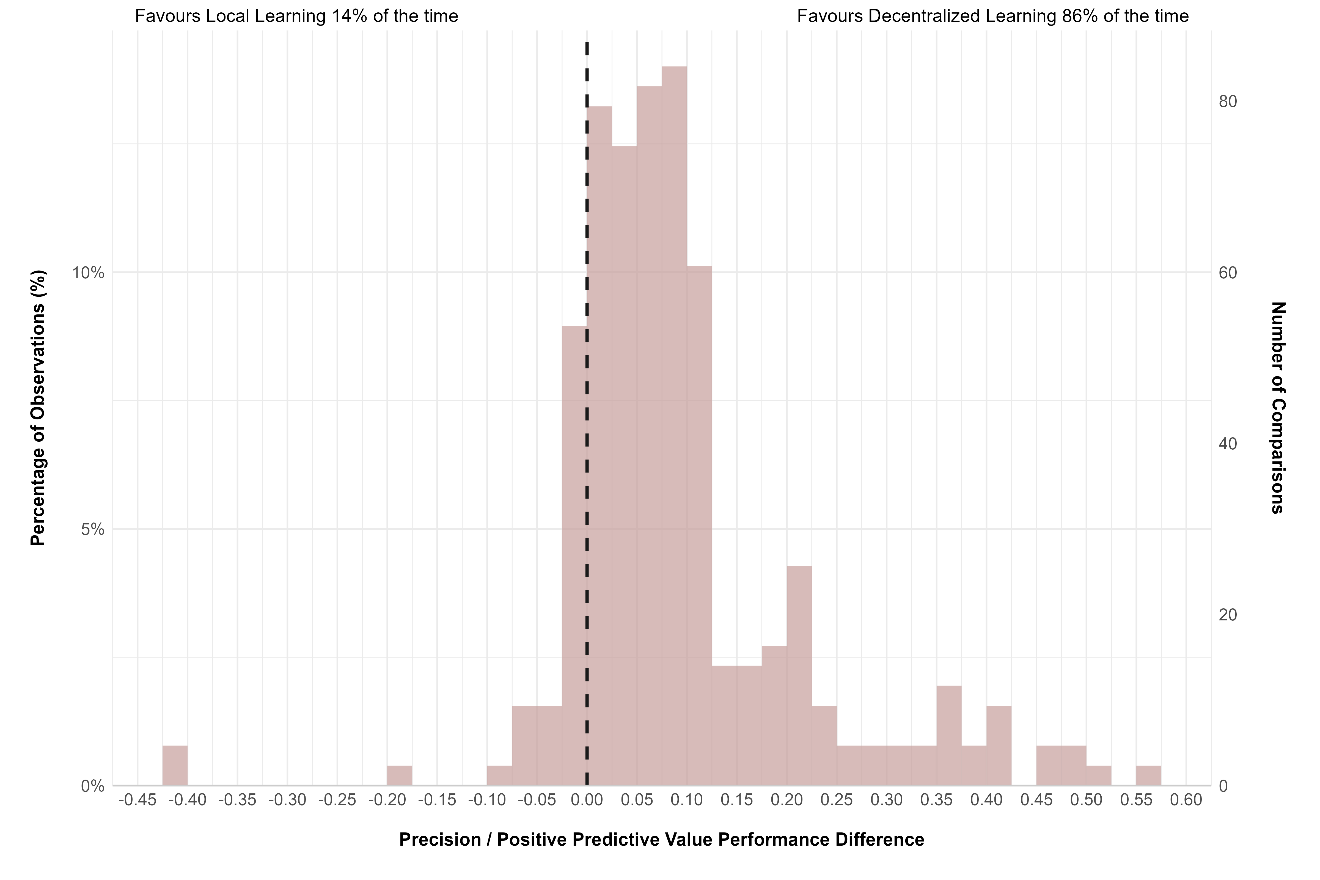


Supplementary Figure 76 - Sensitivity Analysis of Distribution of Individual Model Performance Differences - Across Precision / Positive Predictive Value (Comparing Decentralized Learning versus Local Learning). Based on 442 observations extracted from 60 models of 23 studies. Summary Results: 25th Percentile = 0.0120 | 75th Percentile = 0.1090 | Mean difference: -0.0012. Note: Dashed vertical line indicates no difference in performance between compared approaches.


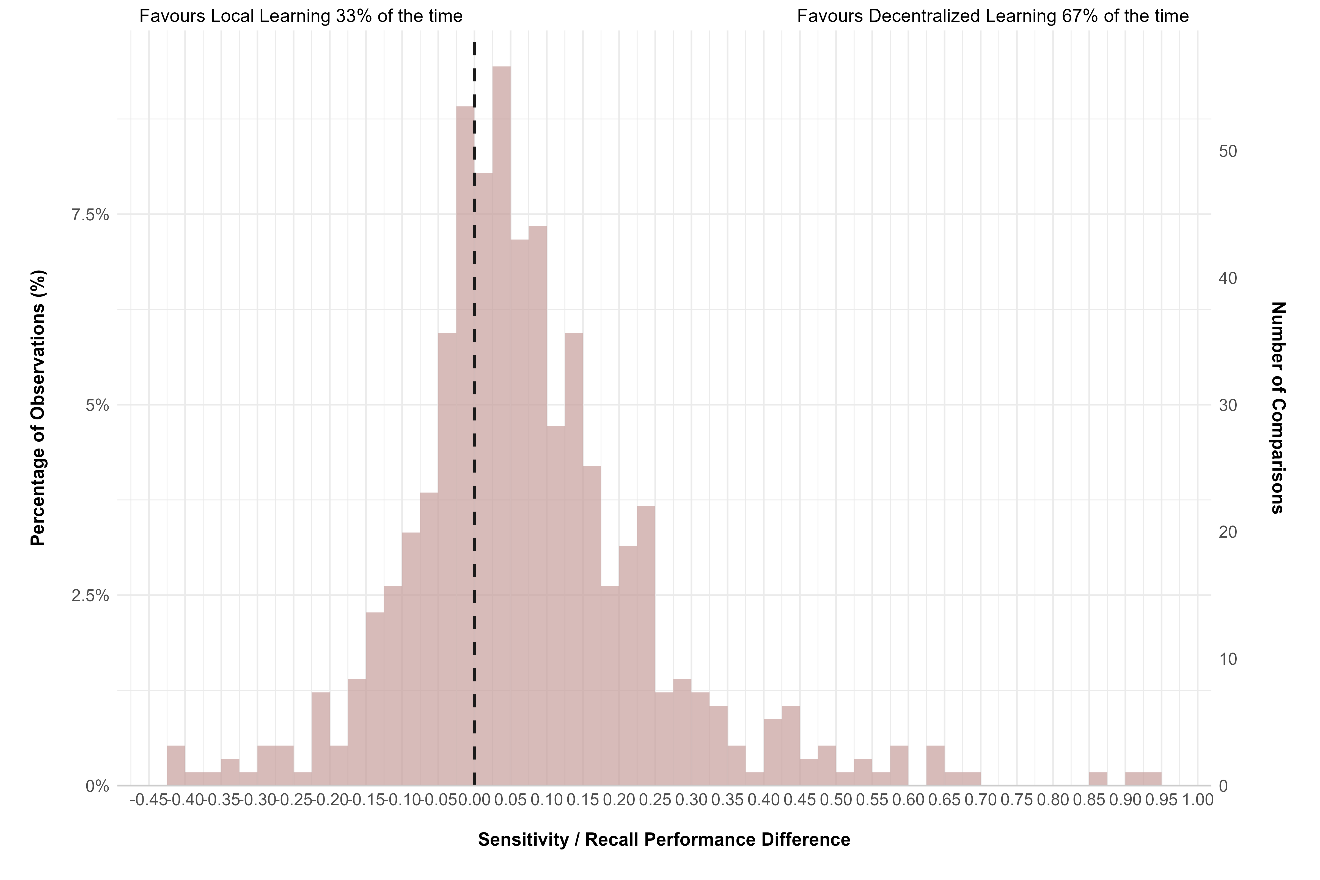


Supplementary Figure 77 - Sensitivity Analysis of Distribution of Individual Model Performance Differences - Across Sensitivity / Recall (Comparing Decentralized Learning versus Local Learning). Based on 772 observations extracted from 96 models of 35 studies. Summary Results: 25th Percentile = -0.0093 | 75th Percentile = 0.1427 | Mean difference: -0.0012. Note: Dashed vertical line indicates no difference in performance between compared approaches.


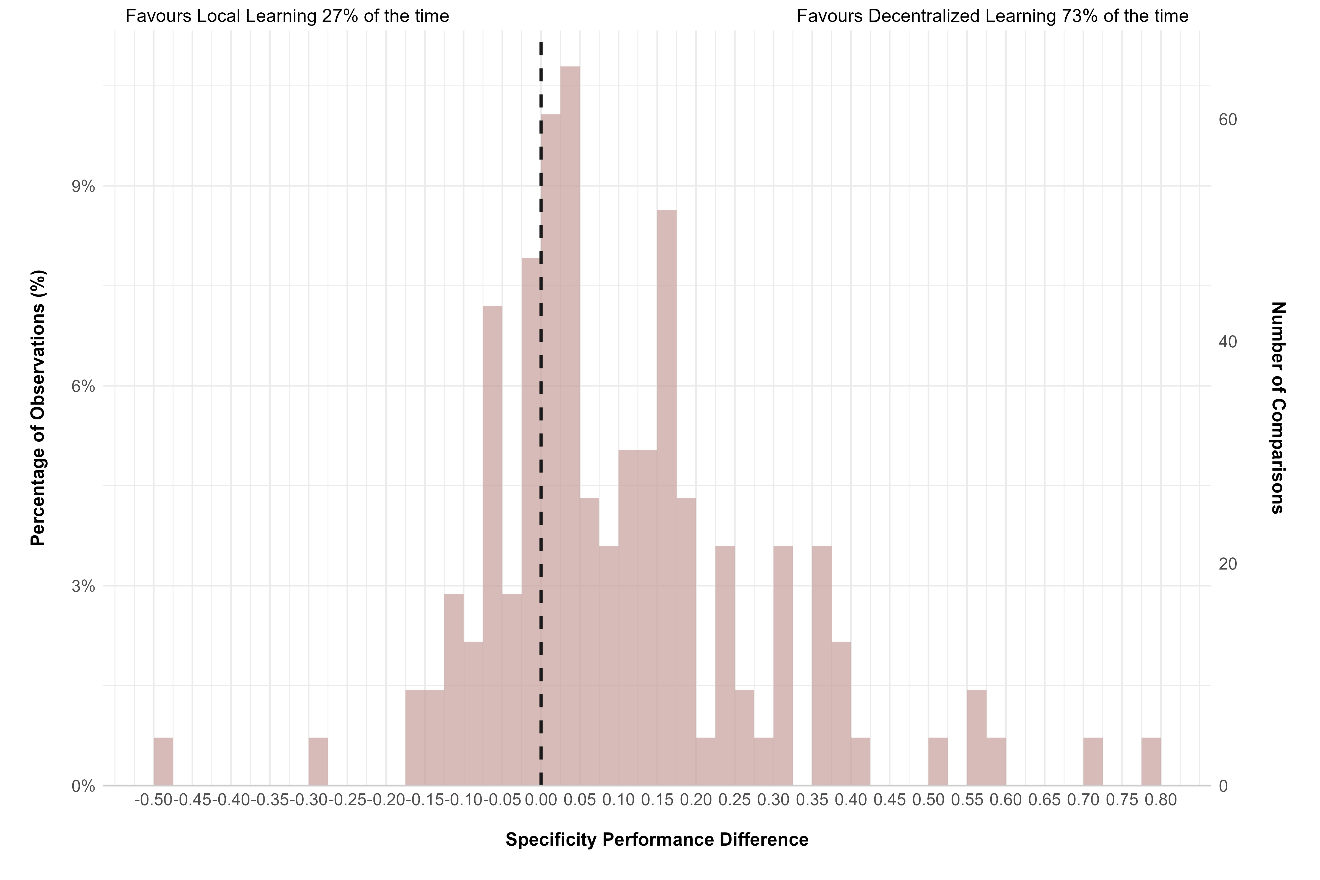


Supplementary Figure 78 - Sensitivity Analysis of Distribution of Individual Model Performance Differences - Across Specificity (Comparing Decentralized Learning versus Local Learning). Based on 339 observations extracted from 47 models of 16 studies. Summary Results: 25th Percentile = 0.0021 | 75th Percentile = 0.0898 | Mean difference: -0.0012. Note: Dashed vertical line indicates no difference in performance between compared approaches.

## PRISMA 2020 Checklist

| **Section and Topic** | **Item #** | **Checklist item** | **Location where item is reported** |
| --- | --- | --- | --- |
| **TITLE** | | |  |
| Title | 1 | Identify the report as a systematic review. | The title clearly identifies this as "A Systematic Review": *"Comparing Decentralized Machine Learning and AI Clinical Models to Local and Centralized Alternatives: A Systematic Review"*. |
| **ABSTRACT** | | |  |
| Abstract | 2 | See the PRISMA 2020 for Abstracts checklist. | The structured abstract includes all key elements: objective, methods (search strategy, eligibility criteria), results (study numbers, key findings), and conclusions. The abstract mentions searching "eight databases (01/2012 to 03/2024), screening 165,010 studies with two independent reviewers" and provides quantitative results. |
| **INTRODUCTION** | | |  |
| Rationale | 3 | Describe the rationale for the review in the context of existing knowledge. | Described in the Introduction (pp. 1-2): Comprehensive rationale provided across multiple paragraphs discussing healthcare challenges, the role of AI/ML, data privacy barriers, and the emergence of decentralized learning as a solution. The authors clearly establish the context for this review. |
| Objectives | 4 | Provide an explicit statement of the objective(s) or question(s) the review addresses. | Described in the Introduction (p. 2): “This systematic literature review seeks to compare the performance of health data models developed using decentralized learning approaches (e.g., federated learning, swarm learning, ensemble) with those developed using traditional centralized or local methods, as the primary objective. The performance comparison is grouped using the metrics reported in the original articles (e.g., accuracy, precision, AUROC), covering a wide range of medical conditions (e.g., COVID-19, breast cancer, type 2 Diabetes), through different clinical tasks (e.g., diagnosis, segmentation, prognosis). Secondary objectives include describing the types of data and datasets used, the nature of the decentralized model architectures, and the reporting of resource demands or privacy impacts.” |
| **METHODS** | | |  |
| Eligibility criteria | 5 | Specify the inclusion and exclusion criteria for the review and how studies were grouped for the syntheses. | Detailed inclusion and exclusion criteria provided in the "Eligibility Criteria" section, including: 1) original published research articles, 2) clinical decisions for human medical conditions, 3) decentralized learning methods, 4) comparison against centralized/local methods, 5) numeric performance reporting. |
| Information sources | 6 | Specify all databases, registers, websites, organisations, reference lists and other sources searched or consulted to identify studies. Specify the date when each source was last searched or consulted. | Presented in the “Information Sources”: Eleven databases listed with specific search dates: "searches were conducted in two moments... first moment... April 6th and April 7^th^, 2023. The second moment targeted articles from April 6th, 2023 to those available on March 28th, 2024." |
| Search strategy | 7 | Present the full search strategies for all databases, registers and websites, including any filters and limits used. | Full search strategy presented in Table 8 and detailed in Supplementary Material. The authors describe using regular expressions for filtration and provide comprehensive search terms grouped by categories. |
| Selection process | 8 | Specify the methods used to decide whether a study met the inclusion criteria of the review, including how many reviewers screened each record and each report retrieved, whether they worked independently, and if applicable, details of automation tools used in the process. | Described in the “Selection Process” section: "papers retrieved through the search strategy were evaluated by researchers acting independently and blinded for each other's decisions. Each paper was classified by two researchers, with a total of seven reviewers." Rayyan platform used, consensus process described. |
| Data collection process | 9 | Specify the methods used to collect data from reports, including how many reviewers collected data from each report, whether they worked independently, any processes for obtaining or confirming data from study investigators, and if applicable, details of automation tools used in the process. | Described in the “Data Collection Process” section: "Data were collected from the full-text version of the selected articles by two researchers, using a prepared online document piloted before its implementation. Researchers worked on different articles and discussed any doubts..." |
| Data items | 10a | List and define all outcomes for which data were sought. Specify whether all results that were compatible with each outcome domain in each study were sought (e.g. for all measures, time points, analyses), and if not, the methods used to decide which results to collect. | Described in the “Selection Process” section: " model performance comparisons were gathered based on written numeric data in the manuscript text or within tables, graphs, and figures, if the numeric information and the model they represent were clear. Efforts were made to also include data from the supplementary material." |
|  | 10b | List and define all other variables for which data were sought (e.g. participant and intervention characteristics, funding sources). Describe any assumptions made about any missing or unclear information. | Detailed in the “Data Items” section of the Supplementary Material: "title, abstract, authorship, scientific journal of publication, year of publication, link... clinical application, and the clinical domain..." |
| Study risk of bias assessment | 11 | Specify the methods used to assess risk of bias in the included studies, including details of the tool(s) used, how many reviewers assessed each study and whether they worked independently, and if applicable, details of automation tools used in the process. | Presented in “Evidence Appraisal” section of Methods: “We applied the PROBAST+AI tool35 to the 25 most cited included research papers. For each paper, up to two models were considered, in order of presentation. Due to their inherent limitations, we opted to exclude TRIPOD Type 1a (i.e., all data used for model development without validation) and Type 1b articles (i.e., all data used for model development, evaluation using resampling). Using an approximation of the relative prevalence of the remaining TRIPOD types, 15 Type 2a articles, 5 Type 2b articles and 5 Type articles were included. Each article and its corresponding appraisals were conducted by a single reviewer.” |
| Effect measures | 12 | Specify for each outcome the effect measure(s) (e.g. risk ratio, mean difference) used in the synthesis or presentation of results. | “The primary effect measures were the performance metrics values of the decentralized learning models and their non-decentralized counterparts. These values were extracted directly from the included studies. To explore non-parametric effect sizes the Wilcoxon two-sample paired signed-rank test were used, comparing the distributions of the individual performance comparisons. Estimates of effect sizes and their respective magnitude are presented.” |
| Synthesis methods | 13a | Describe the processes used to decide which studies were eligible for each synthesis (e.g. tabulating the study intervention characteristics and comparing against the planned groups for each synthesis (item #5)). | Described in the “Synthesis Methods” section: “Data collected were grouped by each performance metric and divided in the classes of the following variables: decentralized learning architecture, larger clinical domain and clinical application. Individual performance metrics with at least 30 comparisons collected were explored. An online dashboard was produced to allow for a customized search of relevant performance comparisons, using the Shiny R package – https://jmdiniz.shinyapps.io/phdiniz_systematic_review_analysis/.  The distribution of individual model performance differences between decentralized and non-decentralized alternatives across the difference performance metrics is presented using histograms and calculating their median difference, the 25th percentile and the 75th percentile, as well as the bootstrapped 95% Confidence Intervals, based on 10.000 simulations. For sensitivity analysis, variations of these histograms are produced without the contributions of the study with the most observations – available in the Supplementary Material. Specific detailed syntheses were produced for performance metrics-larger clinical domain-clinical application combinations, for instances with at least 10 comparisons and featuring at least 5 different studies.” |
|  | 13b | Describe any methods required to prepare the data for presentation or synthesis, such as handling of missing summary statistics, or data conversions. | Presented in the “Synthesis Methods” section: "The only data processing concerned the conversion of values presented in percentages in some instances.” |
|  | 13c | Describe any methods used to tabulate or visually display results of individual studies and syntheses. | Described in the “Synthesis Methods” section: “Data collected were grouped by each performance metric and divided in the classes of the following variables: decentralized learning architecture, larger clinical domain and clinical application. Individual performance metrics with at least 30 comparisons collected were explored. An online dashboard was produced to allow for a customized search of relevant performance comparisons, using the Shiny R package – https://jmdiniz.shinyapps.io/phdiniz_systematic_review_analysis/.  The distribution of individual model performance differences between decentralized and non-decentralized alternatives across the difference performance metrics is presented using histograms and calculating their median difference, the 25th percentile and the 75th percentile, as well as the bootstrapped 95% Confidence Intervals, based on 10.000 simulations. For sensitivity analysis, variations of these histograms are produced without the contributions of the study with the most observations – available in the Supplementary Material. Specific detailed syntheses were produced for performance metrics-larger clinical domain-clinical application combinations, for instances with at least 10 comparisons and featuring at least 5 different studies.” |
|  | 13d | Describe any methods used to synthesize results and provide a rationale for the choice(s). If meta-analysis was performed, describe the model(s), method(s) to identify the presence and extent of statistical heterogeneity, and software package(s) used. | In “Effect Measures”: "To explore non-parametric effect sizes the Wilcoxon two-sample paired signed-rank test were used, comparing the distributions of the individual performance comparisons. Estimates of effect sizes and their respective magnitude are presented."  In “Synthesis Methods”: "Due to the heterogeneity in the data collected, no meta-analysis was conducted." The authors chose descriptive synthesis approaches due to data heterogeneity. |
|  | 13e | Describe any methods used to explore possible causes of heterogeneity among study results (e.g. subgroup analysis, meta-regression). | Described in the “Synthesis Methods” section: "Data collected were grouped by each performance metric and divided in the classes of the following variables: decentralized learning architecture, larger clinical domain and clinical application."  Described in the “Detailed Absolute and Relative Differences Analysis” section of Results and presented in the Supplementary Material: "For each comparison and metric pairing, data was segmented into 10 equal-width intervals based on the range of the decentralized model performance. Within each segment, decentralized models were compared to their counterparts, based on the paired performance comparisons." |
|  | 13f | Describe any sensitivity analyses conducted to assess robustness of the synthesized results. | Described in the “Synthesis Methods” section and presented in the Supplementary Material: "For sensitivity analysis, variations of these histograms are produced without the contributions of the study with the most observations -- available in the Supplementary Material."  In addition: "Our sensitivity analysis, excluding observations from articles with the most comparisons, revealed two patterns based on the number of models considered. For metrics with more than 20 different models (accuracy, AUROC, Dice score, F1 score, precision, sensitivity, and specificity), variations in favorability ratios were generally within single-digit percentage points." |
| Reporting bias assessment | 14 | Describe any methods used to assess risk of bias due to missing results in a synthesis (arising from reporting biases). | "No specific efforts were made to assess the risk of bias due to missing results arising from reporting biases for the syntheses produced" |
| Certainty assessment | 15 | Describe any methods used to assess certainty (or confidence) in the body of evidence for an outcome. | Described in the “Synthesis Methods” section: “The distribution of individual model performance differences between decentralized and non-decentralized alternatives across the difference performance metrics is presented using histograms and calculating their median difference, the 25th percentile and the 75th percentile, as well as the bootstrapped 95% Confidence Intervals, based on 10.000 simulations.” |
| **RESULTS** | | |  |
| Study selection | 16a | Describe the results of the search and selection process, from the number of records identified in the search to the number of studies included in the review, ideally using a flow diagram. | From the Results: "Our systematic review identified a total of 165,010 studies. Figures 1 and 2 describe the phases 1 and 2 of the identification, screening, and selection processes." |
|  | 16b | Cite studies that might appear to meet the inclusion criteria, but which were excluded, and explain why they were excluded. | From the Results: Figures 1 and 2 (PRISMA flow diagrams) present numbers and reasons for exclusion shown in flow diagrams. |
| Study characteristics | 17 | Cite each included study and present its characteristics. | Presented in “Table 1 - Characteristics of included studies” |
| Risk of bias in studies | 18 | Present assessments of risk of bias for each included study. | Described in “Evidence Appraisal” section and summarised in Figures 26-29: "We used the PROBAST+AI tool to appraise the most cited articles... Findings are presented in Figures 26 to 29." |
| Results of individual studies | 19 | For all outcomes, present, for each study: (a) summary statistics for each group (where appropriate) and (b) an effect estimate and its precision (e.g. confidence/credible interval), ideally using structured tables or plots. | Presented in Figures 4 to 17: Performance distributions shown across multiple figures with detailed statistical summaries in each caption. |
| Results of syntheses | 20a | For each synthesis, briefly summarise the characteristics and risk of bias among contributing studies. | Presented in “General Results Summary” and “Evidence Appraisal” sections of the Results. |
|  | 20b | Present results of all statistical syntheses conducted. If meta-analysis was done, present for each the summary estimate and its precision (e.g. confidence/credible interval) and measures of statistical heterogeneity. If comparing groups, describe the direction of the effect. | Presented in Figures 4 to 17 and Figure 18 (Effect sizes) in the Results. In addition, “Wilcoxon signed-rank test results with confidence intervals for all performance metrics.” |
|  | 20c | Present results of all investigations of possible causes of heterogeneity among study results. | Presented in the “Detailed Absolute and Relative Differences Analysis” section of the Results: "For each comparison and metric pairing, data was segmented into 10 equal-width intervals based on the range of the decentralized model performance... This pattern is most evident in accuracy measurements, where centralized and decentralized approaches demonstrate similar performance trajectories, particularly in the upper performance range (80% to 100%)."  Presented in the Tables 6 and 7 of the Results: Performance by clinical domain, application, and metric combinations with minimum thresholds for reporting. |
|  | 20d | Present results of all sensitivity analyses conducted to assess the robustness of the synthesized results. | Detailed in Supplementary Material: “For sensitivity analysis, variations of these histograms are produced without the contributions of the study with the most observations – available in the Supplementary Material.”  In addition: "Our sensitivity analysis, excluding observations from articles with the most comparisons, revealed two patterns based on the number of models considered. For metrics with more than 20 different models (accuracy, AUROC, Dice score, F1 score, precision, sensitivity, and specificity), variations in favorability ratios were generally within single-digit percentage points. For metrics with fewer models (Hausdorff Distance, Jaccard score, Matthews Correlation Coefficient, and negative predictive value), changes were more substantial, ranging from a 2-percentage point reduction to a 34-percentage point increase." |
| Reporting biases | 21 | Present assessments of risk of bias due to missing results (arising from reporting biases) for each synthesis assessed. | "No specific efforts were made to assess the risk of bias due to missing results arising from reporting biases for the syntheses produced" |
| Certainty of evidence | 22 | Present assessments of certainty (or confidence) in the body of evidence for each outcome assessed. | Presented in Figures 4 to 17: Performance distributions shown across multiple figures with detailed statistical summaries in each caption, including bootstrapped 95% Confidence Intervals, based on 10.000 simulations. |
| **DISCUSSION** | | |  |
| Discussion | 23a | Provide a general interpretation of the results in the context of other evidence. | Described in the paragraphs 1 to 4 of the Discussion: "This systematic review provides the most comprehensive analysis to date... Our findings reveal clear patterns in the relative performance..." |
|  | 23b | Discuss any limitations of the evidence included in the review. | Described in the following paragraphs of the Discussion: "Despite the robustness of this review..."; "Many included studies rely on secondary data or inadequately detailed primary data collection methods..." |
|  | 23c | Discuss any limitations of the review processes used. | Described in the following paragraphs of the Discussion: "Our methodology offers several strengths..."; "However, we did not examine grey literature or publications outside primary scientific articles, and no tailored investigation of publication bias was conducted." |
|  | 23d | Discuss implications of the results for practice, policy, and future research. | Described in the following paragraphs of the Discussion: "These findings have important implications..."; "For specific applications such as COVID-19 and cancer diagnosis... Future research should prioritize higher methodological quality..." |
| **OTHER INFORMATION** | | |  |
| Registration and protocol | 24a | Provide registration information for the review, including register name and registration number, or state that the review was not registered. | Detailed in the “Registration and protocol” section: “The research protocol for this study was published, on June 6th, 2023. It was previously registered with PROSPERO, under the number 393126, on February 3rd, 2023, and accessible through https://www.crd.york.ac.uk/prospero/display_record.php?ID=CRD42023393126.  Details about the changes made to the protocol, and the rational used, are presented in the Supplementary Material.” |
|  | 24b | Indicate where the review protocol can be accessed, or state that a protocol was not prepared. | Detailed in the “Registration and protocol” section: “The research protocol for this study was published, on June 6th, 2023. It was previously registered with PROSPERO, under the number 393126, on February 3rd, 2023, and accessible through https://www.crd.york.ac.uk/prospero/display_record.php?ID=CRD42023393126.  Details about the changes made to the protocol, and the rational used, are presented in the Supplementary Material.” |
|  | 24c | Describe and explain any amendments to information provided at registration or in the protocol. | Detailed in the “Details about the changes made to the protocol, and the rational used, are presented in the Supplementary Material.” |
| Support | 25 | Describe sources of financial or non-financial support for the review, and the role of the funders or sponsors in the review. | Presented in the “Acknowledgements” section: "Some authors... were researchers of the 'Secur-e-Health: Privacy preserving cross-organizational data analysis...' with the reference NORTE-01-0247-FEDER-181418. The funding agency did not have a role in either the study design..." |
| Competing interests | 26 | Declare any competing interests of review authors. | Presented in the “Conflicts of Interest” section: "The authors declare they have no known competing interests (financial, personal, or otherwise) that could have influenced the work reported in this paper." |
| Availability of data, code and other materials | 27 | Report which of the following are publicly available and where they can be found: template data collection forms; data extracted from included studies; data used for all analyses; analytic code; any other materials used in the review. | Presented in the “Data Availability” section: "A dashboard for select metrics are made available. Detailed data extracted from the included studies, including data used for analyses, the data processing and analytic code, is made available upon request." |
